# A cosmopolitan fungal pathogen of dicots adopts an endophytic lifestyle on cereal crops and protects them from major fungal diseases

**DOI:** 10.1038/s41396-020-00744-6

**Published:** 2020-08-19

**Authors:** Binnian Tian, Jiatao Xie, Yanping Fu, Jiasen Cheng, Bo Li, Tao Chen, Ying Zhao, Zhixiao Gao, Puyun Yang, Martin J. Barbetti, Brett M. Tyler, Daohong Jiang

**Affiliations:** 1 State Key Laboratory of Agricultural Microbiology, Huazhong Agricultural University, Wuhan, China; 2 The Provincial Key Lab of Plant Pathology of Hubei Province, Huazhong Agricultural University, Wuhan, China; 3 National Agro-Technical Extension and Service Center, Ministry of Agriculture and Rural Affairs, Beijing, China; 4 The UWA School of Agriculture and Environment and The UWA Institute of Agriculture, The University of Western Australia, Crawley, WA Australia; 5 Center for Genome Research and Biocomputing and Department of Botany and Plant Pathology, Oregon State University, Corvallis, Oregon 97331 USA; 6 Present Address: Research and Extension Unit, Agriculture and Consumer Protection Department, Food and Agriculture Organisation of the United Nations, Rome, Italy

**Keywords:** Fungal ecology, Microbial ecology

## Abstract

Fungal pathogens are seriously threatening food security and natural ecosystems; efficient and environmentally friendly control methods are essential to help safeguard such resources for increasing human populations on a global scale. Here, we find that *Sclerotinia sclerotiorum*, a widespread pathogen of dicotyledons, can grow endophytically in wheat, rice, barley, maize, and oat, providing protection against *Fusarium* head blight, stripe rust, and rice blast. Protection is also provided by disabled *S. sclerotiorum* strains harboring a hypovirulence virus. The disabled strain DT-8 promoted wheat yields by 4–18% in the field and consistently reduced *Fusarium* disease by 40–60% across multiple field trials. We term the host-dependent trophism of *S. sclerotiorum*, destructively pathogenic or mutualistically endophytic, as schizotrophism. As a biotroph, *S. sclerotiorum* modified the expression of wheat genes involved in disease resistance and photosynthesis and increased the level of IAA. Our study shows that a broad-spectrum pathogen of one group of plants may be employed as a biocontrol agent in a different group of plants where they can be utilized as beneficial microorganisms while avoiding the risk of in-field release of pathogens. Our study also raises provocative questions about the potential role of schizotrophic endophytes in natural ecosystems.

## Introduction

More than 80% of crop diseases are caused by fungi or fungus-like pathogens that threaten food security. Many important plant diseases are recorded as leading to famine, human health problems, ecosystem devastation, or even a changed course of human history [1]. Furthermore, many fungal pathogens also produce mycotoxins, such as ergotamine, deoxynivalenol (DON), and aflatoxin that further threaten human and livestock health [2, 3]. Cereal crops provide a major food source for human beings and animals. However, they are heavily attacked by fungal pathogens, such as the rice blast fungus *Magnaporthe oryzae*, wheat rust fungi *Puccinia* spp, and the wheat head blight fungus *Fusarium graminearum* [4]. Emergence of novel fungal pathogens can threaten food supplies and/or ecological environments [5]. For example, strain Ug99 of the wheat stem rust fungus [6] and the recently emerged wheat blast fungus [7] threaten wheat crops that support ~20% of human food consumption [8]. Successful control of fungal diseases is challenging as fungi can easily overcome host vertical (i.e., major gene) resistance. Widespread use of fungicides on crops pollutes the environment and food. Furthermore, fungal pathogens can quickly develop effective resistance against fungicides [9]. Together, these challenges motivate the development of novel fungal crop disease control methods that are highly efficient, persistent, and environmentally friendly.

Biological control of fungal diseases is one such approach that offers much toward increasing world crop production. However, for more than 100 years since the first paper on biological control of plant diseases appeared [10], only very few fungal species have been developed successfully as biological control agents [11, 12]. One important limitation is that beneficial microbes that can be successfully deployed in the field are very rare. New ideas and methods are required if we are to successfully identify and deploy beneficial microbes, despite a wide range of microbes being available for screening and testing. Largely ignored in this search for new beneficial microbes have been plant pathogens, despite being common and successfully adapted to survive in nature, likely because the approach seems quite counter-intuitive.

*S. sclerotiorum* destroys many economically important dicotyledonous crops, including numerous leguminous and brassicaceous crops such as soybean and rapeseed (*Brassica napus*) [13–15]. Wheat and other cereal crops are generally considered nonhosts of *S. sclerotiorum* and are widely used in rotation with rapeseed and other susceptible host crops [16–20]. Due to these rotations, we assessed wheat leaves and found by PCR amplification that *S. sclerotiorum* occurred on wheat leaves. This led us to hypothesize that *S. sclerotiorum* may, in fact, grow inside wheat plants. In this research, we evaluate the potential impact of *S. sclerotiorum* on wheat growth and disease resistance, and also examine the endophytic growth of *S. sclerotiorum* on other cereal crops, such as rice, barley, maize, and oats.

## Materials and methods

### Plant and fungal materials, maintenance, and preparation

The winter wheat cultivar Zheng 9023 and the spring wheat cultivar Yongliang 4 were purchased from the commercial seed market in Wuhan City and Minqin County in China, respectively. The barley cultivar Huadamai 14 was provided from Prof. Dongfa Sun in Huazhong Agricultural University, Wuhan, China. The oat cultivar Mengmai 2 was donated by Prof. Jun Zhao in Neimeng Agricultural University. The maize cultivar Zhengdan 958 was purchased from the commercial seed market in Wuhan City. The rice cultivar LTH was donated by Prof. Youliang Peng of China Agricultural University. All seeds were surface-sterilized with a 0.5% sodium hypochlorite solution (NaClO) before sowing or *S. sclerotiorum* treatment.

*S. sclerotiorum* strain DT-8, which was originally isolated from a sclerotium collected from a diseased rapeseed, is a hypovirulent strain infected by a DNA mycovirus. Strain DT-8VF, a virulent strain, is a virus-free derivative of DT-8 [21]. Strain DT-8VF^RFP^ is a derivative of DT-8VF labeled with the mCherry fluorophore by DNA transformation; it shows normal virulence. The wheat *Fusarium* head blight (FHB) pathogen, *F. graminearum* strain PH-1, was used to inoculate wheat spikes. An *S. sclerotiorum* virulent strain Ep-1PNA367 and three hypovirulent strains, AH98, SCH941, and T1-1-20, were also used to investigate their potential endophytic growth on wheat. AH98 is infected by a negative-stranded RNA virus [22], while SCH941 and T1-1-20 are infected by various other mycoviruses. All *S. sclerotiorum* strains and *F. graminearum* strain PH-1 were grown on potato dextrose agar (PDA) or potato dextrose broth (PDB) at 20 °C; *Magnaporthe oryzae* strain 131 was grown on PDA at 28 °C, or grown on tomato–oat medium to produce conidia, and was stored on PDA slants at 4 °C.

### Microscopic observation

To observe the growth of *S. sclerotiorum* in wheat by confocal microscopy, seeds were surface-sterilized with NaClO and sown on half-strength Murashige and Skoog (MS) agarose medium amended with 25 mM sucrose for 8 days, then root crowns were inoculated with mycelia of the strain DT-8VF^RFP^. Wheat seedlings were maintained at 20 °C for 4 days under a 12-h photoperiod. Seedlings were subsequently washed three times with PBS for 10 min each, and then root sections were separated and incubated in a 1:100 dilution of wheat germ agglutinin conjugated to FITC (Sigma) for 1 h at room temperature according to the manufacturer’s instructions. The roots were visualized with a LEICA confocal microscope (LEICA SP8) using the 488-nm line of a 25-mW Argon ion laser for FITC and the 561-nm line of a 20-mW solid-state laser for mCherry.

For further observation with a transmission electron microscopy (TEM), 5-mm root segments from wheat seedlings grown on MS medium for 15 days after inoculation with strain DT-8VF^RFP^ were fixed in 0.4% (v/v) glutaraldehyde solution overnight at 4 °C. After washing in PBS buffer, roots were dehydrated with a graded ethanol series. Samples were then embedded in Epon-821 and polymerized at 60 °C. Thin sections (50 nm) were cut using a Leica ULTRACUT UCT ultramicrotome with a diamond knife.

For TEM immunodetection, wheat seedling roots were fixed in 4% (w/v) freshly depolymerized paraformaldehyde and 0.4% (v/v) glutaraldehyde in 1× PBS, pH 7.4, for 1 h at 4 °C. The samples were then embedded using an LR white embedding kit (Fluka) and polymerized at 50 °C for 24 h. Immunogold labeling specificity was detected by displacing the anti-mCherry antibodies with rabbit preimmune serum. The method for TEM immunodetection was performed as previously described [23].

For scanning electron microscopy (SEM) observation, 2-mm root segments from the wheat, barley, oat, maize, and rice seedlings treated with different strains of *S. sclerotiorum* grown on MS medium for 15 days were used. All segments were fixed in a 0.4% (v/v) glutaraldehyde solution overnight at 4 °C. For SEM analysis, the sections were allowed to air-dry overnight in a desiccator at room temperature, sputter-coated with gold, and prepared for SEM analysis (EVO MA 10 Carl Zeiss SMT AG, Germany). Root segments from nontreated wheat were sampled and observed as a control comparison.

For confocal microscopy observations, 45 days after the root crown inoculation with mycelia of the DT-8VF^RFP^ strain, stems from wheat plants growing in soil in the greenhouse were carefully washed with distilled water and embedded in Tissue-Tek O.C.T. compound medium (Sakura Finetek USA, Inc., Torrance, CA) at −23 °C overnight. Microtome sections (25 µm thick) were sliced using a freezing microtome (LEICA SP8, Germany). The stem microtome sections were then visualized with a LEICA confocal microscope using the 561-nm excitation wavelength for mCherry.

### Re-isolation of *S. sclerotiorum*

To further probe whether *S. sclerotiorum* can go to aerial parts of wheat when inoculated on root of wheat seedling, plants were grown in sterile nutrient soil for 45 days. The samples for re-isolation were taken from the second segment near the base of wheat stem of eleven individual DT-8VF^RFP^*-*treated wheat plants, and were cut into 5 mm long segments, then surface-sterilized by dipping them in 70% EtOH for 2 min and then in 0.5% NaClO for 2 min, followed by rinsing three times with sterile distilled water. The sterilized stem segments were placed on hygromycin-amended PDA medium plates (50 µg/mL) and incubated at 20 °C for 8 days. Then, the emerging colonies were identified as *S. sclerotiorum* based on colony morphology, and PCR amplification [24].

### PCR determination of *S. sclerotiorum* and mycoviruses

DNA samples of either wheat or fungi were extracted using a cetyltrimethylammonium bromide method. A primer pair XJJ21/XJJ222 (GTTGCTTTGGCGTGCTGCTC/CTGACATGGACTCAATACCAATCTG) was used for detection of *S. sclerotiorum* [24], and a primer pair pRep-F/pRep-R (GTCACCACCCAAACATTACAAAGAGCGTATTCC/ACGTCA GGTGC) was used for detection of viral DNA of SsHADV-1. A procedure described by Yu et al. [21] was used for PCR amplification.

### Seed treatment of wheat with *S. sclerotiorum* and sowing

To determine whether *S. sclerotiorum* could promote growth and disease resistance of wheat in the greenhouse, wheat seeds were washed with tap water and surface-sterilized with 0.5% NaClO for 10 min, then washed three times with sterile water. Surface-sterilized seeds were soaked in sterile water for 4 h, and then collected and blotted dry. Meanwhile, fresh mycelium of strains DT-8 and DT-8VF were collected from PDA medium and then cultured in PDB medium in 250-mL flasks in a shaker at 20 °C for 4 days to obtain hyphal fragment suspensions; the number of viable fungal fragments was adjusted to 1.4 × 10^5^ cfu/mL before inoculation of wheat seeds. The hyphal fragment suspensions were then used to inoculate the prepared seeds (100 mL of hyphal fragment suspension/kg wheat seeds) by thoroughly mixing the hyphal fragment suspension and wheat seeds for 6 h. The inoculated seeds were further dried using an electric fan for 12 h at room temperature. Wheat seeds soaked only in sterile water for 6 h and then dried with the same method were used as a control.

To test whether the treated seeds were successfully colonized by *S. sclerotiorum*, *S. sclerotiorum*-treated seeds were surface-sterilized with 0.5% NaClO for 10 min, rinsed three times with sterile distilled water, and then cut in half and placed on PDA plates and incubated at 20 °C for 7 days. All emerging colonies from *S. sclerotiorum*-treated seeds were confirmed as *S. sclerotiorum* based on colony morphology and PCR amplification. In addition, a small number of *S.* sclerotiorum-treated seeds were randomly picked and sown into soil taken from the field and grown in the greenhouse. The seedlings were then tested after 21 days for the presence of *S. sclerotiorum* by PCR amplification; all seedlings tested positive. Hence, the seed treatment was confirmed as an efficient method for inoculation of wheat seeds with *S. sclerotiorum*. Consequently, we used this method to treat wheat seeds for the rest of the study, and kept treated seeds under dry conditions at room temperature for up to 7 days before sowing.

Seeds treated either with strains DT-8VF or DT-8 were sown in pots in a greenhouse. In a laboratory test, treated seeds were allowed to germinate in a Petri dish on a layer of wet filter paper. Germinating seeds were then sown either into soil that was taken from the field or sterile nutrient soil. Twelve plants were grown in each pot. Wheat seedlings were maintained under greenhouse conditions at 20 °C. Strain DT-8-treated seeds were also tested in the field. Those seeds were sown as rows in the wheat field in exactly the same way as farmers normally do in each sowing season. Field management was conducted as per normal farmer practice, except that no fungicide was applied.

In order to investigate whether *S. sclerotiorum* colonization can spread to aerial parts of wheat plants originating from DT-8-treated seeds, ten plants from each plot were randomly sampled from the field. A total of 30 wheat plants from the strain DT-8-treated group and 20 plants from the nontreated group were sampled. First, the roots were rinsed thoroughly with tap water. Then the roots, flag leaves, and spikes were given three brief rinses in distilled water. Each wheat plant was given a number, from 1 to 30 for DT-8-inoculated plants, and from 1 to 20 for nontreated plants. DNA samples were extracted individually from the root, flag leaf, and spike of each plant and used to determine the presence of *S. sclerotiorum* and mycovirus.

### Evaluating the growth of treated wheat in the greenhouse and field

To evaluate the growth of *S. sclerotiorum*-treated wheat in greenhouse experiments, plant height was measured at the seedling and anthesis stages, while flag leaf and spike lengths were evaluated only at the anthesis stage. There were 60 plants in each group treated with strain DT-8 and strain DT-8VF and 60 plants in the nontreated group. Determination of 1000-grain weight was repeated, four in each group. Measurement data for each group were calculated for statistical analysis.

To investigate whether other strains could promote wheat growth, strains Ep-1PNA367, AH98, SCH941, and T1-1-20 were used instead of strains DT-8 and DT-8VF. Wheat seeds were treated and then sown in a sterile mixture of vermiculite and perlite at the ratio of 3:1 in pots and placed in greenhouse, with ~50 seedlings in each pot. Seedling shoot fresh weight was measured at 25 days after planting. There were 30 plants in each treatment and control, and the average weight of ten seedlings was calculated.

For field tests, plant height and the length, width, and thickness of flag leaves at the early flowering stage were measured in the field at EZhou. Forty plants from each plot were randomly measured from a total of 120 plants in the DT-8-treated group and from 120 plants in the nontreated group. Measurement data for each group were calculated for statistical analysis. All the results were confirmed with independent lines and over two planting seasons.

### Field experiments and wheat yield tests

To examine whether *S. sclerotiorum* treatment could enhance wheat yield under natural field conditions, DT-8-treated seeds were sown in a wheat field located at EZhou in late October of 2016 and harvested in mid-May of 2017. This experiment was repeated at EZhou, Jingmen City, and Xiangyang City in late October of 2017 and 2018 and harvested in mid-May of 2018 and 2019. Furthermore, seeds of the spring wheat cultivar Yongliang 4 were also treated with strain DT-8 and sown in mid-April of 2017 at Minqin and Tianzhu Counties in Gansu province and harvested in late July of 2017. All wheat was managed as per normal farmer practice, except that no fungicide was applied. The treatments with or without strain DT-8 were replicated four times at Tianzhu County and Jingmen City in 2017 and five times at other places and the wheat yield from 5 m^2^ was measured in each plot and used for statistical analysis.

### Analysis of chlorophyll content and photosynthetic rate in flag leaves

To determine chlorophyll content, leaf tissues were harvested using a circular punch that yields 0.5-cm diameter leaf discs. There were four flag leaf replicates for each treatment. Chlorophyll was extracted from wheat flag leaves obtained from the field at EZhou using 95% (v/v) ethanol (analytically pure, Sinopharm Chemical Reagent Co., Ltd) and the extracted chlorophyll concentration was determined using a spectrophotometer (UV2102, Unico, Shanghai, China) [25].

For the photosynthetic rate, flag leaf samples were obtained from the field at EZhou. Each treatment had three flag leaf replicates. Photosynthetic rate determination was performed as previously described [26].

### Assay of plant hormones

Five frozen flag leaf and spike replicates from each treatment (~100 mg for each flag leaf and spike sample) were ground to a fine powder in liquid nitrogen using a mortar and pestle. Each sample was weighed into a 1.5-mL tube, mixed with 750 μL of cold extraction buffer (methanol: water: acetic acid, 80:19:1, v/v/v) supplemented with internal standards, 10 ng of 2H_6_ABA, 10 ng of DHJA, and 5 μg of NAA, vigorously shaken on a shaking bed for 16 h at 4 °C in the dark, and then centrifuged at 12,000 rpm for 15 min at 4 °C. Supernatant was carefully transferred to a new 1.5-mL tube and pellets remixed with 400 μL of extraction buffer, shaken for 4 h at 4 °C, and centrifuged. The two supernatants were combined and filtered using a syringe-facilitated 13-mm diameter nylon filter with a pore size of 0.22 μm (Nylon 66; Jinteng Experiment Equipment Co., Ltd, Tianjing, China). The filtrate was dried by evaporation under nitrogen gas flow for ~5 h at room temperature and then dissolved in 200 μL of methanol. Aliquots of dissolved samples were further diluted 40 times using methanol for jasmonic acid (JA), abscisic acid (ABA), and indole-3-acetic acid (IAA) quantification. Liquid chromatography was carried out using an ultrafast liquid chromatography with an autosampler (Shimadzu Corporation, Kyoto, Japan). The method used for hormone determination was as previously described [27].

### Inoculation of *F. graminearum* and rating of disease

Infection assays on flowering wheat spikes were performed as previously described [28]. At the early flowering stage, a conidial suspension of *F. graminearum* strain PH-1 was collected from 5-day-old cultures growing in carboxymethylcellulose medium, then filtered through three layers of lens-wiping paper and then mixed with 0.01% (v/v) Tween 20. Ten microliters of 1 × 10^5^ conidia mL^−1^ conidial suspension was inoculated individually onto the fourth spikelet from the bottom using a micropipette. The inoculated wheat spikes were maintained at a relative humidity of 95% for 72 h. Symptomatic spikes were examined and images captured after 14 days.

For the greenhouse test, 15 spikes from each treatment were inoculated and the spikelet infection rate of each spike was calculated; then, the average spikelet infection rate for each treatment was calculated for statistical analysis. For the field test, ten spikes from each plot were inoculated from a total of 30 inoculated spikes in the strain DT-8-treated plots and 30 spikes in the nontreated plots. The spikelet infection rate for each plot was calculated and the average spikelet infection rate for strain DT-8 treatment and nontreated control were then calculated for statistical analysis. The field test was conducted twice, once in 2017 and repeated in 2018.

### FHB survey in a natural, noninoculated field

To investigate natural FHB infection in strain DT-8-treated wheat, an FHB field survey was conducted in experimental fields located at EZhou City, Jingmen City, and Xiangyang City in 2018. The field survey protocol described by the National Agricultural Technology Extension Service Center of China was adopted for the FHB survey with minor modifications. A total of 500 spikes were sampled randomly in each plot; in total, 1500 spikes were collected from DT-8-treated plots, with the same number of spikes being collected from nontreated plots to calculate disease incidence (spikelet infection rate) and severity (disease index). The number of infected and noninfected spikelets on each spike was counted and the average spikelet infection rate for each plot was calculated and used for statistical analysis. To calculate the disease index, the infected spikes were divided into five grades, namely: grade 0, no spikelet was infected; grade 1, the spike was infected, but less than 25% of spikelets were infected; grade 2, more than 25%, but less than 50%, of the spikelets were infected; grade 3, more than 50%, but less than 75%, of the spikelets were infected; and grade 4, more than 75% of the spikelets were infected. Finally, the disease severity for each plot was calculated using a formula for disease index, DI = ∑(*nX*/4 *N*) × 100, where “*X*” is the scale value of each spike, “*n*” is the number of spikes in the category, and “*N*” is the total number of spikes assessed for each plot. The disease index for each group was used for statistical analysis.

### Inoculation of *M. oryzae* on barley and rice

To probe if *S. sclerotiorum* could enhance resistance against the rice blast fungus (*M. oryzae*) in barley and rice, an inoculation test was carried out according to a method described by Kong et al. [29]. Conidia of *M. oryzae* were collected with sterile water from 4-day-old cultures growing on tomato–oat medium and then filtered through three layers of lens-wiping paper. Infection assays were performed in whole plant leaves by spray inoculation using an airbrush nebulizer compressor. Strain DT-8-treated seedlings, which were grown in a sterile mixture of vermiculite and perlite at the ratio of 3:1 in pots and placed in greenhouse for 9 days for barley at 20 °C and 20 days for rice at 28 °C, were sprayed with a conidial suspension [10^5^ conidia/mL mixed with 0.02% (v/v) Tween 20], using 4 mL suspension for each pot. The plants were further incubated at 28 °C, 80% relative humidity, under a 16 h light/8 h darkness photoperiod. Then, we assessed the presence of *S. sclerotiorum* in aerial parts of DT-8-treated barley and rice by PCR amplification in greenhouse plants; 92% of samples were confirmed as positive for *S. sclerotiorum*. For barley, lesions of leaves with the same leaf age (bottom leaves) were examined and typical infected leaves were photographed with a digital camera at 5 days post inoculation (dpi); for rice, the leaves were examined and photographed at 7 dpi.

### Detection of toxins (DON)

To assay point-inoculated spikelets from strain DT-8-treated and nontreated plants, each sample was placed in a 50-mL centrifuge tube and mixed with 400 μL of a mixed isotope internal standard. The solution was remixed with 20 mL of an acetonitrile–water solution after standing for 30 min, vigorously shaken on a shaking bed for 4 h at 4 °C, and then centrifuged at 10,000 rpm for 5 min. The supernatants were carefully transferred to a new tube. The supernatants were combined and filtered using a syringe-facilitated 13-mm diameter nylon filter with a pore size of 0.22 μm (Nylon 66; Jinteng Experiment Equipment Co., Ltd, Tianjing, China). The filtrate was dried by evaporation under the nitrogen gas flow for ~5 h at room temperature, and then dissolved in 200 μL of methanol. The method for DON determination was performed as described by the National Agricultural Technology Extension Service Center of China (GB5009.111-2016).

### RNA sequencing and analysis

Sterilized wheat seeds were inoculated with strain DT-8, and then were sown in experimental fields located at EZhou City in 2017. Wheat flag leaves and spikes were collected from this field during the initial bloom stage, with three spikes and leaves being randomly sampled from each of the three replicate plots. The flag leaves and spikes of nontreated wheat plants in the same field were randomly taken from each of the three control replicate plots. The samples were immediately placed in liquid nitrogen and ground into powder. Total RNA samples were extracted with a TRIzol Plus RNA Purification Kit (Takara, Dalian, China) and treated with RNase-free DNase I (Takara, Dalian, China) according to the manufacturer’s instructions. The RNA quality was checked using a Nanodrop Spectrophotometer (Thermo Fisher Scientific Inc., Wilmington, DE, USA). Then, mRNA was enriched with magnetic beads Oligo (dT) (TransGen Biotech, Beijing, China). Subsequently, cDNA was synthesized using the mRNA as template. The cDNA fragments were linked with adapters, and suitable fragments were selected for PCR amplification. Agilent 2100 Bioanalyzer and ABI StepOnePlus Real-Time PCR Systems were used in the quantification and qualification of the sample library. Subsequently, the library was sequenced for raw data using an Illumina HiSeq X sequencer at BGI (The Beijing Genomics Institute, China). Then, adapters, low-quality sequences, and reads with high content of unknown base (N) reads were removed to obtain clean reads. The clean reads were then mapped to the wheat genome or *S. sclerotiorum* genome and the sequence results evaluated in terms of read quality, alignment, saturation, and the distribution of reads on reference genes [30]. Mismatches of no more than two bases were accepted in the alignment. Gene expression was calculated by the number of reads mapped to the reference genomes using the fragments per kilobase of transcript per million mapped reads method [31]. Subsequently, differentially expressed genes (DEGs) were selected with FDR < 0.01 and |log2 ratio| ≥ 1 between the *S.* sclerotiorum-treated and nontreated group. Gene Ontology (GO) enrichment analyses of all genes were performed to examine the biological significance of the genes, and GO enrichment analysis with Fisher’s exact tests were performed on differentially expressed transcripts, using *p* values < 0.01. GO-Slim was run to reduce complexity of GO terms for gene class analysis. Based on GO-Slim annotations, transcripts were classified into three ontological categories: cellular component, biological process, and molecular function [32]. All genes were mapped onto the databases of Kyoto Encyclopedia of Genes and Genomes pathway for pathway annotation [33]. The RNA-seq data used in this study were deposited in the NCBI GEO database with accession no. PRJNA546228.

### Statistical analyses

For all experiments that generated quantitative data, biological replicates were used and the mean values are presented with bars representing the standard deviations (SD). Statistical analyses were performed on the data using the unpaired Student’s *t* test. The SPSS (SPSS 19) statistical software was used for these analyses.

## Results

### *S. sclerotiorum* grows endophytically in cereal crops

In the field, ascospores of *S. sclerotiorum* or hyphae from germinating sclerotia in the soil may contact rapeseed or nonhost plants such as wheat as they are often together in the same growing season. Hence, we collected foliage of wheat seedlings in early December 2015 from a field located in Dangyang County, Hubei province, PR China (Supplementary Fig. 1a, b) and successfully detected *S. sclerotiorum* in most tested wheat plants using PCR amplification with an *S. sclerotiorum*-specific primer pair (Supplementary Fig. 1c). Wheat appeared to be a nonhost of *S. sclerotiorum*, as the fungus neither killed wheat seedlings nor caused lesions on healthy plants under suitable environmental conditions in the laboratory (Supplementary Fig. 1d, e).

**Fig. 1:**
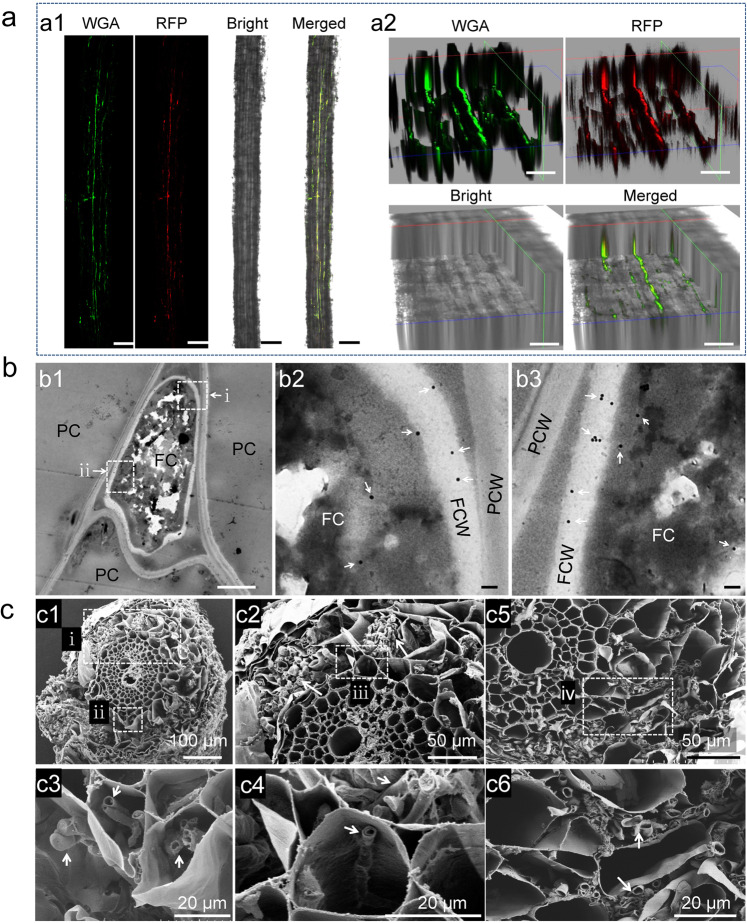
*S. sclerotiorum* growing endophytically in wheat roots. **a** Visualized by confocal microscopy, hyphae of strain DT-8VF^RFP^ in a segment of a rootlet stained with wheat germ agglutinin (WGA). a2 *Z*-stack images to further show the hyphae inside the rootlet of a1; Scale bars for a1 and a2 are 200 and 50 µm. **b** Visualized by colloidal gold immunoelectron microscopy, hyphae of strain DT-8VF^RFP^ in the intercellular spaces of roots probed with the anti-mCherry antibody (b1), (b2), and (b3) are enlargements of the boxed regions (i and ii) in b1. **c** Visualized by scanning electron microscopy, hyphae of strains DT-8VF and DT-8 in the intercellular and intracellular spaces of roots (indicated by arrows). c1 Transverse section of a rootlet of DT-8VF-treated plant; c2–c4 enlargement of the regions in dotted boxes (i–iii) in c1; c2, c4 a single hypha in a root cell; c5 transverse section of a rootlet of strain DT-8-treated plant, showing hyphae in the intercellular and intracellular spaces of roots; and c6 enlargement of the regions in a dotted box (iv) of c5. No hyphae were observed in root of noninoculated wheat (CK), its SEM pictures were presented in Supplementary Fig. 3. Seedlings germinated from surface-sterilized seeds were inoculated with strains DT-8VF and DT-8 and allowed to grow for an additional 15 days on MS medium in a sterile culture bottle before sampling.

To investigate the growth of *S. sclerotiorum* in wheat, we labeled strain DT-8VF with the mCherry fluorescence protein and inoculated this strain onto wheat seedlings. We found that mCherry-labeled hyphae could grow inside the root epidermal cells of wheat (Fig. 1a). The growth of *S. sclerotiorum* in wheat roots was confirmed by TEM using an immunogold labeling technique with an antibody against the mCherry protein, the latter observed to be randomly distributed in the fungal cells (Fig. 1b1–b3). Under TEM, *S. sclerotiorum* was observed to grow both inter- and intracellularly and hyphae entered neighboring cells by breaking the plant cell walls (Supplementary Fig. 2a, b). When in contact with *S. sclerotiorum* hyphae, the plant cell wall thickened significantly (Supplementary Fig. 2c). Furthermore, when *S. sclerotiorum* was growing intracellularly, the plant generally deposited substances around the fungal cell wall and membrane-like structures accumulated around the fungal cell walls (Supplementary Fig. 2d). Using SEM, a mycelial film was observed around the root surface and masses of hyphae could clearly be seen in root cells (Fig. 1c1), hyphae could be observed both in root cells or intercellular space, even filled cells (Fig. 1c2–c4). No hyphae could be observed in the root cells or in the intercellular space of noninoculated wheat under SEM (Supplementary Fig. 3). These observations supported that *S. sclerotiorum* could grow as an endophytic fungus in wheat roots.

We next tested whether mycovirus-mediated hypovirulent strains of *S. sclerotiorum* could grow in wheat roots. Using SEM and TEM, we found that the mycovirus-infected hypovirulent strain, DT-8, could infect wheat roots just as well as DT-8VF and DT-8VF^RFP^ (Fig. 1c5, c6, Supplementary Fig. 2e, f). Furthermore, other strains, such as virulent strain Ep-1PNA367, and mycovirus-mediated hypovirulent strains, AH98, SCH941, and T1-1-20, could also infect wheat roots (Supplementary Fig. 4a–c). Thus, the endophytic growth of *S. sclerotiorum* on wheat roots was not strain dependent and did not depend on full virulence on susceptible hosts.

To explore whether *S. sclerotiorum* could grow systemically throughout wheat plants, strain DT-8VF^RFP^-inoculated seedlings were grown in soil for 45 days and then observed using confocal microscopy. Red fluorescence was observed in the stalks of the wheat seedlings, whereas no signal was detected in the stalks of noninoculated seedlings (Supplementary Fig. 5a). In addition, we successfully isolated four *S. sclerotiorum* cultures that were identified using PCR amplification and colony morphology observation from eleven individual DT-8VF^RFP^*-*treated wheat plants growing in soil in the greenhouse for 45 days (Supplementary Fig. 5b, c).

In order to determine whether the endophytic growth of *S. sclerotiorum* from wheat roots to aerial parts could occur in natural wheat field located at EZhou City in Hubei province, the roots, flag leaves, and spikes of DT-8-treated and nontreated wheat growing in fields were sampled for *S. sclerotiorum* and mycovirus assays. The results showed that *S. sclerotiorum* and mycoviral DNA could be successfully detected with high frequency. The detection rates of *S. sclerotiorum* for roots, flag leaves, and spikelet were 30/30, 27/30, and 30/30, respectively; and the rates for mycoviral DNA for roots, flag leaves, and spikelet were 27/30, 20/30, and 25/30, respectively (Supplementary Fig. 6a–c). When *S. sclerotiorum* was assayed from the roots, flag leaves, and spikes of nontreated wheat, the detection rates of *S. sclerotiorum* for roots, flag leaves, and spikes were 6/20, 2/20, and 8/20, respectively, mycoviral DNA was not detected in noninoculated wheat plant, suggesting a low-to-moderate level of colonization by environmental *S. sclerotiorum*.

Furthermore, we also could identify transcripts from 97 *S. sclerotiorum* genes in wheat spike RNA samples of plants from DT-8-inoculated seeds by RNA-seq analysis. In contrast, no *S. sclerotiorum* transcripts were identified from spike RNA samples of nontreated plants. Transcripts from a few *S. sclerotiorum* genes were identified from flag leaf RNA samples, both from DT-8-treated plants (19 genes) and nontreated plants (16 genes) (Supplementary Table 1), again suggesting some colonization by environmental *S. sclerotiorum*. Overall, these results suggest that *S. sclerotiorum*, in the field, can not only colonize the roots of wheat, but also can move to the aerial parts from the roots, reaching spikes.

### *S. sclerotiorum* promotes wheat growth in the greenhouse and in the field

**Fig. 2:**
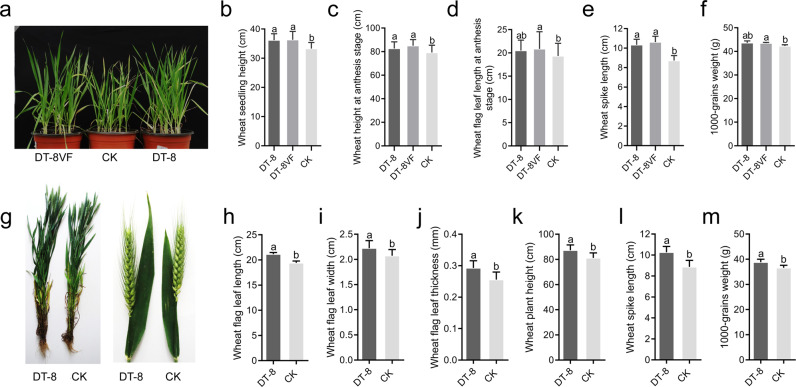
*S. sclerotiorum* promotes wheat growth. **a** Representative image of wheat plants treated with strains DT-8VF and DT-8; seedlings were grown in a greenhouse for 50 days. **b** Wheat seedling height 50 days after planting (*t*-test, *p* < 0.01) (*n* = 28). **c** Wheat plant height at the anthesis stage (*t*-test, *p* < 0.01) (*n* = 60). **d** Flag leaf length at the anthesis stage (*t*-test, *p* > 0.05) (*n* = 60). **e** Wheat spike length at the anthesis stage (*t*-test, *p* < 0.01) (*n* = 60). **f** The 1000-grain weight (*t*-test, *p* > 0.05) (*n* = 4). **g** Representative image of DT-8-treated and nontreated wheat plant at the anthesis stage in field. The length (**h**), width (**i**), and thickness (**j**) of flag leaves, the height of plant (**k**), and length of spike (**l**) of wheat plant at the anthesis stage in field (*t*-test, *p* < 0.01) (*n* = 120). **m** The weight of 1000-grains of DT-8-treated and nontreated wheat plant in field (*t*-test, *p* < 0.05) (*n* = 5). In **b**–**f** and **h**–**m**, error bars indicate standard deviation and different letters indicate significant differences.

To understand the possible effects of *S. sclerotiorum* on wheat growth, we sowed seeds treated with either strain DT-8VF or strain DT-8 in sterilized soil in pots. Seedlings were allowed to grow in the greenhouse until harvest. We found that *S. sclerotiorum*-treated plants grew more robustly (Fig. 2a). At the seedling stage, the average height of seedlings treated with strains DT-8 and DT-8VF was 36.2 ± 2.2 and 36.3 ± 2.9 cm, respectively, significantly greater than those of the nontreated plants (33.3 ± 2.1 cm) (*p* < 0.01) (Fig. 2b).

At the anthesis stage, the average plant heights of those treated with strains DT-8 and DT-8VF were 82.7 ± 5.6 and 85.1 ± 5.2 cm, respectively, significantly greater than those for the nontreated plants (79.3 ± 6.1 cm) (*p* < 0.01) (Fig. 2c). The lengths of flag leaves of plants treated with strains DT-8 and DT-8VF were 20.5 ± 2.2 and 20.9 ± 3.7 cm, while the lengths from the nontreated plants were 19.5 ± 2.7 (Fig. 2d). At the maturation stage, the spike lengths of plants treated with strains DT-8 and DT-8VF were 10.3 ± 0.6 and 10.6 ± 0.6 cm, respectively, significantly longer than those of nontreated plants (8.73 ± 0.51 cm) (*p* < 0.01) (Fig. 2e). The 1000-grain weights from plants treated with strains DT-8 and DT-8VF were 43.6 ± 0.9 and 43.5 ± 0.3 g; however, there was no significant difference between DT-8-treated and nontreated plants (42.3 ± 0.5) (Fig. 2f). However, overall, in the greenhouse, both DT-8VF and DT-8-treated wheat plants grew larger than nontreated plants.

In experimental wheat fields, at the filling stage, the length, width, and thickness of flag leaves, the height of plant and length of spike, and the weight of 1000-grains (harvested) of DT-8-treated and nontreated wheat were determined. All these observed data showed that the DT-8-treated wheat grew significantly better than nontreated wheat (Fig. 2g–m). In particular, the 1000-grain weights were significantly greater (4.3% increase; *p* < 0.05; Fig. 2m).

Other tested strains of *S. sclerotiorum*, whether virulent strains or mycovirus-mediated disabled strains, also could promote wheat growth, as showed at the seedling stage (Supplementary Fig. 4d, e). Therefore, we concluded that for *S. sclerotiorum*, both virulent strains and disabled strains, had the potential to promote wheat growth and enhance wheat yield.

### *S. sclerotiorum* increases wheat yield under natural farming conditions

To examine the impact of *S. sclerotiorum* colonization on wheat growth and grain yield under natural farming conditions, we conducted three seasons of field tests at different farming locations. In 2016, wheat seeds were treated with hypovirulent strain DT-8 and then sown in a natural field located at EZhou City. We found the yield per 5 m^2^ of treated wheat increased by 5.4% (*p* < 0.05) compared to the nontreated wheat in the same location (Table 1). In late autumn of 2017, we again planted strain DT-8-treated wheat at EZhou City, as well as at two additional fields located at Jingmen City and Xiangyang City in Hubei province. For wheat harvested in late May of 2018, seed yields per 5 m^2^ of DT-8-treated wheat at the three locations increased by 17.2%, 10.6%, and 4.3% (*p* < 0.05), respectively, compared with the nontreated controls (Table 1). To examine whether *S. sclerotiorum* could promote seed yield in spring wheat that was planted in the northwest of China, we repeated the field experiment in early summer of 2017 at both Minqin County and Tianzhu County in Gansu province with cultivar Yongliang 4. When harvested in mid-July, seed yields per 5 m^2^ of DT-8-treated wheat increased significantly by 6.2% and 9.0% (*p* < 0.05), respectively, compared with the control (Table 1). In late autumn of 2018, we again planted strain DT-8-treated wheat at EZhou City and Jingmen City. Wheat was harvested in late May of 2019, and seed yield per 5 m^2^ of DT-8-treated wheat at the two locations increased by 10.7% and 11.6% (*p* < 0.05), respectively, compared with the control (Table 1). These data confirmed that *S. sclerotiorum* could significantly improve wheat yields under natural farming conditions in multiple locations.

**Table 1:** *S. sclerotiorum* strain DT-8 significantly promotes the wheat seed yield in the natural fields (*t*-test, *p* < 0.05; mean ± SD) (Tianzhu County and Jingmen City in 2017, *n* = 4; other places, *n* = 5).

Years	Wheat type/cultivar	Location	Yield		Yield increased (%)
			DT-8 treated (g)	Control (g)	
2016–2017	Winter, Zheng 9023	EZhou, Hubei	1903 ± 165	1808 ± 112	5.4
2017–2018	Winter, Zheng 9023	EZhou, Hubei	2098 ± 211	1790 ± 70	17.2
2017–2018	Winter, Zheng 9023	Jingmen, Hubei	2090 ± 40	1890 ± 124	10.6
2017–2018	Wintert, Zheng 9023	Xiangyang City, Hubei	1975 ± 76	1895 ± 67	4.3
2017	Spring, Yongliang 4	Minqin County, Gansu	4546 ± 165	4309 ± 94	6.2
2017	Spring, Yongliang 4	Tianzhu County, Gansu	5132 ± 212	4709 ± 85	9.0
2018–2019	Winter, Zheng 9023	EZhou, Hubei	2440 ± 201	2202 ± 102	10.7
2018–2019	Winter, Zheng 9023	Jingmen City, Hubei	2259 ± 252	2023 ± 97	11.6

### *S. sclerotiorum* alleviates the severity of FHB

**Fig. 3:**
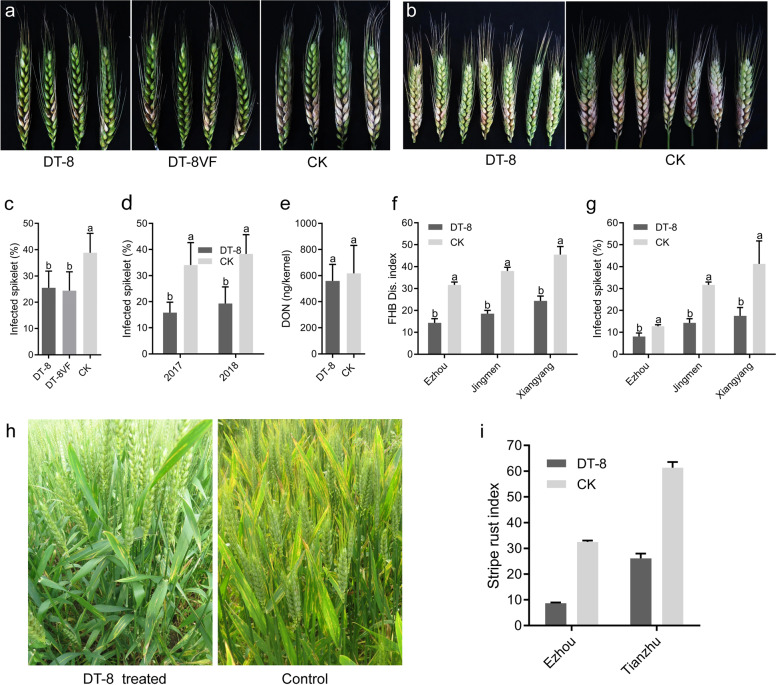
*S. sclerotiorum* enhances wheat resistance against *Fusarium* head blight and stripe rust. **a**, **b** Spikes of wheat plants inoculated with *F. graminearum* in a greenhouse and under field conditions in 2017; photographs taken at 14 days post inoculation (dpi). Wheat seeds of cultivar Zheng 9023 were inoculated with *S. sclerotiorum* strains DT-8 and DT-8VF or water (mock) before sowing. **c** Percent of infected spikelets on *F. graminearum*-inoculated spikes under greenhouse conditions in 2017, 14 dpi (12 spikes for each treatment) (*t*-test, *p* < 0.05) (**c**; *n* = 12). **d** Percent of infected spikelets on *F. graminearum*-inoculated spikes under natural field conditions in 2017 and 2018. Ten plants from each plot were measured from a total of 30 plants in each treated group, (*t*-test between the two groups, *p* < 0.05) (**d**; *n* = 3). **e** DON content in spikelets collected from *F. graminearum* point-inoculated spikelets 14 days after inoculation. Values on black bars followed by the same letter are not significantly different at *p* < 0.05 (**e**; *n* = 6). **f**, **g** Disease index and percent of infected spikelets on spikes from three locations in Hubei province under natural conditions a week before harvest in 2018; 500 spikes were collected from each plot (a total of 1500 spikes from DT-8-treated plots and 1500 spikes from control plots) in each field and then examined (*t*-test between the two groups, *p* < 0.01) (**f**; *n* = 3) and (**g**; *n* = 3). **h**, **i** Strain DT-8-treated wheat revealed strong resistance against stripe rust. **h** Representative images at the anthesis stage of wheat (EZhou Experimental field); **i** disease index of stripe rust naturally occurred in wheat fields located at EZhou, Hubei province (winter wheat) and Tianzhu, Gansu Province (spring wheat) in 2017. In **c**–**g** and **i**, error bars indicate standard deviation and different letters indicate significant differences.

We undertook a pot experiment to investigate whether the endophytic growth of *S. sclerotiorum* could enhance wheat resistance against FHB. At the initial bloom period, both wheat spikes from *S. sclerotiorum*-treated seeds and from nontreated controls were inoculated with a conidial suspension of *F. graminearum* strain PH-1 that is known to cause FHB. At 4 dpi, FHB symptoms appeared first on spikelets around the inoculated site, then the lesions subsequently extended to adjacent spikelets. However, expansion of FHB symptoms on spikelets of the nontreated controls was significantly faster than on the *S. sclerotiorum*-treated plants. At 14 dpi, the percentages of *F. graminearum*-infected spikelets in the virulent DT-8VF-treated plants were 25 ± 6.3% compared to 39 ± 7.3% in the control. The mycovirus-containing hypovirulent strain DT-8 provided the same level of control of disease, namely 24 ± 7.2% *F. graminearum*-infected spikelets (Fig. 3a, c).

This experiment was conducted twice in the field, in 2017 and 2018 at EZhou City. Spikes were inoculated with *F. graminearum* conidia as in the pot experiments. When the plants were treated with the hypovirulent strain, DT-8, the *F. graminearum*-infected spikelets were 16 ± 4.0% in 2017 and 19 ± 6.4% in 2018, significantly less than the percentages for the nontreated controls (34 ± 8.6% in 2017 and 38 ± 7.3% in 2018). The *F. graminearum*-infected spikelets in the DT-8-treated wheat were decreased by 54 ± 2.7 and 50 ± 2.9% in 2017 and 2018, respectively, compared with the control (Fig. 3b, d). To investigate the DON produced in the infected spikelets, DON content in *F. graminearum* point-inoculated spikelets was determined by LC–MS. Results showed that the DON content of DT-8-inoculated spikelets was not significantly different from the infected spikelets in the nontreated control (Fig. 3e). Despite this, since the percentage of infected grains was significantly reduced, strain DT-8 clearly offers significant potential for reducing DON (mycotoxin) accumulation in bulk wheat grain.

To obtain a further field assessment of the ability of strain DT-8 to enhance wheat resistance against FHB, we assessed FHB in the fields in EZhou City, Xiangyang City, and Jingmen City where DT-8-treated wheat and controls were being assessed for growth and yield. In late May of 2018, 1 week before harvest, we carried out the FHB survey. We found that the percentages of *F. graminearum*-infected spikelets in DT-8-treated wheat were 8 ± 0.9%, 18 ± 2.2%, and 14 ± 1.1%, respectively, while in the nontreated fields the percentages were 13 ± 0.4%, 41 ± 6.0%, and 32 ± 0.8%, respectively (Fig. 3f). The disease indices of DT-8-treated wheat were 14 ± 1.4, 25 ± 2.2, and 19 ± 1.5, respectively, whereas in the nontreated fields the disease indices were, 19 ± 1.6, 38 ± 3.7, and 50 ± 1.7, respectively (Fig. 3g). The disease control efficiencies were 40 ± 5.8%, 55 ± 4.1% and 59 ± 3.5% in the DT-8-treated wheat, respectively, across the three locations.

### *S. sclerotiorum* provides protection against wheat stripe rust and rice blast diseases

**Fig. 4:**
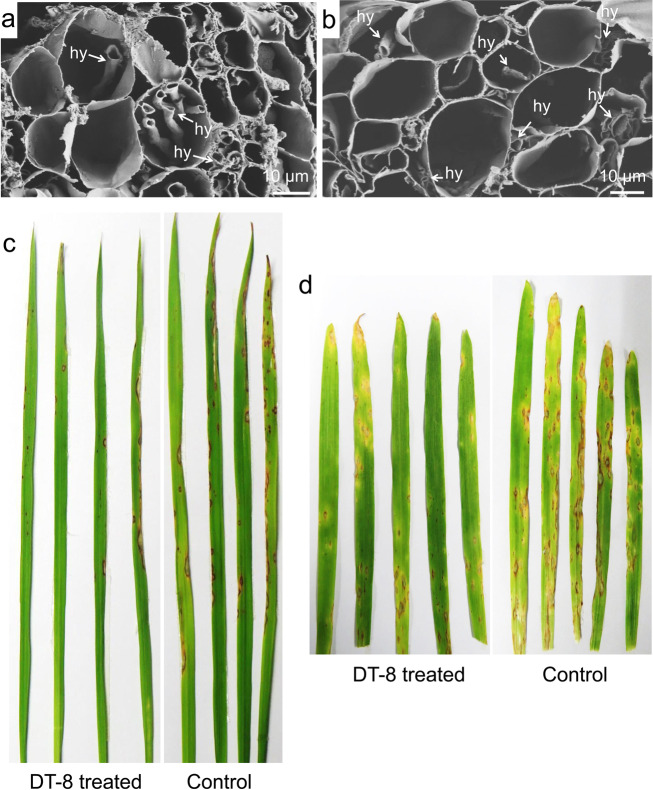
*S*. *sclerotiorum* grows in and enhances the resistance of barley and rice against blast fungi. Hyphae of strain DT-8 in the roots of rice (**a**) and barley (**b**), located in the intercellular and intracellular spaces. hy indicates hyphae, arrows indicate hyphae in root cells. Images were visualized by SEM. **c**, **d** DT-8-treated rice and barley, respectively, showing strong resistance against the rice blast fungus, *M. oryzae*; representative images of barley and rice inoculated with *M. oryzae* in a chamber. Barley and rice seeds were treated with DT-8 or water (mock) before sowing; photographs were taken at 5 dpi for barley and 7 dpi for rice.

The field surveys also revealed that strain DT-8 significantly improved wheat resistance against stripe rust (Fig. 3h, i). To further determine whether *S. sclerotiorum* could provide protection against a wider range of important cereal diseases, we investigated the growth of *S. sclerotiorum* in barley, rice, oat and maize grown on MS medium for 15 days using SEM, and found that both virulent strain DT-8VF and hypovirulent strain DT-8 could grow endophytically, intracellularly and intercellularly, in the roots of these four cereal species (Fig. 4a, b; Supplementary Fig. 7a–d). Interestingly, we found that both *S. sclerotiorum*-treated barley and rice demonstrated strong resistance against the rice blast fungus (Fig. 4c, d), which is a major pathogen of rice and has recently emerged as a new pathogen of wheat [7].

### *S. sclerotiorum* modulates wheat gene expression

To understand the possible mechanisms by which the endophytic growth of *S. sclerotiorum* could promote wheat yield and enhance resistance against FHB, wheat flag leaf and spike samples of the DT-8-treated and control plants were collected from the field located at EZhou City at the initial bloom stage and subjected to RNA-seq analysis.

**Fig. 5:**
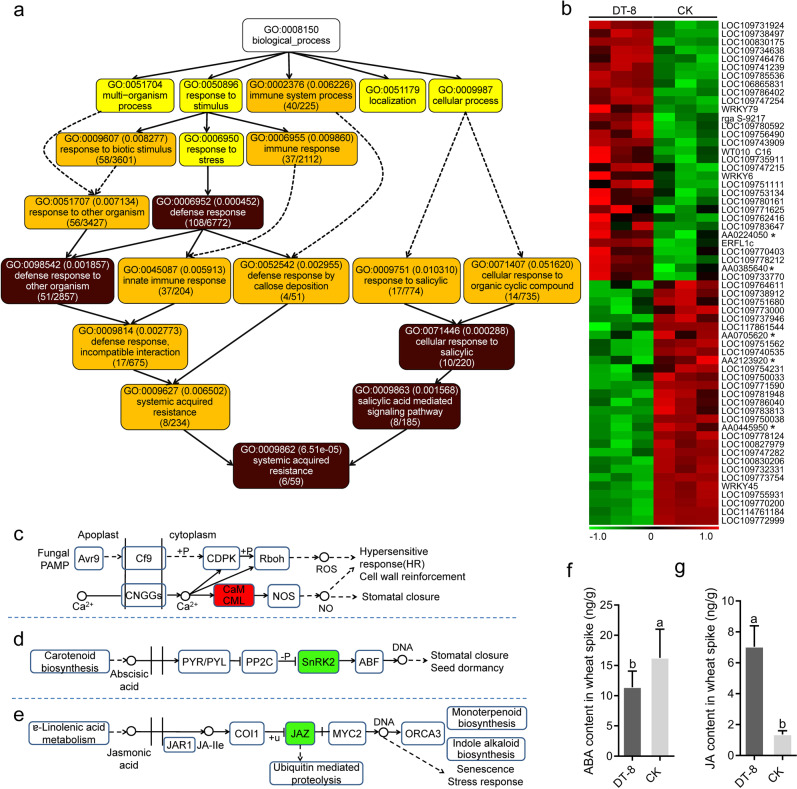
Disease resistance-associated processes in wheat spikes harboring endophytic *S. sclerotiorum*. **a** Schematic diagram of wheat disease resistance processes enriched in the Gene Ontology (GO) analysis. The number in the top bracket in each box is the *p* value, and the lower bracket in the same box represents the number of enriched genes and the total genes in the pathway. The *p* values in the dark-colored boxes were lower than those in light-colored boxes. **b** Heat maps of the transcriptional profiles of 60 wheat DEGs associated with the wheat defense response in wheat spikes from DT-8-treated and control plants grown under natural conditions (|log2FC| > 1, FDR < 0.01). Each gene is represented by a colored bar. Significantly upregulated genes are highlighted in red and downregulated genes in green. All genesʼ gene_id were listed in Supplementary Table 4, genes labeled with “*” do not have common name. **c** Pattern-triggered immunity (PTI) pathway-related genes enriched following the Kyoto Encyclopedia of Genes and Genomes (KEGG) enrichment analysis. **d**, **e** Abscisic acid (ABA) and jasmonic acid (JA) metabolism-related genes enriched following the KEGG analysis. Determination of ABA (**f**; *n* = 6) and JA (**g**; *n* = 6) content in wheat spikes (*t-*test, *p* < 0.05). Error bars indicate standard deviation and different letters indicate significant differences.

GO analysis showed that DEGs were significantly enriched in “defense response” and “systemic acquired resistance” terms in spikes of treated plants compared to nontreated plants (Fig. 5a, Supplementary Fig. 8a). A total of 60 DEGs were enriched in “defense response” pathways in the spikes of DT-8-treated plants, and 31 genes were significantly upregulated (FDR < 0.01 and |log2 ratio| ≥ 1) (Fig. 5b; Supplementary Table 2). We also found that pattern-triggered immunity (PTI) pathway genes related to FHB resistance in wheat were upregulated (Fig. 5c, Supplementary Table 3), while ABA and JA metabolism-related genes (including JAZ, a gene that negatively regulates JA synthesis) were significantly downregulated in the spikes of DT-8-treated plants (Fig. 5d, e). Therefore, we tested the ABA and JA levels directly. We found that ABA levels in DT-8-treated plants were reduced, while JA levels in the spikes were increased, consistent with the gene expression patterns obtained by RNA-seq analysis (Fig. 5f, g). These observations are consistent with increased resistance against FHB since JA has been reported as a positive regulator of resistance against FHB, while ABA has been reported as a negative regulator [34, 35].

Similar regulation of wheat defense pathways was also found in wheat flag leaves. The “defense response” term was significantly enriched in biological process analysis of GO (Supplementary Figs. 8b and 9a). Similarly, 93 DEGs were enriched in the “defense response” term in flag leaves of DT-8-treated plants, with 49 genes showing strong upregulation (Supplementary Fig. 9b, Supplementary Table 4). The PTI and effector-triggered immunity pathway-related disease resistance genes (Supplementary Table 5), as well as salicylic acid metabolism-related genes were significantly upregulated, whereas ABA and JA metabolism-related genes were significantly downregulated (Supplementary Fig. 9c–e). We also measured ABA and JA content and found that JA levels in flag leaves of treated plants compared to nontreated plants were not significantly different, while ABA content was reduced (Supplementary Fig. 9f, g). These results suggest that resistance against foliar diseases caused by biotrophic pathogens may be enhanced in *S. sclerotiorum*-treated wheat, as we observed for wheat stripe rust (Fig. 3h, i).

Regarding the possible mechanism of wheat growth promotion by *S. sclerotiorum*, we observed that “chloroplast” term was significantly enriched in the cellular component analysis of GO, and 101 DEGs were enriched in “chloroplast” pathways in flag leaves of DT-8-treated plants (Supplementary Figs. 8c and 10a, b, Supplementary Table 6). We found that the flag leaf photosynthetic rate and chlorophyll content were elevated consistent with the transcript patterns observed by RNA-seq (Supplementary Fig. 10c, d). Furthermore, we also observed that flag leaf size in DT-8-treated plants, including width, length and thickness, was significantly greater than in nontreated plants (Fig. 2g–i). In wheat spikes, ten DEGs related to “chloroplast inner membrane” and “chloroplast starch grain” were enriched in DT-8-treated plants (Supplementary Figs. 8d and 11a, b, Supplementary Table 7). Furthermore, the IAA content in *S. sclerotiorum*-treated wheat spikes was significantly increased (Supplementary Fig. 11c, d). These results suggested that *S. sclerotiorum* promotes plant growth by enhancing photosynthetic activity and increasing the levels of IAA.

## Discussion

Previously, *Colletotrichum* spp., a group of hemibiotrophic pathogens, have been found to endophytically grow in nonhosts and confer beneficial effects for plants [36]. In the current study, we found that although *S. sclerotiorum* is a widespread, destructive necrotrophic pathogen of dicot plants, and causes huge economic losses every year, it could not only grow as a beneficial endophyte on wheat, rice, barley, corn, and other cereal plants, but that it provided protection against FHB and wheat rust. It not only increased the growth and yield of wheat, but clearly it likely provides protection for other cereal crops against diseases, for example, in protecting rice against blast in the field. As well as direct effects on the physiology of the cereal plants that we observed, it is plausible that *S. sclerotiorum* has direct or indirect effects on the microbiome of the plants, and that those microbiome effects may contribute to the growth and disease resistance benefits we observed. These “opposite lifestyles” of *S. sclerotiorum* and *Colletotrichum* spp. on different hosts has important implications, and there will likely be microbes other than *S. sclerotiorum* with this characteristic in nature. For microbes in which single strains can act as a destructive pathogen on one group of plants, while also able to live mutualistically as an endophyte on another group of plants, we have created the term schizotrophism (split nutritional strategy). We currently do not know the possible mechanisms responsible for the schizotrophism of *S. sclerotiorum* on cereals versus dicots. However, the fact that both the disabled strain and the virulent strain could live on wheat without obvious differences suggests that *S. sclerotiorum* is most likely to employ unknown mechanism(s) to enter into wheat and other cereal crops, or possibly pathogenicity factors, such as oxalic acid, plant cell wall degrading enzymes, or effector-like proteins, that have targets in their dicot hosts that are absent of unrecognizable in wheat and other cereal plants.

Cereal crops provide the vast majority of food for human and animals, yet they are often heavily attacked by fungal pathogens [5, 37–39]. Our results suggest that *S. sclerotiorum* could be developed as an environmentally friendly agent to protect cereal crops against major diseases. That these effects of *S. sclerotiorum* were not strain dependent, but common across virulent and disabled strains, was a notable finding. *S. sclerotiorum* is widespread across many different host crops [14], and grows on wheat and possibly also on other cereal crops naturally as presented in this study and also by Comby et al. [40]. These characteristics, and that it survives in soil for long periods, are both properties that readily facilitate its broad utilization as an environment-friendly and efficient agent to improve production of wheat and other cereal crops and their profitability for farmers worldwide. Furthermore, we found that *S. sclerotiorum*-treated rice and barley demonstrated strong resistance against the rice blast fungus, suggesting that *S. sclerotiorum* would likely also improve resistance against the wheat blast fungus, a newly emerging fungal pathogen that “jumped” from rice to wheat [7].

Our findings open a strategy to screen a much wider range of potentially beneficial microbes including pathogens that might operate as schizotrophic microbes. This could dramatically expand the diversity and potential of beneficial microorganisms for promoting crop health. However, there is a potential risk if virulent strains of *S. sclerotiorum* were directly applied in wheat fields in areas where susceptible host crops are also widely planted. In this study, we reduced, even eliminated, this risk using a mycovirus-mediated disabled strain that could not induce any disease on rapeseed and other susceptible host crops in the field. We demonstrated that mycovirus-mediated disabled strains could endophytically grow on wheat and were equally effective in promoting wheat growth and enhancing wheat resistance against FHB as virulent strains of *S. sclerotiorum*. Furthermore, we found that the endophytic growth of DT-8 and its mycovirus could colonize the aerial parts, and reach flag leaves and spikes when it was inoculated on seeds. This suggests that the mycovirus was quite stable in *S. sclerotiorum* on wheat. Hence, mycovirus-mediated disabled strains rather than virulent strains could to be deployed in the field.

Mycoviruses are very common and widespread in the fungal kingdom [41], and hypovirulence-associated mycoviruses that infect plant fungal pathogens are believed to play a role in counterbalancing plant diseases in nature [42], making them an ideal resource for biological control of plant fungal diseases [43–45]. However, how to release mycoviruses into the field, especially those that have difficulty in overcoming the host vegetative incompatibility, is an urgent technical problem [43, 45, 46]. Our approach not only eliminates the risk of *S. sclerotiorum* attacking nearby susceptible crops, but also likely fosters the spread and accumulation of hypovirulence-associated mycoviruses within *S. sclerotiorum* populations; the latter in turn further alleviating damage in the long-term to host crops from this devastating pathogen of susceptible species. Notably, our study is very likely provides a novel approach for future control of fungal crop diseases.

Schizotrophic microbes when growing on one host plant may produce hyphae, spores, or other dormant bodies in the field that then serve as inocula for disease cycles on the susceptible host crops. In this study, the potential contribution of *S. sclerotiorum* growing on wheat and other cereal crops naturally to the cycles of *Sclerotinia*-associated disease in host crops should be considered. The dormant resting sclerotia of *S. sclerotiorum* function as a soil hub for the life cycle of this fungus since they can germinate both myceliogenically to produce infectious hyphae and carpogenically to produce ascospores [47]. Strategies have been developed to reduce sclerotia in soil to control *Sclerotinia*-associated diseases, for example, in heavily infected fields; farmers are advised to rotate susceptible host crops with wheat or other nonhost crops [16–18]. Although the *S. sclerotiorum* life cycle when growing on wheat is currently unknown, it is possible that *S. sclerotiorum* can produce sclerotia or dormant hyphae on wheat debris or residues on/in the soil since it can saprophytically grow on plant debris [48]. If these structures serve as inocula for *Sclerotinia* disease and contribute to disease severity in host crops, then the current strategies for disease control, such as crop rotation, should be modified to manage this aspect. Furthermore, as schizotrophic microbes may be common in nature [36, 49], more attention to this lifestyle is needed in future. Pathogen life cycles should be reconsidered, not only where there are distinct endophytic, necrotrophic, and saprophytic components of the life cycle, but where there is a role of mycoviruses in making otherwise-highly virulent strains to be nonvirulent but still effective endophytes, as happens with *S. sclerotiorum*.

Our discovery of schizotrophism in *S. sclerotiorum* also raises important ecological implications. First, it should transform our thinking about how invasive pathogens may be spread around the world. Endophytes of some healthy plants may in some cases be destructive pathogens of other plant species, and they may spread to new locations via otherwise healthy plants by human activities. Screening of plants species thought to be nonsusceptible hosts may be required to prevent the invasion of schizotrophic fungi into new locations. Second, schizotrophic microbes may have a potential role in regulating the species compositions of natural ecosystems. Here, we speculate that in some cases, dominant plant species might maintain schizotrophic endophytes as weapons to suppress their competitors. For example, schizotrophic *Sclerotinia* endemic in gramineous plants may help those plants to compete with dicotyledons in mixed communities. Furthermore, when plant species that host schizotrophic endophytes arrive in novel ecosystems, those endophytes could enable their hosts to replace native plant species if they act as destructive pathogens of the native species. Related to the above, our findings may also provide a plausible explanation for the emergence of some new diseases [50, 51].

## Supplementary information

Supplementary Table 1

Supplementary Table 2

Supplementary Table 3

Supplementary Table 4

Supplementary Table 5

Supplementary Table 6

Supplementary Table 7

Supplementary Figures legends

Supplementary Fig. 1

Supplementary Fig. 2

Supplementary Fig. 3

Supplementary Fig. 4

Supplementary Fig. 5

Supplementary Fig. 6

Supplementary Fig. 7

Supplementary Fig. 8

Supplementary Fig. 9

Supplementary Fig. 10

Supplementary Fig. 11
