# Supplementary material for: A cosmopolitan fungal pathogen of dicots adopts an endophytic lifestyle on cereal crops and protects them from major fungal diseases
Source: ISME J. 2020 Aug 19;14(12):3120–35. doi: 10.1038/s41396-020-00744-6 (PMC7784893; doi:10.1038/s41396-020-00744-6)
Supplement: Supplementary file 1 — Supplementary Table 1 [file 41396_2020_744_MOESM1_ESM.docx]

**Supplementary Table 1** *Sclerotinia sclerotiorum* genes that identified from the RNA-Seq data of wheat spikes and wheat flag leaves

| **chr** | **strand** | **start** | **end** | **t_name** | **num_exons** | **length** | **gene_id** | **gene_name** | **DT-8-treated wheat plant spike** | | | **DT-8-treated wheat plant flag leaf** | | | **non-treated wheat plant spike** | | | **non-treated wheat plant flag leaf** | | |
| --- | --- | --- | --- | --- | --- | --- | --- | --- | --- | --- | --- | --- | --- | --- | --- | --- | --- | --- | --- | --- |
|  |  |  |  |  |  |  |  |  | **FPKM.D1-1_sample** | **FPKM.D1-2_sample** | **FPKM.D1-3_sample** | **FPKM.D2-1_sample** | **FPKM.D2-2_sample** | **FPKM.D2-3_sample** | **FPKM.C1-1_sample** | **FPKM.C1-2_sample** | **FPKM.C1-3_sample** | **FPKM.C2-1_sample** | **FPKM.C2-2_sample** | **FPKM.C2-3_sample** |
| CP017827.1 | - | 707317 | 708585 | rna10123 | 4 | 1011 | gene10121 | sscle_14g098970 | 4646.333008 | 0 | 0 | 0 | 0 | 0 | 0 | 0 | 0 | 0 | 0 | 0 |
| CP017827.1 | + | 898884 | 902140 | rna10177 | 4 | 2997 | gene10175 | sscle_14g099510 | 0 | 0 | 0 | 2396.686279 | 0 | 0 | 0 | 0 | 0 | 0 | 0 | 0 |
| CP017827.1 | - | 932190 | 933040 | rna10186 | 2 | 801 | gene10184 | sscle_14g099600 | 0 | 2010.268311 | 0 | 0 | 0 | 0 | 0 | 0 | 0 | 0 | 0 | 0 |
| CP017828.1 | + | 245291 | 246431 | rna10512 | 2 | 1089 | gene10510 | sscle_15g102860 | 449.157013 | 0 | 0 | 0 | 0 | 0 | 0 | 0 | 0 | 0 | 0 | 0 |
| CP017828.1 | + | 495072 | 497225 | rna10579 | 2 | 2094 | gene10577 | sscle_15g103520 | 0 | 0 | 0 | 3407.353027 | 0 | 0 | 0 | 0 | 0 | 0 | 0 | 0 |
| CP017828.1 | - | 539507 | 539978 | rna10585 | 3 | 312 | gene10583 | sscle_15g103580 | 0 | 17143.5 | 0 | 0 | 0 | 0 | 0 | 0 | 0 | 0 | 0 | 0 |
| CP017828.1 | - | 1037445 | 1038029 | rna10742 | 3 | 249 | gene10740 | sscle_15g105130 | 3733.988525 | 0 | 0 | 0 | 0 | 0 | 0 | 0 | 0 | 18437.77148 | 0 | 0 |
| CP017828.1 | - | 1416854 | 1417633 | rna10865 | 3 | 486 | gene10863 | sscle_15g106330 | 8605.689453 | 0 | 0 | 0 | 0 | 0 | 0 | 0 | 0 | 0 | 0 | 0 |
| CP017829.1 | - | 36163 | 38945 | rna10977 | 7 | 2319 | gene10975 | sscle_16g107440 | 0 | 0 | 0 | 0 | 2635.791992 | 0 | 0 | 0 | 0 | 0 | 0 | 0 |
| CP017829.1 | + | 305126 | 306268 | rna11052 | 1 | 1143 | gene11050 | sscle_16g108170 | 0 | 0 | 0 | 0 | 570.4199751 | 0 | 0 | 0 | 0 | 0 | 0 | 0 |
| CP017829.1 | - | 672776 | 673861 | rna11163 | 3 | 891 | gene11161 | sscle_16g109260 | 15999.25781 | 2903.679443 | 4459.136719 | 0 | 0 | 0 | 0 | 0 | 0 | 2325.539307 | 0 | 0 |
| CP017814.1 | - | 3732864 | 3733770 | rna1129 | 5 | 576 | gene1129 | sscle_01g010960 | 10730.09375 | 4471.672363 | 0 | 0 | 0 | 6012.016602 | 0 | 0 | 0 | 0 | 0 | 0 |
| CP017829.1 | + | 1128252 | 1129142 | rna11292 | 4 | 540 | gene11290 | sscle_16g110530 | 2743.125 | 0 | 0 | 0 | 0 | 0 | 0 | 0 | 0 | 0 | 0 | 0 |
| CP017829.1 | + | 1168358 | 1171393 | rna11303 | 8 | 1659 | gene11301 | sscle_16g110640 | 1781.782227 | 0 | 1197.436035 | 0 | 0 | 0 | 0 | 0 | 0 | 0 | 0 | 0 |
| CP017814.1 | - | 3745871 | 3746777 | rna1133 | 3 | 603 | gene1133 | sscle_01g011000 | 6595.814453 | 0 | 0 | 0 | 0 | 0 | 0 | 0 | 0 | 0 | 0 | 0 |
| CP017829.1 | - | 1380153 | 1380955 | rna11358 | 2 | 738 | gene11356 | sscle_16g111190 | 0 | 4107.184082 | 0 | 0 | 0 | 0 | 0 | 0 | 0 | 0 | 0 | 0 |
| CP017814.1 | - | 3782899 | 3783993 | rna1147 | 4 | 930 | gene1147 | sscle_01g011120 | 0 | 3464.091309 | 0 | 0 | 0 | 0 | 0 | 0 | 0 | 0 | 0 | 0 |
| CP017814.1 | - | 394433 | 395892 | rna116 | 2 | 1404 | gene116 | sscle_01g001170 | 0 | 2456.959473 | 0 | 0 | 0 | 0 | 0 | 0 | 0 | 0 | 0 | 0 |
| CP017814.1 | - | 3908675 | 3911326 | rna1184 | 1 | 2652 | gene1184 | sscle_01g011480 | 0 | 531.136719 | 0 | 0 | 0 | 0 | 0 | 0 | 0 | 0 | 0 | 0 |
| CP017815.1 | + | 79248 | 80170 | rna1221 | 4 | 621 | gene1221 | sscle_02g011850 | 1268.17749 | 0 | 0 | 0 | 0 | 0 | 0 | 0 | 0 | 0 | 0 | 0 |
| CP017815.1 | - | 1445158 | 1446866 | rna1619 | 3 | 1584 | gene1618 | sscle_02g015760 | 609.000916 | 0 | 0 | 0 | 0 | 0 | 0 | 0 | 0 | 0 | 0 | 0 |
| CP017815.1 | - | 1758832 | 1760315 | rna1707 | 5 | 1197 | gene1706 | sscle_02g016630 | 1237.49939 | 0 | 0 | 0 | 0 | 0 | 0 | 0 | 0 | 1492.280396 | 0 | 0 |
| CP017815.1 | - | 1932113 | 1933956 | rna1766 | 3 | 1554 | gene1765 | sscle_02g017210 | 3173.826904 | 0 | 0 | 0 | 0 | 0 | 0 | 0 | 0 | 0 | 0 | 0 |
| CP017815.1 | - | 3045415 | 3047839 | rna2103 | 7 | 2013 | gene2102 | sscle_02g020510 | 0 | 0 | 0 | 0 | 0 | 1720.276489 | 0 | 0 | 0 | 0 | 0 | 0 |
| CP017815.1 | - | 3103271 | 3104262 | rna2124 | 6 | 666 | gene2123 | sscle_02g020720 | 1486.072144 | 0 | 0 | 0 | 0 | 0 | 0 | 0 | 0 | 0 | 0 | 0 |
| CP017814.1 | - | 705246 | 707044 | rna217 | 5 | 1479 | gene217 | sscle_01g002160 | 2007.555054 | 4073.872803 | 0 | 0 | 0 | 0 | 0 | 0 | 0 | 0 | 0 | 0 |
| CP017815.1 | + | 3532817 | 3533746 | rna2237 | 3 | 789 | gene2236 | sscle_02g021820 | 3717.030762 | 0 | 0 | 0 | 0 | 0 | 0 | 0 | 0 | 0 | 0 | 0 |
| CP017816.1 | - | 339498 | 340139 | rna2375 | 3 | 408 | gene2374 | sscle_03g023190 | 18896.95898 | 3239.363037 | 0 | 0 | 0 | 0 | 0 | 0 | 0 | 0 | 0 | 0 |
| CP017816.1 | - | 395614 | 397022 | rna2396 | 4 | 1179 | gene2395 | sscle_03g023390 | 15859.43164 | 5851.697266 | 6728.524902 | 0 | 0 | 0 | 0 | 0 | 0 | 0 | 0 | 0 |
| CP017816.1 | - | 451286 | 452530 | rna2415 | 3 | 951 | gene2414 | sscle_03g023580 | 6698.768555 | 8110.696777 | 8355.604492 | 0 | 0 | 0 | 0 | 0 | 0 | 0 | 0 | 0 |
| CP017816.1 | + | 603619 | 604502 | rna2459 | 4 | 642 | gene2458 | sscle_03g024020 | 22353.60547 | 23077.76367 | 6384.555176 | 0 | 0 | 0 | 0 | 0 | 0 | 0 | 9975.701172 | 0 |
| CP017816.1 | - | 649931 | 650437 | rna2474 | 3 | 294 | gene2473 | sscle_03g024170 | 79245.32813 | 16270.10254 | 43852.63281 | 0 | 0 | 0 | 0 | 0 | 0 | 6075.70166 | 0 | 0 |
| CP017816.1 | + | 719808 | 721426 | rna2490 | 5 | 942 | gene2489 | sscle_03g024330 | 18367.07617 | 0 | 7324.766113 | 0 | 0 | 0 | 0 | 0 | 0 | 0 | 0 | 0 |
| CP017816.1 | + | 1014848 | 1018128 | rna2571 | 5 | 3072 | gene2570 | sscle_03g025140 | 0 | 373.249695 | 0 | 0 | 0 | 0 | 0 | 0 | 0 | 0 | 0 | 0 |
| CP017816.1 | + | 1229065 | 1230142 | rna2636 | 4 | 765 | gene2635 | sscle_03g025780 | 19993.48438 | 0 | 0 | 0 | 0 | 0 | 0 | 0 | 0 | 0 | 0 | 0 |
| CP017816.1 | + | 1360623 | 1361136 | rna2680 | 3 | 156 | gene2679 | sscle_03g026220 | 20830.75195 | 0 | 0 | 0 | 0 | 0 | 0 | 0 | 0 | 0 | 0 | 0 |
| CP017816.1 | - | 1387522 | 1388378 | rna2688 | 4 | 654 | gene2687 | sscle_03g026300 | 16344.08106 | 10264.57617 | 6824.316406 | 0 | 0 | 0 | 0 | 0 | 0 | 3462.056641 | 0 | 0 |
| CP017816.1 | - | 1420829 | 1422613 | rna2698 | 2 | 1734 | gene2697 | sscle_03g026400 | 0 | 0 | 0 | 0 | 0 | 3994.147461 | 0 | 0 | 0 | 0 | 0 | 0 |
| CP017816.1 | - | 1479304 | 1480057 | rna2717 | 3 | 450 | gene2716 | sscle_03g026580 | 13885.47559 | 1712.012451 | 6621.814941 | 0 | 0 | 0 | 0 | 0 | 0 | 0 | 0 | 0 |
| CP017816.1 | + | 2132384 | 2133795 | rna2924 | 5 | 582 | gene2923 | sscle_03g028580 | 0 | 1133.144897 | 0 | 0 | 0 | 0 | 0 | 0 | 0 | 0 | 0 | 0 |
| CP017816.1 | - | 2269024 | 2270106 | rna2971 | 5 | 771 | gene2970 | sscle_03g029030 | 9422.28418 | 7321.462891 | 0 | 0 | 0 | 0 | 0 | 0 | 0 | 0 | 0 | 0 |
| CP017816.1 | - | 2401230 | 2402458 | rna3006 | 3 | 936 | gene3005 | sscle_03g029380 | 2643.496094 | 0 | 0 | 0 | 0 | 0 | 0 | 0 | 0 | 0 | 0 | 0 |
| CP017816.1 | - | 2421395 | 2423834 | rna3013 | 3 | 1890 | gene3012 | sscle_03g029450 | 783.75 | 0 | 0 | 0 | 0 | 0 | 0 | 0 | 0 | 0 | 0 | 0 |
| CP017816.1 | - | 2538297 | 2538980 | rna3046 | 4 | 453 | gene3045 | sscle_03g029780 | 9483.6875 | 0 | 13068.21777 | 0 | 0 | 0 | 0 | 0 | 0 | 0 | 0 | 0 |
| CP017816.1 | - | 2541709 | 2542339 | rna3048 | 4 | 432 | gene3047 | sscle_03g029800 | 18741.15625 | 13907.42578 | 6790.425781 | 33254.05859 | 0 | 0 | 0 | 0 | 0 | 0 | 0 | 0 |
| CP017816.1 | + | 2606531 | 2607207 | rna3059 | 3 | 450 | gene3058 | sscle_03g029910 | 34611.05078 | 0 | 8787.605469 | 0 | 11998.43164 | 0 | 0 | 0 | 0 | 13374.24902 | 0 | 0 |
| CP017816.1 | + | 2709434 | 2712314 | rna3093 | 4 | 1950 | gene3092 | sscle_03g030250 | 507.550781 | 0 | 0 | 0 | 0 | 0 | 0 | 0 | 0 | 0 | 0 | 0 |
| CP017816.1 | - | 2714204 | 2714983 | rna3095 | 4 | 465 | gene3094 | sscle_03g030270 | 4157.554199 | 0 | 0 | 0 | 0 | 0 | 0 | 0 | 0 | 0 | 0 | 0 |
| CP017816.1 | + | 2759588 | 2761049 | rna3109 | 3 | 750 | gene3108 | sscle_03g030410 | 5278.533203 | 3449.574463 | 0 | 0 | 0 | 0 | 0 | 0 | 0 | 0 | 0 | 0 |
| CP017817.1 | + | 726428 | 728446 | rna3492 | 2 | 1968 | gene3491 | sscle_04g034160 | 0 | 0 | 0 | 0 | 3892.727051 | 0 | 0 | 0 | 0 | 0 | 0 | 0 |
| CP017817.1 | + | 1080532 | 1081106 | rna3591 | 3 | 411 | gene3590 | sscle_04g035090 | 4455.557129 | 0 | 0 | 0 | 0 | 0 | 0 | 0 | 0 | 0 | 0 | 0 |
| CP017817.1 | - | 1205229 | 1208176 | rna3631 | 5 | 2754 | gene3630 | sscle_04g035460 | 0 | 626.281738 | 0 | 0 | 0 | 0 | 0 | 0 | 0 | 0 | 0 | 0 |
| CP017817.1 | + | 2183630 | 2184962 | rna3930 | 2 | 1227 | gene3929 | sscle_04g038370 | 1613.245117 | 0 | 0 | 0 | 0 | 0 | 0 | 0 | 0 | 0 | 0 | 0 |
| CP017817.1 | - | 2682394 | 2683405 | rna4067 | 2 | 939 | gene4066 | sscle_04g039740 | 3675.01709 | 0 | 0 | 0 | 0 | 0 | 0 | 0 | 0 | 0 | 0 | 0 |
| CP017818.1 | - | 106469 | 106822 | rna4157 | 2 | 267 | gene4156 | sscle_05g040640 | 3348.50708 | 9646.75293 | 0 | 0 | 0 | 0 | 0 | 0 | 0 | 0 | 0 | 0 |
| CP017818.1 | + | 373364 | 374235 | rna4251 | 5 | 510 | gene4250 | sscle_05g041520 | 5608.441895 | 0 | 0 | 0 | 0 | 0 | 0 | 0 | 0 | 0 | 0 | 0 |
| CP017818.1 | + | 1988353 | 1989476 | rna4739 | 4 | 525 | gene4738 | sscle_05g046330 | 30284.76563 | 0 | 5600.161133 | 0 | 0 | 0 | 0 | 0 | 0 | 0 | 0 | 0 |
| CP017818.1 | - | 2196108 | 2197102 | rna4797 | 5 | 420 | gene4796 | sscle_05g046900 | 3267.663086 | 9431.573242 | 19807.27148 | 0 | 0 | 0 | 0 | 0 | 0 | 0 | 0 | 12220.01758 |
| CP017818.1 | + | 2470603 | 2480301 | rna4874 | 2 | 9648 | gene4873 | sscle_05g047660 | 102.584999 | 0 | 0 | 0 | 0 | 0 | 0 | 0 | 0 | 0 | 0 | 0 |
| CP017819.1 | + | 19114 | 19758 | rna4971 | 2 | 453 | gene4970 | sscle_06g048630 | 19444.92969 | 12869.27539 | 14997.75391 | 0 | 0 | 0 | 0 | 0 | 0 | 0 | 0 | 0 |
| CP017819.1 | + | 406622 | 409517 | rna5064 | 5 | 2166 | gene5063 | sscle_06g049530 | 0 | 0 | 0 | 0 | 1410.988037 | 0 | 0 | 0 | 0 | 0 | 0 | 0 |
| CP017819.1 | - | 819022 | 820032 | rna5183 | 5 | 609 | gene5182 | sscle_06g050700 | 12129.14258 | 0 | 9187.907227 | 0 | 0 | 0 | 0 | 0 | 0 | 0 | 0 | 0 |
| CP017819.1 | - | 1148877 | 1149741 | rna5283 | 2 | 792 | gene5282 | sscle_06g051690 | 15974.72852 | 11752.65527 | 3762.395996 | 0 | 0 | 0 | 0 | 0 | 0 | 3345.474121 | 0 | 0 |
| CP017819.1 | + | 1189650 | 1191046 | rna5295 | 4 | 1107 | gene5294 | sscle_06g051810 | 2682.181152 | 0 | 0 | 0 | 0 | 0 | 0 | 0 | 0 | 0 | 0 | 0 |
| CP017819.1 | - | 1383239 | 1384600 | rna5355 | 1 | 1362 | gene5354 | sscle_06g052390 | 0 | 0 | 1458.547363 | 0 | 0 | 0 | 0 | 0 | 0 | 0 | 0 | 0 |
| CP017819.1 | - | 1456558 | 1456983 | rna5374 | 4 | 198 | gene5373 | sscle_06g052580 | 0 | 30024.07227 | 0 | 0 | 0 | 0 | 0 | 0 | 0 | 0 | 0 | 0 |
| CP017814.1 | + | 1852225 | 1852958 | rna554 | 5 | 399 | gene554 | sscle_01g005340 | 7242.560059 | 0 | 0 | 0 | 0 | 0 | 0 | 0 | 0 | 0 | 0 | 0 |
| CP017819.1 | - | 2110022 | 2111120 | rna5542 | 5 | 564 | gene5541 | sscle_06g054240 | 2389.206055 | 0 | 0 | 0 | 0 | 0 | 0 | 0 | 0 | 0 | 0 | 0 |
| CP017819.1 | + | 2111392 | 2112140 | rna5543 | 4 | 471 | gene5542 | sscle_06g054250 | 10450.59863 | 0 | 0 | 0 | 0 | 0 | 0 | 0 | 0 | 0 | 0 | 0 |
| CP017819.1 | + | 2180021 | 2180811 | rna5562 | 5 | 498 | gene5561 | sscle_06g054430 | 0 | 5978.779785 | 0 | 0 | 0 | 0 | 0 | 0 | 0 | 0 | 8379.165039 | 0 |
| CP017820.1 | - | 691128 | 692067 | rna5853 | 6 | 477 | gene5852 | sscle_07g057240 | 7262.133301 | 0 | 0 | 0 | 0 | 0 | 0 | 0 | 0 | 0 | 0 | 0 |
| CP017820.1 | - | 1099689 | 1100300 | rna5971 | 3 | 345 | gene5970 | sscle_07g058340 | 14343.84082 | 0 | 0 | 0 | 0 | 0 | 0 | 0 | 0 | 0 | 0 | 0 |
| CP017820.1 | + | 1845428 | 1848945 | rna6180 | 4 | 3321 | gene6179 | sscle_07g060430 | 0 | 1038.712891 | 0 | 0 | 0 | 0 | 0 | 0 | 0 | 0 | 0 | 0 |
| CP017820.1 | + | 2391261 | 2395947 | rna6348 | 1 | 4687 | gene6354 | sscle_012335 | 3791.956543 | 3474.446045 | 38230.94922 | 5832.255859 | 5996.291504 | 2442.5271 | 0 | 0 | 0 | 3502.718018 | 0 | 0 |
| CP017821.1 | + | 87462 | 89454 | rna6379 | 8 | 777 | gene6378 | sscle_08g062310 | 2534.817627 | 0 | 0 | 0 | 0 | 0 | 0 | 0 | 0 | 0 | 0 | 0 |
| CP017821.1 | + | 1020705 | 1021549 | rna6659 | 2 | 795 | gene6658 | sscle_08g065070 | 622.467712 | 0 | 0 | 0 | 0 | 0 | 0 | 0 | 0 | 0 | 0 | 0 |
| CP017821.1 | + | 1092277 | 1093680 | rna6680 | 2 | 1350 | gene6679 | sscle_08g065270 | 0 | 0 | 1471.512939 | 0 | 0 | 0 | 0 | 0 | 0 | 0 | 0 | 0 |
| CP017821.1 | + | 1201810 | 1202304 | rna6708 | 2 | 438 | gene6707 | sscle_08g065540 | 4383.712891 | 0 | 2267.746338 | 0 | 0 | 0 | 0 | 0 | 0 | 0 | 0 | 0 |
| CP017821.1 | + | 1777583 | 1778121 | rna6875 | 3 | 336 | gene6874 | sscle_08g067190 | 0 | 7665.719238 | 0 | 0 | 0 | 0 | 0 | 0 | 0 | 0 | 0 | 0 |
| CP017821.1 | + | 1778772 | 1779831 | rna6876 | 4 | 666 | gene6875 | sscle_08g067200 | 11824.19141 | 6362.205078 | 0 | 7221.651367 | 0 | 0 | 0 | 0 | 0 | 8046.19873 | 0 | 0 |
| CP017821.1 | - | 1825122 | 1827013 | rna6889 | 5 | 1383 | gene6888 | sscle_08g067290 | 32168.4707 | 29615.29102 | 9998.443359 | 6457.489746 | 0 | 0 | 0 | 0 | 0 | 0 | 0 | 0 |
| CP017814.1 | - | 2380331 | 2381307 | rna715 | 7 | 570 | gene715 | sscle_01g006920 | 4184.626465 | 0 | 3473.545654 | 0 | 0 | 0 | 0 | 0 | 0 | 0 | 0 | 0 |
| CP017822.1 | + | 492058 | 492635 | rna7171 | 2 | 438 | gene7170 | sscle_09g070050 | 27891.56641 | 23062.79102 | 15677.67578 | 0 | 0 | 0 | 0 | 0 | 0 | 0 | 0 | 2725.077881 |
| CP017822.1 | - | 542752 | 543821 | rna7182 | 5 | 708 | gene7181 | sscle_09g070160 | 18009.82617 | 26748.82227 | 4208.779297 | 0 | 0 | 0 | 0 | 0 | 0 | 0 | 0 | 0 |
| CP017822.1 | - | 574070 | 574752 | rna7192 | 2 | 615 | gene7191 | sscle_09g070260 | 16044.81055 | 14003.96094 | 11294.76953 | 0 | 0 | 0 | 0 | 0 | 0 | 0 | 0 | 0 |
| CP017822.1 | - | 876770 | 877777 | rna7285 | 6 | 411 | gene7284 | sscle_09g071170 | 15453.43457 | 5752.125488 | 0 | 0 | 0 | 0 | 0 | 0 | 0 | 0 | 0 | 0 |
| CP017822.1 | + | 1492349 | 1493758 | rna7465 | 5 | 1197 | gene7464 | sscle_09g072950 | 0 | 0 | 0 | 6000.730469 | 0 | 0 | 0 | 0 | 0 | 0 | 0 | 0 |
| CP017822.1 | + | 1835150 | 1836327 | rna7565 | 6 | 750 | gene7564 | sscle_09g073930 | 14498.37305 | 20674.44922 | 0 | 0 | 0 | 0 | 0 | 0 | 0 | 0 | 0 | 0 |
| CP017822.1 | - | 2046261 | 2048841 | rna7626 | 3 | 2460 | gene7625 | sscle_09g074540 | 804.653015 | 0 | 0 | 0 | 0 | 0 | 0 | 0 | 0 | 0 | 0 | 0 |
| CP017823.1 | - | 119039 | 119433 | rna7689 | 2 | 264 | gene7688 | sscle_10g075140 | 0 | 9495.040039 | 0 | 0 | 0 | 0 | 0 | 0 | 0 | 0 | 0 | 0 |
| CP017823.1 | - | 1818834 | 1821962 | rna8185 | 4 | 2958 | gene8184 | sscle_10g080020 | 836.482605 | 2324.593506 | 664.869934 | 0 | 0 | 0 | 0 | 0 | 0 | 1497.603394 | 0 | 0 |
| CP017823.1 | + | 1900573 | 1901492 | rna8207 | 4 | 639 | gene8206 | sscle_10g080240 | 1548.866821 | 0 | 0 | 0 | 0 | 0 | 0 | 0 | 0 | 0 | 0 | 0 |
| CP017824.1 | + | 545528 | 548114 | rna8443 | 8 | 1665 | gene8442 | sscle_11g082560 | 1188.857666 | 0 | 0 | 0 | 0 | 0 | 0 | 0 | 0 | 0 | 0 | 0 |
| CP017824.1 | - | 799219 | 800487 | rna8512 | 5 | 1041 | gene8511 | sscle_11g083230 | 1410.272339 | 800.812988 | 1787.446289 | 3426.98584 | 0 | 0 | 0 | 0 | 0 | 0 | 0 | 0 |
| CP017824.1 | + | 811597 | 813707 | rna8516 | 8 | 1530 | gene8515 | sscle_11g083270 | 2904.486572 | 0 | 0 | 0 | 0 | 0 | 0 | 0 | 0 | 0 | 0 | 0 |
| CP017824.1 | + | 1027090 | 1029859 | rna8587 | 3 | 2673 | gene8586 | sscle_11g083980 | 356.132751 | 0 | 0 | 0 | 0 | 0 | 0 | 0 | 0 | 0 | 0 | 0 |
| CP017825.1 | - | 177209 | 177881 | rna8920 | 4 | 408 | gene8918 | sscle_12g087220 | 14344.54395 | 23250.80469 | 0 | 0 | 0 | 0 | 0 | 0 | 0 | 0 | 0 | 0 |
| CP017825.1 | + | 496517 | 497223 | rna9006 | 3 | 450 | gene9004 | sscle_12g088050 | 9838.599609 | 0 | 0 | 0 | 0 | 0 | 0 | 0 | 0 | 7489.047363 | 0 | 0 |
| CP017825.1 | - | 779726 | 780412 | rna9096 | 4 | 360 | gene9094 | sscle_12g088920 | 3802.540039 | 0 | 4648.924805 | 0 | 0 | 0 | 0 | 0 | 0 | 0 | 0 | 0 |
| CP017825.1 | + | 780462 | 781323 | rna9097 | 4 | 645 | gene9095 | sscle_12g088930 | 36245.06641 | 14636.18164 | 4619.867676 | 13177.88477 | 4738.297363 | 0 | 0 | 0 | 0 | 0 | 0 | 0 |
| CP017814.1 | - | 3107204 | 3108594 | rna938 | 4 | 1122 | gene938 | sscle_01g009130 | 2171.677734 | 0 | 0 | 0 | 0 | 0 | 0 | 0 | 0 | 0 | 0 | 0 |
| CP017826.1 | + | 1180523 | 1181068 | rna9740 | 1 | 546 | gene9738 | sscle_13g095240 | 1812.684692 | 0 | 0 | 0 | 0 | 0 | 0 | 0 | 0 | 0 | 0 | 0 |
| CP017826.1 | + | 1366026 | 1366697 | rna9794 | 4 | 384 | gene9792 | sscle_13g095780 | 19476.62109 | 32594.28711 | 4586.984375 | 8074.498047 | 0 | 0 | 0 | 0 | 0 | 0 | 0 | 0 |
| CP017814.1 | + | 3266898 | 3269686 | rna983 | 3 | 2535 | gene983 | sscle_01g009540 | 8358.979492 | 4061.925537 | 0 | 3206.555908 | 0 | 0 | 0 | 0 | 0 | 1409.272949 | 0 | 0 |
| CP017827.1 | - | 93447 | 95274 | rna9939 | 2 | 1758 | gene9937 | sscle_14g097230 | 559.230957 | 0 | 0 | 0 | 0 | 0 | 0 | 0 | 0 | 0 | 0 | 0 |
| CP017827.1 | - | 282296 | 282869 | rna9999 | 3 | 279 | gene9997 | sscle_14g097800 | 4966.36084 | 44226.94141 | 27998.42969 | 0 | 0 | 0 | 0 | 0 | 0 | 0 | 0 | 0 |
| Total gene number | | | | | | | | | 97 | | | 19 | | | 0 | | | 16 | | |
