# Supplementary material for: A cosmopolitan fungal pathogen of dicots adopts an endophytic lifestyle on cereal crops and protects them from major fungal diseases
Source: ISME J. 2020 Aug 19;14(12):3120–35. doi: 10.1038/s41396-020-00744-6 (PMC7784893; doi:10.1038/s41396-020-00744-6)
Supplement: Supplementary file 2 — Supplementary Table 2 [file 41396_2020_744_MOESM2_ESM.docx]

**Supplementary Table 2** Wheat DEGs associated with the defense response in DT-8 treated and control wheat spikes

| **gene** | **gene_id** | **DT-8 Sample 1_count** | **DT-8 Sample 2_count** | **DT-8 Sample 3_count** | **Control Sample 1_count** | **Control Sample 2_count** | **Control Sample 3_count** | **logFC** | **FDR** | **exp** | **eggnog** | **Kegg** | **GO** | **uniprot_hit** |
| --- | --- | --- | --- | --- | --- | --- | --- | --- | --- | --- | --- | --- | --- | --- |
| LOC109731924 | TRIAE_CS42_3B_TGACv1_224883_AA0802750 | 1202 | 718 | 645 | 92 | 48 | 71 | 3.577488 | 1.42E-15 | up | ENOG410YZDC^Cysteine proteinase inhibitor | KEGG:osa:4327535 | GO:0005576^cellular_component^extracellular region`GO:0004869^molecular_function^cysteine-type endopeptidase inhibitor activity`GO:0002020^molecular_function^protease binding`GO:0006952^biological_process^defense response`GO:2000117^biological_process^negative regulation of cysteine-type endopeptidase activity | CYT1_ORYSJ^CYT1_ORYSJ^Q:7-351,H:1-113^38.14%ID^E:4e-12^RecName: Full=Cysteine proteinase inhibitor 1;^Eukaryota; Viridiplantae; Streptophyta; Embryophyta; Tracheophyta; Spermatophyta; Magnoliophyta; Liliopsida; Poales; Poaceae; BOP clade; Oryzoideae; Oryzeae; Oryzinae; Oryza; Oryza sativa |
| LOC109738497 | TRIAE_CS42_3DS_TGACv1_271549_AA0902370 | 244 | 661 | 500 | 48 | 34 | 34 | 3.569458 | 4.89E-15 | up | ENOG410YAV4^Auxin-induced protein | KEGG:ath:AT1G75500 | GO:0016021^cellular_component^integral component of membrane`GO:0009705^cellular_component^plant-type vacuole membrane`GO:0005886^cellular_component^plasma membrane`GO:0005774^cellular_component^vacuolar membrane`GO:0022857^molecular_function^transmembrane transporter activity`GO:0009851^biological_process^auxin biosynthetic process`GO:0010315^biological_process^auxin efflux`GO:0009734^biological_process^auxin-activated signaling pathway`GO:0071555^biological_process^cell wall organization`GO:0006952^biological_process^defense response`GO:0009834^biological_process^plant-type secondary cell wall biogenesis`GO:0090355^biological_process^positive regulation of auxin metabolic process`GO:0090358^biological_process^positive regulation of tryptophan metabolic process`GO:0000162^biological_process^tryptophan biosynthetic process`GO:0009826^biological_process^unidimensional cell growth | WAT1_ARATH^WAT1_ARATH^Q:1-807,H:1-272^76.1%ID^E:3e-145^RecName: Full=Protein WALLS ARE THIN 1;^Eukaryota; Viridiplantae; Streptophyta; Embryophyta; Tracheophyta; Spermatophyta; Magnoliophyta; eudicotyledons; Gunneridae; Pentapetalae; rosids; malvids; Brassicales; Brassicaceae; Camelineae; Arabidopsis |
| LOC100830175 | TRIAE_CS42_2BS_TGACv1_146330_AA0462730 | 320 | 325 | 343 | 24 | 32 | 36 | 3.392824 | 1.60E-13 | up | COG4886^leucine Rich Repeat | KEGG:ath:AT3G14460 | GO:0005886^cellular_component^plasma membrane`GO:0043531^molecular_function^ADP binding`GO:0005524^molecular_function^ATP binding`GO:0006952^biological_process^defense response`GO:0007165^biological_process^signal transduction | DRL21_ARATH^DRL21_ARATH^Q:367-1761,H:166-628^35.23%ID^E:3e-75^RecName: Full=Putative disease resistance protein At3g14460;^Eukaryota; Viridiplantae; Streptophyta; Embryophyta; Tracheophyta; Spermatophyta; Magnoliophyta; eudicotyledons; Gunneridae; Pentapetalae; rosids; malvids; Brassicales; Brassicaceae; Camelineae; Arabidopsis |
| LOC109734638 | TRIAE_CS42_7BS_TGACv1_592906_AA1946090 | 184 | 417 | 380 | 55 | 25 | 29 | 3.142546 | 5.68E-12 | up | ENOG410XQ5A^mitogen-activated protein kinase kinase | KEGG:ath:AT3G21220`KO:K13413 | GO:0005737^cellular_component^cytoplasm`GO:0005524^molecular_function^ATP binding`GO:0004674^molecular_function^protein serine/threonine kinase activity`GO:0032147^biological_process^activation of protein kinase activity`GO:0009814^biological_process^defense response, incompatible interaction`GO:0010227^biological_process^floral organ abscission`GO:0010229^biological_process^inflorescence development`GO:0009626^biological_process^plant-type hypersensitive response`GO:0010365^biological_process^positive regulation of ethylene biosynthetic process`GO:0007346^biological_process^regulation of mitotic cell cycle`GO:0023014^biological_process^signal transduction by protein phosphorylation`GO:0031098^biological_process^stress-activated protein kinase signaling cascade | M2K5_ARATH^M2K5_ARATH^Q:211-996,H:67-325^70.61%ID^E:1e-126^RecName: Full=Mitogen-activated protein kinase kinase 5;^Eukaryota; Viridiplantae; Streptophyta; Embryophyta; Tracheophyta; Spermatophyta; Magnoliophyta; eudicotyledons; Gunneridae; Pentapetalae; rosids; malvids; Brassicales; Brassicaceae; Camelineae; Arabidopsis |
| LOC109746476 | TRIAE_CS42_2BL_TGACv1_130448_AA0411640 | 28 | 77 | 74 | 8 | 9 | 4 | 3.041537 | 1.58E-08 | up | . | . | GO:0005737^cellular_component^cytoplasm`GO:0006952^biological_process^defense response`GO:0009607^biological_process^response to biotic stimulus | PR1_ASPOF^PR1_ASPOF^Q:7-477,H:2-157^50.63%ID^E:8e-54^RecName: Full=Pathogenesis-related protein 1;^Eukaryota; Viridiplantae; Streptophyta; Embryophyta; Tracheophyta; Spermatophyta; Magnoliophyta; Liliopsida; Asparagales; Asparagaceae; Asparagoideae; Asparagus |
| LOC109741239 | TRIAE_CS42_2BL_TGACv1_132575_AA0438480 | 1186 | 1781 | 1181 | 231 | 164 | 145 | 2.915806 | 2.97E-11 | up | . | KEGG:nta:107791137 | GO:0016298^molecular_function^lipase activity`GO:0080031^molecular_function^methyl salicylate esterase activity`GO:0008152^biological_process^metabolic process`GO:0009862^biological_process^systemic acquired resistance, salicylic acid mediated signaling pathway | SABP2_TOBAC^SABP2_TOBAC^Q:46-786,H:6-260^43.92%ID^E:6e-67^RecName: Full=Salicylic acid-binding protein 2;^Eukaryota; Viridiplantae; Streptophyta; Embryophyta; Tracheophyta; Spermatophyta; Magnoliophyta; eudicotyledons; Gunneridae; Pentapetalae; asterids; lamiids; Solanales; Solanaceae; Nicotianoideae; Nicotianeae; Nicotiana |
| LOC109785536 | TRIAE_CS42_2AL_TGACv1_094670_AA0301260 | 502 | 360 | 340 | 73 | 47 | 50 | 2.794507 | 5.19E-10 | up | COG1028^Dehydrogenase reductase | KEGG:ath:AT3G61220`KO:K15095 | GO:0005737^cellular_component^cytoplasm`GO:0047501^molecular_function^(+)-neomenthol dehydrogenase activity`GO:0006952^biological_process^defense response | SDR1_ARATH^SDR1_ARATH^Q:25-843,H:7-296^47.6%ID^E:8e-76^RecName: Full=(+)-neomenthol dehydrogenase {ECO:0000303\|PubMed:18599651};^Eukaryota; Viridiplantae; Streptophyta; Embryophyta; Tracheophyta; Spermatophyta; Magnoliophyta; eudicotyledons; Gunneridae; Pentapetalae; rosids; malvids; Brassicales; Brassicaceae; Camelineae; Arabidopsis |
| LOC106865831 | TRIAE_CS42_5DL_TGACv1_433432_AA1413030 | 87 | 70 | 61 | 16 | 6 | 12 | 2.642727 | 2.60E-07 | up | COG4886^leucine Rich Repeat | KEGG:osa:4337016`KO:K13420 | GO:0016021^cellular_component^integral component of membrane`GO:0005886^cellular_component^plasma membrane`GO:0005524^molecular_function^ATP binding`GO:0004674^molecular_function^protein serine/threonine kinase activity`GO:0006952^biological_process^defense response | FLS2_ORYSJ^FLS2_ORYSJ^Q:811-1614,H:89-352^34.94%ID^E:1e-23^RecName: Full=LRR receptor-like serine/threonine-protein kinase FLS2 {ECO:0000305};^Eukaryota; Viridiplantae; Streptophyta; Embryophyta; Tracheophyta; Spermatophyta; Magnoliophyta; Liliopsida; Poales; Poaceae; BOP clade; Oryzoideae; Oryzeae; Oryzinae; Oryza; Oryza sativa |
| LOC109786402 | TRIAE_CS42_4BS_TGACv1_328313_AA1086120 | 184 | 291 | 246 | 35 | 35 | 49 | 2.569758 | 2.22E-08 | up | . | . | GO:0043531^molecular_function^ADP binding`GO:0005524^molecular_function^ATP binding`GO:0006952^biological_process^defense response | RGA3_SOLBU^RGA3_SOLBU^Q:67-3087,H:20-991^26.57%ID^E:9e-68^RecName: Full=Putative disease resistance protein RGA3;^Eukaryota; Viridiplantae; Streptophyta; Embryophyta; Tracheophyta; Spermatophyta; Magnoliophyta; eudicotyledons; Gunneridae; Pentapetalae; asterids; lamiids; Solanales; Solanaceae; Solanoideae; Solaneae; Solanum |
| LOC109747254 | TRIAE_CS42_1AL_TGACv1_001501_AA0031460 | 225 | 337 | 351 | 60 | 53 | 41 | 2.539337 | 2.28E-08 | up | ENOG410YVV0^VQ motif-containing protein | KEGG:ath:AT1G28280 | GO:0005634^cellular_component^nucleus`GO:0006952^biological_process^defense response`GO:0051245^biological_process^negative regulation of cellular defense response`GO:0043433^biological_process^negative regulation of sequence-specific DNA binding transcription factor activity | VQ4_ARATH^VQ4_ARATH^Q:169-690,H:53-238^48.39%ID^E:3e-31^RecName: Full=VQ motif-containing protein 4 {ECO:0000303\|PubMed:22535423};^Eukaryota; Viridiplantae; Streptophyta; Embryophyta; Tracheophyta; Spermatophyta; Magnoliophyta; eudicotyledons; Gunneridae; Pentapetalae; rosids; malvids; Brassicales; Brassicaceae; Camelineae; Arabidopsis |
| WRKY79 | TRIAE_CS42_7BL_TGACv1_577185_AA1868200 | 247 | 87 | 118 | 29 | 35 | 27 | 2.280278 | 1.53E-06 | up | ENOG410YHES^WRKY transcription factor | . | GO:0005634^cellular_component^nucleus`GO:0043565^molecular_function^sequence-specific DNA binding`GO:0003700^molecular_function^transcription factor activity, sequence-specific DNA binding`GO:0006952^biological_process^defense response`GO:0045892^biological_process^negative regulation of transcription, DNA-templated`GO:1905034^biological_process^regulation of antifungal innate immune response`GO:0010200^biological_process^response to chitin`GO:0009620^biological_process^response to fungus`GO:0006351^biological_process^transcription, DNA-templated | WRK28_ORYSJ^WRK28_ORYSJ^Q:7-978,H:61-394^61.42%ID^E:3e-115^RecName: Full=WRKY transcription factor WRKY28 {ECO:0000303\|PubMed:15618416};^Eukaryota; Viridiplantae; Streptophyta; Embryophyta; Tracheophyta; Spermatophyta; Magnoliophyta; Liliopsida; Poales; Poaceae; BOP clade; Oryzoideae; Oryzeae; Oryzinae; Oryza; Oryza sativa |
| rga S-9217 | TRIAE_CS42_2BL_TGACv1_132250_AA0436350 | 33 | 29 | 42 | 5 | 9 | 11 | 2.01308 | 0.000608 | up | . | . | GO:0043531^molecular_function^ADP binding`GO:0005524^molecular_function^ATP binding`GO:0006952^biological_process^defense response | RGA2_SOLBU^RGA2_SOLBU^Q:3619-4095,H:786-951^31.18%ID^E:1e-05^RecName: Full=Disease resistance protein RGA2;^Eukaryota; Viridiplantae; Streptophyta; Embryophyta; Tracheophyta; Spermatophyta; Magnoliophyta; eudicotyledons; Gunneridae; Pentapetalae; asterids; lamiids; Solanales; Solanaceae; Solanoideae; Solaneae; Solanum |
| LOC109780592 | TRIAE_CS42_2BL_TGACv1_130687_AA0416130 | 45 | 66 | 95 | 12 | 15 | 23 | 2.008812 | 0.000109 | up | ENOG41107PJ^Terpene synthase, N-terminal domain | KEGG:zma:541974`KO:K15793 | GO:0005737^cellular_component^cytoplasm`GO:0102145^molecular_function^(3R)-(E)-nerolidol synthase activity`GO:0016787^molecular_function^hydrolase activity`GO:0000287^molecular_function^magnesium ion binding`GO:0010333^molecular_function^terpene synthase activity`GO:0006952^biological_process^defense response`GO:0008152^biological_process^metabolic process | ACSS_MAIZE^ACSS_MAIZE^Q:364-1869,H:69-580^67.25%ID^E:0^RecName: Full=Acyclic sesquiterpene synthase;^Eukaryota; Viridiplantae; Streptophyta; Embryophyta; Tracheophyta; Spermatophyta; Magnoliophyta; Liliopsida; Poales; Poaceae; PACMAD clade; Panicoideae; Andropogonodae; Andropogoneae; Tripsacinae; Zea |
| LOC109756490 | TRIAE_CS42_U_TGACv1_640941_AA2080000 | 51 | 31 | 57 | 10 | 11 | 16 | 1.872831 | 0.000781 | up | COG4886^leucine Rich Repeat | KEGG:ath:AT5G43470 | GO:0005886^cellular_component^plasma membrane`GO:0043531^molecular_function^ADP binding`GO:0005524^molecular_function^ATP binding`GO:0000166^molecular_function^nucleotide binding`GO:0071446^biological_process^cellular response to salicylic acid stimulus`GO:0006952^biological_process^defense response`GO:0051607^biological_process^defense response to virus`GO:0009626^biological_process^plant-type hypersensitive response`GO:0002230^biological_process^positive regulation of defense response to virus by host`GO:0009646^biological_process^response to absence of light`GO:0009637^biological_process^response to blue light`GO:0009416^biological_process^response to light stimulus`GO:0002239^biological_process^response to oomycetes`GO:0051707^biological_process^response to other organism`GO:0009751^biological_process^response to salicylic acid`GO:0009611^biological_process^response to wounding`GO:0007165^biological_process^signal transduction | RPP8_ARATH^RPP8_ARATH^Q:1-1920,H:1-620^30.34%ID^E:1e-59^RecName: Full=Disease resistance protein RPP8;^Eukaryota; Viridiplantae; Streptophyta; Embryophyta; Tracheophyta; Spermatophyta; Magnoliophyta; eudicotyledons; Gunneridae; Pentapetalae; rosids; malvids; Brassicales; Brassicaceae; Camelineae; Arabidopsis |
| LOC109743909 | TRIAE_CS42_2BL_TGACv1_131715_AA0431080 | 79 | 64 | 66 | 21 | 14 | 22 | 1.842671 | 0.000433 | up | COG0515^Serine Threonine protein kinase | KEGG:ath:AT2G17220 | GO:0005634^cellular_component^nucleus`GO:0005886^cellular_component^plasma membrane`GO:0005524^molecular_function^ATP binding`GO:0004675^molecular_function^transmembrane receptor protein serine/threonine kinase activity`GO:0007166^biological_process^cell surface receptor signaling pathway`GO:0006952^biological_process^defense response`GO:0006468^biological_process^protein phosphorylation | PIX13_ARATH^PIX13_ARATH^Q:256-1179,H:62-367^71.43%ID^E:3e-161^RecName: Full=Probable serine/threonine-protein kinase PIX13 {ECO:0000305};^Eukaryota; Viridiplantae; Streptophyta; Embryophyta; Tracheophyta; Spermatophyta; Magnoliophyta; eudicotyledons; Gunneridae; Pentapetalae; rosids; malvids; Brassicales; Brassicaceae; Camelineae; Arabidopsis |
| WT010_C16 | TRIAE_CS42_1BL_TGACv1_034262_AA0144000 | 1380 | 789 | 615 | 237 | 216 | 361 | 1.748189 | 0.000149 | up | ENOG410ZJIW^MAC/Perforin domain | KEGG:ath:AT1G28380 | GO:0008219^biological_process^cell death`GO:0052542^biological_process^defense response by callose deposition`GO:0006955^biological_process^immune response`GO:0009626^biological_process^plant-type hypersensitive response`GO:0010337^biological_process^regulation of salicylic acid metabolic process`GO:0009651^biological_process^response to salt stress | NSL1_ARATH^NSL1_ARATH^Q:229-1869,H:75-612^51.28%ID^E:0^RecName: Full=MACPF domain-containing protein NSL1;^Eukaryota; Viridiplantae; Streptophyta; Embryophyta; Tracheophyta; Spermatophyta; Magnoliophyta; eudicotyledons; Gunneridae; Pentapetalae; rosids; malvids; Brassicales; Brassicaceae; Camelineae; Arabidopsis |
| LOC109735911 | TRIAE_CS42_7AS_TGACv1_571347_AA1847010 | 1349 | 715 | 598 | 292 | 182 | 339 | 1.68665 | 0.000285 | up | COG1902^NADH flavin oxidoreductase, NADH oxidase | KEGG:osa:4340490`KO:K05894 | GO:0005622^cellular_component^intracellular`GO:0016629^molecular_function^12-oxophytodienoate reductase activity`GO:0010181^molecular_function^FMN binding`GO:0006952^biological_process^defense response`GO:0009695^biological_process^jasmonic acid biosynthetic process`GO:0031408^biological_process^oxylipin biosynthetic process | OPR1_ORYSJ^OPR1_ORYSJ^Q:19-1089,H:14-369^87.68%ID^E:0^RecName: Full=12-oxophytodienoate reductase 1;^Eukaryota; Viridiplantae; Streptophyta; Embryophyta; Tracheophyta; Spermatophyta; Magnoliophyta; Liliopsida; Poales; Poaceae; BOP clade; Oryzoideae; Oryzeae; Oryzinae; Oryza; Oryza sativa |
| LOC109747215 | TRIAE_CS42_7BL_TGACv1_577914_AA1886020 | 93 | 164 | 182 | 69 | 35 | 30 | 1.686084 | 0.000649 | up | COG4886^leucine Rich Repeat | KEGG:osa:4337016`KO:K13420 | GO:0016021^cellular_component^integral component of membrane`GO:0005886^cellular_component^plasma membrane`GO:0005524^molecular_function^ATP binding`GO:0004674^molecular_function^protein serine/threonine kinase activity`GO:0006952^biological_process^defense response | FLS2_ORYSJ^FLS2_ORYSJ^Q:193-3633,H:46-1183^72.06%ID^E:0^RecName: Full=LRR receptor-like serine/threonine-protein kinase FLS2 {ECO:0000305};^Eukaryota; Viridiplantae; Streptophyta; Embryophyta; Tracheophyta; Spermatophyta; Magnoliophyta; Liliopsida; Poales; Poaceae; BOP clade; Oryzoideae; Oryzeae; Oryzinae; Oryza; Oryza sativa |
| WRKY6 | TRIAE_CS42_7AL_TGACv1_557324_AA1779770 | 842 | 366 | 358 | 220 | 139 | 156 | 1.579224 | 0.000922 | up | ENOG410YHES^WRKY transcription factor | . | GO:0005634^cellular_component^nucleus`GO:0043565^molecular_function^sequence-specific DNA binding`GO:0003700^molecular_function^transcription factor activity, sequence-specific DNA binding`GO:0006952^biological_process^defense response`GO:0045892^biological_process^negative regulation of transcription, DNA-templated`GO:1905034^biological_process^regulation of antifungal innate immune response`GO:0010200^biological_process^response to chitin`GO:0009620^biological_process^response to fungus`GO:0006351^biological_process^transcription, DNA-templated | WRK28_ORYSJ^WRK28_ORYSJ^Q:7-975,H:61-394^59.05%ID^E:1e-109^RecName: Full=WRKY transcription factor WRKY28 {ECO:0000303\|PubMed:15618416};^Eukaryota; Viridiplantae; Streptophyta; Embryophyta; Tracheophyta; Spermatophyta; Magnoliophyta; Liliopsida; Poales; Poaceae; BOP clade; Oryzoideae; Oryzeae; Oryzinae; Oryza; Oryza sativa |
| LOC109751111 | TRIAE_CS42_3DL_TGACv1_251757_AA0884610 | 690 | 1309 | 1595 | 529 | 336 | 329 | 1.566222 | 0.000905 | up | ENOG410YA4N^glucan endo-1-3-beta-glucosidase | KEGG:ath:AT2G27500 | GO:0046658^cellular_component^anchored component of plasma membrane`GO:0005618^cellular_component^cell wall`GO:0005737^cellular_component^cytoplasm`GO:0005576^cellular_component^extracellular region`GO:0005634^cellular_component^nucleus`GO:0042973^molecular_function^glucan endo-1,3-beta-D-glucosidase activity`GO:0004553^molecular_function^hydrolase activity, hydrolyzing O-glycosyl compounds`GO:0030247^molecular_function^polysaccharide binding`GO:0005975^biological_process^carbohydrate metabolic process`GO:0071555^biological_process^cell wall organization`GO:0006952^biological_process^defense response | E1314_ARATH^E1314_ARATH^Q:97-1050,H:27-346^63.75%ID^E:1e-150^RecName: Full=Glucan endo-1,3-beta-glucosidase 14;^Eukaryota; Viridiplantae; Streptophyta; Embryophyta; Tracheophyta; Spermatophyta; Magnoliophyta; eudicotyledons; Gunneridae; Pentapetalae; rosids; malvids; Brassicales; Brassicaceae; Camelineae; Arabidopsis |
| LOC109753134 | TRIAE_CS42_4AS_TGACv1_308110_AA1025950 | 963 | 451 | 523 | 306 | 139 | 199 | 1.56527 | 0.001002 | up | COG3621^Patatin group | . | GO:0016787^molecular_function^hydrolase activity`GO:0006952^biological_process^defense response`GO:0016042^biological_process^lipid catabolic process | PLP1_ORYSI^PLP1_ORYSI^Q:139-1281,H:14-396^57.96%ID^E:4e-159^RecName: Full=Patatin-like protein 1;^Eukaryota; Viridiplantae; Streptophyta; Embryophyta; Tracheophyta; Spermatophyta; Magnoliophyta; Liliopsida; Poales; Poaceae; BOP clade; Oryzoideae; Oryzeae; Oryzinae; Oryza; Oryza sativa |
| LOC109780161 | TRIAE_CS42_U_TGACv1_642091_AA2111160 | 1379 | 611 | 633 | 320 | 228 | 339 | 1.539173 | 0.001219 | up | ENOG410ZJIW^MAC/Perforin domain | KEGG:ath:AT1G28380 | GO:0008219^biological_process^cell death`GO:0052542^biological_process^defense response by callose deposition`GO:0006955^biological_process^immune response`GO:0009626^biological_process^plant-type hypersensitive response`GO:0010337^biological_process^regulation of salicylic acid metabolic process`GO:0009651^biological_process^response to salt stress | NSL1_ARATH^NSL1_ARATH^Q:223-1854,H:75-612^51.37%ID^E:0^RecName: Full=MACPF domain-containing protein NSL1;^Eukaryota; Viridiplantae; Streptophyta; Embryophyta; Tracheophyta; Spermatophyta; Magnoliophyta; eudicotyledons; Gunneridae; Pentapetalae; rosids; malvids; Brassicales; Brassicaceae; Camelineae; Arabidopsis |
| LOC109771625 | TRIAE_CS42_4BL_TGACv1_320860_AA1050450 | 74 | 119 | 45 | 18 | 25 | 42 | 1.454691 | 0.00745 | up | ENOG410YZZY^Transcription factor | KEGG:ath:AT3G56400 | GO:0005634^cellular_component^nucleus`GO:0043565^molecular_function^sequence-specific DNA binding`GO:0003700^molecular_function^transcription factor activity, sequence-specific DNA binding`GO:0042742^biological_process^defense response to bacterium`GO:0050832^biological_process^defense response to fungus`GO:0009864^biological_process^induced systemic resistance, jasmonic acid mediated signaling pathway`GO:1900056^biological_process^negative regulation of leaf senescence`GO:0045892^biological_process^negative regulation of transcription, DNA-templated`GO:0031347^biological_process^regulation of defense response`GO:0010200^biological_process^response to chitin`GO:0009753^biological_process^response to jasmonic acid`GO:0009751^biological_process^response to salicylic acid`GO:0009862^biological_process^systemic acquired resistance, salicylic acid mediated signaling pathway`GO:0006351^biological_process^transcription, DNA-templated | WRK70_ARATH^WRK70_ARATH^Q:58-537,H:12-186^36%ID^E:4e-22^RecName: Full=Probable WRKY transcription factor 70;^Eukaryota; Viridiplantae; Streptophyta; Embryophyta; Tracheophyta; Spermatophyta; Magnoliophyta; eudicotyledons; Gunneridae; Pentapetalae; rosids; malvids; Brassicales; Brassicaceae; Camelineae; Arabidopsis |
| LOC109762416 | TRIAE_CS42_5DL_TGACv1_436080_AA1457820 | 2539 | 910 | 1054 | 687 | 526 | 459 | 1.403687 | 0.003939 | up | . | . | GO:0043531^molecular_function^ADP binding`GO:0005524^molecular_function^ATP binding`GO:0006952^biological_process^defense response | RGA1_SOLBU^RGA1_SOLBU^Q:838-2628,H:112-720^29.78%ID^E:2e-56^RecName: Full=Putative disease resistance protein RGA1;^Eukaryota; Viridiplantae; Streptophyta; Embryophyta; Tracheophyta; Spermatophyta; Magnoliophyta; eudicotyledons; Gunneridae; Pentapetalae; asterids; lamiids; Solanales; Solanaceae; Solanoideae; Solaneae; Solanum |
| LOC109783647 | TRIAE_CS42_3B_TGACv1_223389_AA0781480 | 313 | 132 | 297 | 86 | 87 | 104 | 1.394935 | 0.005848 | up | ENOG410ZEWF^late embryogenesis abundant protein | KEGG:ath:AT4G02380 | GO:0005739^cellular_component^mitochondrion`GO:0042631^biological_process^cellular response to water deprivation`GO:0006952^biological_process^defense response`GO:0010150^biological_process^leaf senescence`GO:1900424^biological_process^regulation of defense response to bacterium`GO:1900150^biological_process^regulation of defense response to fungus`GO:1900055^biological_process^regulation of leaf senescence`GO:2000028^biological_process^regulation of photoperiodism, flowering`GO:0009737^biological_process^response to abscisic acid`GO:0009646^biological_process^response to absence of light`GO:0009409^biological_process^response to cold`GO:0009723^biological_process^response to ethylene`GO:0009620^biological_process^response to fungus`GO:0042542^biological_process^response to hydrogen peroxide`GO:0009625^biological_process^response to insect`GO:0009416^biological_process^response to light stimulus`GO:0006979^biological_process^response to oxidative stress`GO:0000302^biological_process^response to reactive oxygen species`GO:1902074^biological_process^response to salt`GO:0009414^biological_process^response to water deprivation`GO:0009611^biological_process^response to wounding`GO:0048364^biological_process^root development | SAG21_ARATH^SAG21_ARATH^Q:16-270,H:16-93^40%ID^E:2e-09^RecName: Full=Protein SENESCENCE-ASSOCIATED GENE 21, mitochondrial {ECO:0000303\|PubMed:9617813};^Eukaryota; Viridiplantae; Streptophyta; Embryophyta; Tracheophyta; Spermatophyta; Magnoliophyta; eudicotyledons; Gunneridae; Pentapetalae; rosids; malvids; Brassicales; Brassicaceae; Camelineae; Arabidopsis |
| AA0224050 | TRIAE_CS42_1DL_TGACv1_063112_AA0224050 | 1134 | 614 | 651 | 280 | 195 | 439 | 1.367796 | 0.005677 | up | ENOG410ZJIW^MAC/Perforin domain | KEGG:ath:AT1G28380 | GO:0008219^biological_process^cell death`GO:0052542^biological_process^defense response by callose deposition`GO:0006955^biological_process^immune response`GO:0009626^biological_process^plant-type hypersensitive response`GO:0010337^biological_process^regulation of salicylic acid metabolic process`GO:0009651^biological_process^response to salt stress | NSL1_ARATH^NSL1_ARATH^Q:79-1851,H:23-612^50.08%ID^E:0^RecName: Full=MACPF domain-containing protein NSL1;^Eukaryota; Viridiplantae; Streptophyta; Embryophyta; Tracheophyta; Spermatophyta; Magnoliophyta; eudicotyledons; Gunneridae; Pentapetalae; rosids; malvids; Brassicales; Brassicaceae; Camelineae; Arabidopsis |
| ERFL1c | TRIAE_CS42_3AL_TGACv1_194146_AA0627440 | 1111 | 1064 | 1159 | 327 | 356 | 594 | 1.359046 | 0.005919 | up | . | . | GO:0005634^cellular_component^nucleus`GO:0003677^molecular_function^DNA binding`GO:0003700^molecular_function^transcription factor activity, sequence-specific DNA binding`GO:0006952^biological_process^defense response`GO:0009873^biological_process^ethylene-activated signaling pathway`GO:0006351^biological_process^transcription, DNA-templated | ERF4_NICSY^ERF4_NICSY^Q:97-273,H:26-84^91.53%ID^E:3e-16^RecName: Full=Ethylene-responsive transcription factor 4;^Eukaryota; Viridiplantae; Streptophyta; Embryophyta; Tracheophyta; Spermatophyta; Magnoliophyta; eudicotyledons; Gunneridae; Pentapetalae; asterids; lamiids; Solanales; Solanaceae; Nicotianoideae; Nicotianeae; Nicotiana |
| LOC109770403 | TRIAE_CS42_2AL_TGACv1_094374_AA0296690 | 2582 | 1327 | 1366 | 467 | 472 | 1083 | 1.358216 | 0.005782 | up | ENOG410YQNQ^ethylene mediated signaling pathway | KEGG:ath:AT3G20310`KO:K09286 | GO:0005634^cellular_component^nucleus`GO:0043565^molecular_function^sequence-specific DNA binding`GO:0003700^molecular_function^transcription factor activity, sequence-specific DNA binding`GO:0044212^molecular_function^transcription regulatory region DNA binding`GO:0006952^biological_process^defense response`GO:0009873^biological_process^ethylene-activated signaling pathway`GO:0045892^biological_process^negative regulation of transcription, DNA-templated`GO:0006355^biological_process^regulation of transcription, DNA-templated`GO:0009737^biological_process^response to abscisic acid`GO:0009414^biological_process^response to water deprivation`GO:0006351^biological_process^transcription, DNA-templated | ERF83_ARATH^ERF83_ARATH^Q:52-267,H:24-95^69.44%ID^E:2e-19^RecName: Full=Ethylene-responsive transcription factor 7;^Eukaryota; Viridiplantae; Streptophyta; Embryophyta; Tracheophyta; Spermatophyta; Magnoliophyta; eudicotyledons; Gunneridae; Pentapetalae; rosids; malvids; Brassicales; Brassicaceae; Camelineae; Arabidopsis |
| LOC109778212 | TRIAE_CS42_3DL_TGACv1_249152_AA0839720 | 378 | 268 | 248 | 109 | 101 | 136 | 1.34308 | 0.008304 | up | . | KEGG:osa:4327497 | GO:0005737^cellular_component^cytoplasm`GO:0005886^cellular_component^plasma membrane`GO:0005544^molecular_function^calcium-dependent phospholipid binding`GO:0006952^biological_process^defense response | ERG1_ORYSJ^ERG1_ORYSJ^Q:16-411,H:27-159^70.68%ID^E:2e-66^RecName: Full=Elicitor-responsive protein 1;^Eukaryota; Viridiplantae; Streptophyta; Embryophyta; Tracheophyta; Spermatophyta; Magnoliophyta; Liliopsida; Poales; Poaceae; BOP clade; Oryzoideae; Oryzeae; Oryzinae; Oryza; Oryza sativa |
| AA0385640 | TRIAE_CS42_2BL_TGACv1_129484_AA0385640 | 831 | 503 | 500 | 142 | 223 | 349 | 1.33381 | 0.007754 | up | . | KEGG:nta:107778042 | GO:0005634^cellular_component^nucleus`GO:0003677^molecular_function^DNA binding`GO:0003700^molecular_function^transcription factor activity, sequence-specific DNA binding`GO:0006952^biological_process^defense response`GO:0009873^biological_process^ethylene-activated signaling pathway`GO:0006351^biological_process^transcription, DNA-templated | ERF4_TOBAC^ERF4_TOBAC^Q:46-237,H:20-83^76.56%ID^E:3e-19^RecName: Full=Ethylene-responsive transcription factor 4;^Eukaryota; Viridiplantae; Streptophyta; Embryophyta; Tracheophyta; Spermatophyta; Magnoliophyta; eudicotyledons; Gunneridae; Pentapetalae; asterids; lamiids; Solanales; Solanaceae; Nicotianoideae; Nicotianeae; Nicotiana |
| LOC109733770 | TRIAE_CS42_2DL_TGACv1_162895_AA0563810 | 1097 | 562 | 641 | 198 | 216 | 491 | 1.320152 | 0.008397 | up | ENOG410YQNQ^ethylene mediated signaling pathway | KEGG:ath:AT3G20310`KO:K09286 | GO:0005634^cellular_component^nucleus`GO:0043565^molecular_function^sequence-specific DNA binding`GO:0003700^molecular_function^transcription factor activity, sequence-specific DNA binding`GO:0044212^molecular_function^transcription regulatory region DNA binding`GO:0006952^biological_process^defense response`GO:0009873^biological_process^ethylene-activated signaling pathway`GO:0045892^biological_process^negative regulation of transcription, DNA-templated`GO:0006355^biological_process^regulation of transcription, DNA-templated`GO:0009737^biological_process^response to abscisic acid`GO:0009414^biological_process^response to water deprivation`GO:0006351^biological_process^transcription, DNA-templated | ERF83_ARATH^ERF83_ARATH^Q:10-240,H:6-85^70%ID^E:1e-19^RecName: Full=Ethylene-responsive transcription factor 7;^Eukaryota; Viridiplantae; Streptophyta; Embryophyta; Tracheophyta; Spermatophyta; Magnoliophyta; eudicotyledons; Gunneridae; Pentapetalae; rosids; malvids; Brassicales; Brassicaceae; Camelineae; Arabidopsis |
| LOC109764611 | TRIAE_CS42_4AL_TGACv1_291630_AA0995690 | 24 | 44 | 22 | 56 | 103 | 77 | -1.41518 | 0.00968 | down | COG4886^leucine Rich Repeat | KEGG:ath:AT3G14460 | GO:0005886^cellular_component^plasma membrane`GO:0043531^molecular_function^ADP binding`GO:0005524^molecular_function^ATP binding`GO:0006952^biological_process^defense response`GO:0007165^biological_process^signal transduction | DRL21_ARATH^DRL21_ARATH^Q:256-1827,H:190-784^30.97%ID^E:5e-66^RecName: Full=Putative disease resistance protein At3g14460;^Eukaryota; Viridiplantae; Streptophyta; Embryophyta; Tracheophyta; Spermatophyta; Magnoliophyta; eudicotyledons; Gunneridae; Pentapetalae; rosids; malvids; Brassicales; Brassicaceae; Camelineae; Arabidopsis |
| LOC109738912 | TRIAE_CS42_3B_TGACv1_221435_AA0740740 | 229 | 232 | 319 | 675 | 797 | 591 | -1.42918 | 0.003415 | down | . | . | GO:0030598^molecular_function^rRNA N-glycosylase activity`GO:0006952^biological_process^defense response`GO:0017148^biological_process^negative regulation of translation | JI60_HORVU^JI60_HORVU^Q:2920-3513,H:8-234^24.02%ID^E:7e-06^RecName: Full=60 kDa jasmonate-induced protein;^Eukaryota; Viridiplantae; Streptophyta; Embryophyta; Tracheophyta; Spermatophyta; Magnoliophyta; Liliopsida; Poales; Poaceae; BOP clade; Pooideae; Triticodae; Triticeae; Hordeinae; Hordeum |
| LOC109751680 | TRIAE_CS42_2BS_TGACv1_146600_AA0469170 | 265 | 213 | 416 | 959 | 1048 | 564 | -1.54983 | 0.001102 | down | . | . | GO:0043531^molecular_function^ADP binding`GO:0005524^molecular_function^ATP binding`GO:0006952^biological_process^defense response | RGA4_SOLBU^RGA4_SOLBU^Q:2467-3687,H:627-963^23.88%ID^E:1e-05^RecName: Full=Putative disease resistance protein RGA4;^Eukaryota; Viridiplantae; Streptophyta; Embryophyta; Tracheophyta; Spermatophyta; Magnoliophyta; eudicotyledons; Gunneridae; Pentapetalae; asterids; lamiids; Solanales; Solanaceae; Solanoideae; Solaneae; Solanum |
| LOC109773000 | TRIAE_CS42_1AL_TGACv1_000540_AA0014260 | 34 | 23 | 44 | 66 | 110 | 121 | -1.57904 | 0.002279 | down | COG4886^leucine Rich Repeat | KEGG:ath:AT3G46530 | GO:0005737^cellular_component^cytoplasm`GO:0005886^cellular_component^plasma membrane`GO:0043531^molecular_function^ADP binding`GO:0005524^molecular_function^ATP binding`GO:0009814^biological_process^defense response, incompatible interaction`GO:0009626^biological_process^plant-type hypersensitive response`GO:0007165^biological_process^signal transduction | RPP13_ARATH^RPP13_ARATH^Q:19-1533,H:9-519^30.71%ID^E:6e-56^RecName: Full=Disease resistance protein RPP13;^Eukaryota; Viridiplantae; Streptophyta; Embryophyta; Tracheophyta; Spermatophyta; Magnoliophyta; eudicotyledons; Gunneridae; Pentapetalae; rosids; malvids; Brassicales; Brassicaceae; Camelineae; Arabidopsis |
| LOC109737946 | TRIAE_CS42_2BL_TGACv1_131855_AA0432910 | 44 | 43 | 33 | 126 | 152 | 95 | -1.66009 | 0.000925 | down | ENOG410YXDY^Lipid-transfer protein | KEGG:ath:AT5G48485 | GO:0048046^cellular_component^apoplast`GO:0005783^cellular_component^endoplasmic reticulum`GO:0009506^cellular_component^plasmodesma`GO:0005504^molecular_function^fatty acid binding`GO:0043621^molecular_function^protein self-association`GO:0008270^molecular_function^zinc ion binding`GO:0006869^biological_process^lipid transport`GO:0009627^biological_process^systemic acquired resistance`GO:0009862^biological_process^systemic acquired resistance, salicylic acid mediated signaling pathway | DIRL1_ARATH^DIRL1_ARATH^Q:82-294,H:29-96^47.89%ID^E:8e-14^RecName: Full=Putative lipid-transfer protein DIR1;^Eukaryota; Viridiplantae; Streptophyta; Embryophyta; Tracheophyta; Spermatophyta; Magnoliophyta; eudicotyledons; Gunneridae; Pentapetalae; rosids; malvids; Brassicales; Brassicaceae; Camelineae; Arabidopsis |
| LOC117861544 | TRIAE_CS42_U_TGACv1_642399_AA2116970 | 31 | 56 | 66 | 160 | 160 | 161 | -1.6751 | 0.00065 | down | . | . | GO:0043531^molecular_function^ADP binding`GO:0005524^molecular_function^ATP binding`GO:0006952^biological_process^defense response | RGA2_SOLBU^RGA2_SOLBU^Q:472-2502,H:112-760^35.24%ID^E:7e-106^RecName: Full=Disease resistance protein RGA2;^Eukaryota; Viridiplantae; Streptophyta; Embryophyta; Tracheophyta; Spermatophyta; Magnoliophyta; eudicotyledons; Gunneridae; Pentapetalae; asterids; lamiids; Solanales; Solanaceae; Solanoideae; Solaneae; Solanum |
| AA0705620 | TRIAE_CS42_3AS_TGACv1_213134_AA0705620 | 78 | 140 | 68 | 407 | 194 | 313 | -1.69762 | 0.00035 | down | ENOG410YAV4^Auxin-induced protein | KEGG:ath:AT1G75500 | GO:0016021^cellular_component^integral component of membrane`GO:0009705^cellular_component^plant-type vacuole membrane`GO:0005886^cellular_component^plasma membrane`GO:0005774^cellular_component^vacuolar membrane`GO:0022857^molecular_function^transmembrane transporter activity`GO:0009851^biological_process^auxin biosynthetic process`GO:0010315^biological_process^auxin efflux`GO:0009734^biological_process^auxin-activated signaling pathway`GO:0071555^biological_process^cell wall organization`GO:0006952^biological_process^defense response`GO:0009834^biological_process^plant-type secondary cell wall biogenesis`GO:0090355^biological_process^positive regulation of auxin metabolic process`GO:0090358^biological_process^positive regulation of tryptophan metabolic process`GO:0000162^biological_process^tryptophan biosynthetic process`GO:0009826^biological_process^unidimensional cell growth | WAT1_ARATH^WAT1_ARATH^Q:1-1146,H:1-388^73.97%ID^E:0^RecName: Full=Protein WALLS ARE THIN 1;^Eukaryota; Viridiplantae; Streptophyta; Embryophyta; Tracheophyta; Spermatophyta; Magnoliophyta; eudicotyledons; Gunneridae; Pentapetalae; rosids; malvids; Brassicales; Brassicaceae; Camelineae; Arabidopsis |
| LOC109751562 | TRIAE_CS42_2DL_TGACv1_159977_AA0545060 | 225 | 153 | 143 | 496 | 544 | 629 | -1.70503 | 0.000261 | down | . | KEGG:osa:9270267`KO:K14431 | GO:0005634^cellular_component^nucleus`GO:0043565^molecular_function^sequence-specific DNA binding`GO:0003700^molecular_function^transcription factor activity, sequence-specific DNA binding`GO:0006952^biological_process^defense response`GO:0006351^biological_process^transcription, DNA-templated | TGAL6_ORYSJ^TGAL6_ORYSJ^Q:112-1191,H:50-397^62.84%ID^E:7e-136^RecName: Full=Transcription factor TGAL6 {ECO:0000305};^Eukaryota; Viridiplantae; Streptophyta; Embryophyta; Tracheophyta; Spermatophyta; Magnoliophyta; Liliopsida; Poales; Poaceae; BOP clade; Oryzoideae; Oryzeae; Oryzinae; Oryza; Oryza sativa |
| LOC109740535 | TRIAE_CS42_3B_TGACv1_224223_AA0794090 | 107 | 73 | 66 | 273 | 313 | 262 | -1.81054 | 0.000118 | down | ENOG410Y5C6^e3 ubiquitin protein ligase | KEGG:osa:4349420`KO:K10696 | GO:0005634^cellular_component^nucleus`GO:0046872^molecular_function^metal ion binding`GO:0042803^molecular_function^protein homodimerization activity`GO:0016740^molecular_function^transferase activity`GO:0033523^biological_process^histone H2B ubiquitination`GO:0010390^biological_process^histone monoubiquitination`GO:0045087^biological_process^innate immune response`GO:0009965^biological_process^leaf morphogenesis`GO:0010162^biological_process^seed dormancy process`GO:0010228^biological_process^vegetative to reproductive phase transition of meristem | BRE1B_ORYSJ^BRE1B_ORYSJ^Q:1-1545,H:1-515^76.5%ID^E:0^RecName: Full=E3 ubiquitin-protein ligase BRE1-like 2;^Eukaryota; Viridiplantae; Streptophyta; Embryophyta; Tracheophyta; Spermatophyta; Magnoliophyta; Liliopsida; Poales; Poaceae; BOP clade; Oryzoideae; Oryzeae; Oryzinae; Oryza; Oryza sativa |
| AA2123920 | TRIAE_CS42_U_TGACv1_642833_AA2123920 | 208 | 302 | 141 | 583 | 684 | 1272 | -1.98861 | 1.03E-05 | down | . | . | GO:0030598^molecular_function^rRNA N-glycosylase activity`GO:0006952^biological_process^defense response`GO:0017148^biological_process^negative regulation of translation | JI60_HORVU^JI60_HORVU^Q:232-873,H:52-291^26.56%ID^E:6e-15^RecName: Full=60 kDa jasmonate-induced protein;^Eukaryota; Viridiplantae; Streptophyta; Embryophyta; Tracheophyta; Spermatophyta; Magnoliophyta; Liliopsida; Poales; Poaceae; BOP clade; Pooideae; Triticodae; Triticeae; Hordeinae; Hordeum |
| LOC109754231 | TRIAE_CS42_4AL_TGACv1_288745_AA0957120 | 11 | 8 | 23 | 37 | 73 | 65 | -2.07555 | 8.47E-05 | down | COG4886^leucine Rich Repeat | KEGG:ath:AT1G50180 | GO:0005886^cellular_component^plasma membrane`GO:0009506^cellular_component^plasmodesma`GO:0043531^molecular_function^ADP binding`GO:0005524^molecular_function^ATP binding`GO:0006952^biological_process^defense response`GO:0007165^biological_process^signal transduction | DRL4_ARATH^DRL4_ARATH^Q:1765-3474,H:58-625^29.73%ID^E:6e-54^RecName: Full=Putative disease resistance protein At1g50180;^Eukaryota; Viridiplantae; Streptophyta; Embryophyta; Tracheophyta; Spermatophyta; Magnoliophyta; eudicotyledons; Gunneridae; Pentapetalae; rosids; malvids; Brassicales; Brassicaceae; Camelineae; Arabidopsis |
| LOC109750033 | TRIAE_CS42_2AL_TGACv1_094756_AA0302660 | 17 | 15 | 27 | 111 | 73 | 65 | -2.09351 | 3.18E-05 | down | . | . | GO:0043531^molecular_function^ADP binding`GO:0005524^molecular_function^ATP binding`GO:0006952^biological_process^defense response | RGA4_SOLBU^RGA4_SOLBU^Q:232-3315,H:1-988^26.26%ID^E:7e-85^RecName: Full=Putative disease resistance protein RGA4;^Eukaryota; Viridiplantae; Streptophyta; Embryophyta; Tracheophyta; Spermatophyta; Magnoliophyta; eudicotyledons; Gunneridae; Pentapetalae; asterids; lamiids; Solanales; Solanaceae; Solanoideae; Solaneae; Solanum |
| LOC109771590 | TRIAE_CS42_2DL_TGACv1_158074_AA0508010 | 303 | 337 | 329 | 1440 | 1363 | 1307 | -2.10962 | 2.15E-06 | down | ENOG410XQVB^DiacylGlycerol Kinase | KEGG:ath:AT2G20900`KO:K00901 | GO:0005524^molecular_function^ATP binding`GO:0004143^molecular_function^diacylglycerol kinase activity`GO:0003951^molecular_function^NAD+ kinase activity`GO:0006952^biological_process^defense response`GO:0007205^biological_process^protein kinase C-activating G-protein coupled receptor signaling pathway | DGK5_ARATH^DGK5_ARATH^Q:67-1485,H:11-484^67.92%ID^E:0^RecName: Full=Diacylglycerol kinase 5;^Eukaryota; Viridiplantae; Streptophyta; Embryophyta; Tracheophyta; Spermatophyta; Magnoliophyta; eudicotyledons; Gunneridae; Pentapetalae; rosids; malvids; Brassicales; Brassicaceae; Camelineae; Arabidopsis |
| LOC109781948 | TRIAE_CS42_2BL_TGACv1_139496_AA0445540 | 39 | 33 | 24 | 180 | 153 | 90 | -2.1607 | 5.96E-06 | down | ENOG410YXDY^Lipid-transfer protein | KEGG:ath:AT5G48485 | GO:0048046^cellular_component^apoplast`GO:0005783^cellular_component^endoplasmic reticulum`GO:0009506^cellular_component^plasmodesma`GO:0005504^molecular_function^fatty acid binding`GO:0043621^molecular_function^protein self-association`GO:0008270^molecular_function^zinc ion binding`GO:0006869^biological_process^lipid transport`GO:0009627^biological_process^systemic acquired resistance`GO:0009862^biological_process^systemic acquired resistance, salicylic acid mediated signaling pathway | DIRL1_ARATH^DIRL1_ARATH^Q:40-252,H:29-96^47.89%ID^E:4e-14^RecName: Full=Putative lipid-transfer protein DIR1;^Eukaryota; Viridiplantae; Streptophyta; Embryophyta; Tracheophyta; Spermatophyta; Magnoliophyta; eudicotyledons; Gunneridae; Pentapetalae; rosids; malvids; Brassicales; Brassicaceae; Camelineae; Arabidopsis |
| LOC109786040 | TRIAE_CS42_3B_TGACv1_224601_AA0798610 | 41 | 21 | 33 | 204 | 143 | 97 | -2.24426 | 2.23E-06 | down | . | . | GO:0043531^molecular_function^ADP binding`GO:0005524^molecular_function^ATP binding`GO:0006952^biological_process^defense response | RGA2_SOLBU^RGA2_SOLBU^Q:19-1035,H:4-342^23.51%ID^E:5e-07^RecName: Full=Disease resistance protein RGA2;^Eukaryota; Viridiplantae; Streptophyta; Embryophyta; Tracheophyta; Spermatophyta; Magnoliophyta; eudicotyledons; Gunneridae; Pentapetalae; asterids; lamiids; Solanales; Solanaceae; Solanoideae; Solaneae; Solanum |
| LOC109783813 | TRIAE_CS42_5AL_TGACv1_375025_AA1214150 | 244 | 251 | 240 | 1768 | 1164 | 712 | -2.33337 | 1.26E-07 | down | ENOG410ZUVB^May be involved in modulation of pathogen defense and leaf cell death (By similarity) | . | GO:0016021^cellular_component^integral component of membrane`GO:0005516^molecular_function^calmodulin binding`GO:0006952^biological_process^defense response`GO:0009607^biological_process^response to biotic stimulus | MLO_HORVU^MLO_HORVU^Q:25-1602,H:8-533^89.02%ID^E:0^RecName: Full=Protein MLO;^Eukaryota; Viridiplantae; Streptophyta; Embryophyta; Tracheophyta; Spermatophyta; Magnoliophyta; Liliopsida; Poales; Poaceae; BOP clade; Pooideae; Triticodae; Triticeae; Hordeinae; Hordeum |
| LOC109750038 | TRIAE_CS42_2DL_TGACv1_159908_AA0544220 | 155 | 107 | 165 | 978 | 942 | 1192 | -2.88939 | 5.11E-11 | down | COG1310^Component of the eukaryotic translation initiation factor 3 (eIF-3) complex, which is involved in protein synthesis and, together with other initiation factors, stimulates binding of mRNA and methionyl-tRNAi to the 40S ribosome (By similarity) | KEGG:ath:AT5G05780`KO:K03038 | GO:0005829^cellular_component^cytosol`GO:0000502^cellular_component^proteasome complex`GO:0005838^cellular_component^proteasome regulatory particle`GO:0045087^biological_process^innate immune response`GO:0009965^biological_process^leaf morphogenesis`GO:0043161^biological_process^proteasome-mediated ubiquitin-dependent protein catabolic process | PSD7A_ARATH^PSD7A_ARATH^Q:1-924,H:1-308^89.94%ID^E:0^RecName: Full=26S proteasome non-ATPase regulatory subunit 7 homolog A;^Eukaryota; Viridiplantae; Streptophyta; Embryophyta; Tracheophyta; Spermatophyta; Magnoliophyta; eudicotyledons; Gunneridae; Pentapetalae; rosids; malvids; Brassicales; Brassicaceae; Camelineae; Arabidopsis |
| AA0445950 | TRIAE_CS42_2BL_TGACv1_143683_AA0445950 | 9 | 5 | 11 | 93 | 72 | 39 | -3.03297 | 7.04E-09 | down | ENOG410YXDY^Lipid-transfer protein | KEGG:ath:AT5G48485 | GO:0048046^cellular_component^apoplast`GO:0005783^cellular_component^endoplasmic reticulum`GO:0009506^cellular_component^plasmodesma`GO:0005504^molecular_function^fatty acid binding`GO:0043621^molecular_function^protein self-association`GO:0008270^molecular_function^zinc ion binding`GO:0006869^biological_process^lipid transport`GO:0009627^biological_process^systemic acquired resistance`GO:0009862^biological_process^systemic acquired resistance, salicylic acid mediated signaling pathway | DIRL1_ARATH^DIRL1_ARATH^Q:1-204,H:32-96^47.06%ID^E:2e-12^RecName: Full=Putative lipid-transfer protein DIR1;^Eukaryota; Viridiplantae; Streptophyta; Embryophyta; Tracheophyta; Spermatophyta; Magnoliophyta; eudicotyledons; Gunneridae; Pentapetalae; rosids; malvids; Brassicales; Brassicaceae; Camelineae; Arabidopsis |
| LOC109778124 | TRIAE_CS42_5BL_TGACv1_406159_AA1341520 | 27 | 19 | 25 | 183 | 153 | 248 | -3.05724 | 6.44E-11 | down | . | . | GO:0005576^cellular_component^extracellular region`GO:0005975^biological_process^carbohydrate metabolic process`GO:0006952^biological_process^defense response | XIP1_WHEAT^XIP1_WHEAT^Q:7-897,H:4-304^55.92%ID^E:2e-85^RecName: Full=Xylanase inhibitor protein 1;^Eukaryota; Viridiplantae; Streptophyta; Embryophyta; Tracheophyta; Spermatophyta; Magnoliophyta; Liliopsida; Poales; Poaceae; BOP clade; Pooideae; Triticodae; Triticeae; Triticinae; Triticum |
| LOC100827979 | TRIAE_CS42_1DL_TGACv1_062188_AA0210280 | 12 | 5 | 11 | 105 | 59 | 78 | -3.11626 | 1.23E-09 | down | ENOG410Y41C^Metacaspase | KEGG:ath:AT1G02170 | GO:0004197^molecular_function^cysteine-type endopeptidase activity`GO:0006952^biological_process^defense response`GO:0043068^biological_process^positive regulation of programmed cell death | MCA1_ARATH^MCA1_ARATH^Q:43-1146,H:18-367^49.6%ID^E:5e-115^RecName: Full=Metacaspase-1;^Eukaryota; Viridiplantae; Streptophyta; Embryophyta; Tracheophyta; Spermatophyta; Magnoliophyta; eudicotyledons; Gunneridae; Pentapetalae; rosids; malvids; Brassicales; Brassicaceae; Camelineae; Arabidopsis |
| LOC109747282 | TRIAE_CS42_5BS_TGACv1_424493_AA1390090 | 6 | 6 | 13 | 86 | 66 | 67 | -3.13444 | 1.76E-09 | down | COG4886^leucine Rich Repeat | KEGG:ath:AT1G58390 | GO:0005886^cellular_component^plasma membrane`GO:0043531^molecular_function^ADP binding`GO:0005524^molecular_function^ATP binding`GO:0006952^biological_process^defense response`GO:0007165^biological_process^signal transduction | DRL7_ARATH^DRL7_ARATH^Q:70-909,H:17-303^22.71%ID^E:4e-10^RecName: Full=Probable disease resistance protein At1g58390;^Eukaryota; Viridiplantae; Streptophyta; Embryophyta; Tracheophyta; Spermatophyta; Magnoliophyta; eudicotyledons; Gunneridae; Pentapetalae; rosids; malvids; Brassicales; Brassicaceae; Camelineae; Arabidopsis |
| AA0403640 | TRIAE_CS42_2BL_TGACv1_130094_AA0403640 | 17 | 14 | 8 | 149 | 104 | 134 | -3.32173 | 1.18E-11 | down | ENOG410XQ8V^synthase | KEGG:ath:AT4G03550`KO:K11000 | GO:0000148^cellular_component^1,3-beta-D-glucan synthase complex`GO:0005794^cellular_component^Golgi apparatus`GO:0016021^cellular_component^integral component of membrane`GO:0005886^cellular_component^plasma membrane`GO:0009506^cellular_component^plasmodesma`GO:0003843^molecular_function^1,3-beta-D-glucan synthase activity`GO:0006075^biological_process^(1->3)-beta-D-glucan biosynthetic process`GO:0006952^biological_process^defense response`GO:0052542^biological_process^defense response by callose deposition`GO:0052544^biological_process^defense response by callose deposition in cell wall`GO:0009870^biological_process^defense response signaling pathway, resistance gene-dependent`GO:0042742^biological_process^defense response to bacterium`GO:0050832^biological_process^defense response to fungus`GO:0009965^biological_process^leaf morphogenesis`GO:0010150^biological_process^leaf senescence`GO:0009555^biological_process^pollen development`GO:0008360^biological_process^regulation of cell shape`GO:0000003^biological_process^reproduction`GO:0009620^biological_process^response to fungus`GO:0009863^biological_process^salicylic acid mediated signaling pathway | CALSC_ARATH^CALSC_ARATH^Q:49-3174,H:475-1517^65.65%ID^E:0^RecName: Full=Callose synthase 12;^Eukaryota; Viridiplantae; Streptophyta; Embryophyta; Tracheophyta; Spermatophyta; Magnoliophyta; eudicotyledons; Gunneridae; Pentapetalae; rosids; malvids; Brassicales; Brassicaceae; Camelineae; Arabidopsis |
| LOC100830206 | TRIAE_CS42_2BL_TGACv1_130094_AA0403640 | 17 | 14 | 8 | 149 | 104 | 134 | -3.32173 | 1.18E-11 | down | ENOG410XQ8V^synthase | KEGG:ath:AT4G03550`KO:K11000 | GO:0000148^cellular_component^1,3-beta-D-glucan synthase complex`GO:0005794^cellular_component^Golgi apparatus`GO:0016021^cellular_component^integral component of membrane`GO:0005886^cellular_component^plasma membrane`GO:0009506^cellular_component^plasmodesma`GO:0003843^molecular_function^1,3-beta-D-glucan synthase activity`GO:0006075^biological_process^(1->3)-beta-D-glucan biosynthetic process`GO:0006952^biological_process^defense response`GO:0052542^biological_process^defense response by callose deposition`GO:0052544^biological_process^defense response by callose deposition in cell wall`GO:0009870^biological_process^defense response signaling pathway, resistance gene-dependent`GO:0042742^biological_process^defense response to bacterium`GO:0050832^biological_process^defense response to fungus`GO:0009965^biological_process^leaf morphogenesis`GO:0010150^biological_process^leaf senescence`GO:0009555^biological_process^pollen development`GO:0008360^biological_process^regulation of cell shape`GO:0000003^biological_process^reproduction`GO:0009620^biological_process^response to fungus`GO:0009863^biological_process^salicylic acid mediated signaling pathway | CALSC_ARATH^CALSC_ARATH^Q:49-3174,H:475-1517^65.65%ID^E:0^RecName: Full=Callose synthase 12;^Eukaryota; Viridiplantae; Streptophyta; Embryophyta; Tracheophyta; Spermatophyta; Magnoliophyta; eudicotyledons; Gunneridae; Pentapetalae; rosids; malvids; Brassicales; Brassicaceae; Camelineae; Arabidopsis |
| LOC109732331 | TRIAE_CS42_3B_TGACv1_221313_AA0736930 | 20 | 11 | 11 | 171 | 105 | 173 | -3.42929 | 1.60E-12 | down | ENOG410ZNK7^Somatic embryogenesis receptor kinase | KEGG:osa:4336035 | GO:0016021^cellular_component^integral component of membrane`GO:0005886^cellular_component^plasma membrane`GO:0005524^molecular_function^ATP binding`GO:0005102^molecular_function^receptor binding`GO:0004675^molecular_function^transmembrane receptor protein serine/threonine kinase activity`GO:0009742^biological_process^brassinosteroid mediated signaling pathway`GO:0030154^biological_process^cell differentiation`GO:0007166^biological_process^cell surface receptor signaling pathway`GO:0006952^biological_process^defense response`GO:0006468^biological_process^protein phosphorylation | SERK2_ORYSJ^SERK2_ORYSJ^Q:67-537,H:31-187^71.34%ID^E:3e-65^RecName: Full=LRR receptor kinase SERK2 {ECO:0000305};^Eukaryota; Viridiplantae; Streptophyta; Embryophyta; Tracheophyta; Spermatophyta; Magnoliophyta; Liliopsida; Poales; Poaceae; BOP clade; Oryzoideae; Oryzeae; Oryzinae; Oryza; Oryza sativa |
| LOC109773754 | TRIAE_CS42_2AS_TGACv1_114810_AA0369470 | 9 | 16 | 9 | 157 | 181 | 165 | -3.89734 | 2.67E-15 | down | . | . | GO:0043531^molecular_function^ADP binding`GO:0005524^molecular_function^ATP binding`GO:0006952^biological_process^defense response | RGA4_SOLBU^RGA4_SOLBU^Q:355-1704,H:142-606^26.46%ID^E:1e-42^RecName: Full=Putative disease resistance protein RGA4;^Eukaryota; Viridiplantae; Streptophyta; Embryophyta; Tracheophyta; Spermatophyta; Magnoliophyta; eudicotyledons; Gunneridae; Pentapetalae; asterids; lamiids; Solanales; Solanaceae; Solanoideae; Solaneae; Solanum |
| WRKY45 | TRIAE_CS42_2DL_TGACv1_158819_AA0526620 | 141 | 101 | 61 | 1591 | 1216 | 1957 | -3.99777 | 6.20E-19 | down | ENOG410YZZY^Transcription factor | KEGG:ath:AT3G56400 | GO:0005634^cellular_component^nucleus`GO:0043565^molecular_function^sequence-specific DNA binding`GO:0003700^molecular_function^transcription factor activity, sequence-specific DNA binding`GO:0042742^biological_process^defense response to bacterium`GO:0050832^biological_process^defense response to fungus`GO:0009864^biological_process^induced systemic resistance, jasmonic acid mediated signaling pathway`GO:1900056^biological_process^negative regulation of leaf senescence`GO:0045892^biological_process^negative regulation of transcription, DNA-templated`GO:0031347^biological_process^regulation of defense response`GO:0010200^biological_process^response to chitin`GO:0009753^biological_process^response to jasmonic acid`GO:0009751^biological_process^response to salicylic acid`GO:0009862^biological_process^systemic acquired resistance, salicylic acid mediated signaling pathway`GO:0006351^biological_process^transcription, DNA-templated | WRK70_ARATH^WRK70_ARATH^Q:256-492,H:102-180^50.63%ID^E:2e-14^RecName: Full=Probable WRKY transcription factor 70;^Eukaryota; Viridiplantae; Streptophyta; Embryophyta; Tracheophyta; Spermatophyta; Magnoliophyta; eudicotyledons; Gunneridae; Pentapetalae; rosids; malvids; Brassicales; Brassicaceae; Camelineae; Arabidopsis |
| LOC109755931 | TRIAE_CS42_2DL_TGACv1_158102_AA0509170 | 9 | 7 | 6 | 186 | 141 | 217 | -4.62685 | 2.44E-19 | down | COG0515^Serine Threonine protein kinase | KEGG:ath:AT5G47070`KO:K00924 | GO:0005886^cellular_component^plasma membrane`GO:0005524^molecular_function^ATP binding`GO:0004675^molecular_function^transmembrane receptor protein serine/threonine kinase activity`GO:0007166^biological_process^cell surface receptor signaling pathway`GO:0006952^biological_process^defense response`GO:0006468^biological_process^protein phosphorylation | PBL19_ARATH^PBL19_ARATH^Q:172-1158,H:39-367^52.68%ID^E:8e-117^RecName: Full=Probable serine/threonine-protein kinase PBL19 {ECO:0000305};^Eukaryota; Viridiplantae; Streptophyta; Embryophyta; Tracheophyta; Spermatophyta; Magnoliophyta; eudicotyledons; Gunneridae; Pentapetalae; rosids; malvids; Brassicales; Brassicaceae; Camelineae; Arabidopsis |
| LOC109770200 | TRIAE_CS42_2BS_TGACv1_149972_AA0497860 | 15 | 14 | 7 | 309 | 260 | 353 | -4.68809 | 2.27E-21 | down | COG0515^Serine Threonine protein kinase | KEGG:ath:AT1G69790`KO:K04733 | GO:0005886^cellular_component^plasma membrane`GO:0005524^molecular_function^ATP binding`GO:0004675^molecular_function^transmembrane receptor protein serine/threonine kinase activity`GO:0007166^biological_process^cell surface receptor signaling pathway`GO:0006952^biological_process^defense response`GO:0006468^biological_process^protein phosphorylation | PBL18_ARATH^PBL18_ARATH^Q:205-1116,H:68-365^62.5%ID^E:2e-134^RecName: Full=Probable serine/threonine-protein kinase PBL18 {ECO:0000305};^Eukaryota; Viridiplantae; Streptophyta; Embryophyta; Tracheophyta; Spermatophyta; Magnoliophyta; eudicotyledons; Gunneridae; Pentapetalae; rosids; malvids; Brassicales; Brassicaceae; Camelineae; Arabidopsis |
| LOC114761184 | TRIAE_CS42_6BL_TGACv1_509328_AA1629790 | 48 | 32 | 17 | 929 | 842 | 851 | -4.77666 | 4.37E-24 | down | . | . | GO:0005789^cellular_component^endoplasmic reticulum membrane`GO:0016021^cellular_component^integral component of membrane`GO:0005634^cellular_component^nucleus`GO:0005886^cellular_component^plasma membrane`GO:0005524^molecular_function^ATP binding`GO:0004674^molecular_function^protein serine/threonine kinase activity`GO:0006952^biological_process^defense response`GO:0031349^biological_process^positive regulation of defense response`GO:1900426^biological_process^positive regulation of defense response to bacterium`GO:0046777^biological_process^protein autophosphorylation | XA21_ORYSI^XA21_ORYSI^Q:1-681,H:789-1013^42.11%ID^E:1e-48^RecName: Full=Receptor kinase-like protein Xa21 {ECO:0000303\|Ref.1};^Eukaryota; Viridiplantae; Streptophyta; Embryophyta; Tracheophyta; Spermatophyta; Magnoliophyta; Liliopsida; Poales; Poaceae; BOP clade; Oryzoideae; Oryzeae; Oryzinae; Oryza; Oryza sativa |
| LOC109772999 | TRIAE_CS42_1BL_TGACv1_031054_AA0106850 | 5 | 3 | 2 | 208 | 163 | 169 | -5.72293 | 3.38E-24 | down | COG4886^leucine Rich Repeat | KEGG:ath:AT3G46530 | GO:0005737^cellular_component^cytoplasm`GO:0005886^cellular_component^plasma membrane`GO:0043531^molecular_function^ADP binding`GO:0005524^molecular_function^ATP binding`GO:0009814^biological_process^defense response, incompatible interaction`GO:0009626^biological_process^plant-type hypersensitive response`GO:0007165^biological_process^signal transduction | RPP13_ARATH^RPP13_ARATH^Q:22-2601,H:1-777^26.81%ID^E:3e-63^RecName: Full=Disease resistance protein RPP13;^Eukaryota; Viridiplantae; Streptophyta; Embryophyta; Tracheophyta; Spermatophyta; Magnoliophyta; eudicotyledons; Gunneridae; Pentapetalae; rosids; malvids; Brassicales; Brassicaceae; Camelineae; Arabidopsis |
