# Supplementary material for: A cosmopolitan fungal pathogen of dicots adopts an endophytic lifestyle on cereal crops and protects them from major fungal diseases
Source: ISME J. 2020 Aug 19;14(12):3120–35. doi: 10.1038/s41396-020-00744-6 (PMC7784893; doi:10.1038/s41396-020-00744-6)
Supplement: Supplementary file 3 — Supplementary Table 3 [file 41396_2020_744_MOESM3_ESM.docx]

**Supplementary Table 3** Wheat DEGs associated with the Pattern-Triggered Immunity (PTI) pathway in DT-8 treated and control wheat spikes

| **gene** | **gene_id** | **DT-8 Sample 1_count** | **DT-8 Sample 2_count** | **DT-8 Sample 3_count** | **Control Sample 1_count** | **Control Sample 2_count** | **Control Sample 3_count** | **logFC** | **FDR** | **exp** | **eggnog** | **Kegg** | **GO** | **uniprot_hit** |
| --- | --- | --- | --- | --- | --- | --- | --- | --- | --- | --- | --- | --- | --- | --- |
| LOC109768055 | TRIAE_CS42_3DS_TGACv1_272365_AA0919640 | 488 | 657 | 496 | 227 | 151 | 197 | 1.488267 | 0.002114 | up | COG5126^Calcium-binding protein | KEGG:osa:4325693`KO:K13448 | GO:0005509^molecular_function^calcium ion binding | CML16_ORYSJ^CML16_ORYSJ^Q:214-702,H:25-179^74.23%ID^E:4e-69^RecName: Full=Probable calcium-binding protein CML16;^Eukaryota; Viridiplantae; Streptophyta; Embryophyta; Tracheophyta; Spermatophyta; Magnoliophyta; Liliopsida; Poales; Poaceae; BOP clade; Oryzoideae; Oryzeae; Oryzinae; Oryza; Oryza sativa |
| LOC109753075 | TRIAE_CS42_1AL_TGACv1_004249_AA0052940 | 700 | 848 | 1074 | 181 | 352 | 421 | 1.431124 | 0.003265 | up | COG5126^Calcium-binding protein | KEGG:osa:4325762`KO:K13448 | GO:0005509^molecular_function^calcium ion binding | CML10_ORYSJ^CML10_ORYSJ^Q:118-312,H:112-176^43.08%ID^E:5e-09^RecName: Full=Probable calcium-binding protein CML10;^Eukaryota; Viridiplantae; Streptophyta; Embryophyta; Tracheophyta; Spermatophyta; Magnoliophyta; Liliopsida; Poales; Poaceae; BOP clade; Oryzoideae; Oryzeae; Oryzinae; Oryza; Oryza sativa |
| LOC109771128 | TRIAE_CS42_3AL_TGACv1_193824_AA0620270 | 184 | 123 | 116 | 43 | 58 | 60 | 1.364452 | 0.009494 | up | COG5126^Calcium-binding protein | KEGG:osa:4325408`KO:K13448 | GO:0005509^molecular_function^calcium ion binding | CML31_ORYSJ^CML31_ORYSJ^Q:7-456,H:6-150^64.67%ID^E:1e-46^RecName: Full=Probable calcium-binding protein CML31;^Eukaryota; Viridiplantae; Streptophyta; Embryophyta; Tracheophyta; Spermatophyta; Magnoliophyta; Liliopsida; Poales; Poaceae; BOP clade; Oryzoideae; Oryzeae; Oryzinae; Oryza; Oryza sativa |
