# Supplementary material for: A cosmopolitan fungal pathogen of dicots adopts an endophytic lifestyle on cereal crops and protects them from major fungal diseases
Source: ISME J. 2020 Aug 19;14(12):3120–35. doi: 10.1038/s41396-020-00744-6 (PMC7784893; doi:10.1038/s41396-020-00744-6)
Supplement: Supplementary file 4 — Supplementary Table 4 [file 41396_2020_744_MOESM4_ESM.docx]

**Supplementary Table 4** Wheat DEGs associated with the defense response in DT-8 treated and control wheat flag leaves

| gene | gene_id | **DT-8 Sample 1_count** | **DT-8 Sample 2_count** | **DT-8 Sample 3_count** | **Control Sample 1_count** | **Control Sample 2_count** | **Control Sample 3_count** | logFC | FDR | exp | eggnog | Kegg | GO | uniprot_hit |
| --- | --- | --- | --- | --- | --- | --- | --- | --- | --- | --- | --- | --- | --- | --- |
| LOC109775479 | TRIAE_CS42_3DS_TGACv1_271932_AA0911260 | 334 | 565 | 511 | 10 | 6 | 7 | 5.901103 | 1.34E-29 | up | . | . | GO:0005789^cellular_component^endoplasmic reticulum membrane`GO:0016021^cellular_component^integral component of membrane`GO:0005634^cellular_component^nucleus`GO:0005886^cellular_component^plasma membrane`GO:0005524^molecular_function^ATP binding`GO:0004674^molecular_function^protein serine/threonine kinase activity`GO:0006952^biological_process^defense response`GO:0031349^biological_process^positive regulation of defense response`GO:1900426^biological_process^positive regulation of defense response to bacterium`GO:0046777^biological_process^protein autophosphorylation | XA21_ORYSI^XA21_ORYSI^Q:262-612,H:91-238^32.43%ID^E:5e-08^RecName: Full=Receptor kinase-like protein Xa21 {ECO:0000303\|Ref.1};^Eukaryota; Viridiplantae; Streptophyta; Embryophyta; Tracheophyta; Spermatophyta; Magnoliophyta; Liliopsida; Poales; Poaceae; BOP clade; Oryzoideae; Oryzeae; Oryzinae; Oryza; Oryza sativa |
| LOC109774315 | TRIAE_CS42_4DS_TGACv1_361066_AA1160130 | 116 | 171 | 232 | 6 | 7 | 13 | 4.286132 | 1.94E-17 | up | COG0515^Serine Threonine protein kinase`COG4886^leucine Rich Repeat | KEGG:ath:AT5G46330`KO:K13420 | GO:0005768^cellular_component^endosome`GO:0010008^cellular_component^endosome membrane`GO:0016021^cellular_component^integral component of membrane`GO:0016020^cellular_component^membrane`GO:0005886^cellular_component^plasma membrane`GO:0005524^molecular_function^ATP binding`GO:0004675^molecular_function^transmembrane receptor protein serine/threonine kinase activity`GO:0052544^biological_process^defense response by callose deposition in cell wall`GO:0042742^biological_process^defense response to bacterium`GO:0016045^biological_process^detection of bacterium`GO:0006898^biological_process^receptor-mediated endocytosis`GO:0010359^biological_process^regulation of anion channel activity | FLS2_ARATH^FLS2_ARATH^Q:133-2679,H:30-813^28.46%ID^E:1e-56^RecName: Full=LRR receptor-like serine/threonine-protein kinase FLS2;^Eukaryota; Viridiplantae; Streptophyta; Embryophyta; Tracheophyta; Spermatophyta; Magnoliophyta; eudicotyledons; Gunneridae; Pentapetalae; rosids; malvids; Brassicales; Brassicaceae; Camelineae; Arabidopsis |
| LOC109785536 | TRIAE_CS42_2AL_TGACv1_094670_AA0301260 | 524 | 511 | 476 | 23 | 26 | 38 | 4.093302 | 1.17E-18 | up | COG1028^Dehydrogenase reductase | KEGG:ath:AT3G61220`KO:K15095 | GO:0005737^cellular_component^cytoplasm`GO:0047501^molecular_function^(+)-neomenthol dehydrogenase activity`GO:0006952^biological_process^defense response | SDR1_ARATH^SDR1_ARATH^Q:25-843,H:7-296^47.6%ID^E:8e-76^RecName: Full=(+)-neomenthol dehydrogenase {ECO:0000303\|PubMed:18599651};^Eukaryota; Viridiplantae; Streptophyta; Embryophyta; Tracheophyta; Spermatophyta; Magnoliophyta; eudicotyledons; Gunneridae; Pentapetalae; rosids; malvids; Brassicales; Brassicaceae; Camelineae; Arabidopsis |
| LOC109784788 | TRIAE_CS42_7BL_TGACv1_579395_AA1906910 | 182 | 205 | 171 | 17 | 14 | 18 | 3.481727 | 2.68E-13 | up | COG4886^leucine Rich Repeat | KEGG:ath:AT1G58400 | GO:0005886^cellular_component^plasma membrane`GO:0043531^molecular_function^ADP binding`GO:0005524^molecular_function^ATP binding`GO:0006952^biological_process^defense response`GO:0007165^biological_process^signal transduction | DRL8_ARATH^DRL8_ARATH^Q:22-993,H:1-301^21.1%ID^E:2e-06^RecName: Full=Putative disease resistance protein At1g58400;^Eukaryota; Viridiplantae; Streptophyta; Embryophyta; Tracheophyta; Spermatophyta; Magnoliophyta; eudicotyledons; Gunneridae; Pentapetalae; rosids; malvids; Brassicales; Brassicaceae; Camelineae; Arabidopsis |
| LOC109773063 | TRIAE_CS42_1AL_TGACv1_001423_AA0030310 | 1479 | 1116 | 864 | 65 | 145 | 132 | 3.310337 | 5.67E-14 | up | ENOG410YA3P^NA | . | GO:0006952^biological_process^defense response`GO:0009607^biological_process^response to biotic stimulus | PRPX_HORVU^PRPX_HORVU^Q:1-705,H:1-233^88.98%ID^E:7e-143^RecName: Full=Pathogen-related protein;^Eukaryota; Viridiplantae; Streptophyta; Embryophyta; Tracheophyta; Spermatophyta; Magnoliophyta; Liliopsida; Poales; Poaceae; BOP clade; Pooideae; Triticodae; Triticeae; Hordeinae; Hordeum |
| LOC109774817 | TRIAE_CS42_5AL_TGACv1_374630_AA1204880 | 299 | 296 | 319 | 30 | 43 | 55 | 2.813691 | 4.32E-10 | up | COG2072^Monooxygenase | KEGG:ath:AT1G19250`KO:K00485 | GO:0050660^molecular_function^flavin adenine dinucleotide binding`GO:0004497^molecular_function^monooxygenase activity`GO:0004499^molecular_function^N,N-dimethylaniline monooxygenase activity`GO:0050661^molecular_function^NADP binding`GO:0071456^biological_process^cellular response to hypoxia`GO:0009870^biological_process^defense response signaling pathway, resistance gene-dependent`GO:0010204^biological_process^defense response signaling pathway, resistance gene-independent`GO:0042742^biological_process^defense response to bacterium`GO:0050832^biological_process^defense response to fungus`GO:0055114^biological_process^oxidation-reduction process`GO:0009626^biological_process^plant-type hypersensitive response`GO:0051707^biological_process^response to other organism`GO:0009627^biological_process^systemic acquired resistance | FMO1_ARATH^FMO1_ARATH^Q:82-1548,H:33-518^36.95%ID^E:3e-107^RecName: Full=Probable flavin-containing monooxygenase 1;^Eukaryota; Viridiplantae; Streptophyta; Embryophyta; Tracheophyta; Spermatophyta; Magnoliophyta; eudicotyledons; Gunneridae; Pentapetalae; rosids; malvids; Brassicales; Brassicaceae; Camelineae; Arabidopsis |
| LOC109731924 | TRIAE_CS42_3B_TGACv1_224883_AA0802750 | 1322 | 1335 | 908 | 140 | 193 | 219 | 2.667881 | 8.78E-10 | up | ENOG410YZDC^Cysteine proteinase inhibitor | KEGG:osa:4327535 | GO:0005576^cellular_component^extracellular region`GO:0004869^molecular_function^cysteine-type endopeptidase inhibitor activity`GO:0002020^molecular_function^protease binding`GO:0006952^biological_process^defense response`GO:2000117^biological_process^negative regulation of cysteine-type endopeptidase activity | CYT1_ORYSJ^CYT1_ORYSJ^Q:7-351,H:1-113^38.14%ID^E:4e-12^RecName: Full=Cysteine proteinase inhibitor 1;^Eukaryota; Viridiplantae; Streptophyta; Embryophyta; Tracheophyta; Spermatophyta; Magnoliophyta; Liliopsida; Poales; Poaceae; BOP clade; Oryzoideae; Oryzeae; Oryzinae; Oryza; Oryza sativa |
| LOC109747254 | TRIAE_CS42_1AL_TGACv1_001501_AA0031460 | 153 | 240 | 242 | 39 | 24 | 38 | 2.636283 | 8.40E-09 | up | ENOG410YVV0^VQ motif-containing protein | KEGG:ath:AT1G28280 | GO:0005634^cellular_component^nucleus`GO:0006952^biological_process^defense response`GO:0051245^biological_process^negative regulation of cellular defense response`GO:0043433^biological_process^negative regulation of sequence-specific DNA binding transcription factor activity | VQ4_ARATH^VQ4_ARATH^Q:169-690,H:53-238^48.39%ID^E:3e-31^RecName: Full=VQ motif-containing protein 4 {ECO:0000303\|PubMed:22535423};^Eukaryota; Viridiplantae; Streptophyta; Embryophyta; Tracheophyta; Spermatophyta; Magnoliophyta; eudicotyledons; Gunneridae; Pentapetalae; rosids; malvids; Brassicales; Brassicaceae; Camelineae; Arabidopsis |
| LOC109752722 | TRIAE_CS42_7BL_TGACv1_577676_AA1880730 | 24 | 49 | 35 | 5 | 9 | 8 | 2.258069 | 6.06E-05 | up | COG0515^Serine Threonine protein kinase`COG4886^leucine Rich Repeat | KEGG:osa:107276510`KO:K13437 | GO:0032541^cellular_component^cortical endoplasmic reticulum`GO:0005789^cellular_component^endoplasmic reticulum membrane`GO:0016021^cellular_component^integral component of membrane`GO:0005634^cellular_component^nucleus`GO:1990578^cellular_component^perinuclear endoplasmic reticulum membrane`GO:0005886^cellular_component^plasma membrane`GO:0005524^molecular_function^ATP binding`GO:0004674^molecular_function^protein serine/threonine kinase activity`GO:0006952^biological_process^defense response | XA21_ORYSJ^XA21_ORYSJ^Q:103-1938,H:41-678^33.23%ID^E:2e-50^RecName: Full=Receptor kinase-like protein Xa21 {ECO:0000303\|PubMed:22735448};^Eukaryota; Viridiplantae; Streptophyta; Embryophyta; Tracheophyta; Spermatophyta; Magnoliophyta; Liliopsida; Poales; Poaceae; BOP clade; Oryzoideae; Oryzeae; Oryzinae; Oryza; Oryza sativa |
| LOC109751111 | TRIAE_CS42_3DL_TGACv1_251757_AA0884610 | 601 | 469 | 466 | 113 | 84 | 138 | 2.177496 | 8.99E-07 | up | ENOG410YA4N^glucan endo-1-3-beta-glucosidase | KEGG:ath:AT2G27500 | GO:0046658^cellular_component^anchored component of plasma membrane`GO:0005618^cellular_component^cell wall`GO:0005737^cellular_component^cytoplasm`GO:0005576^cellular_component^extracellular region`GO:0005634^cellular_component^nucleus`GO:0042973^molecular_function^glucan endo-1,3-beta-D-glucosidase activity`GO:0004553^molecular_function^hydrolase activity, hydrolyzing O-glycosyl compounds`GO:0030247^molecular_function^polysaccharide binding`GO:0005975^biological_process^carbohydrate metabolic process`GO:0071555^biological_process^cell wall organization`GO:0006952^biological_process^defense response | E1314_ARATH^E1314_ARATH^Q:97-1050,H:27-346^63.75%ID^E:1e-150^RecName: Full=Glucan endo-1,3-beta-glucosidase 14;^Eukaryota; Viridiplantae; Streptophyta; Embryophyta; Tracheophyta; Spermatophyta; Magnoliophyta; eudicotyledons; Gunneridae; Pentapetalae; rosids; malvids; Brassicales; Brassicaceae; Camelineae; Arabidopsis |
| LOC109779702 | TRIAE_CS42_6AL_TGACv1_472950_AA1527420 | 97 | 95 | 135 | 17 | 31 | 26 | 2.120816 | 9.54E-06 | up | ENOG41121N2^zinc ion binding | KEGG:ath:AT1G20823 | GO:0016021^cellular_component^integral component of membrane`GO:0005886^cellular_component^plasma membrane`GO:0046872^molecular_function^metal ion binding`GO:0061630^molecular_function^ubiquitin protein ligase activity`GO:0070417^biological_process^cellular response to cold`GO:0006952^biological_process^defense response`GO:0043161^biological_process^proteasome-mediated ubiquitin-dependent protein catabolic process`GO:0009909^biological_process^regulation of flower development`GO:0010966^biological_process^regulation of phosphate transport`GO:0010200^biological_process^response to chitin | ATL80_ARATH^ATL80_ARATH^Q:202-420,H:85-157^57.53%ID^E:1e-19^RecName: Full=RING-H2 finger protein ATL80;^Eukaryota; Viridiplantae; Streptophyta; Embryophyta; Tracheophyta; Spermatophyta; Magnoliophyta; eudicotyledons; Gunneridae; Pentapetalae; rosids; malvids; Brassicales; Brassicaceae; Camelineae; Arabidopsis |
| LOC109778386 | TRIAE_CS42_1AL_TGACv1_002051_AA0038150 | 359 | 572 | 433 | 81 | 112 | 149 | 1.976646 | 9.8E-06 | up | ENOG410YQZM^WRKY transcription factor | KEGG:ath:AT5G64810 | GO:0005634^cellular_component^nucleus`GO:0043565^molecular_function^sequence-specific DNA binding`GO:0003700^molecular_function^transcription factor activity, sequence-specific DNA binding`GO:0042742^biological_process^defense response to bacterium`GO:0050832^biological_process^defense response to fungus`GO:0009867^biological_process^jasmonic acid mediated signaling pathway`GO:0006351^biological_process^transcription, DNA-templated | WRK51_ARATH^WRK51_ARATH^Q:358-591,H:97-174^75.64%ID^E:3e-37^RecName: Full=Probable WRKY transcription factor 51;^Eukaryota; Viridiplantae; Streptophyta; Embryophyta; Tracheophyta; Spermatophyta; Magnoliophyta; eudicotyledons; Gunneridae; Pentapetalae; rosids; malvids; Brassicales; Brassicaceae; Camelineae; Arabidopsis |
| LOC109743072 | TRIAE_CS42_5BL_TGACv1_405693_AA1333240 | 272 | 242 | 173 | 40 | 48 | 84 | 1.973106 | 1.62E-05 | up | ENOG410YIDD^Harpin-induced protein 1 containing protein, expressed | KEGG:ath:AT2G27080 | GO:0016021^cellular_component^integral component of membrane`GO:0005886^cellular_component^plasma membrane`GO:0009506^cellular_component^plasmodesma`GO:0042742^biological_process^defense response to bacterium | NHL13_ARATH^NHL13_ARATH^Q:163-840,H:38-260^33.48%ID^E:1e-33^RecName: Full=NDR1/HIN1-like protein 13 {ECO:0000303\|PubMed:26206852};^Eukaryota; Viridiplantae; Streptophyta; Embryophyta; Tracheophyta; Spermatophyta; Magnoliophyta; eudicotyledons; Gunneridae; Pentapetalae; rosids; malvids; Brassicales; Brassicaceae; Camelineae; Arabidopsis |
| LOC109765296 | TRIAE_CS42_U_TGACv1_644623_AA2140860 | 143 | 135 | 315 | 38 | 83 | 30 | 1.9568 | 2.20E-05 | up | ENOG410YDA6^synthase | KEGG:osa:4328124`KO:K15086 | GO:0009507^cellular_component^chloroplast`GO:0000287^molecular_function^magnesium ion binding`GO:0034007^molecular_function^S-linalool synthase activity`GO:0010333^molecular_function^terpene synthase activity`GO:0042742^biological_process^defense response to bacterium`GO:0043693^biological_process^monoterpene biosynthetic process`GO:0016114^biological_process^terpenoid biosynthetic process | LINS_ORYSJ^LINS_ORYSJ^Q:112-1650,H:51-595^64.04%ID^E:0^RecName: Full=S-(+)-linalool synthase, chloroplastic {ECO:0000305};^Eukaryota; Viridiplantae; Streptophyta; Embryophyta; Tracheophyta; Spermatophyta; Magnoliophyta; Liliopsida; Poales; Poaceae; BOP clade; Oryzoideae; Oryzeae; Oryzinae; Oryza; Oryza sativa |
| LOC109774385 | TRIAE_CS42_5DS_TGACv1_456665_AA1475820 | 126 | 114 | 100 | 18 | 41 | 31 | 1.889954 | 8.24E-05 | up | COG4886^leucine Rich Repeat | KEGG:ath:AT5G43470 | GO:0005886^cellular_component^plasma membrane`GO:0043531^molecular_function^ADP binding`GO:0005524^molecular_function^ATP binding`GO:0000166^molecular_function^nucleotide binding`GO:0071446^biological_process^cellular response to salicylic acid stimulus`GO:0006952^biological_process^defense response`GO:0051607^biological_process^defense response to virus`GO:0009626^biological_process^plant-type hypersensitive response`GO:0002230^biological_process^positive regulation of defense response to virus by host`GO:0009646^biological_process^response to absence of light`GO:0009637^biological_process^response to blue light`GO:0009416^biological_process^response to light stimulus`GO:0002239^biological_process^response to oomycetes`GO:0051707^biological_process^response to other organism`GO:0009751^biological_process^response to salicylic acid`GO:0009611^biological_process^response to wounding`GO:0007165^biological_process^signal transduction | RPP8_ARATH^RPP8_ARATH^Q:43-2928,H:16-880^25.6%ID^E:2e-56^RecName: Full=Disease resistance protein RPP8;^Eukaryota; Viridiplantae; Streptophyta; Embryophyta; Tracheophyta; Spermatophyta; Magnoliophyta; eudicotyledons; Gunneridae; Pentapetalae; rosids; malvids; Brassicales; Brassicaceae; Camelineae; Arabidopsis |
| LOC109756490 | TRIAE_CS42_U_TGACv1_640941_AA2080000 | 49 | 47 | 57 | 12 | 13 | 16 | 1.873304 | 0.000384 | up | COG4886^leucine Rich Repeat | KEGG:ath:AT5G43470 | GO:0005886^cellular_component^plasma membrane`GO:0043531^molecular_function^ADP binding`GO:0005524^molecular_function^ATP binding`GO:0000166^molecular_function^nucleotide binding`GO:0071446^biological_process^cellular response to salicylic acid stimulus`GO:0006952^biological_process^defense response`GO:0051607^biological_process^defense response to virus`GO:0009626^biological_process^plant-type hypersensitive response`GO:0002230^biological_process^positive regulation of defense response to virus by host`GO:0009646^biological_process^response to absence of light`GO:0009637^biological_process^response to blue light`GO:0009416^biological_process^response to light stimulus`GO:0002239^biological_process^response to oomycetes`GO:0051707^biological_process^response to other organism`GO:0009751^biological_process^response to salicylic acid`GO:0009611^biological_process^response to wounding`GO:0007165^biological_process^signal transduction | RPP8_ARATH^RPP8_ARATH^Q:1-1920,H:1-620^30.34%ID^E:1e-59^RecName: Full=Disease resistance protein RPP8;^Eukaryota; Viridiplantae; Streptophyta; Embryophyta; Tracheophyta; Spermatophyta; Magnoliophyta; eudicotyledons; Gunneridae; Pentapetalae; rosids; malvids; Brassicales; Brassicaceae; Camelineae; Arabidopsis |
| LOC109757924 | TRIAE_CS42_3AL_TGACv1_193668_AA0617190 | 25 | 37 | 16 | 8 | 4 | 9 | 1.855068 | 0.002165 | up | ENOG41123N5^Transcription factor | KEGG:ath:AT1G28360 | GO:0005634^cellular_component^nucleus`GO:0003677^molecular_function^DNA binding`GO:0003700^molecular_function^transcription factor activity, sequence-specific DNA binding`GO:0006952^biological_process^defense response`GO:0009873^biological_process^ethylene-activated signaling pathway`GO:0006351^biological_process^transcription, DNA-templated | ERF81_ARATH^ERF81_ARATH^Q:88-264,H:9-67^91.53%ID^E:1e-29^RecName: Full=Ethylene-responsive transcription factor 12;^Eukaryota; Viridiplantae; Streptophyta; Embryophyta; Tracheophyta; Spermatophyta; Magnoliophyta; eudicotyledons; Gunneridae; Pentapetalae; rosids; malvids; Brassicales; Brassicaceae; Camelineae; Arabidopsis |
| LOC109734994 | TRIAE_CS42_5DS_TGACv1_456473_AA1471510 | 114 | 125 | 104 | 19 | 47 | 28 | 1.840773 | 0.000128 | up | ENOG410Z9YD^VQ motif | KEGG:ath:AT3G18360 | GO:0005634^cellular_component^nucleus`GO:0006952^biological_process^defense response | VQ20_ARATH^VQ20_ARATH^Q:79-330,H:46-114^39.29%ID^E:7e-08^RecName: Full=VQ motif-containing protein 20 {ECO:0000303\|PubMed:22535423};^Eukaryota; Viridiplantae; Streptophyta; Embryophyta; Tracheophyta; Spermatophyta; Magnoliophyta; eudicotyledons; Gunneridae; Pentapetalae; rosids; malvids; Brassicales; Brassicaceae; Camelineae; Arabidopsis |
| LOC109753134 | TRIAE_CS42_4AS_TGACv1_308110_AA1025950 | 1769 | 2063 | 2323 | 343 | 734 | 693 | 1.778519 | 6.11E-05 | up | COG3621^Patatin group | . | GO:0016787^molecular_function^hydrolase activity`GO:0006952^biological_process^defense response`GO:0016042^biological_process^lipid catabolic process | PLP1_ORYSI^PLP1_ORYSI^Q:139-1281,H:14-396^57.96%ID^E:4e-159^RecName: Full=Patatin-like protein 1;^Eukaryota; Viridiplantae; Streptophyta; Embryophyta; Tracheophyta; Spermatophyta; Magnoliophyta; Liliopsida; Poales; Poaceae; BOP clade; Oryzoideae; Oryzeae; Oryzinae; Oryza; Oryza sativa |
| LOC109749828 | TRIAE_CS42_3AL_TGACv1_195024_AA0643600 | 150 | 66 | 75 | 34 | 26 | 24 | 1.765967 | 0.000309 | up | ENOG410XQVB^DiacylGlycerol Kinase | KEGG:ath:AT2G20900`KO:K00901 | GO:0005524^molecular_function^ATP binding`GO:0004143^molecular_function^diacylglycerol kinase activity`GO:0003951^molecular_function^NAD+ kinase activity`GO:0006952^biological_process^defense response`GO:0007205^biological_process^protein kinase C-activating G-protein coupled receptor signaling pathway | DGK5_ARATH^DGK5_ARATH^Q:1-1434,H:1-480^64.67%ID^E:0^RecName: Full=Diacylglycerol kinase 5;^Eukaryota; Viridiplantae; Streptophyta; Embryophyta; Tracheophyta; Spermatophyta; Magnoliophyta; eudicotyledons; Gunneridae; Pentapetalae; rosids; malvids; Brassicales; Brassicaceae; Camelineae; Arabidopsis |
| LOC109766294 | TRIAE_CS42_2BL_TGACv1_132242_AA0436300 | 1339 | 2011 | 2330 | 511 | 542 | 632 | 1.739995 | 9.23E-05 | up | . | . | GO:0005789^cellular_component^endoplasmic reticulum membrane`GO:0016021^cellular_component^integral component of membrane`GO:0005634^cellular_component^nucleus`GO:0005886^cellular_component^plasma membrane`GO:0005524^molecular_function^ATP binding`GO:0004674^molecular_function^protein serine/threonine kinase activity`GO:0006952^biological_process^defense response`GO:0031349^biological_process^positive regulation of defense response`GO:1900426^biological_process^positive regulation of defense response to bacterium`GO:0046777^biological_process^protein autophosphorylation | XA21_ORYSI^XA21_ORYSI^Q:118-2946,H:35-1007^39.41%ID^E:9e-168^RecName: Full=Receptor kinase-like protein Xa21 {ECO:0000303\|Ref.1};^Eukaryota; Viridiplantae; Streptophyta; Embryophyta; Tracheophyta; Spermatophyta; Magnoliophyta; Liliopsida; Poales; Poaceae; BOP clade; Oryzoideae; Oryzeae; Oryzinae; Oryza; Oryza sativa |
| LOC109749583 | TRIAE_CS42_3B_TGACv1_223056_AA0775790 | 133 | 147 | 208 | 62 | 46 | 39 | 1.717363 | 0.000274 | up | COG4886^leucine Rich Repeat | KEGG:ath:AT4G26090`KO:K13459 | GO:0005737^cellular_component^cytoplasm`GO:0005886^cellular_component^plasma membrane`GO:0043531^molecular_function^ADP binding`GO:0005524^molecular_function^ATP binding`GO:0006952^biological_process^defense response`GO:0042742^biological_process^defense response to bacterium`GO:0016045^biological_process^detection of bacterium`GO:0009626^biological_process^plant-type hypersensitive response`GO:0007165^biological_process^signal transduction | RPS2_ARATH^RPS2_ARATH^Q:1-2592,H:1-904^34.98%ID^E:3e-133^RecName: Full=Disease resistance protein RPS2;^Eukaryota; Viridiplantae; Streptophyta; Embryophyta; Tracheophyta; Spermatophyta; Magnoliophyta; eudicotyledons; Gunneridae; Pentapetalae; rosids; malvids; Brassicales; Brassicaceae; Camelineae; Arabidopsis |
| LOC109782505 | TRIAE_CS42_1BL_TGACv1_034148_AA0143620 | 233 | 220 | 125 | 52 | 69 | 56 | 1.681478 | 0.000336 | up | ENOG410YZZY^Transcription factor | KEGG:ath:AT3G56400 | GO:0005634^cellular_component^nucleus`GO:0043565^molecular_function^sequence-specific DNA binding`GO:0003700^molecular_function^transcription factor activity, sequence-specific DNA binding`GO:0042742^biological_process^defense response to bacterium`GO:0050832^biological_process^defense response to fungus`GO:0009864^biological_process^induced systemic resistance, jasmonic acid mediated signaling pathway`GO:1900056^biological_process^negative regulation of leaf senescence`GO:0045892^biological_process^negative regulation of transcription, DNA-templated`GO:0031347^biological_process^regulation of defense response`GO:0010200^biological_process^response to chitin`GO:0009753^biological_process^response to jasmonic acid`GO:0009751^biological_process^response to salicylic acid`GO:0009862^biological_process^systemic acquired resistance, salicylic acid mediated signaling pathway`GO:0006351^biological_process^transcription, DNA-templated | WRK70_ARATH^WRK70_ARATH^Q:133-450,H:75-179^45.37%ID^E:2e-24^RecName: Full=Probable WRKY transcription factor 70;^Eukaryota; Viridiplantae; Streptophyta; Embryophyta; Tracheophyta; Spermatophyta; Magnoliophyta; eudicotyledons; Gunneridae; Pentapetalae; rosids; malvids; Brassicales; Brassicaceae; Camelineae; Arabidopsis |
| AA1714750 | TRIAE_CS42_6DL_TGACv1_528520_AA1714750 | 70 | 88 | 56 | 14 | 25 | 27 | 1.669756 | 0.001026 | up | ENOG41121N2^zinc ion binding | KEGG:ath:AT1G20823 | GO:0016021^cellular_component^integral component of membrane`GO:0005886^cellular_component^plasma membrane`GO:0046872^molecular_function^metal ion binding`GO:0061630^molecular_function^ubiquitin protein ligase activity`GO:0070417^biological_process^cellular response to cold`GO:0006952^biological_process^defense response`GO:0043161^biological_process^proteasome-mediated ubiquitin-dependent protein catabolic process`GO:0009909^biological_process^regulation of flower development`GO:0010966^biological_process^regulation of phosphate transport`GO:0010200^biological_process^response to chitin | ATL80_ARATH^ATL80_ARATH^Q:193-420,H:82-157^57.89%ID^E:9e-22^RecName: Full=RING-H2 finger protein ATL80;^Eukaryota; Viridiplantae; Streptophyta; Embryophyta; Tracheophyta; Spermatophyta; Magnoliophyta; eudicotyledons; Gunneridae; Pentapetalae; rosids; malvids; Brassicales; Brassicaceae; Camelineae; Arabidopsis |
| LOC109785629 | TRIAE_CS42_2AS_TGACv1_112531_AA0340030 | 389 | 271 | 236 | 72 | 83 | 135 | 1.603317 | 0.000543 | up | COG2072^Monooxygenase | KEGG:ath:AT1G19250`KO:K00485 | GO:0050660^molecular_function^flavin adenine dinucleotide binding`GO:0004497^molecular_function^monooxygenase activity`GO:0004499^molecular_function^N,N-dimethylaniline monooxygenase activity`GO:0050661^molecular_function^NADP binding`GO:0071456^biological_process^cellular response to hypoxia`GO:0009870^biological_process^defense response signaling pathway, resistance gene-dependent`GO:0010204^biological_process^defense response signaling pathway, resistance gene-independent`GO:0042742^biological_process^defense response to bacterium`GO:0050832^biological_process^defense response to fungus`GO:0055114^biological_process^oxidation-reduction process`GO:0009626^biological_process^plant-type hypersensitive response`GO:0051707^biological_process^response to other organism`GO:0009627^biological_process^systemic acquired resistance | FMO1_ARATH^FMO1_ARATH^Q:1-1539,H:8-518^38.85%ID^E:4e-120^RecName: Full=Probable flavin-containing monooxygenase 1;^Eukaryota; Viridiplantae; Streptophyta; Embryophyta; Tracheophyta; Spermatophyta; Magnoliophyta; eudicotyledons; Gunneridae; Pentapetalae; rosids; malvids; Brassicales; Brassicaceae; Camelineae; Arabidopsis |
| LOC109760424 | TRIAE_CS42_3DL_TGACv1_249034_AA0835450 | 19 | 51 | 35 | 15 | 11 | 8 | 1.602861 | 0.005068 | up | ENOG41114CW^WRKY | KEGG:ath:AT5G52830 | GO:0005634^cellular_component^nucleus`GO:0043565^molecular_function^sequence-specific DNA binding`GO:0003700^molecular_function^transcription factor activity, sequence-specific DNA binding`GO:0044212^molecular_function^transcription regulatory region DNA binding`GO:0042742^biological_process^defense response to bacterium`GO:0045892^biological_process^negative regulation of transcription, DNA-templated`GO:0007263^biological_process^nitric oxide mediated signal transduction`GO:0009739^biological_process^response to gibberellin`GO:0006351^biological_process^transcription, DNA-templated | WRK27_ARATH^WRK27_ARATH^Q:577-900,H:150-254^57.41%ID^E:2e-33^RecName: Full=Probable WRKY transcription factor 27;^Eukaryota; Viridiplantae; Streptophyta; Embryophyta; Tracheophyta; Spermatophyta; Magnoliophyta; eudicotyledons; Gunneridae; Pentapetalae; rosids; malvids; Brassicales; Brassicaceae; Camelineae; Arabidopsis |
| LOC100846489 | TRIAE_CS42_3AL_TGACv1_194206_AA0628470 | 30 | 30 | 39 | 10 | 11 | 11 | 1.601756 | 0.005692 | up | . | . | GO:0043531^molecular_function^ADP binding`GO:0005524^molecular_function^ATP binding`GO:0006952^biological_process^defense response | RGA4_SOLBU^RGA4_SOLBU^Q:202-3498,H:4-987^33.78%ID^E:4e-171^RecName: Full=Putative disease resistance protein RGA4;^Eukaryota; Viridiplantae; Streptophyta; Embryophyta; Tracheophyta; Spermatophyta; Magnoliophyta; eudicotyledons; Gunneridae; Pentapetalae; asterids; lamiids; Solanales; Solanaceae; Solanoideae; Solaneae; Solanum |
| LOC109731409 | TRIAE_CS42_5DL_TGACv1_433458_AA1413630 | 1863 | 2674 | 2533 | 427 | 708 | 1173 | 1.597118 | 3.90E-04 | up | COG5147^Myblike DNAbinding domain containing protein | KEGG:ath:AT5G67300`KO:K09422 | GO:0005634^cellular_component^nucleus`GO:0000981^molecular_function^RNA polymerase II transcription factor activity, sequence-specific DNA binding`GO:0043565^molecular_function^sequence-specific DNA binding`GO:0001135^molecular_function^transcription factor activity, RNA polymerase II transcription factor recruiting`GO:0003700^molecular_function^transcription factor activity, sequence-specific DNA binding`GO:0044212^molecular_function^transcription regulatory region DNA binding`GO:0009738^biological_process^abscisic acid-activated signaling pathway`GO:0030154^biological_process^cell differentiation`GO:0042742^biological_process^defense response to bacterium`GO:0050832^biological_process^defense response to fungus`GO:2000022^biological_process^regulation of jasmonic acid mediated signaling pathway`GO:2000031^biological_process^regulation of salicylic acid mediated signaling pathway`GO:0006357^biological_process^regulation of transcription from RNA polymerase II promoter`GO:0009737^biological_process^response to abscisic acid`GO:0009733^biological_process^response to auxin`GO:0046686^biological_process^response to cadmium ion`GO:0010200^biological_process^response to chitin`GO:0009723^biological_process^response to ethylene`GO:0009739^biological_process^response to gibberellin`GO:0009753^biological_process^response to jasmonic acid`GO:0009751^biological_process^response to salicylic acid`GO:0009651^biological_process^response to salt stress`GO:0009414^biological_process^response to water deprivation`GO:0006351^biological_process^transcription, DNA-templated | MYB44_ARATH^MYB44_ARATH^Q:46-357,H:3-106^83.65%ID^E:3e-57^RecName: Full=Transcription factor MYB44;^Eukaryota; Viridiplantae; Streptophyta; Embryophyta; Tracheophyta; Spermatophyta; Magnoliophyta; eudicotyledons; Gunneridae; Pentapetalae; rosids; malvids; Brassicales; Brassicaceae; Camelineae; Arabidopsis |
| *WRKY9* | TRIAE_CS42_2DL_TGACv1_158128_AA0509990 | 1295 | 1232 | 1270 | 333 | 374 | 532 | 1.59706 | 4.11E-04 | up | ENOG410YD1E^WRKY transcription factor | KEGG:ath:AT4G31550 | GO:0005634^cellular_component^nucleus`GO:0005516^molecular_function^calmodulin binding`GO:0043565^molecular_function^sequence-specific DNA binding`GO:0003700^molecular_function^transcription factor activity, sequence-specific DNA binding`GO:0042742^biological_process^defense response to bacterium`GO:0010200^biological_process^response to chitin`GO:0006351^biological_process^transcription, DNA-templated | WRK11_ARATH^WRK11_ARATH^Q:1-873,H:1-304^50.62%ID^E:8e-68^RecName: Full=Probable WRKY transcription factor 11;^Eukaryota; Viridiplantae; Streptophyta; Embryophyta; Tracheophyta; Spermatophyta; Magnoliophyta; eudicotyledons; Gunneridae; Pentapetalae; rosids; malvids; Brassicales; Brassicaceae; Camelineae; Arabidopsis |
| LOC109759870 | TRIAE_CS42_5BS_TGACv1_424818_AA1391910 | 124 | 130 | 125 | 35 | 53 | 36 | 1.589537 | 0.001043 | up | ENOG410YD1E^WRKY transcription factor | KEGG:ath:AT4G31550 | GO:0005634^cellular_component^nucleus`GO:0005516^molecular_function^calmodulin binding`GO:0043565^molecular_function^sequence-specific DNA binding`GO:0003700^molecular_function^transcription factor activity, sequence-specific DNA binding`GO:0042742^biological_process^defense response to bacterium`GO:0010200^biological_process^response to chitin`GO:0006351^biological_process^transcription, DNA-templated | WRK11_ARATH^WRK11_ARATH^Q:88-870,H:24-304^43.55%ID^E:1e-53^RecName: Full=Probable WRKY transcription factor 11;^Eukaryota; Viridiplantae; Streptophyta; Embryophyta; Tracheophyta; Spermatophyta; Magnoliophyta; eudicotyledons; Gunneridae; Pentapetalae; rosids; malvids; Brassicales; Brassicaceae; Camelineae; Arabidopsis |
| *WRKY51* | TRIAE_CS42_1DL_TGACv1_061726_AA0202510 | 565 | 760 | 621 | 144 | 288 | 231 | 1.532224 | 0.000838 | up | ENOG410YQZM^WRKY transcription factor | KEGG:ath:AT5G64810 | GO:0005634^cellular_component^nucleus`GO:0043565^molecular_function^sequence-specific DNA binding`GO:0003700^molecular_function^transcription factor activity, sequence-specific DNA binding`GO:0042742^biological_process^defense response to bacterium`GO:0050832^biological_process^defense response to fungus`GO:0009867^biological_process^jasmonic acid mediated signaling pathway`GO:0006351^biological_process^transcription, DNA-templated | WRK51_ARATH^WRK51_ARATH^Q:358-591,H:97-174^75.64%ID^E:7e-38^RecName: Full=Probable WRKY transcription factor 51;^Eukaryota; Viridiplantae; Streptophyta; Embryophyta; Tracheophyta; Spermatophyta; Magnoliophyta; eudicotyledons; Gunneridae; Pentapetalae; rosids; malvids; Brassicales; Brassicaceae; Camelineae; Arabidopsis |
| *MAPK* | TRIAE_CS42_4DL_TGACv1_344691_AA1148960 | 1029 | 1345 | 1719 | 268 | 586 | 580 | 1.495671 | 0.001063 | up | ENOG410XNY0^Mitogen-activated protein kinase | KEGG:osa:4332475`KO:K20536 | GO:0005737^cellular_component^cytoplasm`GO:0005634^cellular_component^nucleus`GO:0005524^molecular_function^ATP binding`GO:0004707^molecular_function^MAP kinase activity`GO:0000169^biological_process^activation of MAPK activity involved in osmosensory signaling pathway`GO:0010120^biological_process^camalexin biosynthetic process`GO:0042742^biological_process^defense response to bacterium`GO:0050832^biological_process^defense response to fungus`GO:0010229^biological_process^inflorescence development`GO:0048481^biological_process^plant ovule development`GO:0009555^biological_process^pollen development`GO:0010183^biological_process^pollen tube guidance`GO:1901002^biological_process^positive regulation of response to salt stress`GO:0080136^biological_process^priming of cellular response to stress`GO:0010468^biological_process^regulation of gene expression`GO:0010200^biological_process^response to chitin`GO:0009409^biological_process^response to cold`GO:0006970^biological_process^response to osmotic stress`GO:0006979^biological_process^response to oxidative stress`GO:0010224^biological_process^response to UV-B`GO:0009414^biological_process^response to water deprivation`GO:0009611^biological_process^response to wounding | MPK5_ORYSJ^MPK5_ORYSJ^Q:1-1107,H:1-369^91.33%ID^E:0^RecName: Full=Mitogen-activated protein kinase 5;^Eukaryota; Viridiplantae; Streptophyta; Embryophyta; Tracheophyta; Spermatophyta; Magnoliophyta; Liliopsida; Poales; Poaceae; BOP clade; Oryzoideae; Oryzeae; Oryzinae; Oryza; Oryza sativa |
| *WRKY68-a* | TRIAE_CS42_2AL_TGACv1_093177_AA0274160 | 1252 | 1305 | 1359 | 388 | 460 | 526 | 1.49315 | 0.001091 | up | ENOG410YD1E^WRKY transcription factor | KEGG:ath:AT4G31550 | GO:0005634^cellular_component^nucleus`GO:0005516^molecular_function^calmodulin binding`GO:0043565^molecular_function^sequence-specific DNA binding`GO:0003700^molecular_function^transcription factor activity, sequence-specific DNA binding`GO:0042742^biological_process^defense response to bacterium`GO:0010200^biological_process^response to chitin`GO:0006351^biological_process^transcription, DNA-templated | WRK11_ARATH^WRK11_ARATH^Q:1-243,H:1-73^51.85%ID^E:2e-08^RecName: Full=Probable WRKY transcription factor 11;^Eukaryota; Viridiplantae; Streptophyta; Embryophyta; Tracheophyta; Spermatophyta; Magnoliophyta; eudicotyledons; Gunneridae; Pentapetalae; rosids; malvids; Brassicales; Brassicaceae; Camelineae; Arabidopsis |
| LOC109783404 | TRIAE_CS42_5DL_TGACv1_434125_AA1429890 | 414 | 534 | 732 | 173 | 250 | 176 | 1.472967 | 0.001473 | up | COG0631^Phosphatase | KEGG:osa:4334600 | GO:0005886^cellular_component^plasma membrane`GO:0046872^molecular_function^metal ion binding`GO:0004722^molecular_function^protein serine/threonine phosphatase activity`GO:0006952^biological_process^defense response | P2C35_ORYSJ^P2C35_ORYSJ^Q:4-1374,H:173-639^84.75%ID^E:0^RecName: Full=Protein phosphatase 2C 35;^Eukaryota; Viridiplantae; Streptophyta; Embryophyta; Tracheophyta; Spermatophyta; Magnoliophyta; Liliopsida; Poales; Poaceae; BOP clade; Oryzoideae; Oryzeae; Oryzinae; Oryza; Oryza sativa |
| LOC109734059 | TRIAE_CS42_7DL_TGACv1_603776_AA1989080 | 800 | 1247 | 1606 | 301 | 454 | 553 | 1.467627 | 0.001389 | up | COG4886^leucine Rich Repeat | KEGG:ath:AT1G50180 | GO:0005886^cellular_component^plasma membrane`GO:0009506^cellular_component^plasmodesma`GO:0043531^molecular_function^ADP binding`GO:0005524^molecular_function^ATP binding`GO:0006952^biological_process^defense response`GO:0007165^biological_process^signal transduction | DRL4_ARATH^DRL4_ARATH^Q:94-1878,H:30-625^27.79%ID^E:2e-62^RecName: Full=Putative disease resistance protein At1g50180;^Eukaryota; Viridiplantae; Streptophyta; Embryophyta; Tracheophyta; Spermatophyta; Magnoliophyta; eudicotyledons; Gunneridae; Pentapetalae; rosids; malvids; Brassicales; Brassicaceae; Camelineae; Arabidopsis |
| LOC109776117 | TRIAE_CS42_3AL_TGACv1_197538_AA0666650 | 197 | 246 | 307 | 94 | 103 | 79 | 1.427313 | 0.002729 | up | COG0515^Serine Threonine protein kinase | KEGG:osa:4343500 | GO:0016021^cellular_component^integral component of membrane`GO:0005886^cellular_component^plasma membrane`GO:0005524^molecular_function^ATP binding`GO:0004672^molecular_function^protein kinase activity`GO:0004674^molecular_function^protein serine/threonine kinase activity`GO:0042742^biological_process^defense response to bacterium | CRK6_ORYSJ^CRK6_ORYSJ^Q:112-2019,H:38-691^41.77%ID^E:2e-158^RecName: Full=Cysteine-rich receptor-like protein kinase 6 {ECO:0000305};^Eukaryota; Viridiplantae; Streptophyta; Embryophyta; Tracheophyta; Spermatophyta; Magnoliophyta; Liliopsida; Poales; Poaceae; BOP clade; Oryzoideae; Oryzeae; Oryzinae; Oryza; Oryza sativa |
| LOC109783813 | TRIAE_CS42_4DL_TGACv1_342886_AA1124700 | 2018 | 2348 | 3130 | 672 | 952 | 1194 | 1.395935 | 0.00252 | up | ENOG410ZUVB^May be involved in modulation of pathogen defense and leaf cell death (By similarity) | . | GO:0016021^cellular_component^integral component of membrane`GO:0005516^molecular_function^calmodulin binding`GO:0006952^biological_process^defense response`GO:0009607^biological_process^response to biotic stimulus | MLO_HORVU^MLO_HORVU^Q:10-1278,H:3-422^82.82%ID^E:0^RecName: Full=Protein MLO;^Eukaryota; Viridiplantae; Streptophyta; Embryophyta; Tracheophyta; Spermatophyta; Magnoliophyta; Liliopsida; Poales; Poaceae; BOP clade; Pooideae; Triticodae; Triticeae; Hordeinae; Hordeum |
| LOC109753144 | TRIAE_CS42_1AL_TGACv1_004538_AA0053790 | 97 | 94 | 144 | 24 | 39 | 63 | 1.391859 | 0.005489 | up | COG0515^Serine Threonine protein kinase | KEGG:ath:AT4G23180 | GO:0016021^cellular_component^integral component of membrane`GO:0005886^cellular_component^plasma membrane`GO:0005524^molecular_function^ATP binding`GO:0004674^molecular_function^protein serine/threonine kinase activity`GO:0042742^biological_process^defense response to bacterium`GO:0006468^biological_process^protein phosphorylation | CRK10_ARATH^CRK10_ARATH^Q:1-1044,H:328-669^55.01%ID^E:1e-130^RecName: Full=Cysteine-rich receptor-like protein kinase 10;^Eukaryota; Viridiplantae; Streptophyta; Embryophyta; Tracheophyta; Spermatophyta; Magnoliophyta; eudicotyledons; Gunneridae; Pentapetalae; rosids; malvids; Brassicales; Brassicaceae; Camelineae; Arabidopsis |
| LOC109763170 | TRIAE_CS42_U_TGACv1_641861_AA2105970 | 157 | 130 | 125 | 64 | 66 | 25 | 1.389508 | 0.004896 | up | ENOG410XPC8^copine family | KEGG:ath:AT5G63970 | GO:0005737^cellular_component^cytoplasm`GO:0005634^cellular_component^nucleus`GO:0046872^molecular_function^metal ion binding`GO:0004842^molecular_function^ubiquitin-protein transferase activity`GO:0042742^biological_process^defense response to bacterium`GO:0009867^biological_process^jasmonic acid mediated signaling pathway`GO:0009611^biological_process^response to wounding | RGLG3_ARATH^RGLG3_ARATH^Q:52-1137,H:5-367^64.58%ID^E:3e-165^RecName: Full=E3 ubiquitin-protein ligase RGLG3 {ECO:0000305};^Eukaryota; Viridiplantae; Streptophyta; Embryophyta; Tracheophyta; Spermatophyta; Magnoliophyta; eudicotyledons; Gunneridae; Pentapetalae; rosids; malvids; Brassicales; Brassicaceae; Camelineae; Arabidopsis |
| *MYB70* | TRIAE_CS42_5AL_TGACv1_374035_AA1188520 | 493 | 605 | 728 | 174 | 166 | 351 | 1.387261 | 0.003056 | up | COG5147^Myblike DNAbinding domain containing protein | KEGG:ath:AT5G67300`KO:K09422 | GO:0005634^cellular_component^nucleus`GO:0000981^molecular_function^RNA polymerase II transcription factor activity, sequence-specific DNA binding`GO:0043565^molecular_function^sequence-specific DNA binding`GO:0001135^molecular_function^transcription factor activity, RNA polymerase II transcription factor recruiting`GO:0003700^molecular_function^transcription factor activity, sequence-specific DNA binding`GO:0044212^molecular_function^transcription regulatory region DNA binding`GO:0009738^biological_process^abscisic acid-activated signaling pathway`GO:0030154^biological_process^cell differentiation`GO:0042742^biological_process^defense response to bacterium`GO:0050832^biological_process^defense response to fungus`GO:2000022^biological_process^regulation of jasmonic acid mediated signaling pathway`GO:2000031^biological_process^regulation of salicylic acid mediated signaling pathway`GO:0006357^biological_process^regulation of transcription from RNA polymerase II promoter`GO:0009737^biological_process^response to abscisic acid`GO:0009733^biological_process^response to auxin`GO:0046686^biological_process^response to cadmium ion`GO:0010200^biological_process^response to chitin`GO:0009723^biological_process^response to ethylene`GO:0009739^biological_process^response to gibberellin`GO:0009753^biological_process^response to jasmonic acid`GO:0009751^biological_process^response to salicylic acid`GO:0009651^biological_process^response to salt stress`GO:0009414^biological_process^response to water deprivation`GO:0006351^biological_process^transcription, DNA-templated | MYB44_ARATH^MYB44_ARATH^Q:40-600,H:3-177^58.51%ID^E:5e-59^RecName: Full=Transcription factor MYB44;^Eukaryota; Viridiplantae; Streptophyta; Embryophyta; Tracheophyta; Spermatophyta; Magnoliophyta; eudicotyledons; Gunneridae; Pentapetalae; rosids; malvids; Brassicales; Brassicaceae; Camelineae; Arabidopsis |
| LOC109783019 | TRIAE_CS42_5AL_TGACv1_376042_AA1231170 | 222 | 260 | 345 | 88 | 89 | 136 | 1.386986 | 0.003673 | up | ENOG410YARB^glucan endo-1-3-beta-glucosidase | KEGG:ath:AT1G64760 | GO:0031225^cellular_component^anchored component of membrane`GO:0046658^cellular_component^anchored component of plasma membrane`GO:0005618^cellular_component^cell wall`GO:0005576^cellular_component^extracellular region`GO:0005886^cellular_component^plasma membrane`GO:0009506^cellular_component^plasmodesma`GO:0042973^molecular_function^glucan endo-1,3-beta-D-glucosidase activity`GO:0004553^molecular_function^hydrolase activity, hydrolyzing O-glycosyl compounds`GO:0030247^molecular_function^polysaccharide binding`GO:0005975^biological_process^carbohydrate metabolic process`GO:0071555^biological_process^cell wall organization`GO:0006952^biological_process^defense response | E138_ARATH^E138_ARATH^Q:1-450,H:305-454^64.67%ID^E:2e-67^RecName: Full=Glucan endo-1,3-beta-glucosidase 8;^Eukaryota; Viridiplantae; Streptophyta; Embryophyta; Tracheophyta; Spermatophyta; Magnoliophyta; eudicotyledons; Gunneridae; Pentapetalae; rosids; malvids; Brassicales; Brassicaceae; Camelineae; Arabidopsis |
| *WCK-1* | TRIAE_CS42_4AS_TGACv1_306486_AA1008960 | 1059 | 1214 | 1501 | 330 | 552 | 551 | 1.37965 | 0.003006 | up | ENOG410XNY0^Mitogen-activated protein kinase | KEGG:osa:4332475`KO:K20536 | GO:0005737^cellular_component^cytoplasm`GO:0005634^cellular_component^nucleus`GO:0005524^molecular_function^ATP binding`GO:0004707^molecular_function^MAP kinase activity`GO:0000169^biological_process^activation of MAPK activity involved in osmosensory signaling pathway`GO:0010120^biological_process^camalexin biosynthetic process`GO:0042742^biological_process^defense response to bacterium`GO:0050832^biological_process^defense response to fungus`GO:0010229^biological_process^inflorescence development`GO:0048481^biological_process^plant ovule development`GO:0009555^biological_process^pollen development`GO:0010183^biological_process^pollen tube guidance`GO:1901002^biological_process^positive regulation of response to salt stress`GO:0080136^biological_process^priming of cellular response to stress`GO:0010468^biological_process^regulation of gene expression`GO:0010200^biological_process^response to chitin`GO:0009409^biological_process^response to cold`GO:0006970^biological_process^response to osmotic stress`GO:0006979^biological_process^response to oxidative stress`GO:0010224^biological_process^response to UV-B`GO:0009414^biological_process^response to water deprivation`GO:0009611^biological_process^response to wounding | MPK5_ORYSJ^MPK5_ORYSJ^Q:4-831,H:94-369^90.94%ID^E:0^RecName: Full=Mitogen-activated protein kinase 5;^Eukaryota; Viridiplantae; Streptophyta; Embryophyta; Tracheophyta; Spermatophyta; Magnoliophyta; Liliopsida; Poales; Poaceae; BOP clade; Oryzoideae; Oryzeae; Oryzinae; Oryza; Oryza sativa |
| LOC109734314 | TRIAE_CS42_2AL_TGACv1_093548_AA0282420 | 81 | 58 | 51 | 31 | 13 | 28 | 1.377596 | 0.009724 | up | COG1304^Catalyzes the 1,3-allylic rearrangement of the homoallylic substrate isopentenyl (IPP) to its allylic isomer, dimethylallyl diphosphate (DMAPP) (By similarity) | KEGG:osa:4337048`KO:K11517 | GO:0005782^cellular_component^peroxisomal matrix`GO:0005777^cellular_component^peroxisome`GO:0010181^molecular_function^FMN binding`GO:0008891^molecular_function^glycolate oxidase activity`GO:0052853^molecular_function^long-chain-(S)-2-hydroxy-long-chain-acid oxidase activity`GO:0052854^molecular_function^medium-chain-(S)-2-hydroxy-acid oxidase activity`GO:0052852^molecular_function^very-long-chain-(S)-2-hydroxy-acid oxidase activity`GO:0034641^biological_process^cellular nitrogen compound metabolic process`GO:0010204^biological_process^defense response signaling pathway, resistance gene-independent`GO:0042742^biological_process^defense response to bacterium`GO:0050665^biological_process^hydrogen peroxide biosynthetic process`GO:0019048^biological_process^modulation by virus of host morphology or physiology`GO:0009854^biological_process^oxidative photosynthetic carbon pathway`GO:0009853^biological_process^photorespiration`GO:0010109^biological_process^regulation of photosynthesis | GLO3_ORYSJ^GLO3_ORYSJ^Q:1005-1166,H:304-357^83.33%ID^E:2e-19^RecName: Full=Peroxisomal (S)-2-hydroxy-acid oxidase GLO3;^Eukaryota; Viridiplantae; Streptophyta; Embryophyta; Tracheophyta; Spermatophyta; Magnoliophyta; Liliopsida; Poales; Poaceae; BOP clade; Oryzoideae; Oryzeae; Oryzinae; Oryza; Oryza sativa |
| LOC109755171 | TRIAE_CS42_1DL_TGACv1_061941_AA0206020 | 587 | 858 | 1118 | 230 | 396 | 378 | 1.336825 | 0.004466 | up | ENOG410YIGI^exocyst complex | KEGG:ath:AT5G58430 | GO:0005829^cellular_component^cytosol`GO:0012505^cellular_component^endomembrane system`GO:0000145^cellular_component^exocyst`GO:0070062^cellular_component^extracellular exosome`GO:0045335^cellular_component^phagocytic vesicle`GO:0005886^cellular_component^plasma membrane`GO:0042742^biological_process^defense response to bacterium`GO:0050832^biological_process^defense response to fungus`GO:0006887^biological_process^exocytosis`GO:0009789^biological_process^positive regulation of abscisic acid-activated signaling pathway`GO:0090333^biological_process^regulation of stomatal closure`GO:0009414^biological_process^response to water deprivation | E70B1_ARATH^E70B1_ARATH^Q:1-1953,H:1-618^43.26%ID^E:0^RecName: Full=Exocyst complex component EXO70B1 {ECO:0000303\|PubMed:23944713};^Eukaryota; Viridiplantae; Streptophyta; Embryophyta; Tracheophyta; Spermatophyta; Magnoliophyta; eudicotyledons; Gunneridae; Pentapetalae; rosids; malvids; Brassicales; Brassicaceae; Camelineae; Arabidopsis |
| LOC109747505 | TRIAE_CS42_7DL_TGACv1_604281_AA1996130 | 716 | 1010 | 1849 | 277 | 569 | 562 | 1.331255 | 0.004538 | up | COG0515^Serine Threonine protein kinase | KEGG:ath:AT4G23140 | GO:0016021^cellular_component^integral component of membrane`GO:0005886^cellular_component^plasma membrane`GO:0005524^molecular_function^ATP binding`GO:0004672^molecular_function^protein kinase activity`GO:0004674^molecular_function^protein serine/threonine kinase activity`GO:0042742^biological_process^defense response to bacterium`GO:0000302^biological_process^response to reactive oxygen species | CRK6_ARATH^CRK6_ARATH^Q:64-957,H:338-618^38.08%ID^E:4e-58^RecName: Full=Cysteine-rich receptor-like protein kinase 6;^Eukaryota; Viridiplantae; Streptophyta; Embryophyta; Tracheophyta; Spermatophyta; Magnoliophyta; eudicotyledons; Gunneridae; Pentapetalae; rosids; malvids; Brassicales; Brassicaceae; Camelineae; Arabidopsis |
| LOC109774158 | TRIAE_CS42_U_TGACv1_642789_AA2123260 | 507 | 827 | 1387 | 212 | 310 | 559 | 1.320555 | 0.005077 | up | . | . | GO:0043531^molecular_function^ADP binding`GO:0005524^molecular_function^ATP binding`GO:0006952^biological_process^defense response | RGA1_SOLBU^RGA1_SOLBU^Q:1162-2685,H:518-972^26.86%ID^E:3e-18^RecName: Full=Putative disease resistance protein RGA1;^Eukaryota; Viridiplantae; Streptophyta; Embryophyta; Tracheophyta; Spermatophyta; Magnoliophyta; eudicotyledons; Gunneridae; Pentapetalae; asterids; lamiids; Solanales; Solanaceae; Solanoideae; Solaneae; Solanum |
| LOC109783814 | TRIAE_CS42_4DL_TGACv1_342886_AA1124690 | 843 | 1069 | 933 | 248 | 407 | 508 | 1.270855 | 0.007552 | up | ENOG410ZUVB^May be involved in modulation of pathogen defense and leaf cell death (By similarity) | . | GO:0016021^cellular_component^integral component of membrane`GO:0005516^molecular_function^calmodulin binding`GO:0006952^biological_process^defense response`GO:0009607^biological_process^response to biotic stimulus | MLO_HORVU^MLO_HORVU^Q:728-543,H:468-529^91.94%ID^E:6e-30^RecName: Full=Protein MLO;^Eukaryota; Viridiplantae; Streptophyta; Embryophyta; Tracheophyta; Spermatophyta; Magnoliophyta; Liliopsida; Poales; Poaceae; BOP clade; Pooideae; Triticodae; Triticeae; Hordeinae; Hordeum |
| LOC109738355 | TRIAE_CS42_2DS_TGACv1_177361_AA0574540 | 265 | 358 | 260 | 71 | 174 | 116 | 1.266892 | 0.009336 | up | COG0515^Serine Threonine protein kinase | KEGG:ath:AT3G53810 | GO:0016021^cellular_component^integral component of membrane`GO:0005886^cellular_component^plasma membrane`GO:0005524^molecular_function^ATP binding`GO:0030246^molecular_function^carbohydrate binding`GO:0004674^molecular_function^protein serine/threonine kinase activity`GO:0042742^biological_process^defense response to bacterium`GO:0009555^biological_process^pollen development | LRK42_ARATH^LRK42_ARATH^Q:184-1962,H:61-653^54.68%ID^E:0^RecName: Full=L-type lectin-domain containing receptor kinase IV.2 {ECO:0000303\|PubMed:19773388};^Eukaryota; Viridiplantae; Streptophyta; Embryophyta; Tracheophyta; Spermatophyta; Magnoliophyta; eudicotyledons; Gunneridae; Pentapetalae; rosids; malvids; Brassicales; Brassicaceae; Camelineae; Arabidopsis |
| LOC109733449 | TRIAE_CS42_4BS_TGACv1_328896_AA1095200 | 899 | 1206 | 1814 | 350 | 580 | 707 | 1.245304 | 0.009073 | up | COG0515^Serine Threonine protein kinase | KEGG:ath:AT4G23160 | GO:0016021^cellular_component^integral component of membrane`GO:0005886^cellular_component^plasma membrane`GO:0005524^molecular_function^ATP binding`GO:0004674^molecular_function^protein serine/threonine kinase activity`GO:0042742^biological_process^defense response to bacterium`GO:0006468^biological_process^protein phosphorylation | CRK8_ARATH^CRK8_ARATH^Q:55-957,H:338-631^37.7%ID^E:5e-60^RecName: Full=Cysteine-rich receptor-like protein kinase 8;^Eukaryota; Viridiplantae; Streptophyta; Embryophyta; Tracheophyta; Spermatophyta; Magnoliophyta; eudicotyledons; Gunneridae; Pentapetalae; rosids; malvids; Brassicales; Brassicaceae; Camelineae; Arabidopsis |
| *Glb3* | TRIAE_CS42_3B_TGACv1_223119_AA0776690 | 650 | 582 | 430 | 1717 | 1213 | 935 | -1.23496 | 0.00985 | down | . | . | GO:0005615^cellular_component^extracellular space`GO:0042973^molecular_function^glucan endo-1,3-beta-D-glucosidase activity`GO:0005975^biological_process^carbohydrate metabolic process`GO:0006952^biological_process^defense response | E13B_MAIZE^E13B_MAIZE^Q:64-999,H:23-334^75.8%ID^E:3e-137^RecName: Full=Glucan endo-1,3-beta-glucosidase, acidic isoform;^Eukaryota; Viridiplantae; Streptophyta; Embryophyta; Tracheophyta; Spermatophyta; Magnoliophyta; Liliopsida; Poales; Poaceae; PACMAD clade; Panicoideae; Andropogonodae; Andropogoneae; Tripsacinae; Zea |
| *Pr-1-1* | TRIAE_CS42_5BL_TGACv1_404207_AA1290140 | 1446 | 1157 | 584 | 3847 | 2065 | 1666 | -1.2687 | 0.007293 | down | . | . | GO:0005576^cellular_component^extracellular region`GO:0006952^biological_process^defense response`GO:0009607^biological_process^response to biotic stimulus | PR12_HORVU^PR12_HORVU^Q:1-492,H:1-164^92.07%ID^E:2e-87^RecName: Full=Pathogenesis-related protein PRB1-2;^Eukaryota; Viridiplantae; Streptophyta; Embryophyta; Tracheophyta; Spermatophyta; Magnoliophyta; Liliopsida; Poales; Poaceae; BOP clade; Pooideae; Triticodae; Triticeae; Hordeinae; Hordeum |
| LOC109773965 | TRIAE_CS42_U_TGACv1_641065_AA2083890 | 208 | 256 | 155 | 671 | 456 | 375 | -1.29452 | 0.006729 | down | COG2124^Cytochrome p450 | KEGG:osa:4335096 | GO:0016021^cellular_component^integral component of membrane`GO:0016020^cellular_component^membrane`GO:0020037^molecular_function^heme binding`GO:0005506^molecular_function^iron ion binding`GO:0016709^molecular_function^oxidoreductase activity, acting on paired donors, with incorporation or reduction of molecular oxygen, NAD(P)H as one donor, and incorporation of one atom of oxygen`GO:0006952^biological_process^defense response`GO:0044550^biological_process^secondary metabolite biosynthetic process | C99A2_ORYSJ^C99A2_ORYSJ^Q:1-1290,H:95-526^64.29%ID^E:0^RecName: Full=Cytochrome P450 99A2;^Eukaryota; Viridiplantae; Streptophyta; Embryophyta; Tracheophyta; Spermatophyta; Magnoliophyta; Liliopsida; Poales; Poaceae; BOP clade; Oryzoideae; Oryzeae; Oryzinae; Oryza; Oryza sativa |
| LOC109773967 | TRIAE_CS42_U_TGACv1_641065_AA2083920 | 57 | 107 | 43 | 291 | 139 | 110 | -1.39456 | 0.004061 | down | COG2124^Cytochrome p450 | KEGG:osa:4335096 | GO:0016021^cellular_component^integral component of membrane`GO:0016020^cellular_component^membrane`GO:0020037^molecular_function^heme binding`GO:0005506^molecular_function^iron ion binding`GO:0016709^molecular_function^oxidoreductase activity, acting on paired donors, with incorporation or reduction of molecular oxygen, NAD(P)H as one donor, and incorporation of one atom of oxygen`GO:0006952^biological_process^defense response`GO:0044550^biological_process^secondary metabolite biosynthetic process | C99A2_ORYSJ^C99A2_ORYSJ^Q:151-1530,H:64-531^64.1%ID^E:0^RecName: Full=Cytochrome P450 99A2;^Eukaryota; Viridiplantae; Streptophyta; Embryophyta; Tracheophyta; Spermatophyta; Magnoliophyta; Liliopsida; Poales; Poaceae; BOP clade; Oryzoideae; Oryzeae; Oryzinae; Oryza; Oryza sativa |
| *Cht2* | TRIAE_CS42_2DL_TGACv1_160070_AA0546330 | 251 | 222 | 303 | 857 | 599 | 596 | -1.41495 | 0.002368 | down | COG3979^chitinase | KEGG:osa:4336265`KO:K01183 | GO:0008061^molecular_function^chitin binding`GO:0004568^molecular_function^chitinase activity`GO:0016998^biological_process^cell wall macromolecule catabolic process`GO:0006032^biological_process^chitin catabolic process`GO:0006952^biological_process^defense response`GO:0000272^biological_process^polysaccharide catabolic process | CHI5_ORYSJ^CHI5_ORYSJ^Q:85-690,H:87-288^65.02%ID^E:2e-91^RecName: Full=Chitinase 5;^Eukaryota; Viridiplantae; Streptophyta; Embryophyta; Tracheophyta; Spermatophyta; Magnoliophyta; Liliopsida; Poales; Poaceae; BOP clade; Oryzoideae; Oryzeae; Oryzinae; Oryza; Oryza sativa |
| LOC109744820 | TRIAE_CS42_5DL_TGACv1_435449_AA1450780 | 56 | 52 | 70 | 203 | 143 | 127 | -1.42039 | 0.003537 | down | COG0515^Serine Threonine protein kinase | KEGG:ath:AT2G19190`KO:K16224 | GO:0016021^cellular_component^integral component of membrane`GO:0005524^molecular_function^ATP binding`GO:0004674^molecular_function^protein serine/threonine kinase activity`GO:0042742^biological_process^defense response to bacterium | SIRK_ARATH^SIRK_ARATH^Q:88-2673,H:2-843^38.88%ID^E:1e-176^RecName: Full=Senescence-induced receptor-like serine/threonine-protein kinase;^Eukaryota; Viridiplantae; Streptophyta; Embryophyta; Tracheophyta; Spermatophyta; Magnoliophyta; eudicotyledons; Gunneridae; Pentapetalae; rosids; malvids; Brassicales; Brassicaceae; Camelineae; Arabidopsis |
| LOC109775877 | TRIAE_CS42_5DS_TGACv1_460621_AA1495590 | 46 | 37 | 22 | 79 | 103 | 97 | -1.42998 | 0.004659 | down | . | KEGG:ag:BAM20978`KO:K20506 | GO:0009507^cellular_component^chloroplast`GO:0016787^molecular_function^hydrolase activity`GO:0016829^molecular_function^lyase activity`GO:0006952^biological_process^defense response`GO:0008152^biological_process^metabolic process | TCEA1_TULGE^TCEA1_TULGE^Q:13-927,H:77-383^42.95%ID^E:3e-72^RecName: Full=Tuliposide A-converting enzyme 1, chloroplastic;^Eukaryota; Viridiplantae; Streptophyta; Embryophyta; Tracheophyta; Spermatophyta; Magnoliophyta; Liliopsida; Liliales; Liliaceae; Tulipa |
| *WRKY45* | TRIAE_CS42_2BL_TGACv1_130658_AA0415490 | 315 | 212 | 188 | 899 | 631 | 377 | -1.43303 | 0.002046 | down | ENOG410YZZY^Transcription factor | KEGG:ath:AT3G56400 | GO:0005634^cellular_component^nucleus`GO:0043565^molecular_function^sequence-specific DNA binding`GO:0003700^molecular_function^transcription factor activity, sequence-specific DNA binding`GO:0042742^biological_process^defense response to bacterium`GO:0050832^biological_process^defense response to fungus`GO:0009864^biological_process^induced systemic resistance, jasmonic acid mediated signaling pathway`GO:1900056^biological_process^negative regulation of leaf senescence`GO:0045892^biological_process^negative regulation of transcription, DNA-templated`GO:0031347^biological_process^regulation of defense response`GO:0010200^biological_process^response to chitin`GO:0009753^biological_process^response to jasmonic acid`GO:0009751^biological_process^response to salicylic acid`GO:0009862^biological_process^systemic acquired resistance, salicylic acid mediated signaling pathway`GO:0006351^biological_process^transcription, DNA-templated | WRK70_ARATH^WRK70_ARATH^Q:22-498,H:10-180^37.21%ID^E:8e-19^RecName: Full=Probable WRKY transcription factor 70;^Eukaryota; Viridiplantae; Streptophyta; Embryophyta; Tracheophyta; Spermatophyta; Magnoliophyta; eudicotyledons; Gunneridae; Pentapetalae; rosids; malvids; Brassicales; Brassicaceae; Camelineae; Arabidopsis |
| LOC109755336 | TRIAE_CS42_2AL_TGACv1_092905_AA0266710 | 420 | 209 | 157 | 846 | 626 | 647 | -1.45391 | 0.001672 | down | ENOG4111S1X^protein binding zinc ion binding | KEGG:ath:AT3G12920`KO:K19042 | GO:0005634^cellular_component^nucleus`GO:0046872^molecular_function^metal ion binding`GO:0016740^molecular_function^transferase activity`GO:0006952^biological_process^defense response`GO:0043161^biological_process^proteasome-mediated ubiquitin-dependent protein catabolic process`GO:0043067^biological_process^regulation of programmed cell death | BRG3_ARATH^BRG3_ARATH^Q:382-969,H:128-334^33.33%ID^E:3e-17^RecName: Full=Probable BOI-related E3 ubiquitin-protein ligase 3;^Eukaryota; Viridiplantae; Streptophyta; Embryophyta; Tracheophyta; Spermatophyta; Magnoliophyta; eudicotyledons; Gunneridae; Pentapetalae; rosids; malvids; Brassicales; Brassicaceae; Camelineae; Arabidopsis |
| LOC109747767 | TRIAE_CS42_4DL_TGACv1_344247_AA1145400 | 321 | 235 | 116 | 690 | 626 | 557 | -1.50251 | 0.001099 | down | COG0515^Serine Threonine protein kinase | KEGG:ath:AT2G05940 | GO:0005886^cellular_component^plasma membrane`GO:0005524^molecular_function^ATP binding`GO:0004674^molecular_function^protein serine/threonine kinase activity`GO:0004675^molecular_function^transmembrane receptor protein serine/threonine kinase activity`GO:0007166^biological_process^cell surface receptor signaling pathway`GO:0042742^biological_process^defense response to bacterium`GO:0006468^biological_process^protein phosphorylation | RIPK_ARATH^RIPK_ARATH^Q:184-1134,H:45-361^75.39%ID^E:4e-155^RecName: Full=Serine/threonine-protein kinase RIPK {ECO:0000305};^Eukaryota; Viridiplantae; Streptophyta; Embryophyta; Tracheophyta; Spermatophyta; Magnoliophyta; eudicotyledons; Gunneridae; Pentapetalae; rosids; malvids; Brassicales; Brassicaceae; Camelineae; Arabidopsis |
| AA1883380 | TRIAE_CS42_7BL_TGACv1_577789_AA1883380 | 72 | 107 | 68 | 304 | 218 | 181 | -1.52238 | 0.001241 | down | COG0515^Serine Threonine protein kinase`COG4886^leucine Rich Repeat | KEGG:ath:AT5G46330`KO:K13420 | GO:0005768^cellular_component^endosome`GO:0010008^cellular_component^endosome membrane`GO:0016021^cellular_component^integral component of membrane`GO:0016020^cellular_component^membrane`GO:0005886^cellular_component^plasma membrane`GO:0005524^molecular_function^ATP binding`GO:0004675^molecular_function^transmembrane receptor protein serine/threonine kinase activity`GO:0052544^biological_process^defense response by callose deposition in cell wall`GO:0042742^biological_process^defense response to bacterium`GO:0016045^biological_process^detection of bacterium`GO:0006898^biological_process^receptor-mediated endocytosis`GO:0010359^biological_process^regulation of anion channel activity | FLS2_ARATH^FLS2_ARATH^Q:151-2667,H:30-800^29.44%ID^E:8e-57^RecName: Full=LRR receptor-like serine/threonine-protein kinase FLS2;^Eukaryota; Viridiplantae; Streptophyta; Embryophyta; Tracheophyta; Spermatophyta; Magnoliophyta; eudicotyledons; Gunneridae; Pentapetalae; rosids; malvids; Brassicales; Brassicaceae; Camelineae; Arabidopsis |
| LOC109755504 | TRIAE_CS42_3B_TGACv1_222528_AA0766490 | 194 | 125 | 201 | 419 | 443 | 617 | -1.52458 | 0.000946 | down | . | . | GO:0016021^cellular_component^integral component of membrane`GO:0005509^molecular_function^calcium ion binding`GO:0005245^molecular_function^voltage-gated calcium channel activity`GO:0006952^biological_process^defense response | TPC1_WHEAT^TPC1_WHEAT^Q:1-1416,H:271-742^99.79%ID^E:0^RecName: Full=Two pore calcium channel protein 1;^Eukaryota; Viridiplantae; Streptophyta; Embryophyta; Tracheophyta; Spermatophyta; Magnoliophyta; Liliopsida; Poales; Poaceae; BOP clade; Pooideae; Triticodae; Triticeae; Triticinae; Triticum |
| LOC109738912 | TRIAE_CS42_3B_TGACv1_221435_AA0740740 | 131 | 139 | 123 | 476 | 309 | 336 | -1.52575 | 0.001009 | down | . | . | GO:0030598^molecular_function^rRNA N-glycosylase activity`GO:0006952^biological_process^defense response`GO:0017148^biological_process^negative regulation of translation | JI60_HORVU^JI60_HORVU^Q:2920-3513,H:8-234^24.02%ID^E:7e-06^RecName: Full=60 kDa jasmonate-induced protein;^Eukaryota; Viridiplantae; Streptophyta; Embryophyta; Tracheophyta; Spermatophyta; Magnoliophyta; Liliopsida; Poales; Poaceae; BOP clade; Pooideae; Triticodae; Triticeae; Hordeinae; Hordeum |
| LOC109770817 | TRIAE_CS42_3DL_TGACv1_249487_AA0849950 | 25 | 26 | 19 | 64 | 56 | 81 | -1.53467 | 0.002996 | down | COG0515^Serine Threonine protein kinase | KEGG:ath:AT4G05200 | GO:0016021^cellular_component^integral component of membrane`GO:0005886^cellular_component^plasma membrane`GO:0005524^molecular_function^ATP binding`GO:0004674^molecular_function^protein serine/threonine kinase activity`GO:0042742^biological_process^defense response to bacterium`GO:0006468^biological_process^protein phosphorylation | CRK25_ARATH^CRK25_ARATH^Q:121-2070,H:34-639^39.57%ID^E:4e-150^RecName: Full=Cysteine-rich receptor-like protein kinase 25;^Eukaryota; Viridiplantae; Streptophyta; Embryophyta; Tracheophyta; Spermatophyta; Magnoliophyta; eudicotyledons; Gunneridae; Pentapetalae; rosids; malvids; Brassicales; Brassicaceae; Camelineae; Arabidopsis |
| LOC109782871 | TRIAE_CS42_1BL_TGACv1_032406_AA0129510 | 188 | 181 | 152 | 644 | 501 | 360 | -1.54623 | 0.000774 | down | . | . | GO:0043531^molecular_function^ADP binding`GO:0005524^molecular_function^ATP binding`GO:0006952^biological_process^defense response | RGA2_SOLBU^RGA2_SOLBU^Q:3706-4161,H:829-969^31.65%ID^E:7e-07^RecName: Full=Disease resistance protein RGA2;^Eukaryota; Viridiplantae; Streptophyta; Embryophyta; Tracheophyta; Spermatophyta; Magnoliophyta; eudicotyledons; Gunneridae; Pentapetalae; asterids; lamiids; Solanales; Solanaceae; Solanoideae; Solaneae; Solanum |
| LOC109736245 | TRIAE_CS42_7BL_TGACv1_578754_AA1899570 | 302 | 442 | 438 | 1069 | 1239 | 1231 | -1.59687 | 0.000414 | down | COG4886^leucine Rich Repeat | KEGG:ath:AT1G58410 | GO:0005886^cellular_component^plasma membrane`GO:0043531^molecular_function^ADP binding`GO:0005524^molecular_function^ATP binding`GO:0006952^biological_process^defense response`GO:0007165^biological_process^signal transduction | RX24L_ARATH^RX24L_ARATH^Q:442-1251,H:421-694^27.34%ID^E:8e-10^RecName: Full=Probable disease resistance protein RXW24L;^Eukaryota; Viridiplantae; Streptophyta; Embryophyta; Tracheophyta; Spermatophyta; Magnoliophyta; eudicotyledons; Gunneridae; Pentapetalae; rosids; malvids; Brassicales; Brassicaceae; Camelineae; Arabidopsis |
| *TGA4* | TRIAE_CS42_2DL_TGACv1_159977_AA0545060 | 140 | 107 | 80 | 332 | 365 | 279 | -1.59851 | 0.000543 | down | . | KEGG:osa:9270267`KO:K14431 | GO:0005634^cellular_component^nucleus`GO:0043565^molecular_function^sequence-specific DNA binding`GO:0003700^molecular_function^transcription factor activity, sequence-specific DNA binding`GO:0006952^biological_process^defense response`GO:0006351^biological_process^transcription, DNA-templated | TGAL6_ORYSJ^TGAL6_ORYSJ^Q:112-1191,H:50-397^62.84%ID^E:7e-136^RecName: Full=Transcription factor TGAL6 {ECO:0000305};^Eukaryota; Viridiplantae; Streptophyta; Embryophyta; Tracheophyta; Spermatophyta; Magnoliophyta; Liliopsida; Poales; Poaceae; BOP clade; Oryzoideae; Oryzeae; Oryzinae; Oryza; Oryza sativa |
| LOC109785602 | TRIAE_CS42_2AS_TGACv1_115590_AA0372680 | 39 | 25 | 31 | 77 | 103 | 106 | -1.60646 | 0.001161 | down | COG0515^Serine Threonine protein kinase | KEGG:ath:AT4G05200 | GO:0016021^cellular_component^integral component of membrane`GO:0005886^cellular_component^plasma membrane`GO:0005524^molecular_function^ATP binding`GO:0004674^molecular_function^protein serine/threonine kinase activity`GO:0042742^biological_process^defense response to bacterium`GO:0006468^biological_process^protein phosphorylation | CRK25_ARATH^CRK25_ARATH^Q:88-1632,H:33-512^41.01%ID^E:1e-102^RecName: Full=Cysteine-rich receptor-like protein kinase 25;^Eukaryota; Viridiplantae; Streptophyta; Embryophyta; Tracheophyta; Spermatophyta; Magnoliophyta; eudicotyledons; Gunneridae; Pentapetalae; rosids; malvids; Brassicales; Brassicaceae; Camelineae; Arabidopsis |
| *Pr-1-9* | TRIAE_CS42_5BL_TGACv1_405770_AA1335010 | 94 | 126 | 67 | 429 | 292 | 168 | -1.64564 | 0.000364 | down | . | . | GO:0005576^cellular_component^extracellular region`GO:0006952^biological_process^defense response`GO:0009607^biological_process^response to biotic stimulus | PR1_HORVU^PR1_HORVU^Q:70-495,H:25-163^62.94%ID^E:7e-56^RecName: Full=Pathogenesis-related protein 1;^Eukaryota; Viridiplantae; Streptophyta; Embryophyta; Tracheophyta; Spermatophyta; Magnoliophyta; Liliopsida; Poales; Poaceae; BOP clade; Pooideae; Triticodae; Triticeae; Hordeinae; Hordeum |
| LOC109731407 | TRIAE_CS42_2AS_TGACv1_112273_AA0334660 | 15 | 21 | 38 | 98 | 79 | 56 | -1.6585 | 0.000979 | down | COG2124^Cytochrome p450 | KEGG:osa:4335096 | GO:0016021^cellular_component^integral component of membrane`GO:0016020^cellular_component^membrane`GO:0020037^molecular_function^heme binding`GO:0005506^molecular_function^iron ion binding`GO:0016709^molecular_function^oxidoreductase activity, acting on paired donors, with incorporation or reduction of molecular oxygen, NAD(P)H as one donor, and incorporation of one atom of oxygen`GO:0006952^biological_process^defense response`GO:0044550^biological_process^secondary metabolite biosynthetic process | C99A2_ORYSJ^C99A2_ORYSJ^Q:97-1497,H:54-526^64.48%ID^E:0^RecName: Full=Cytochrome P450 99A2;^Eukaryota; Viridiplantae; Streptophyta; Embryophyta; Tracheophyta; Spermatophyta; Magnoliophyta; Liliopsida; Poales; Poaceae; BOP clade; Oryzoideae; Oryzeae; Oryzinae; Oryza; Oryza sativa |
| LOC109763169 | TRIAE_CS42_U_TGACv1_641861_AA2105960 | 173 | 159 | 134 | 569 | 470 | 445 | -1.68798 | 0.0002 | down | ENOG410XPC8^copine family | KEGG:ath:AT5G63970 | GO:0005737^cellular_component^cytoplasm`GO:0005634^cellular_component^nucleus`GO:0046872^molecular_function^metal ion binding`GO:0004842^molecular_function^ubiquitin-protein transferase activity`GO:0042742^biological_process^defense response to bacterium`GO:0009867^biological_process^jasmonic acid mediated signaling pathway`GO:0009611^biological_process^response to wounding | RGLG3_ARATH^RGLG3_ARATH^Q:352-1380,H:17-360^58.24%ID^E:2e-127^RecName: Full=E3 ubiquitin-protein ligase RGLG3 {ECO:0000305};^Eukaryota; Viridiplantae; Streptophyta; Embryophyta; Tracheophyta; Spermatophyta; Magnoliophyta; eudicotyledons; Gunneridae; Pentapetalae; rosids; malvids; Brassicales; Brassicaceae; Camelineae; Arabidopsis |
| LOC109761725 | TRIAE_CS42_5DL_TGACv1_435153_AA1446820 | 132 | 79 | 91 | 505 | 239 | 221 | -1.68911 | 0.000231 | down | COG0515^Serine Threonine protein kinase | KEGG:ath:AT4G23180 | GO:0016021^cellular_component^integral component of membrane`GO:0005886^cellular_component^plasma membrane`GO:0005524^molecular_function^ATP binding`GO:0004674^molecular_function^protein serine/threonine kinase activity`GO:0042742^biological_process^defense response to bacterium`GO:0006468^biological_process^protein phosphorylation | CRK10_ARATH^CRK10_ARATH^Q:61-1566,H:43-513^38.67%ID^E:2e-86^RecName: Full=Cysteine-rich receptor-like protein kinase 10;^Eukaryota; Viridiplantae; Streptophyta; Embryophyta; Tracheophyta; Spermatophyta; Magnoliophyta; eudicotyledons; Gunneridae; Pentapetalae; rosids; malvids; Brassicales; Brassicaceae; Camelineae; Arabidopsis |
| *Pr-1-16* | TRIAE_CS42_5DL_TGACv1_433848_AA1423670 | 172 | 137 | 92 | 674 | 390 | 253 | -1.73182 | 0.000134 | down | . | . | GO:0005576^cellular_component^extracellular region`GO:0006952^biological_process^defense response`GO:0009607^biological_process^response to biotic stimulus | PR1_HORVU^PR1_HORVU^Q:70-501,H:24-164^63.19%ID^E:2e-48^RecName: Full=Pathogenesis-related protein 1;^Eukaryota; Viridiplantae; Streptophyta; Embryophyta; Tracheophyta; Spermatophyta; Magnoliophyta; Liliopsida; Poales; Poaceae; BOP clade; Pooideae; Triticodae; Triticeae; Hordeinae; Hordeum |
| LOC109760959 | TRIAE_CS42_4AS_TGACv1_306982_AA1015780 | 29 | 19 | 18 | 79 | 98 | 40 | -1.73209 | 0.000595 | down | ENOG410YERP^Transcription factor | . | GO:0005634^cellular_component^nucleus`GO:0003677^molecular_function^DNA binding`GO:0003700^molecular_function^transcription factor activity, sequence-specific DNA binding`GO:0006952^biological_process^defense response`GO:0006351^biological_process^transcription, DNA-templated | ERF1_ORYSJ^ERF1_ORYSJ^Q:235-474,H:99-177^67.5%ID^E:3e-18^RecName: Full=Ethylene-responsive transcription factor 1;^Eukaryota; Viridiplantae; Streptophyta; Embryophyta; Tracheophyta; Spermatophyta; Magnoliophyta; Liliopsida; Poales; Poaceae; BOP clade; Oryzoideae; Oryzeae; Oryzinae; Oryza; Oryza sativa |
| LOC109766243 | TRIAE_CS42_1DL_TGACv1_061159_AA0187450 | 6 | 9 | 12 | 41 | 20 | 30 | -1.74845 | 0.002715 | down | COG0515^Serine Threonine protein kinase | KEGG:osa:4343500 | GO:0016021^cellular_component^integral component of membrane`GO:0005886^cellular_component^plasma membrane`GO:0005524^molecular_function^ATP binding`GO:0004672^molecular_function^protein kinase activity`GO:0004674^molecular_function^protein serine/threonine kinase activity`GO:0042742^biological_process^defense response to bacterium | CRK6_ORYSJ^CRK6_ORYSJ^Q:100-1326,H:37-441^45.37%ID^E:7e-100^RecName: Full=Cysteine-rich receptor-like protein kinase 6 {ECO:0000305};^Eukaryota; Viridiplantae; Streptophyta; Embryophyta; Tracheophyta; Spermatophyta; Magnoliophyta; Liliopsida; Poales; Poaceae; BOP clade; Oryzoideae; Oryzeae; Oryzinae; Oryza; Oryza sativa |
| LOC109781337 | TRIAE_CS42_6BS_TGACv1_514128_AA1655770 | 66 | 86 | 109 | 255 | 311 | 304 | -1.74976 | 0.000134 | down | COG0515^Serine Threonine protein kinase`COG4886^leucine Rich Repeat | KEGG:ath:AT5G46330`KO:K13420 | GO:0005768^cellular_component^endosome`GO:0010008^cellular_component^endosome membrane`GO:0016021^cellular_component^integral component of membrane`GO:0016020^cellular_component^membrane`GO:0005886^cellular_component^plasma membrane`GO:0005524^molecular_function^ATP binding`GO:0004675^molecular_function^transmembrane receptor protein serine/threonine kinase activity`GO:0052544^biological_process^defense response by callose deposition in cell wall`GO:0042742^biological_process^defense response to bacterium`GO:0016045^biological_process^detection of bacterium`GO:0006898^biological_process^receptor-mediated endocytosis`GO:0010359^biological_process^regulation of anion channel activity | FLS2_ARATH^FLS2_ARATH^Q:1297-2484,H:77-427^30.4%ID^E:4e-27^RecName: Full=LRR receptor-like serine/threonine-protein kinase FLS2;^Eukaryota; Viridiplantae; Streptophyta; Embryophyta; Tracheophyta; Spermatophyta; Magnoliophyta; eudicotyledons; Gunneridae; Pentapetalae; rosids; malvids; Brassicales; Brassicaceae; Camelineae; Arabidopsis |
| *MLO* | TRIAE_CS42_5AL_TGACv1_375025_AA1214150 | 543 | 608 | 772 | 2291 | 2184 | 2256 | -1.82076 | 3.82E-05 | down | ENOG410ZUVB^May be involved in modulation of pathogen defense and leaf cell death (By similarity) | . | GO:0016021^cellular_component^integral component of membrane`GO:0005516^molecular_function^calmodulin binding`GO:0006952^biological_process^defense response`GO:0009607^biological_process^response to biotic stimulus | MLO_HORVU^MLO_HORVU^Q:25-1602,H:8-533^89.02%ID^E:0^RecName: Full=Protein MLO;^Eukaryota; Viridiplantae; Streptophyta; Embryophyta; Tracheophyta; Spermatophyta; Magnoliophyta; Liliopsida; Poales; Poaceae; BOP clade; Pooideae; Triticodae; Triticeae; Hordeinae; Hordeum |
| LOC109772543 | TRIAE_CS42_5AL_TGACv1_374411_AA1199540 | 48 | 66 | 46 | 348 | 148 | 127 | -1.96914 | 1.82E-05 | down | . | . | GO:0005576^cellular_component^extracellular region`GO:0006952^biological_process^defense response`GO:0009607^biological_process^response to biotic stimulus | PR1_HORVU^PR1_HORVU^Q:70-501,H:24-164^63.19%ID^E:1e-47^RecName: Full=Pathogenesis-related protein 1;^Eukaryota; Viridiplantae; Streptophyta; Embryophyta; Tracheophyta; Spermatophyta; Magnoliophyta; Liliopsida; Poales; Poaceae; BOP clade; Pooideae; Triticodae; Triticeae; Hordeinae; Hordeum |
| AA0131890 | TRIAE_CS42_1BL_TGACv1_032601_AA0131890 | 414 | 443 | 227 | 1765 | 1587 | 1349 | -2.137 | 9.97E-07 | down | ENOG410YA3P^NA | . | GO:0006952^biological_process^defense response`GO:0009607^biological_process^response to biotic stimulus | PRPX_HORVU^PRPX_HORVU^Q:1-711,H:1-235^88.75%ID^E:4e-144^RecName: Full=Pathogen-related protein;^Eukaryota; Viridiplantae; Streptophyta; Embryophyta; Tracheophyta; Spermatophyta; Magnoliophyta; Liliopsida; Poales; Poaceae; BOP clade; Pooideae; Triticodae; Triticeae; Hordeinae; Hordeum |
| LOC109750033 | TRIAE_CS42_2AL_TGACv1_094756_AA0302660 | 12 | 19 | 8 | 77 | 59 | 70 | -2.40627 | 1.85E-06 | down | . | . | GO:0043531^molecular_function^ADP binding`GO:0005524^molecular_function^ATP binding`GO:0006952^biological_process^defense response | RGA4_SOLBU^RGA4_SOLBU^Q:232-3315,H:1-988^26.26%ID^E:7e-85^RecName: Full=Putative disease resistance protein RGA4;^Eukaryota; Viridiplantae; Streptophyta; Embryophyta; Tracheophyta; Spermatophyta; Magnoliophyta; eudicotyledons; Gunneridae; Pentapetalae; asterids; lamiids; Solanales; Solanaceae; Solanoideae; Solaneae; Solanum |
| LOC109773062 | TRIAE_CS42_1BL_TGACv1_034231_AA0143910 | 2 | 4 | 3 | 19 | 16 | 14 | -2.40827 | 0.000532 | down | . | . | GO:0043531^molecular_function^ADP binding`GO:0005524^molecular_function^ATP binding`GO:0006952^biological_process^defense response | RGA1_SOLBU^RGA1_SOLBU^Q:25-2982,H:5-930^32.87%ID^E:1e-133^RecName: Full=Putative disease resistance protein RGA1;^Eukaryota; Viridiplantae; Streptophyta; Embryophyta; Tracheophyta; Spermatophyta; Magnoliophyta; eudicotyledons; Gunneridae; Pentapetalae; asterids; lamiids; Solanales; Solanaceae; Solanoideae; Solaneae; Solanum |
| AA1390550 | TRIAE_CS42_5BS_TGACv1_424552_AA1390550 | 8 | 8 | 3 | 30 | 42 | 30 | -2.42124 | 2.19E-05 | down | . | KEGG:ag:BAM20979`KO:K20506 | GO:0009507^cellular_component^chloroplast`GO:0016787^molecular_function^hydrolase activity`GO:0016829^molecular_function^lyase activity`GO:0006952^biological_process^defense response`GO:0008152^biological_process^metabolic process | TCEA2_TULGE^TCEA2_TULGE^Q:25-927,H:77-380^43.99%ID^E:3e-79^RecName: Full=Tuliposide A-converting enzyme 2, chloroplastic;^Eukaryota; Viridiplantae; Streptophyta; Embryophyta; Tracheophyta; Spermatophyta; Magnoliophyta; Liliopsida; Liliales; Liliaceae; Tulipa |
| LOC109758911 | TRIAE_CS42_U_TGACv1_642356_AA2116260 | 4 | 7 | 5 | 47 | 18 | 27 | -2.50527 | 1.96E-05 | down | . | . | GO:0043531^molecular_function^ADP binding`GO:0005524^molecular_function^ATP binding`GO:0006952^biological_process^defense response | RGA3_SOLBU^RGA3_SOLBU^Q:595-1986,H:139-568^32.26%ID^E:1e-53^RecName: Full=Putative disease resistance protein RGA3;^Eukaryota; Viridiplantae; Streptophyta; Embryophyta; Tracheophyta; Spermatophyta; Magnoliophyta; eudicotyledons; Gunneridae; Pentapetalae; asterids; lamiids; Solanales; Solanaceae; Solanoideae; Solaneae; Solanum |
| LOC109747310 | TRIAE_CS42_U_TGACv1_647004_AA2147290 | 320 | 305 | 352 | 1368 | 2566 | 1688 | -2.54444 | 4.33E-09 | down | COG2124^Cytochrome p450 | KEGG:osa:4329721 | GO:0016021^cellular_component^integral component of membrane`GO:0016020^cellular_component^membrane`GO:0102597^molecular_function^3alpha-hydroxy-ent-sandaracopimardiene 9-beta-monooxygenase activity`GO:0020037^molecular_function^heme binding`GO:0005506^molecular_function^iron ion binding`GO:0004497^molecular_function^monooxygenase activity`GO:0016709^molecular_function^oxidoreductase activity, acting on paired donors, with incorporation or reduction of molecular oxygen, NAD(P)H as one donor, and incorporation of one atom of oxygen`GO:0006952^biological_process^defense response`GO:0051502^biological_process^diterpene phytoalexin biosynthetic process`GO:0055114^biological_process^oxidation-reduction process | C76M5_ORYSJ^C76M5_ORYSJ^Q:160-1425,H:61-471^70.38%ID^E:0^RecName: Full=Cytochrome P450 76M5 {ECO:0000305};^Eukaryota; Viridiplantae; Streptophyta; Embryophyta; Tracheophyta; Spermatophyta; Magnoliophyta; Liliopsida; Poales; Poaceae; BOP clade; Oryzoideae; Oryzeae; Oryzinae; Oryza; Oryza sativa |
| LOC109760257 | TRIAE_CS42_3DL_TGACv1_253018_AA0892990 | 63 | 48 | 56 | 405 | 464 | 328 | -2.85718 | 1.52E-10 | down | COG0515^Serine Threonine protein kinase | KEGG:ath:AT5G48380 | GO:0016021^cellular_component^integral component of membrane`GO:0005886^cellular_component^plasma membrane`GO:0009506^cellular_component^plasmodesma`GO:0005524^molecular_function^ATP binding`GO:0016301^molecular_function^kinase activity`GO:0004672^molecular_function^protein kinase activity`GO:0033612^molecular_function^receptor serine/threonine kinase binding`GO:0042742^biological_process^defense response to bacterium`GO:0060548^biological_process^negative regulation of cell death`GO:0031348^biological_process^negative regulation of defense response | Y5838_ARATH^Y5838_ARATH^Q:64-759,H:27-253^44.58%ID^E:4e-58^RecName: Full=Probably inactive leucine-rich repeat receptor-like protein kinase At5g48380;^Eukaryota; Viridiplantae; Streptophyta; Embryophyta; Tracheophyta; Spermatophyta; Magnoliophyta; eudicotyledons; Gunneridae; Pentapetalae; rosids; malvids; Brassicales; Brassicaceae; Camelineae; Arabidopsis |
| LOC109747282 | TRIAE_CS42_1DL_TGACv1_062188_AA0210280 | 15 | 19 | 20 | 123 | 129 | 149 | -2.89916 | 1.11E-09 | down | ENOG410Y41C^Metacaspase | KEGG:ath:AT1G02170 | GO:0004197^molecular_function^cysteine-type endopeptidase activity`GO:0006952^biological_process^defense response`GO:0043068^biological_process^positive regulation of programmed cell death | MCA1_ARATH^MCA1_ARATH^Q:43-1146,H:18-367^49.6%ID^E:5e-115^RecName: Full=Metacaspase-1;^Eukaryota; Viridiplantae; Streptophyta; Embryophyta; Tracheophyta; Spermatophyta; Magnoliophyta; eudicotyledons; Gunneridae; Pentapetalae; rosids; malvids; Brassicales; Brassicaceae; Camelineae; Arabidopsis |
| LOC109743039 | TRIAE_CS42_3DL_TGACv1_251716_AA0884140 | 46 | 63 | 67 | 608 | 653 | 525 | -3.35474 | 6E-14 | down | COG0515^Serine Threonine protein kinase | KEGG:ath:AT5G48380 | GO:0016021^cellular_component^integral component of membrane`GO:0005886^cellular_component^plasma membrane`GO:0009506^cellular_component^plasmodesma`GO:0005524^molecular_function^ATP binding`GO:0016301^molecular_function^kinase activity`GO:0004672^molecular_function^protein kinase activity`GO:0033612^molecular_function^receptor serine/threonine kinase binding`GO:0042742^biological_process^defense response to bacterium`GO:0060548^biological_process^negative regulation of cell death`GO:0031348^biological_process^negative regulation of defense response | Y5838_ARATH^Y5838_ARATH^Q:61-627,H:29-219^52.88%ID^E:1e-54^RecName: Full=Probably inactive leucine-rich repeat receptor-like protein kinase At5g48380;^Eukaryota; Viridiplantae; Streptophyta; Embryophyta; Tracheophyta; Spermatophyta; Magnoliophyta; eudicotyledons; Gunneridae; Pentapetalae; rosids; malvids; Brassicales; Brassicaceae; Camelineae; Arabidopsis |
| LOC109736246 | TRIAE_CS42_7BL_TGACv1_578754_AA1899590 | 23 | 48 | 32 | 360 | 407 | 343 | -3.44083 | 4.35E-14 | down | . | KEGG:osa:4327497 | GO:0005737^cellular_component^cytoplasm`GO:0005886^cellular_component^plasma membrane`GO:0005544^molecular_function^calcium-dependent phospholipid binding`GO:0006952^biological_process^defense response | ERG1_ORYSJ^ERG1_ORYSJ^Q:19-417,H:6-152^23.81%ID^E:4e-10^RecName: Full=Elicitor-responsive protein 1;^Eukaryota; Viridiplantae; Streptophyta; Embryophyta; Tracheophyta; Spermatophyta; Magnoliophyta; Liliopsida; Poales; Poaceae; BOP clade; Oryzoideae; Oryzeae; Oryzinae; Oryza; Oryza sativa |
| LOC100827979 | TRIAE_CS42_2BL_TGACv1_130094_AA0403640 | 32 | 33 | 46 | 646 | 531 | 520 | -3.94231 | 8.00E-18 | down | ENOG410XQ8V^synthase | KEGG:ath:AT4G03550`KO:K11000 | GO:0000148^cellular_component^1,3-beta-D-glucan synthase complex`GO:0005794^cellular_component^Golgi apparatus`GO:0016021^cellular_component^integral component of membrane`GO:0005886^cellular_component^plasma membrane`GO:0009506^cellular_component^plasmodesma`GO:0003843^molecular_function^1,3-beta-D-glucan synthase activity`GO:0006075^biological_process^(1->3)-beta-D-glucan biosynthetic process`GO:0006952^biological_process^defense response`GO:0052542^biological_process^defense response by callose deposition`GO:0052544^biological_process^defense response by callose deposition in cell wall`GO:0009870^biological_process^defense response signaling pathway, resistance gene-dependent`GO:0042742^biological_process^defense response to bacterium`GO:0050832^biological_process^defense response to fungus`GO:0009965^biological_process^leaf morphogenesis`GO:0010150^biological_process^leaf senescence`GO:0009555^biological_process^pollen development`GO:0008360^biological_process^regulation of cell shape`GO:0000003^biological_process^reproduction`GO:0009620^biological_process^response to fungus`GO:0009863^biological_process^salicylic acid mediated signaling pathway | CALSC_ARATH^CALSC_ARATH^Q:49-3174,H:475-1517^65.65%ID^E:0^RecName: Full=Callose synthase 12;^Eukaryota; Viridiplantae; Streptophyta; Embryophyta; Tracheophyta; Spermatophyta; Magnoliophyta; eudicotyledons; Gunneridae; Pentapetalae; rosids; malvids; Brassicales; Brassicaceae; Camelineae; Arabidopsis |
| LOC109732331 | TRIAE_CS42_3B_TGACv1_221313_AA0736930 | 17 | 13 | 9 | 316 | 181 | 164 | -4.08498 | 4.39E-17 | down | ENOG410ZNK7^Somatic embryogenesis receptor kinase | KEGG:osa:4336035 | GO:0016021^cellular_component^integral component of membrane`GO:0005886^cellular_component^plasma membrane`GO:0005524^molecular_function^ATP binding`GO:0005102^molecular_function^receptor binding`GO:0004675^molecular_function^transmembrane receptor protein serine/threonine kinase activity`GO:0009742^biological_process^brassinosteroid mediated signaling pathway`GO:0030154^biological_process^cell differentiation`GO:0007166^biological_process^cell surface receptor signaling pathway`GO:0006952^biological_process^defense response`GO:0006468^biological_process^protein phosphorylation | SERK2_ORYSJ^SERK2_ORYSJ^Q:67-537,H:31-187^71.34%ID^E:3e-65^RecName: Full=LRR receptor kinase SERK2 {ECO:0000305};^Eukaryota; Viridiplantae; Streptophyta; Embryophyta; Tracheophyta; Spermatophyta; Magnoliophyta; Liliopsida; Poales; Poaceae; BOP clade; Oryzoideae; Oryzeae; Oryzinae; Oryza; Oryza sativa |
| LOC109770200 | TRIAE_CS42_2BS_TGACv1_149972_AA0497860 | 16 | 21 | 29 | 238 | 497 | 523 | -4.26197 | 1.65E-19 | down | COG0515^Serine Threonine protein kinase | KEGG:ath:AT1G69790`KO:K04733 | GO:0005886^cellular_component^plasma membrane`GO:0005524^molecular_function^ATP binding`GO:0004675^molecular_function^transmembrane receptor protein serine/threonine kinase activity`GO:0007166^biological_process^cell surface receptor signaling pathway`GO:0006952^biological_process^defense response`GO:0006468^biological_process^protein phosphorylation | PBL18_ARATH^PBL18_ARATH^Q:205-1116,H:68-365^62.5%ID^E:2e-134^RecName: Full=Probable serine/threonine-protein kinase PBL18 {ECO:0000305};^Eukaryota; Viridiplantae; Streptophyta; Embryophyta; Tracheophyta; Spermatophyta; Magnoliophyta; eudicotyledons; Gunneridae; Pentapetalae; rosids; malvids; Brassicales; Brassicaceae; Camelineae; Arabidopsis |
| LOC109773754 | TRIAE_CS42_2AS_TGACv1_114810_AA0369470 | 7 | 7 | 5 | 198 | 135 | 160 | -4.68392 | 2.77E-19 | down | . | . | GO:0043531^molecular_function^ADP binding`GO:0005524^molecular_function^ATP binding`GO:0006952^biological_process^defense response | RGA4_SOLBU^RGA4_SOLBU^Q:355-1704,H:142-606^26.46%ID^E:1e-42^RecName: Full=Putative disease resistance protein RGA4;^Eukaryota; Viridiplantae; Streptophyta; Embryophyta; Tracheophyta; Spermatophyta; Magnoliophyta; eudicotyledons; Gunneridae; Pentapetalae; asterids; lamiids; Solanales; Solanaceae; Solanoideae; Solaneae; Solanum |
| LOC100826942 | TRIAE_CS42_6BL_TGACv1_509328_AA1629790 | 87 | 110 | 138 | 2918 | 3000 | 2826 | -4.71777 | 1.08E-24 | down | . | . | GO:0005789^cellular_component^endoplasmic reticulum membrane`GO:0016021^cellular_component^integral component of membrane`GO:0005634^cellular_component^nucleus`GO:0005886^cellular_component^plasma membrane`GO:0005524^molecular_function^ATP binding`GO:0004674^molecular_function^protein serine/threonine kinase activity`GO:0006952^biological_process^defense response`GO:0031349^biological_process^positive regulation of defense response`GO:1900426^biological_process^positive regulation of defense response to bacterium`GO:0046777^biological_process^protein autophosphorylation | XA21_ORYSI^XA21_ORYSI^Q:1-681,H:789-1013^42.11%ID^E:1e-48^RecName: Full=Receptor kinase-like protein Xa21 {ECO:0000303\|Ref.1};^Eukaryota; Viridiplantae; Streptophyta; Embryophyta; Tracheophyta; Spermatophyta; Magnoliophyta; Liliopsida; Poales; Poaceae; BOP clade; Oryzoideae; Oryzeae; Oryzinae; Oryza; Oryza sativa |
| LOC109781548 | TRIAE_CS42_2DS_TGACv1_177399_AA0575660 | 3 | 3 | 7 | 200 | 302 | 322 | -5.95953 | 1.69E-27 | down | ENOG410YBN4^G-type lectin S-receptor-like serine threonine-protein kinase | . | GO:0016021^cellular_component^integral component of membrane`GO:0005524^molecular_function^ATP binding`GO:0030246^molecular_function^carbohydrate binding`GO:0004674^molecular_function^protein serine/threonine kinase activity`GO:0006952^biological_process^defense response | LERK2_ORYSI^LERK2_ORYSI^Q:58-2445,H:18-810^73.16%ID^E:0^RecName: Full=G-type lectin S-receptor-like serine/threonine-protein kinase LECRK2 {ECO:0000305};^Eukaryota; Viridiplantae; Streptophyta; Embryophyta; Tracheophyta; Spermatophyta; Magnoliophyta; Liliopsida; Poales; Poaceae; BOP clade; Oryzoideae; Oryzeae; Oryzinae; Oryza; Oryza sativa |
