# Supplementary material for: A cosmopolitan fungal pathogen of dicots adopts an endophytic lifestyle on cereal crops and protects them from major fungal diseases
Source: ISME J. 2020 Aug 19;14(12):3120–35. doi: 10.1038/s41396-020-00744-6 (PMC7784893; doi:10.1038/s41396-020-00744-6)
Supplement: Supplementary file 5 — Supplementary Table 5 [file 41396_2020_744_MOESM5_ESM.docx]

**Supplementary Table 5** Wheat DEGs associated with the Pattern-Triggered Immunity (PTI) and Effector-Triggered Immunity (ETI)pathway in DT-8 treated and control wheat leaf leaves

| **gene** | **gene_id** | **DT-8 Sample 1_count** | **DT-8 Sample 2_count** | **DT-8 Sample 3_count** | **Control Sample 1_count** | **Control Sample 2_count** | **Control Sample 3_count** | **logFC** | **FDR** | **exp** | **eggnog** | **Kegg** | **GO** | **uniprot_hit** |
| --- | --- | --- | --- | --- | --- | --- | --- | --- | --- | --- | --- | --- | --- | --- |
| LOC109760120 | TRIAE_CS42_1AL_TGACv1_000890_AA0021220 | 112 | 126 | 107 | 20 | 30 | 42 | 1.882743 | 8.62E-05 | up | COG5126^Calcium-binding protein | KEGG:osa:4339701`KO:K13448 | GO:0005509^molecular_function^calcium ion binding | CML14_ORYSJ^CML14_ORYSJ^Q:4-507,H:9-172^79.76%ID^E:3e-69^RecName: Full=Probable calcium-binding protein CML14;^Eukaryota; Viridiplantae; Streptophyta; Embryophyta; Tracheophyta; Spermatophyta; Magnoliophyta; Liliopsida; Poales; Poaceae; BOP clade; Oryzoideae; Oryzeae; Oryzinae; Oryza; Oryza sativa |
| LOC109734638 | TRIAE_CS42_7BS_TGACv1_592906_AA1946090 | 209 | 225 | 176 | 27 | 19 | 14 | 3.320694 | 1.49E-12 | up | ENOG410XQ5A^mitogen-activated protein kinase kinase | KEGG:ath:AT3G21220`KO:K13413 | GO:0005737^cellular_component^cytoplasm`GO:0005524^molecular_function^ATP binding`GO:0004674^molecular_function^protein serine/threonine kinase activity`GO:0032147^biological_process^activation of protein kinase activity`GO:0009814^biological_process^defense response, incompatible interaction`GO:0010227^biological_process^floral organ abscission`GO:0010229^biological_process^inflorescence development`GO:0009626^biological_process^plant-type hypersensitive response`GO:0010365^biological_process^positive regulation of ethylene biosynthetic process`GO:0007346^biological_process^regulation of mitotic cell cycle`GO:0023014^biological_process^signal transduction by protein phosphorylation`GO:0031098^biological_process^stress-activated protein kinase signaling cascade | M2K5_ARATH^M2K5_ARATH^Q:211-996,H:67-325^70.61%ID^E:1e-126^RecName: Full=Mitogen-activated protein kinase kinase 5;^Eukaryota; Viridiplantae; Streptophyta; Embryophyta; Tracheophyta; Spermatophyta; Magnoliophyta; eudicotyledons; Gunneridae; Pentapetalae; rosids; malvids; Brassicales; Brassicaceae; Camelineae; Arabidopsis |
| LOC109786129 | TRIAE_CS42_5AL_TGACv1_374180_AA1192590 | 222 | 245 | 243 | 126 | 62 | 53 | 1.546151 | 0.001008 | up | ENOG4112BTF^WRKY transcription factor | KEGG:ath:AT5G56270`KO:K18835 | GO:0005634^cellular_component^nucleus`GO:0043565^molecular_function^sequence-specific DNA binding`GO:0003700^molecular_function^transcription factor activity, sequence-specific DNA binding`GO:0030010^biological_process^establishment of cell polarity`GO:0009942^biological_process^longitudinal axis specification`GO:0009555^biological_process^pollen development`GO:0006355^biological_process^regulation of transcription, DNA-templated`GO:0006351^biological_process^transcription, DNA-templated | WRKY2_ARATH^WRKY2_ARATH^Q:1-1740,H:1-685^41.38%ID^E:3e-113^RecName: Full=Probable WRKY transcription factor 2 {ECO:0000303\|Ref.1};^Eukaryota; Viridiplantae; Streptophyta; Embryophyta; Tracheophyta; Spermatophyta; Magnoliophyta; eudicotyledons; Gunneridae; Pentapetalae; rosids; malvids; Brassicales; Brassicaceae; Camelineae; Arabidopsis |
| *RbohI* | TRIAE_CS42_4BL_TGACv1_321503_AA1061470 | 1140 | 1408 | 1791 | 536 | 630 | 570 | 1.307373 | 0.00547 | up | ENOG410XNZY^NADPH Oxidase | KEGG:sot:102598898`KO:K13447 | GO:0016021^cellular_component^integral component of membrane`GO:0005509^molecular_function^calcium ion binding`GO:0050664^molecular_function^oxidoreductase activity, acting on NAD(P)H, oxygen as acceptor`GO:0004601^molecular_function^peroxidase activity | RBOHC_SOLTU^RBOHC_SOLTU^Q:67-2367,H:31-810^66.25%ID^E:0^RecName: Full=Respiratory burst oxidase homolog protein C;^Eukaryota; Viridiplantae; Streptophyta; Embryophyta; Tracheophyta; Spermatophyta; Magnoliophyta; eudicotyledons; Gunneridae; Pentapetalae; asterids; lamiids; Solanales; Solanaceae; Solanoideae; Solaneae; Solanum |
