# Supplementary material for: A cosmopolitan fungal pathogen of dicots adopts an endophytic lifestyle on cereal crops and protects them from major fungal diseases
Source: ISME J. 2020 Aug 19;14(12):3120–35. doi: 10.1038/s41396-020-00744-6 (PMC7784893; doi:10.1038/s41396-020-00744-6)
Supplement: Supplementary file 6 — Supplementary Table 6 [file 41396_2020_744_MOESM6_ESM.docx]

**Supplementary Table 6** Wheat DEGs associated with the chloroplast in DT-8 treated and control wheat flag leaves

| **gene** | **gene_id** | **DT-8 Sample 1_count** | **DT-8 Sample 2_count** | **DT-8 Sample 3_count** | **Control Sample 1_count** | **Control Sample 2_count** | **Control Sample 3_count** | **logFC** | **FDR** | **exp** | **eggnog** | **Kegg** | **GO** | **uniprot_hit** |
| --- | --- | --- | --- | --- | --- | --- | --- | --- | --- | --- | --- | --- | --- | --- |
| LOC109783704 | TRIAE_CS42_7BS_TGACv1_592411_AA1937580 | 927 | 1022 | 698 | 18 | 13 | 10 | 5.980929 | 3.53E-32 | up | COG4638^rieske 2fe-2S domain-containing protein | KEGG:ath:AT4G25650 | GO:0009507^cellular_component^chloroplast`GO:0009941^cellular_component^chloroplast envelope`GO:0009706^cellular_component^chloroplast inner membrane`GO:0016021^cellular_component^integral component of membrane`GO:0009536^cellular_component^plastid`GO:0051537^molecular_function^2 iron, 2 sulfur cluster binding`GO:0010277^molecular_function^chlorophyllide a oxygenase [overall] activity`GO:0046872^molecular_function^metal ion binding`GO:0015031^biological_process^protein transport | PTC52_ARATH^PTC52_ARATH^Q:190-1506,H:73-557^48.48%ID^E:1e-160^RecName: Full=Protochlorophyllide-dependent translocon component 52, chloroplastic;^Eukaryota; Viridiplantae; Streptophyta; Embryophyta; Tracheophyta; Spermatophyta; Magnoliophyta; eudicotyledons; Gunneridae; Pentapetalae; rosids; malvids; Brassicales; Brassicaceae; Camelineae; Arabidopsis |
| LOC109745952 | TRIAE_CS42_4BS_TGACv1_328584_AA1090480 | 329 | 365 | 242 | 8 | 5 | 9 | 5.366822 | 4.13E-25 | up | . | . | GO:0009706^cellular_component^chloroplast inner membrane`GO:0005516^molecular_function^calmodulin binding`GO:0016491^molecular_function^oxidoreductase activity`GO:0015031^biological_process^protein transport | TIC32_PEA^TIC32_PEA^Q:10-942,H:2-313^66.35%ID^E:2e-144^RecName: Full=Short-chain dehydrogenase TIC 32, chloroplastic;^Eukaryota; Viridiplantae; Streptophyta; Embryophyta; Tracheophyta; Spermatophyta; Magnoliophyta; eudicotyledons; Gunneridae; Pentapetalae; rosids; fabids; Fabales; Fabaceae; Papilionoideae; Fabeae; Pisum |
| LOC109772271 | TRIAE_CS42_1AL_TGACv1_001907_AA0036520 | 784 | 860 | 982 | 27 | 40 | 37 | 4.635841 | 4.31E-23 | up | COG0265^Serine protease | KEGG:ath:AT3G27925 | GO:0009507^cellular_component^chloroplast`GO:0009534^cellular_component^chloroplast thylakoid`GO:0009535^cellular_component^chloroplast thylakoid membrane`GO:0005634^cellular_component^nucleus`GO:0009579^cellular_component^thylakoid`GO:0031977^cellular_component^thylakoid lumen`GO:0004252^molecular_function^serine-type endopeptidase activity`GO:0008236^molecular_function^serine-type peptidase activity`GO:0010206^biological_process^photosystem II repair`GO:0030163^biological_process^protein catabolic process`GO:0009735^biological_process^response to cytokinin | DEGP1_ARATH^DEGP1_ARATH^Q:277-1101,H:106-380^91.27%ID^E:2e-178^RecName: Full=Protease Do-like 1, chloroplastic;^Eukaryota; Viridiplantae; Streptophyta; Embryophyta; Tracheophyta; Spermatophyta; Magnoliophyta; eudicotyledons; Gunneridae; Pentapetalae; rosids; malvids; Brassicales; Brassicaceae; Camelineae; Arabidopsis |
| LOC109734575 | TRIAE_CS42_1DS_TGACv1_080190_AA0242660 | 734 | 1112 | 1308 | 38 | 69 | 63 | 4.19493 | 3.66E-20 | up | COG0227^50s ribosomal protein L28 | KEGG:ath:AT2G33450`KO:K02902 | GO:0009507^cellular_component^chloroplast`GO:0009941^cellular_component^chloroplast envelope`GO:0009570^cellular_component^chloroplast stroma`GO:0022625^cellular_component^cytosolic large ribosomal subunit`GO:0016020^cellular_component^membrane`GO:0003735^molecular_function^structural constituent of ribosome`GO:0006412^biological_process^translation | RK28_ARATH^RK28_ARATH^Q:169-423,H:59-143^84.71%ID^E:8e-45^RecName: Full=50S ribosomal protein L28, chloroplastic;^Eukaryota; Viridiplantae; Streptophyta; Embryophyta; Tracheophyta; Spermatophyta; Magnoliophyta; eudicotyledons; Gunneridae; Pentapetalae; rosids; malvids; Brassicales; Brassicaceae; Camelineae; Arabidopsis |
| LOC109778458 | TRIAE_CS42_5AL_TGACv1_378403_AA1253130 | 143 | 138 | 148 | 7 | 12 | 17 | 3.541636 | 4.48E-13 | up | COG0152^SAICAR synthetase | KEGG:ath:AT3G21110`KO:K01923 | GO:0009507^cellular_component^chloroplast`GO:0009570^cellular_component^chloroplast stroma`GO:0005524^molecular_function^ATP binding`GO:0004639^molecular_function^phosphoribosylaminoimidazolesuccinocarboxamide synthase activity`GO:0006189^biological_process^'de novo' IMP biosynthetic process`GO:0009733^biological_process^response to auxin | PUR7_ARATH^PUR7_ARATH^Q:199-1191,H:78-408^73.72%ID^E:0^RecName: Full=Phosphoribosylaminoimidazole-succinocarboxamide synthase, chloroplastic;^Eukaryota; Viridiplantae; Streptophyta; Embryophyta; Tracheophyta; Spermatophyta; Magnoliophyta; eudicotyledons; Gunneridae; Pentapetalae; rosids; malvids; Brassicales; Brassicaceae; Camelineae; Arabidopsis |
| LOC109773878 | TRIAE_CS42_3DS_TGACv1_271613_AA0903850 | 1514 | 1549 | 1571 | 86 | 172 | 141 | 3.515624 | 1.68E-15 | up | ENOG410ZHAF^protein EXECUTER 1, chloroplastic-like | KEGG:osa:4326159 | GO:0009507^cellular_component^chloroplast`GO:0042651^cellular_component^thylakoid membrane`GO:0000304^biological_process^response to singlet oxygen | EXEC2_ORYSJ^EXEC2_ORYSJ^Q:181-1863,H:59-618^85.74%ID^E:0^RecName: Full=Protein EXECUTER 2, chloroplastic {ECO:0000305};^Eukaryota; Viridiplantae; Streptophyta; Embryophyta; Tracheophyta; Spermatophyta; Magnoliophyta; Liliopsida; Poales; Poaceae; BOP clade; Oryzoideae; Oryzeae; Oryzinae; Oryza; Oryza sativa |
| LOC100832197 | TRIAE_CS42_4BS_TGACv1_329699_AA1103360 | 1475 | 1629 | 1946 | 107 | 210 | 209 | 3.243645 | 1.25E-13 | up | ENOG410XPWC^solute carrier family 17 | KEGG:osa:4347913 | GO:0009706^cellular_component^chloroplast inner membrane`GO:0016021^cellular_component^integral component of membrane`GO:0009536^cellular_component^plastid`GO:0005315^molecular_function^inorganic phosphate transmembrane transporter activity`GO:0015229^molecular_function^L-ascorbic acid transporter activity`GO:0098656^biological_process^anion transmembrane transport`GO:0010028^biological_process^xanthophyll cycle | PHT44_ORYSJ^PHT44_ORYSJ^Q:190-1698,H:66-561^81.18%ID^E:0^RecName: Full=Probable anion transporter 4, chloroplastic;^Eukaryota; Viridiplantae; Streptophyta; Embryophyta; Tracheophyta; Spermatophyta; Magnoliophyta; Liliopsida; Poales; Poaceae; BOP clade; Oryzoideae; Oryzeae; Oryzinae; Oryza; Oryza sativa |
| LOC109742559 | TRIAE_CS42_6BL_TGACv1_499991_AA1596700 | 229 | 163 | 131 | 28 | 17 | 18 | 3.025999 | 1.08E-10 | up | COG0534^Mate efflux family protein | KEGG:ath:AT2G45980 | GO:0031969^cellular_component^chloroplast membrane`GO:0009570^cellular_component^chloroplast stroma`GO:0005829^cellular_component^cytosol`GO:0005783^cellular_component^endoplasmic reticulum`GO:0005789^cellular_component^endoplasmic reticulum membrane`GO:0016021^cellular_component^integral component of membrane`GO:0043231^cellular_component^intracellular membrane-bounded organelle`GO:1904962^biological_process^plastid to vacuole vesicle-mediated transport`GO:0071211^biological_process^protein targeting to vacuole involved in autophagy | ATI1_ARATH^ATI1_ARATH^Q:37-762,H:9-203^27.69%ID^E:3e-17^RecName: Full=ATG8-interacting protein 1 {ECO:0000303\|PubMed:22253227};^Eukaryota; Viridiplantae; Streptophyta; Embryophyta; Tracheophyta; Spermatophyta; Magnoliophyta; eudicotyledons; Gunneridae; Pentapetalae; rosids; malvids; Brassicales; Brassicaceae; Camelineae; Arabidopsis |
| LOC109774911 | TRIAE_CS42_7BL_TGACv1_577677_AA1880880 | 75 | 74 | 115 | 7 | 13 | 16 | 2.845284 | 1.06E-08 | up | COG2226^Methyltransferase required for the conversion of demethylmenaquinone (DMKH2) to menaquinone (MKH2) | KEGG:ath:AT2G41040 | GO:0009507^cellular_component^chloroplast`GO:0010287^cellular_component^plastoglobule`GO:0008168^molecular_function^methyltransferase activity`GO:0080167^biological_process^response to karrikin | Y2104_ARATH^Y2104_ARATH^Q:1-789,H:96-352^63.81%ID^E:1e-119^RecName: Full=Uncharacterized methyltransferase At2g41040, chloroplastic;^Eukaryota; Viridiplantae; Streptophyta; Embryophyta; Tracheophyta; Spermatophyta; Magnoliophyta; eudicotyledons; Gunneridae; Pentapetalae; rosids; malvids; Brassicales; Brassicaceae; Camelineae; Arabidopsis |
| LOC109761795 | TRIAE_CS42_4BS_TGACv1_330186_AA1106430 | 211 | 171 | 107 | 27 | 20 | 21 | 2.817879 | 1.69E-09 | up | ENOG410XNXS^beta-amylase | KEGG:ath:AT4G00490`KO:K01177 | GO:0009507^cellular_component^chloroplast`GO:0009570^cellular_component^chloroplast stroma`GO:0102229^molecular_function^amylopectin maltohydrolase activity`GO:0016161^molecular_function^beta-amylase activity`GO:0000272^biological_process^polysaccharide catabolic process | BAM2_ARATH^BAM2_ARATH^Q:1-1191,H:134-530^77.58%ID^E:0^RecName: Full=Beta-amylase 2, chloroplastic;^Eukaryota; Viridiplantae; Streptophyta; Embryophyta; Tracheophyta; Spermatophyta; Magnoliophyta; eudicotyledons; Gunneridae; Pentapetalae; rosids; malvids; Brassicales; Brassicaceae; Camelineae; Arabidopsis |
| LOC109780993 | TRIAE_CS42_2BL_TGACv1_131418_AA0427470 | 26 | 40 | 48 | 6 | 6 | 6 | 2.623263 | 3.38E-06 | up | ENOG410Z7Z7^Pentatricopeptide repeat-containing protein | KEGG:ath:AT5G04780 | GO:0009507^cellular_component^chloroplast`GO:0043231^cellular_component^intracellular membrane-bounded organelle`GO:0005739^cellular_component^mitochondrion`GO:0004519^molecular_function^endonuclease activity`GO:0003723^molecular_function^RNA binding`GO:0008270^molecular_function^zinc ion binding`GO:0009451^biological_process^RNA modification | PP364_ARATH^PP364_ARATH^Q:334-894,H:71-257^31.02%ID^E:6e-26^RecName: Full=Pentatricopeptide repeat-containing protein At5g04780, mitochondrial;^Eukaryota; Viridiplantae; Streptophyta; Embryophyta; Tracheophyta; Spermatophyta; Magnoliophyta; eudicotyledons; Gunneridae; Pentapetalae; rosids; malvids; Brassicales; Brassicaceae; Camelineae; Arabidopsis |
| LOC109776543 | TRIAE_CS42_5BL_TGACv1_407510_AA1357570 | 772 | 920 | 1022 | 158 | 212 | 161 | 2.336378 | 9.29E-08 | up | ENOG410Y1GS^NA | KEGG:ath:AT4G02510 | GO:0009507^cellular_component^chloroplast`GO:0009941^cellular_component^chloroplast envelope`GO:0009707^cellular_component^chloroplast outer membrane`GO:0005829^cellular_component^cytosol`GO:0016021^cellular_component^integral component of membrane`GO:0016020^cellular_component^membrane`GO:0009536^cellular_component^plastid`GO:0051087^molecular_function^chaperone binding`GO:0004930^molecular_function^G-protein coupled receptor activity`GO:0005525^molecular_function^GTP binding`GO:0003924^molecular_function^GTPase activity`GO:0046872^molecular_function^metal ion binding`GO:0043024^molecular_function^ribosomal small subunit binding`GO:0004888^molecular_function^transmembrane signaling receptor activity`GO:0045037^biological_process^protein import into chloroplast stroma`GO:0045036^biological_process^protein targeting to chloroplast | TC159_ARATH^TC159_ARATH^Q:1462-3642,H:760-1495^47.12%ID^E:0^RecName: Full=Translocase of chloroplast 159, chloroplastic;^Eukaryota; Viridiplantae; Streptophyta; Embryophyta; Tracheophyta; Spermatophyta; Magnoliophyta; eudicotyledons; Gunneridae; Pentapetalae; rosids; malvids; Brassicales; Brassicaceae; Camelineae; Arabidopsis |
| LOC109746998 | TRIAE_CS42_3AL_TGACv1_196507_AA0660960 | 32 | 48 | 38 | 6 | 9 | 8 | 2.321794 | 2.77E-05 | up | ENOG410YJYE^Domain of unknown function (DUF3411) | KEGG:ath:AT5G12470 | GO:0009507^cellular_component^chloroplast`GO:0009941^cellular_component^chloroplast envelope`GO:0009706^cellular_component^chloroplast inner membrane`GO:0016021^cellular_component^integral component of membrane`GO:0005739^cellular_component^mitochondrion`GO:0009536^cellular_component^plastid`GO:0007275^biological_process^multicellular organism development | RER4_ARATH^RER4_ARATH^Q:376-756,H:160-301^36.55%ID^E:1e-10^RecName: Full=Protein RETICULATA-RELATED 4, chloroplastic {ECO:0000303\|PubMed:23596191};^Eukaryota; Viridiplantae; Streptophyta; Embryophyta; Tracheophyta; Spermatophyta; Magnoliophyta; eudicotyledons; Gunneridae; Pentapetalae; rosids; malvids; Brassicales; Brassicaceae; Camelineae; Arabidopsis |
| LOC109738799 | TRIAE_CS42_4DL_TGACv1_343749_AA1139150 | 2659 | 2816 | 2635 | 485 | 601 | 571 | 2.27225 | 1.64E-07 | up | COG4638^rieske 2fe-2S domain-containing protein | KEGG:ath:AT3G44880`KO:K13071 | GO:0009507^cellular_component^chloroplast`GO:0009941^cellular_component^chloroplast envelope`GO:0009706^cellular_component^chloroplast inner membrane`GO:0009534^cellular_component^chloroplast thylakoid`GO:0009535^cellular_component^chloroplast thylakoid membrane`GO:0051537^molecular_function^2 iron, 2 sulfur cluster binding`GO:0010277^molecular_function^chlorophyllide a oxygenase [overall] activity`GO:0051536^molecular_function^iron-sulfur cluster binding`GO:0046872^molecular_function^metal ion binding`GO:0032441^molecular_function^pheophorbide a oxygenase activity`GO:0008219^biological_process^cell death`GO:0015996^biological_process^chlorophyll catabolic process`GO:0009816^biological_process^defense response to bacterium, incompatible interaction`GO:0009908^biological_process^flower development`GO:0010154^biological_process^fruit development | PAO_ARATH^PAO_ARATH^Q:145-1605,H:48-537^72.65%ID^E:0^RecName: Full=Pheophorbide a oxygenase, chloroplastic;^Eukaryota; Viridiplantae; Streptophyta; Embryophyta; Tracheophyta; Spermatophyta; Magnoliophyta; eudicotyledons; Gunneridae; Pentapetalae; rosids; malvids; Brassicales; Brassicaceae; Camelineae; Arabidopsis |
| LOC109737821 | TRIAE_CS42_7BL_TGACv1_578732_AA1899320 | 223 | 285 | 194 | 37 | 61 | 50 | 2.221809 | 1.03E-06 | up | COG0694^metallo-sulfur cluster assembly | KEGG:ath:AT4G25910 | GO:0009507^cellular_component^chloroplast`GO:0009570^cellular_component^chloroplast stroma`GO:0005506^molecular_function^iron ion binding`GO:0051536^molecular_function^iron-sulfur cluster binding`GO:0032947^molecular_function^protein complex scaffold activity`GO:0005198^molecular_function^structural molecule activity`GO:0016226^biological_process^iron-sulfur cluster assembly`GO:0048564^biological_process^photosystem I assembly | NIFU3_ARATH^NIFU3_ARATH^Q:283-747,H:81-236^82.69%ID^E:1e-84^RecName: Full=NifU-like protein 3, chloroplastic;^Eukaryota; Viridiplantae; Streptophyta; Embryophyta; Tracheophyta; Spermatophyta; Magnoliophyta; eudicotyledons; Gunneridae; Pentapetalae; rosids; malvids; Brassicales; Brassicaceae; Camelineae; Arabidopsis |
| LOC109751445 | TRIAE_CS42_5DL_TGACv1_434857_AA1442640 | 141 | 137 | 132 | 18 | 36 | 33 | 2.210444 | 2.55E-06 | up | COG3670^dioxygenase | KEGG:osa:4333566`KO:K09840 | GO:0009507^cellular_component^chloroplast`GO:0045549^molecular_function^9-cis-epoxycarotenoid dioxygenase activity`GO:0046872^molecular_function^metal ion binding`GO:0009688^biological_process^abscisic acid biosynthetic process | NCED3_ORYSJ^NCED3_ORYSJ^Q:1-1785,H:1-608^83.58%ID^E:0^RecName: Full=9-cis-epoxycarotenoid dioxygenase NCED3, chloroplastic {ECO:0000305};^Eukaryota; Viridiplantae; Streptophyta; Embryophyta; Tracheophyta; Spermatophyta; Magnoliophyta; Liliopsida; Poales; Poaceae; BOP clade; Oryzoideae; Oryzeae; Oryzinae; Oryza; Oryza sativa |
| LOC109765435 | TRIAE_CS42_2AL_TGACv1_093773_AA0286430 | 1249 | 1653 | 1559 | 164 | 402 | 398 | 2.189376 | 5.25E-07 | up | ENOG410YGQV^harpin-induced protein 1 domain containing protein, expressed | KEGG:ath:AT2G35980 | GO:0046658^cellular_component^anchored component of plasma membrane`GO:0009507^cellular_component^chloroplast`GO:0016021^cellular_component^integral component of membrane`GO:0009506^cellular_component^plasmodesma`GO:0004871^molecular_function^signal transducer activity`GO:0051607^biological_process^defense response to virus`GO:0010150^biological_process^leaf senescence`GO:0051707^biological_process^response to other organism | NHL10_ARATH^NHL10_ARATH^Q:112-417,H:53-152^49.02%ID^E:9e-26^RecName: Full=NDR1/HIN1-like protein 10 {ECO:0000303\|PubMed:14666423};^Eukaryota; Viridiplantae; Streptophyta; Embryophyta; Tracheophyta; Spermatophyta; Magnoliophyta; eudicotyledons; Gunneridae; Pentapetalae; rosids; malvids; Brassicales; Brassicaceae; Camelineae; Arabidopsis |
| LOC109765911 | TRIAE_CS42_U_TGACv1_640731_AA2071450 | 300 | 254 | 246 | 53 | 53 | 69 | 2.171017 | 1.59E-06 | up | COG0473^3-isopropylmalate dehydrogenase activity | KEGG:ath:AT1G80560`KO:K00052 | GO:0009507^cellular_component^chloroplast`GO:0009941^cellular_component^chloroplast envelope`GO:0009570^cellular_component^chloroplast stroma`GO:0009536^cellular_component^plastid`GO:0003862^molecular_function^3-isopropylmalate dehydrogenase activity`GO:0000287^molecular_function^magnesium ion binding`GO:0051287^molecular_function^NAD binding`GO:0009098^biological_process^leucine biosynthetic process | LEU32_ARATH^LEU32_ARATH^Q:94-1194,H:31-400^81.62%ID^E:0^RecName: Full=3-isopropylmalate dehydrogenase 2, chloroplastic;^Eukaryota; Viridiplantae; Streptophyta; Embryophyta; Tracheophyta; Spermatophyta; Magnoliophyta; eudicotyledons; Gunneridae; Pentapetalae; rosids; malvids; Brassicales; Brassicaceae; Camelineae; Arabidopsis |
| LOC109736812 | TRIAE_CS42_2BL_TGACv1_129991_AA0401180 | 193 | 195 | 174 | 38 | 42 | 44 | 2.158047 | 2.77E-06 | up | COG1324^tolerance protein | . | GO:0009507^cellular_component^chloroplast`GO:0005507^molecular_function^copper ion binding`GO:0070207^biological_process^protein homotrimerization`GO:0010038^biological_process^response to metal ion | CUTA1_ORYSJ^CUTA1_ORYSJ^Q:193-531,H:65-177^93.81%ID^E:7e-71^RecName: Full=Protein CutA 1, chloroplastic;^Eukaryota; Viridiplantae; Streptophyta; Embryophyta; Tracheophyta; Spermatophyta; Magnoliophyta; Liliopsida; Poales; Poaceae; BOP clade; Oryzoideae; Oryzeae; Oryzinae; Oryza; Oryza sativa |
| LOC109734994 | TRIAE_CS42_5BS_TGACv1_424281_AA1388450 | 42 | 56 | 91 | 11 | 16 | 20 | 1.985981 | 9.90E-05 | up | ENOG410YP72^VQ motif | KEGG:ath:AT1G68450 | GO:0009507^cellular_component^chloroplast | VQ8_ARATH^VQ8_ARATH^Q:61-285,H:11-71^41.03%ID^E:1e-05^RecName: Full=VQ motif-containing protein 8, chloroplastic {ECO:0000303\|PubMed:22535423};^Eukaryota; Viridiplantae; Streptophyta; Embryophyta; Tracheophyta; Spermatophyta; Magnoliophyta; eudicotyledons; Gunneridae; Pentapetalae; rosids; malvids; Brassicales; Brassicaceae; Camelineae; Arabidopsis |
| *WRKY19-b* | TRIAE_CS42_1DL_TGACv1_061663_AA0201260 | 502 | 628 | 287 | 85 | 140 | 128 | 1.979544 | 9.34E-06 | up | ENOG410YZU1^Transcription factor | KEGG:ath:AT4G23810 | GO:0009507^cellular_component^chloroplast`GO:0005634^cellular_component^nucleus`GO:0003677^molecular_function^DNA binding`GO:0043565^molecular_function^sequence-specific DNA binding`GO:0003700^molecular_function^transcription factor activity, sequence-specific DNA binding`GO:0009816^biological_process^defense response to bacterium, incompatible interaction`GO:0010150^biological_process^leaf senescence`GO:0045893^biological_process^positive regulation of transcription, DNA-templated`GO:0031347^biological_process^regulation of defense response`GO:0010200^biological_process^response to chitin`GO:0042542^biological_process^response to hydrogen peroxide`GO:0010193^biological_process^response to ozone`GO:0009751^biological_process^response to salicylic acid`GO:0006351^biological_process^transcription, DNA-templated | WRK53_ARATH^WRK53_ARATH^Q:295-522,H:155-233^60.76%ID^E:7e-26^RecName: Full=Probable WRKY transcription factor 53;^Eukaryota; Viridiplantae; Streptophyta; Embryophyta; Tracheophyta; Spermatophyta; Magnoliophyta; eudicotyledons; Gunneridae; Pentapetalae; rosids; malvids; Brassicales; Brassicaceae; Camelineae; Arabidopsis |
| LOC109767051 | TRIAE_CS42_6BS_TGACv1_513993_AA1653660 | 261 | 262 | 125 | 51 | 61 | 50 | 1.973714 | 1.70E-05 | up | COG0204^Acyltransferase | KEGG:ath:AT1G54570 | GO:0009507^cellular_component^chloroplast`GO:0010287^cellular_component^plastoglobule`GO:0004144^molecular_function^diacylglycerol O-acyltransferase activity`GO:0033306^biological_process^phytol metabolic process`GO:0019432^biological_process^triglyceride biosynthetic process | Y1457_ARATH^Y1457_ARATH^Q:127-2061,H:61-704^51.16%ID^E:0^RecName: Full=Acyltransferase-like protein At1g54570, chloroplastic;^Eukaryota; Viridiplantae; Streptophyta; Embryophyta; Tracheophyta; Spermatophyta; Magnoliophyta; eudicotyledons; Gunneridae; Pentapetalae; rosids; malvids; Brassicales; Brassicaceae; Camelineae; Arabidopsis |
| LOC109765296 | TRIAE_CS42_U_TGACv1_644623_AA2140860 | 143 | 135 | 315 | 38 | 83 | 30 | 1.9568 | 2.20E-05 | up | ENOG410YDA6^synthase | KEGG:osa:4328124`KO:K15086 | GO:0009507^cellular_component^chloroplast`GO:0000287^molecular_function^magnesium ion binding`GO:0034007^molecular_function^S-linalool synthase activity`GO:0010333^molecular_function^terpene synthase activity`GO:0042742^biological_process^defense response to bacterium`GO:0043693^biological_process^monoterpene biosynthetic process`GO:0016114^biological_process^terpenoid biosynthetic process | LINS_ORYSJ^LINS_ORYSJ^Q:112-1650,H:51-595^64.04%ID^E:0^RecName: Full=S-(+)-linalool synthase, chloroplastic {ECO:0000305};^Eukaryota; Viridiplantae; Streptophyta; Embryophyta; Tracheophyta; Spermatophyta; Magnoliophyta; Liliopsida; Poales; Poaceae; BOP clade; Oryzoideae; Oryzeae; Oryzinae; Oryza; Oryza sativa |
| LOC100846290 | TRIAE_CS42_3DS_TGACv1_273247_AA0929860 | 338 | 374 | 339 | 76 | 125 | 87 | 1.846126 | 4.61E-05 | up | COG0652^peptidyl-prolyl cis-trans isomerase activity | KEGG:ath:AT1G74070`KO:K03768 | GO:0009507^cellular_component^chloroplast`GO:0009534^cellular_component^chloroplast thylakoid`GO:0003755^molecular_function^peptidyl-prolyl cis-trans isomerase activity`GO:0006457^biological_process^protein folding | CP26B_ARATH^CP26B_ARATH^Q:211-882,H:85-313^56.71%ID^E:3e-67^RecName: Full=Peptidyl-prolyl cis-trans isomerase CYP26-2, chloroplastic;^Eukaryota; Viridiplantae; Streptophyta; Embryophyta; Tracheophyta; Spermatophyta; Magnoliophyta; eudicotyledons; Gunneridae; Pentapetalae; rosids; malvids; Brassicales; Brassicaceae; Camelineae; Arabidopsis |
| LOC109780946 | TRIAE_CS42_5DL_TGACv1_433895_AA1424750 | 28 | 33 | 48 | 11 | 12 | 7 | 1.834234 | 0.001076 | up | COG0465^Acts as a processive, ATP-dependent zinc metallopeptidase for both cytoplasmic and membrane proteins. Plays a role in the quality control of integral membrane proteins (By similarity) | KEGG:ath:AT2G26140 | GO:0009941^cellular_component^chloroplast envelope`GO:0016021^cellular_component^integral component of membrane`GO:0016020^cellular_component^membrane`GO:0005743^cellular_component^mitochondrial inner membrane`GO:0005739^cellular_component^mitochondrion`GO:0009536^cellular_component^plastid`GO:0005524^molecular_function^ATP binding`GO:0004176^molecular_function^ATP-dependent peptidase activity`GO:0046872^molecular_function^metal ion binding`GO:0004222^molecular_function^metalloendopeptidase activity`GO:0008237^molecular_function^metallopeptidase activity`GO:0010073^biological_process^meristem maintenance`GO:0006508^biological_process^proteolysis | FTSH4_ARATH^FTSH4_ARATH^Q:115-1695,H:130-656^62.45%ID^E:0^RecName: Full=ATP-dependent zinc metalloprotease FTSH 4, mitochondrial;^Eukaryota; Viridiplantae; Streptophyta; Embryophyta; Tracheophyta; Spermatophyta; Magnoliophyta; eudicotyledons; Gunneridae; Pentapetalae; rosids; malvids; Brassicales; Brassicaceae; Camelineae; Arabidopsis |
| LOC109748375 | TRIAE_CS42_6BL_TGACv1_500072_AA1598170 | 306 | 358 | 408 | 174 | 65 | 64 | 1.814162 | 6.35E-05 | up | COG5059^Kinesin family member | KEGG:osa:4330897`KO:K11498 | GO:0009507^cellular_component^chloroplast`GO:0005871^cellular_component^kinesin complex`GO:0005874^cellular_component^microtubule`GO:0043531^molecular_function^ADP binding`GO:0005524^molecular_function^ATP binding`GO:0016887^molecular_function^ATPase activity`GO:0000287^molecular_function^magnesium ion binding`GO:0008017^molecular_function^microtubule binding`GO:0003777^molecular_function^microtubule motor activity`GO:0042803^molecular_function^protein homodimerization activity`GO:0007018^biological_process^microtubule-based movement | KN7D_ORYSJ^KN7D_ORYSJ^Q:1-2541,H:152-1007^83.47%ID^E:0^RecName: Full=Kinesin-like protein KIN-7D, chloroplastic {ECO:0000305};^Eukaryota; Viridiplantae; Streptophyta; Embryophyta; Tracheophyta; Spermatophyta; Magnoliophyta; Liliopsida; Poales; Poaceae; BOP clade; Oryzoideae; Oryzeae; Oryzinae; Oryza; Oryza sativa |
| LOC109762109 | TRIAE_CS42_1AL_TGACv1_000466_AA0012780 | 219 | 348 | 306 | 49 | 86 | 129 | 1.706204 | 0.000207 | up | ENOG410ZB51^Rubisco LSMT substrate-binding | KEGG:ath:AT4G20130 | GO:0009507^cellular_component^chloroplast`GO:0009534^cellular_component^chloroplast thylakoid`GO:0009295^cellular_component^nucleoid`GO:0009508^cellular_component^plastid chromosome`GO:0000427^cellular_component^plastid-encoded plastid RNA polymerase complex`GO:0016279^molecular_function^protein-lysine N-methyltransferase activity`GO:0009658^biological_process^chloroplast organization`GO:0018026^biological_process^peptidyl-lysine monomethylation`GO:0006355^biological_process^regulation of transcription, DNA-templated`GO:0009416^biological_process^response to light stimulus`GO:0010027^biological_process^thylakoid membrane organization`GO:0042793^biological_process^transcription from plastid promoter | PTA14_ARATH^PTA14_ARATH^Q:265-1479,H:79-483^63.3%ID^E:0^RecName: Full=Protein PLASTID TRANSCRIPTIONALLY ACTIVE 14 {ECO:0000303\|PubMed:16326926};^Eukaryota; Viridiplantae; Streptophyta; Embryophyta; Tracheophyta; Spermatophyta; Magnoliophyta; eudicotyledons; Gunneridae; Pentapetalae; rosids; malvids; Brassicales; Brassicaceae; Camelineae; Arabidopsis |
| LOC109770990 | TRIAE_CS42_6DS_TGACv1_542491_AA1722230 | 67 | 51 | 58 | 15 | 23 | 15 | 1.70395 | 0.001059 | up | ENOG410XT49^mitochondrial transcription termination | KEGG:ath:AT5G54180 | GO:0009507^cellular_component^chloroplast`GO:0009295^cellular_component^nucleoid`GO:0009508^cellular_component^plastid chromosome`GO:0003690^molecular_function^double-stranded DNA binding`GO:0019843^molecular_function^rRNA binding`GO:0003727^molecular_function^single-stranded RNA binding`GO:0009658^biological_process^chloroplast organization`GO:0032502^biological_process^developmental process`GO:0006353^biological_process^DNA-templated transcription, termination`GO:0006355^biological_process^regulation of transcription, DNA-templated`GO:0042255^biological_process^ribosome assembly`GO:0008380^biological_process^RNA splicing | MTEF8_ARATH^MTEF8_ARATH^Q:280-1065,H:233-488^25.19%ID^E:2e-08^RecName: Full=Transcription termination factor MTERF8, chloroplastic {ECO:0000305};^Eukaryota; Viridiplantae; Streptophyta; Embryophyta; Tracheophyta; Spermatophyta; Magnoliophyta; eudicotyledons; Gunneridae; Pentapetalae; rosids; malvids; Brassicales; Brassicaceae; Camelineae; Arabidopsis |
| *NCED2D* | TRIAE_CS42_5DL_TGACv1_434857_AA1442630 | 175 | 144 | 130 | 28 | 56 | 63 | 1.5851 | 0.000948 | up | COG3670^dioxygenase | KEGG:osa:4333566`KO:K09840 | GO:0009507^cellular_component^chloroplast`GO:0045549^molecular_function^9-cis-epoxycarotenoid dioxygenase activity`GO:0046872^molecular_function^metal ion binding`GO:0009688^biological_process^abscisic acid biosynthetic process | NCED3_ORYSJ^NCED3_ORYSJ^Q:1899-118,H:1-607^83.22%ID^E:0^RecName: Full=9-cis-epoxycarotenoid dioxygenase NCED3, chloroplastic {ECO:0000305};^Eukaryota; Viridiplantae; Streptophyta; Embryophyta; Tracheophyta; Spermatophyta; Magnoliophyta; Liliopsida; Poales; Poaceae; BOP clade; Oryzoideae; Oryzeae; Oryzinae; Oryza; Oryza sativa |
| LOC109746808 | TRIAE_CS42_7AL_TGACv1_556007_AA1752420 | 180 | 152 | 111 | 25 | 70 | 52 | 1.562609 | 0.001161 | up | COG0484^ATP binding to DnaK triggers the release of the substrate protein, thus completing the reaction cycle. Several rounds of ATP-dependent interactions between DnaJ, DnaK and GrpE are required for fully efficient folding. Also involved, together with DnaK and GrpE, in the DNA replication of plasmids through activation of initiation proteins (By similarity) | KEGG:ath:AT1G80920 | GO:0009507^cellular_component^chloroplast`GO:0009570^cellular_component^chloroplast stroma`GO:0005634^cellular_component^nucleus`GO:0009416^biological_process^response to light stimulus | DNAJ8_ARATH^DNAJ8_ARATH^Q:157-345,H:55-111^38.1%ID^E:6e-08^RecName: Full=Chaperone protein dnaJ 8, chloroplastic;^Eukaryota; Viridiplantae; Streptophyta; Embryophyta; Tracheophyta; Spermatophyta; Magnoliophyta; eudicotyledons; Gunneridae; Pentapetalae; rosids; malvids; Brassicales; Brassicaceae; Camelineae; Arabidopsis |
| LOC109743853 | TRIAE_CS42_3AL_TGACv1_194305_AA0630850 | 111 | 69 | 58 | 20 | 39 | 23 | 1.507029 | 0.003122 | up | ENOG410YGQV^harpin-induced protein 1 domain containing protein, expressed | KEGG:ath:AT2G35980 | GO:0046658^cellular_component^anchored component of plasma membrane`GO:0009507^cellular_component^chloroplast`GO:0016021^cellular_component^integral component of membrane`GO:0009506^cellular_component^plasmodesma`GO:0004871^molecular_function^signal transducer activity`GO:0051607^biological_process^defense response to virus`GO:0010150^biological_process^leaf senescence`GO:0051707^biological_process^response to other organism | NHL10_ARATH^NHL10_ARATH^Q:325-837,H:36-206^37.14%ID^E:3e-16^RecName: Full=NDR1/HIN1-like protein 10 {ECO:0000303\|PubMed:14666423};^Eukaryota; Viridiplantae; Streptophyta; Embryophyta; Tracheophyta; Spermatophyta; Magnoliophyta; eudicotyledons; Gunneridae; Pentapetalae; rosids; malvids; Brassicales; Brassicaceae; Camelineae; Arabidopsis |
| LOC100821676 | TRIAE_CS42_2AL_TGACv1_094415_AA0297280 | 507 | 506 | 477 | 125 | 188 | 211 | 1.486754 | 0.001339 | up | COG0534^Mate efflux family protein | KEGG:ath:AT2G32230`KO:K18213 | GO:0009507^cellular_component^chloroplast`GO:0005739^cellular_component^mitochondrion`GO:0004526^molecular_function^ribonuclease P activity`GO:0001682^biological_process^tRNA 5'-leader removal`GO:0008033^biological_process^tRNA processing | PRRP1_ARATH^PRRP1_ARATH^Q:265-1680,H:94-568^59.54%ID^E:4e-171^RecName: Full=Proteinaceous RNase P 1, chloroplastic/mitochondrial;^Eukaryota; Viridiplantae; Streptophyta; Embryophyta; Tracheophyta; Spermatophyta; Magnoliophyta; eudicotyledons; Gunneridae; Pentapetalae; rosids; malvids; Brassicales; Brassicaceae; Camelineae; Arabidopsis |
| LOC109735302 | TRIAE_CS42_2DS_TGACv1_178116_AA0590920 | 240 | 224 | 425 | 50 | 145 | 119 | 1.483045 | 0.001599 | up | ENOG410Z7Z7^Pentatricopeptide repeat-containing protein | KEGG:ath:AT4G37380 | GO:0009507^cellular_component^chloroplast`GO:0043231^cellular_component^intracellular membrane-bounded organelle`GO:0004519^molecular_function^endonuclease activity`GO:0003723^molecular_function^RNA binding`GO:0008270^molecular_function^zinc ion binding`GO:1900865^biological_process^chloroplast RNA modification`GO:0006397^biological_process^mRNA processing`GO:0009451^biological_process^RNA modification | PP354_ARATH^PP354_ARATH^Q:418-1815,H:160-627^46.58%ID^E:8e-151^RecName: Full=Pentatricopeptide repeat-containing protein ELI1, chloroplastic {ECO:0000305};^Eukaryota; Viridiplantae; Streptophyta; Embryophyta; Tracheophyta; Spermatophyta; Magnoliophyta; eudicotyledons; Gunneridae; Pentapetalae; rosids; malvids; Brassicales; Brassicaceae; Camelineae; Arabidopsis |
| LOC109772726 | TRIAE_CS42_7DS_TGACv1_623759_AA2055930 | 703 | 617 | 770 | 158 | 277 | 317 | 1.454614 | 0.001671 | up | ENOG410YZU1^Transcription factor | KEGG:ath:AT4G23810 | GO:0009507^cellular_component^chloroplast`GO:0005634^cellular_component^nucleus`GO:0003677^molecular_function^DNA binding`GO:0043565^molecular_function^sequence-specific DNA binding`GO:0003700^molecular_function^transcription factor activity, sequence-specific DNA binding`GO:0009816^biological_process^defense response to bacterium, incompatible interaction`GO:0010150^biological_process^leaf senescence`GO:0045893^biological_process^positive regulation of transcription, DNA-templated`GO:0031347^biological_process^regulation of defense response`GO:0010200^biological_process^response to chitin`GO:0042542^biological_process^response to hydrogen peroxide`GO:0010193^biological_process^response to ozone`GO:0009751^biological_process^response to salicylic acid`GO:0006351^biological_process^transcription, DNA-templated | WRK53_ARATH^WRK53_ARATH^Q:334-762,H:132-284^50%ID^E:6e-35^RecName: Full=Probable WRKY transcription factor 53;^Eukaryota; Viridiplantae; Streptophyta; Embryophyta; Tracheophyta; Spermatophyta; Magnoliophyta; eudicotyledons; Gunneridae; Pentapetalae; rosids; malvids; Brassicales; Brassicaceae; Camelineae; Arabidopsis |
| LOC109764841 | TRIAE_CS42_3AL_TGACv1_195655_AA0652390 | 85 | 81 | 148 | 24 | 42 | 53 | 1.382002 | 0.006186 | up | ENOG4111T2I^PsbP | KEGG:ath:AT3G05410 | GO:0009507^cellular_component^chloroplast`GO:0019898^cellular_component^extrinsic component of membrane`GO:0009654^cellular_component^photosystem II oxygen evolving complex`GO:0005509^molecular_function^calcium ion binding`GO:0015979^biological_process^photosynthesis | PPD7_ARATH^PPD7_ARATH^Q:1-597,H:87-279^54.27%ID^E:1e-72^RecName: Full=PsbP domain-containing protein 7, chloroplastic;^Eukaryota; Viridiplantae; Streptophyta; Embryophyta; Tracheophyta; Spermatophyta; Magnoliophyta; eudicotyledons; Gunneridae; Pentapetalae; rosids; malvids; Brassicales; Brassicaceae; Camelineae; Arabidopsis |
| LOC109745942 | TRIAE_CS42_4AL_TGACv1_289623_AA0974220 | 1819 | 2333 | 2550 | 515 | 1065 | 1039 | 1.336849 | 0.00419 | up | ENOG410XPWC^solute carrier family 17 | KEGG:osa:4347913 | GO:0009706^cellular_component^chloroplast inner membrane`GO:0016021^cellular_component^integral component of membrane`GO:0009536^cellular_component^plastid`GO:0005315^molecular_function^inorganic phosphate transmembrane transporter activity`GO:0015229^molecular_function^L-ascorbic acid transporter activity`GO:0098656^biological_process^anion transmembrane transport`GO:0010028^biological_process^xanthophyll cycle | PHT44_ORYSJ^PHT44_ORYSJ^Q:208-1830,H:55-591^81.97%ID^E:0^RecName: Full=Probable anion transporter 4, chloroplastic;^Eukaryota; Viridiplantae; Streptophyta; Embryophyta; Tracheophyta; Spermatophyta; Magnoliophyta; Liliopsida; Poales; Poaceae; BOP clade; Oryzoideae; Oryzeae; Oryzinae; Oryza; Oryza sativa |
| LOC109741864 | TRIAE_CS42_6AL_TGACv1_473688_AA1532460 | 449 | 609 | 536 | 148 | 227 | 268 | 1.290815 | 0.006885 | up | ENOG4111RPC^galactolipase activity | KEGG:ath:AT4G16820 | GO:0009507^cellular_component^chloroplast`GO:0047714^molecular_function^galactolipase activity`GO:0008970^molecular_function^phosphatidylcholine 1-acylhydrolase activity`GO:0004806^molecular_function^triglyceride lipase activity`GO:0016042^biological_process^lipid catabolic process | PLA14_ARATH^PLA14_ARATH^Q:184-1293,H:43-470^43.76%ID^E:5e-86^RecName: Full=Phospholipase A1-Ibeta2, chloroplastic;^Eukaryota; Viridiplantae; Streptophyta; Embryophyta; Tracheophyta; Spermatophyta; Magnoliophyta; eudicotyledons; Gunneridae; Pentapetalae; rosids; malvids; Brassicales; Brassicaceae; Camelineae; Arabidopsis |
| LOC109746463 | TRIAE_CS42_4BL_TGACv1_321153_AA1056280 | 372 | 334 | 210 | 1012 | 596 | 551 | -1.25418 | 0.008852 | down | COG2214^DNAj domain protein | KEGG:ath:AT4G36040 | GO:0009507^cellular_component^chloroplast`GO:0009570^cellular_component^chloroplast stroma`GO:0005634^cellular_component^nucleus`GO:0009536^cellular_component^plastid | DNJ11_ARATH^DNJ11_ARATH^Q:202-492,H:66-161^50.49%ID^E:3e-23^RecName: Full=Chaperone protein dnaJ 11, chloroplastic;^Eukaryota; Viridiplantae; Streptophyta; Embryophyta; Tracheophyta; Spermatophyta; Magnoliophyta; eudicotyledons; Gunneridae; Pentapetalae; rosids; malvids; Brassicales; Brassicaceae; Camelineae; Arabidopsis |
| *rpl22* | AIG90467 | 588 | 1229 | 1288 | 2889 | 2729 | 2332 | -1.36672 | 0.003228 | down | . | . | GO:0009507^cellular_component^chloroplast`GO:0015934^cellular_component^large ribosomal subunit`GO:0019843^molecular_function^rRNA binding`GO:0003735^molecular_function^structural constituent of ribosome`GO:0006412^biological_process^translation | RK22_WHEAT^RK22_WHEAT^Q:1-444,H:1-148^100%ID^E:4e-103^RecName: Full=50S ribosomal protein L22, chloroplastic;^Eukaryota; Viridiplantae; Streptophyta; Embryophyta; Tracheophyta; Spermatophyta; Magnoliophyta; Liliopsida; Poales; Poaceae; BOP clade; Pooideae; Triticodae; Triticeae; Triticinae; Triticum |
| LOC109736138 | TRIAE_CS42_5BL_TGACv1_407613_AA1358380 | 35 | 42 | 37 | 114 | 111 | 74 | -1.40413 | 0.005373 | down | COG1022^Amp-dependent synthetase and ligase | KEGG:ath:AT3G23790`KO:K01897 | GO:0009941^cellular_component^chloroplast envelope`GO:0016874^molecular_function^ligase activity`GO:0006631^biological_process^fatty acid metabolic process | AAE16_ARATH^AAE16_ARATH^Q:205-2232,H:50-722^65.93%ID^E:0^RecName: Full=Probable acyl-activating enzyme 16, chloroplastic;^Eukaryota; Viridiplantae; Streptophyta; Embryophyta; Tracheophyta; Spermatophyta; Magnoliophyta; eudicotyledons; Gunneridae; Pentapetalae; rosids; malvids; Brassicales; Brassicaceae; Camelineae; Arabidopsis |
| *rps8* | AIG90463 | 1897 | 3743 | 4050 | 8222 | 9299 | 8120 | -1.41571 | 0.002054 | down | . | . | GO:0009507^cellular_component^chloroplast`GO:0005840^cellular_component^ribosome`GO:0019843^molecular_function^rRNA binding`GO:0003735^molecular_function^structural constituent of ribosome`GO:0006412^biological_process^translation | RR8_WHEAT^RR8_WHEAT^Q:1-408,H:1-136^100%ID^E:8e-94^RecName: Full=30S ribosomal protein S8, chloroplastic;^Eukaryota; Viridiplantae; Streptophyta; Embryophyta; Tracheophyta; Spermatophyta; Magnoliophyta; Liliopsida; Poales; Poaceae; BOP clade; Pooideae; Triticodae; Triticeae; Triticinae; Triticum |
| *rsp19* | AIG90468 | 32 | 38 | 66 | 149 | 115 | 98 | -1.41909 | 0.0042 | down | . | . | GO:0009507^cellular_component^chloroplast`GO:0015935^cellular_component^small ribosomal subunit`GO:0019843^molecular_function^rRNA binding`GO:0003735^molecular_function^structural constituent of ribosome`GO:0006412^biological_process^translation | RR19_WHEAT^RR19_WHEAT^Q:1-279,H:1-93^98.92%ID^E:5e-62^RecName: Full=30S ribosomal protein S19, chloroplastic;^Eukaryota; Viridiplantae; Streptophyta; Embryophyta; Tracheophyta; Spermatophyta; Magnoliophyta; Liliopsida; Poales; Poaceae; BOP clade; Pooideae; Triticodae; Triticeae; Triticinae; Triticum |
| LOC109763736 | TRIAE_CS42_6BS_TGACv1_515797_AA1672390 | 131 | 128 | 86 | 329 | 272 | 312 | -1.42219 | 0.002649 | down | . | . | GO:0009507^cellular_component^chloroplast`GO:0005524^molecular_function^ATP binding`GO:0043295^molecular_function^glutathione binding`GO:0004363^molecular_function^glutathione synthase activity`GO:0000287^molecular_function^magnesium ion binding`GO:0042803^molecular_function^protein homodimerization activity | GSHB_BRAJU^GSHB_BRAJU^Q:223-1575,H:80-530^69.62%ID^E:0^RecName: Full=Glutathione synthetase, chloroplastic;^Eukaryota; Viridiplantae; Streptophyta; Embryophyta; Tracheophyta; Spermatophyta; Magnoliophyta; eudicotyledons; Gunneridae; Pentapetalae; rosids; malvids; Brassicales; Brassicaceae; Brassiceae; Brassica |
| *Wcab* | TRIAE_CS42_6AS_TGACv1_485824_AA1552860 | 492 | 238 | 260 | 951 | 1243 | 424 | -1.42718 | 0.002061 | down | ENOG410ZHBU^Chlorophyll A-B binding protein | KEGG:sly:108491835`KO:K08912 | GO:0009941^cellular_component^chloroplast envelope`GO:0009535^cellular_component^chloroplast thylakoid membrane`GO:0016021^cellular_component^integral component of membrane`GO:0009522^cellular_component^photosystem I`GO:0009523^cellular_component^photosystem II`GO:0010287^cellular_component^plastoglobule`GO:0016168^molecular_function^chlorophyll binding`GO:0046872^molecular_function^metal ion binding`GO:0031409^molecular_function^pigment binding`GO:0009768^biological_process^photosynthesis, light harvesting in photosystem I`GO:0018298^biological_process^protein-chromophore linkage`GO:0009416^biological_process^response to light stimulus | CB2G_SOLLC^CB2G_SOLLC^Q:1-657,H:1-267^76.03%ID^E:8e-131^RecName: Full=Chlorophyll a-b binding protein 3C, chloroplastic;^Eukaryota; Viridiplantae; Streptophyta; Embryophyta; Tracheophyta; Spermatophyta; Magnoliophyta; eudicotyledons; Gunneridae; Pentapetalae; asterids; lamiids; Solanales; Solanaceae; Solanoideae; Solaneae; Solanum; Lycopersicon |
| LOC109775877 | TRIAE_CS42_5DS_TGACv1_460621_AA1495590 | 46 | 37 | 22 | 79 | 103 | 97 | -1.42998 | 0.004659 | down | . | KEGG:ag:BAM20978`KO:K20506 | GO:0009507^cellular_component^chloroplast`GO:0016787^molecular_function^hydrolase activity`GO:0016829^molecular_function^lyase activity`GO:0006952^biological_process^defense response`GO:0008152^biological_process^metabolic process | TCEA1_TULGE^TCEA1_TULGE^Q:13-927,H:77-383^42.95%ID^E:3e-72^RecName: Full=Tuliposide A-converting enzyme 1, chloroplastic;^Eukaryota; Viridiplantae; Streptophyta; Embryophyta; Tracheophyta; Spermatophyta; Magnoliophyta; Liliopsida; Liliales; Liliaceae; Tulipa |
| LOC109785379 | TRIAE_CS42_U_TGACv1_641871_AA2106210 | 49 | 28 | 24 | 133 | 59 | 79 | -1.43738 | 0.004532 | down | COG0706^Required for the insertion and or proper folding and or complex formation of integral membrane proteins into the membrane. Involved in integration of membrane proteins that insert both dependently and independently of the Sec translocase complex, as well as at least some lipoproteins | KEGG:ath:AT1G24490`KO:K03217 | GO:0009507^cellular_component^chloroplast`GO:0009535^cellular_component^chloroplast thylakoid membrane`GO:0016021^cellular_component^integral component of membrane`GO:0009579^cellular_component^thylakoid`GO:0009658^biological_process^chloroplast organization`GO:0051205^biological_process^protein insertion into membrane`GO:0072598^biological_process^protein localization to chloroplast | ALB31_ARATH^ALB31_ARATH^Q:16-1047,H:146-493^56.29%ID^E:1e-118^RecName: Full=ALBINO3-like protein 1, chloroplastic;^Eukaryota; Viridiplantae; Streptophyta; Embryophyta; Tracheophyta; Spermatophyta; Magnoliophyta; eudicotyledons; Gunneridae; Pentapetalae; rosids; malvids; Brassicales; Brassicaceae; Camelineae; Arabidopsis |
| LOC109746093 | TRIAE_CS42_7AL_TGACv1_558526_AA1794040 | 767 | 497 | 303 | 1623 | 1618 | 978 | -1.45317 | 0.00156 | down | COG2214^DNAj domain protein | KEGG:ath:AT4G36040 | GO:0009507^cellular_component^chloroplast`GO:0009570^cellular_component^chloroplast stroma`GO:0005634^cellular_component^nucleus`GO:0009536^cellular_component^plastid | DNJ11_ARATH^DNJ11_ARATH^Q:142-420,H:67-161^46%ID^E:1e-18^RecName: Full=Chaperone protein dnaJ 11, chloroplastic;^Eukaryota; Viridiplantae; Streptophyta; Embryophyta; Tracheophyta; Spermatophyta; Magnoliophyta; eudicotyledons; Gunneridae; Pentapetalae; rosids; malvids; Brassicales; Brassicaceae; Camelineae; Arabidopsis |
| *rps2* | AIG90421 | 137 | 182 | 153 | 431 | 415 | 435 | -1.45568 | 0.001813 | down | . | . | GO:0009507^cellular_component^chloroplast`GO:0015935^cellular_component^small ribosomal subunit`GO:0003735^molecular_function^structural constituent of ribosome`GO:0006412^biological_process^translation | RR2_WHEAT^RR2_WHEAT^Q:1-708,H:1-236^100%ID^E:7e-176^RecName: Full=30S ribosomal protein S2, chloroplastic;^Eukaryota; Viridiplantae; Streptophyta; Embryophyta; Tracheophyta; Spermatophyta; Magnoliophyta; Liliopsida; Poales; Poaceae; BOP clade; Pooideae; Triticodae; Triticeae; Triticinae; Triticum |
| *rps11* | TRIAE_CS42_6AS_TGACv1_485972_AA1555000 | 1100 | 2294 | 2266 | 5198 | 5536 | 4705 | -1.45953 | 0.001392 | down | . | . | GO:0009507^cellular_component^chloroplast`GO:0005840^cellular_component^ribosome`GO:0019843^molecular_function^rRNA binding`GO:0003735^molecular_function^structural constituent of ribosome`GO:0006412^biological_process^translation | RR11_WHEAT^RR11_WHEAT^Q:1-429,H:1-143^94.41%ID^E:1e-92^RecName: Full=30S ribosomal protein S11, chloroplastic {ECO:0000255\|HAMAP-Rule:MF_01310};^Eukaryota; Viridiplantae; Streptophyta; Embryophyta; Tracheophyta; Spermatophyta; Magnoliophyta; Liliopsida; Poales; Poaceae; BOP clade; Pooideae; Triticodae; Triticeae; Triticinae; Triticum |
| *rpoC1* | AIG90419 | 142 | 86 | 120 | 236 | 354 | 360 | -1.46894 | 0.001754 | down | . | . | GO:0009507^cellular_component^chloroplast`GO:0003677^molecular_function^DNA binding`GO:0003899^molecular_function^DNA-directed 5'-3' RNA polymerase activity`GO:0006351^biological_process^transcription, DNA-templated | RPOC1_WHEAT^RPOC1_WHEAT^Q:1-2049,H:1-683^100%ID^E:0^RecName: Full=DNA-directed RNA polymerase subunit beta' {ECO:0000255\|HAMAP-Rule:MF_01323};^Eukaryota; Viridiplantae; Streptophyta; Embryophyta; Tracheophyta; Spermatophyta; Magnoliophyta; Liliopsida; Poales; Poaceae; BOP clade; Pooideae; Triticodae; Triticeae; Triticinae; Triticum |
| LOC109753858 | TRIAE_CS42_1AS_TGACv1_019934_AA0073050 | 64 | 60 | 90 | 153 | 184 | 250 | -1.46952 | 0.002124 | down | COG1226^PotAssium voltage-gated channel | KEGG:ath:AT5G43745 | GO:0009507^cellular_component^chloroplast`GO:0009941^cellular_component^chloroplast envelope`GO:0016021^cellular_component^integral component of membrane`GO:0008324^molecular_function^cation transmembrane transporter activity`GO:0006813^biological_process^potassium ion transport | POLL2_ARATH^POLL2_ARATH^Q:322-2493,H:104-817^60.66%ID^E:0^RecName: Full=Putative ion channel POLLUX-like 2;^Eukaryota; Viridiplantae; Streptophyta; Embryophyta; Tracheophyta; Spermatophyta; Magnoliophyta; eudicotyledons; Gunneridae; Pentapetalae; rosids; malvids; Brassicales; Brassicaceae; Camelineae; Arabidopsis |
| *infA* | AIG90462 | 710 | 1520 | 1523 | 3515 | 3621 | 3295 | -1.48609 | 0.0011 | down | . | . | GO:0009507^cellular_component^chloroplast`GO:0019843^molecular_function^rRNA binding`GO:0003743^molecular_function^translation initiation factor activity | IF1C_LOLPR^IF1C_LOLPR^Q:1-177,H:32-90^100%ID^E:2e-23^RecName: Full=Translation initiation factor IF-1, chloroplastic {ECO:0000255\|HAMAP-Rule:MF_00075};^Eukaryota; Viridiplantae; Streptophyta; Embryophyta; Tracheophyta; Spermatophyta; Magnoliophyta; Liliopsida; Poales; Poaceae; BOP clade; Pooideae; Poodae; Poeae; Poeae Chloroplast Group 2 (Poeae type); Loliinae; Lolium |
| *rpl2* | TRIAE_CS42_2DL_TGACv1_167335_AA0565380 | 91 | 190 | 202 | 469 | 464 | 411 | -1.48671 | 0.001365 | down | . | . | GO:0009507^cellular_component^chloroplast`GO:0015934^cellular_component^large ribosomal subunit`GO:0019843^molecular_function^rRNA binding`GO:0003735^molecular_function^structural constituent of ribosome`GO:0006412^biological_process^translation | RK14_LOLPR^RK14_LOLPR^Q:1-369,H:1-123^98.37%ID^E:1e-82^RecName: Full=50S ribosomal protein L14, chloroplastic {ECO:0000255\|HAMAP-Rule:MF_01367};^Eukaryota; Viridiplantae; Streptophyta; Embryophyta; Tracheophyta; Spermatophyta; Magnoliophyta; Liliopsida; Poales; Poaceae; BOP clade; Pooideae; Poodae; Poeae; Poeae Chloroplast Group 2 (Poeae type); Loliinae; Lolium |
| LOC109771588 | TRIAE_CS42_2DL_TGACv1_160461_AA0550780 | 185 | 206 | 163 | 479 | 547 | 510 | -1.48957 | 0.001291 | down | COG0124^Histidyl-trna synthetase | KEGG:ath:AT3G46100`KO:K01892 | GO:0009507^cellular_component^chloroplast`GO:0005739^cellular_component^mitochondrion`GO:0005524^molecular_function^ATP binding`GO:0004821^molecular_function^histidine-tRNA ligase activity`GO:0006427^biological_process^histidyl-tRNA aminoacylation | SYHM_ARATH^SYHM_ARATH^Q:172-1428,H:67-486^68.81%ID^E:0^RecName: Full=Histidine--tRNA ligase, chloroplastic/mitochondrial {ECO:0000305};^Eukaryota; Viridiplantae; Streptophyta; Embryophyta; Tracheophyta; Spermatophyta; Magnoliophyta; eudicotyledons; Gunneridae; Pentapetalae; rosids; malvids; Brassicales; Brassicaceae; Camelineae; Arabidopsis |
| LOC109768050 | TRIAE_CS42_3AS_TGACv1_212452_AA0700940 | 22 | 23 | 14 | 77 | 58 | 31 | -1.50306 | 0.00473 | down | COG0656^reductase | KEGG:ath:AT2G37770 | GO:0009507^cellular_component^chloroplast`GO:0008106^molecular_function^alcohol dehydrogenase (NADP+) activity`GO:0004033^molecular_function^aldo-keto reductase (NADP) activity`GO:0070401^molecular_function^NADP+ binding`GO:0016229^molecular_function^steroid dehydrogenase activity`GO:0055114^biological_process^oxidation-reduction process`GO:0009409^biological_process^response to cold`GO:0009651^biological_process^response to salt stress`GO:0009636^biological_process^response to toxic substance`GO:0009414^biological_process^response to water deprivation | AKRC9_ARATH^AKRC9_ARATH^Q:82-852,H:57-315^69.11%ID^E:2e-117^RecName: Full=NADPH-dependent aldo-keto reductase, chloroplastic {ECO:0000303\|PubMed:21169366};^Eukaryota; Viridiplantae; Streptophyta; Embryophyta; Tracheophyta; Spermatophyta; Magnoliophyta; eudicotyledons; Gunneridae; Pentapetalae; rosids; malvids; Brassicales; Brassicaceae; Camelineae; Arabidopsis |
| LOC109740650 | TRIAE_CS42_4AS_TGACv1_306976_AA1015620 | 933 | 539 | 654 | 1616 | 2270 | 2158 | -1.52994 | 0.000752 | down | COG0317^In eubacteria ppGpp (guanosine 3'-diphosphate 5-' diphosphate) is a mediator of the stringent response that coordinates a variety of cellular activities in response to changes in nutritional abundance (By similarity) | KEGG:osa:4332791 | GO:0009507^cellular_component^chloroplast`GO:0005524^molecular_function^ATP binding`GO:0005525^molecular_function^GTP binding`GO:0008728^molecular_function^GTP diphosphokinase activity`GO:0008893^molecular_function^guanosine-3',5'-bis(diphosphate) 3'-diphosphatase activity`GO:0016301^molecular_function^kinase activity`GO:0015969^biological_process^guanosine tetraphosphate metabolic process`GO:0010150^biological_process^leaf senescence`GO:0015979^biological_process^photosynthesis`GO:0009611^biological_process^response to wounding | RSH1_ORYSJ^RSH1_ORYSJ^Q:1-2454,H:1-819^86.51%ID^E:0^RecName: Full=Putative GTP diphosphokinase RSH1, chloroplastic;^Eukaryota; Viridiplantae; Streptophyta; Embryophyta; Tracheophyta; Spermatophyta; Magnoliophyta; Liliopsida; Poales; Poaceae; BOP clade; Oryzoideae; Oryzeae; Oryzinae; Oryza; Oryza sativa |
| AA1208110 | TRIAE_CS42_5AL_TGACv1_374748_AA1208110 | 188 | 294 | 377 | 941 | 812 | 735 | -1.54387 | 0.00072 | down | . | . | GO:0009507^cellular_component^chloroplast`GO:0015934^cellular_component^large ribosomal subunit`GO:0003723^molecular_function^RNA binding`GO:0003735^molecular_function^structural constituent of ribosome`GO:0016740^molecular_function^transferase activity`GO:0006412^biological_process^translation | RK2_LOLPR^RK2_LOLPR^Q:1-390,H:133-262^94.62%ID^E:6e-81^RecName: Full=50S ribosomal protein L2, chloroplastic;^Eukaryota; Viridiplantae; Streptophyta; Embryophyta; Tracheophyta; Spermatophyta; Magnoliophyta; Liliopsida; Poales; Poaceae; BOP clade; Pooideae; Poodae; Poeae; Poeae Chloroplast Group 2 (Poeae type); Loliinae; Lolium |
| AIG90460 | AIG90460 | 536 | 1059 | 1397 | 2809 | 3271 | 2614 | -1.54902 | 0.000614 | down | . | . | GO:0009507^cellular_component^chloroplast`GO:0005840^cellular_component^ribosome`GO:0019843^molecular_function^rRNA binding`GO:0003735^molecular_function^structural constituent of ribosome`GO:0006412^biological_process^translation | RR11_WHEAT^RR11_WHEAT^Q:1-429,H:1-143^100%ID^E:3e-98^RecName: Full=30S ribosomal protein S11, chloroplastic {ECO:0000255\|HAMAP-Rule:MF_01310};^Eukaryota; Viridiplantae; Streptophyta; Embryophyta; Tracheophyta; Spermatophyta; Magnoliophyta; Liliopsida; Poales; Poaceae; BOP clade; Pooideae; Triticodae; Triticeae; Triticinae; Triticum |
| *trnK* | AIG90409 | 88 | 68 | 90 | 270 | 150 | 300 | -1.5617 | 0.000869 | down | . | . | GO:0009507^cellular_component^chloroplast`GO:0003723^molecular_function^RNA binding`GO:0006397^biological_process^mRNA processing`GO:0008033^biological_process^tRNA processing | MATK_HORML^MATK_HORML^Q:1-1536,H:1-511^98.05%ID^E:0^RecName: Full=Maturase K {ECO:0000255\|HAMAP-Rule:MF_01390};^Eukaryota; Viridiplantae; Streptophyta; Embryophyta; Tracheophyta; Spermatophyta; Magnoliophyta; Liliopsida; Poales; Poaceae; BOP clade; Pooideae; Triticodae; Triticeae; Hordeinae; Hordeum |
| *AOC1* | TRIAE_CS42_6BL_TGACv1_500025_AA1597550 | 69 | 34 | 30 | 179 | 116 | 97 | -1.57665 | 0.00111 | down | ENOG4111X07^allene oxide cyclase | KEGG:osa:4333201`KO:K10525 | GO:0009507^cellular_component^chloroplast`GO:0046423^molecular_function^allene-oxide cyclase activity`GO:0009734^biological_process^auxin-activated signaling pathway`GO:0007623^biological_process^circadian rhythm`GO:0050832^biological_process^defense response to fungus`GO:0080186^biological_process^developmental vegetative growth`GO:0009908^biological_process^flower development`GO:0009864^biological_process^induced systemic resistance, jasmonic acid mediated signaling pathway`GO:0009695^biological_process^jasmonic acid biosynthetic process`GO:0048573^biological_process^photoperiodism, flowering`GO:1900367^biological_process^positive regulation of defense response to insect`GO:0009737^biological_process^response to abscisic acid`GO:0009646^biological_process^response to absence of light`GO:0009637^biological_process^response to blue light`GO:0009723^biological_process^response to ethylene`GO:0010218^biological_process^response to far red light`GO:0042542^biological_process^response to hydrogen peroxide`GO:0009625^biological_process^response to insect`GO:0009753^biological_process^response to jasmonic acid`GO:0009416^biological_process^response to light stimulus`GO:0010038^biological_process^response to metal ion`GO:0010114^biological_process^response to red light`GO:0009751^biological_process^response to salicylic acid`GO:0009651^biological_process^response to salt stress`GO:0033274^biological_process^response to vitamin B2`GO:0009611^biological_process^response to wounding`GO:0006636^biological_process^unsaturated fatty acid biosynthetic process | AOC_ORYSJ^AOC_ORYSJ^Q:136-717,H:48-240^89.69%ID^E:7e-114^RecName: Full=Allene oxide cyclase, chloroplastic {ECO:0000303\|Ref.1, ECO:0000303\|Ref.2};^Eukaryota; Viridiplantae; Streptophyta; Embryophyta; Tracheophyta; Spermatophyta; Magnoliophyta; Liliopsida; Poales; Poaceae; BOP clade; Oryzoideae; Oryzeae; Oryzinae; Oryza; Oryza sativa |
| *rpoC2* | AIG90420 | 76 | 108 | 193 | 408 | 349 | 366 | -1.58178 | 0.000604 | down | . | . | GO:0009507^cellular_component^chloroplast`GO:0003677^molecular_function^DNA binding`GO:0003899^molecular_function^DNA-directed 5'-3' RNA polymerase activity`GO:0006351^biological_process^transcription, DNA-templated | RPOC2_WHEAT^RPOC2_WHEAT^Q:1-4437,H:1-1479^100%ID^E:0^RecName: Full=DNA-directed RNA polymerase subunit beta'' {ECO:0000255\|HAMAP-Rule:MF_01324};^Eukaryota; Viridiplantae; Streptophyta; Embryophyta; Tracheophyta; Spermatophyta; Magnoliophyta; Liliopsida; Poales; Poaceae; BOP clade; Pooideae; Triticodae; Triticeae; Triticinae; Triticum |
| *rps3* | AIG90466 | 1006 | 1902 | 2445 | 5530 | 5623 | 4952 | -1.59881 | 0.00037 | down | . | . | GO:0009507^cellular_component^chloroplast`GO:0015935^cellular_component^small ribosomal subunit`GO:0019843^molecular_function^rRNA binding`GO:0003735^molecular_function^structural constituent of ribosome`GO:0006412^biological_process^translation | RR3_WHEAT^RR3_WHEAT^Q:1-717,H:1-239^100%ID^E:2e-174^RecName: Full=30S ribosomal protein S3, chloroplastic;^Eukaryota; Viridiplantae; Streptophyta; Embryophyta; Tracheophyta; Spermatophyta; Magnoliophyta; Liliopsida; Poales; Poaceae; BOP clade; Pooideae; Triticodae; Triticeae; Triticinae; Triticum |
| LOC109742413 | TRIAE_CS42_3B_TGACv1_220717_AA0716550 | 34 | 25 | 19 | 93 | 65 | 78 | -1.61094 | 0.001392 | down | COG0462^Phosphoribosyl pyrophosphate synthase | KEGG:osa:4324718 | GO:0009507^cellular_component^chloroplast`GO:0005524^molecular_function^ATP binding`GO:0016301^molecular_function^kinase activity`GO:0000287^molecular_function^magnesium ion binding`GO:0004749^molecular_function^ribose phosphate diphosphokinase activity`GO:0009116^biological_process^nucleoside metabolic process`GO:0009165^biological_process^nucleotide biosynthetic process | KPRS3_ORYSJ^KPRS3_ORYSJ^Q:67-1221,H:39-409^79.38%ID^E:0^RecName: Full=Ribose-phosphate pyrophosphokinase 3, chloroplastic;^Eukaryota; Viridiplantae; Streptophyta; Embryophyta; Tracheophyta; Spermatophyta; Magnoliophyta; Liliopsida; Poales; Poaceae; BOP clade; Oryzoideae; Oryzeae; Oryzinae; Oryza; Oryza sativa |
| LOC109751011 | TRIAE_CS42_5AL_TGACv1_376103_AA1232190 | 93 | 104 | 101 | 176 | 346 | 380 | -1.617 | 0.000473 | down | COG1234^Zinc phosphodiesterase, which displays some tRNA 3'- processing endonuclease activity. Probably involved in tRNA maturation, by removing a 3'-trailer from precursor tRNA (By similarity) | KEGG:ath:AT2G04530`KO:K00784 | GO:0009507^cellular_component^chloroplast`GO:0042781^molecular_function^3'-tRNA processing endoribonuclease activity`GO:0046872^molecular_function^metal ion binding`GO:0042780^biological_process^tRNA 3'-end processing`GO:0008033^biological_process^tRNA processing | RNZ2_ARATH^RNZ2_ARATH^Q:181-1080,H:55-354^67.67%ID^E:5e-156^RecName: Full=tRNase Z TRZ2, chloroplastic {ECO:0000303\|PubMed:16336119};^Eukaryota; Viridiplantae; Streptophyta; Embryophyta; Tracheophyta; Spermatophyta; Magnoliophyta; eudicotyledons; Gunneridae; Pentapetalae; rosids; malvids; Brassicales; Brassicaceae; Camelineae; Arabidopsis |
| *trnH* | TRIAE_CS42_5AL_TGACv1_378987_AA1255240 | 80 | 98 | 124 | 323 | 310 | 289 | -1.62199 | 0.000448 | down | . | . | GO:0009507^cellular_component^chloroplast`GO:0015934^cellular_component^large ribosomal subunit`GO:0003723^molecular_function^RNA binding`GO:0003735^molecular_function^structural constituent of ribosome`GO:0016740^molecular_function^transferase activity`GO:0006412^biological_process^translation | RK2_NICDE^RK2_NICDE^Q:1-402,H:133-266^82.09%ID^E:6e-54^RecName: Full=50S ribosomal protein L2, chloroplastic;^Eukaryota; Viridiplantae; Streptophyta; Embryophyta; Tracheophyta; Spermatophyta; Magnoliophyta; eudicotyledons; Gunneridae; Pentapetalae; asterids; lamiids; Solanales; Solanaceae; Nicotianoideae; Nicotianeae; Nicotiana |
| LOC109731694 | TRIAE_CS42_7BS_TGACv1_592504_AA1939410 | 36 | 44 | 37 | 107 | 177 | 73 | -1.62609 | 0.000796 | down | ENOG410YN4N^Plant lipoxygenase may be involved in a number of diverse aspects of plant physiology including growth and development, pest resistance, and senescence or responses to wounding (By similarity) | KEGG:osa:4345994`KO:K00454 | GO:0009507^cellular_component^chloroplast`GO:0016165^molecular_function^linoleate 13S-lipoxygenase activity`GO:0046872^molecular_function^metal ion binding`GO:0031408^biological_process^oxylipin biosynthetic process | LOXC2_ORYSJ^LOXC2_ORYSJ^Q:316-2718,H:111-919^78.69%ID^E:0^RecName: Full=Probable lipoxygenase 8, chloroplastic;^Eukaryota; Viridiplantae; Streptophyta; Embryophyta; Tracheophyta; Spermatophyta; Magnoliophyta; Liliopsida; Poales; Poaceae; BOP clade; Oryzoideae; Oryzeae; Oryzinae; Oryza; Oryza sativa |
| LOC109746104 | TRIAE_CS42_7AL_TGACv1_556952_AA1774080 | 723 | 479 | 242 | 1974 | 1501 | 915 | -1.62715 | 0.000301 | down | COG2214^DNAj domain protein | KEGG:ath:AT4G36040 | GO:0009507^cellular_component^chloroplast`GO:0009570^cellular_component^chloroplast stroma`GO:0005634^cellular_component^nucleus`GO:0009536^cellular_component^plastid | DNJ11_ARATH^DNJ11_ARATH^Q:142-420,H:67-161^47%ID^E:9e-20^RecName: Full=Chaperone protein dnaJ 11, chloroplastic;^Eukaryota; Viridiplantae; Streptophyta; Embryophyta; Tracheophyta; Spermatophyta; Magnoliophyta; eudicotyledons; Gunneridae; Pentapetalae; rosids; malvids; Brassicales; Brassicaceae; Camelineae; Arabidopsis |
| AIG90464 | AIG90464 | 841 | 1660 | 1847 | 4481 | 4670 | 4227 | -1.63242 | 0.000267 | down | . | . | GO:0009507^cellular_component^chloroplast`GO:0015934^cellular_component^large ribosomal subunit`GO:0019843^molecular_function^rRNA binding`GO:0003735^molecular_function^structural constituent of ribosome`GO:0006412^biological_process^translation | RK14_WHEAT^RK14_WHEAT^Q:1-369,H:1-123^100%ID^E:1e-83^RecName: Full=50S ribosomal protein L14, chloroplastic {ECO:0000255\|HAMAP-Rule:MF_01367};^Eukaryota; Viridiplantae; Streptophyta; Embryophyta; Tracheophyta; Spermatophyta; Magnoliophyta; Liliopsida; Poales; Poaceae; BOP clade; Pooideae; Triticodae; Triticeae; Triticinae; Triticum |
| AA0833730 | TRIAE_CS42_3B_TGACv1_242920_AA0833730 | 11 | 10 | 16 | 49 | 39 | 28 | -1.65107 | 0.003059 | down | . | . | GO:0009507^cellular_component^chloroplast`GO:0015934^cellular_component^large ribosomal subunit`GO:0003723^molecular_function^RNA binding`GO:0003735^molecular_function^structural constituent of ribosome`GO:0016740^molecular_function^transferase activity`GO:0006412^biological_process^translation | RK2_LOLPR^RK2_LOLPR^Q:67-495,H:131-273^97.2%ID^E:6e-83^RecName: Full=50S ribosomal protein L2, chloroplastic;^Eukaryota; Viridiplantae; Streptophyta; Embryophyta; Tracheophyta; Spermatophyta; Magnoliophyta; Liliopsida; Poales; Poaceae; BOP clade; Pooideae; Poodae; Poeae; Poeae Chloroplast Group 2 (Poeae type); Loliinae; Lolium |
| LOC109773061 | TRIAE_CS42_1DL_TGACv1_061488_AA0196780 | 29 | 26 | 31 | 143 | 65 | 63 | -1.66216 | 0.00079 | down | ENOG411126M^Dual specificity phosphatase, catalytic domain | KEGG:ath:AT3G01510 | GO:0009507^cellular_component^chloroplast`GO:0009569^cellular_component^chloroplast starch grain`GO:0009570^cellular_component^chloroplast stroma`GO:0043036^cellular_component^starch grain`GO:0019203^molecular_function^carbohydrate phosphatase activity`GO:0008138^molecular_function^protein tyrosine/serine/threonine phosphatase activity`GO:0005983^biological_process^starch catabolic process | LSF1_ARATH^LSF1_ARATH^Q:226-1788,H:71-590^60.99%ID^E:0^RecName: Full=Phosphoglucan phosphatase LSF1, chloroplastic;^Eukaryota; Viridiplantae; Streptophyta; Embryophyta; Tracheophyta; Spermatophyta; Magnoliophyta; eudicotyledons; Gunneridae; Pentapetalae; rosids; malvids; Brassicales; Brassicaceae; Camelineae; Arabidopsis |
| *rpl14* | TRIAE_CS42_2BL_TGACv1_129449_AA0384230 | 214 | 358 | 424 | 1127 | 1064 | 995 | -1.68815 | 0.000169 | down | . | . | GO:0009507^cellular_component^chloroplast`GO:0015934^cellular_component^large ribosomal subunit`GO:0019843^molecular_function^rRNA binding`GO:0003735^molecular_function^structural constituent of ribosome`GO:0006412^biological_process^translation | RK14_LOLPR^RK14_LOLPR^Q:1-369,H:1-123^95.93%ID^E:2e-79^RecName: Full=50S ribosomal protein L14, chloroplastic {ECO:0000255\|HAMAP-Rule:MF_01367};^Eukaryota; Viridiplantae; Streptophyta; Embryophyta; Tracheophyta; Spermatophyta; Magnoliophyta; Liliopsida; Poales; Poaceae; BOP clade; Pooideae; Poodae; Poeae; Poeae Chloroplast Group 2 (Poeae type); Loliinae; Lolium |
| *IPT3* | TRIAE_CS42_3DL_TGACv1_249092_AA0837320 | 13 | 13 | 11 | 23 | 34 | 62 | -1.6946 | 0.002185 | down | COG0324^Catalyzes the transfer of a dimethylallyl group onto the adenine at position 37 in tRNAs that read codons beginning with uridine, leading to the formation of N6-(dimethylallyl)adenosine (i(6)A) (By similarity) | KEGG:ath:AT1G68460`KO:K10760 | GO:0009507^cellular_component^chloroplast`GO:0005739^cellular_component^mitochondrion`GO:0009536^cellular_component^plastid`GO:0052623^molecular_function^ADP dimethylallyltransferase activity`GO:0009824^molecular_function^AMP dimethylallyltransferase activity`GO:0005524^molecular_function^ATP binding`GO:0052622^molecular_function^ATP dimethylallyltransferase activity`GO:0052381^molecular_function^tRNA dimethylallyltransferase activity`GO:0009691^biological_process^cytokinin biosynthetic process`GO:0006400^biological_process^tRNA modification | IPT1_ARATH^IPT1_ARATH^Q:157-894,H:52-310^44.87%ID^E:8e-59^RecName: Full=Adenylate isopentenyltransferase 1, chloroplastic;^Eukaryota; Viridiplantae; Streptophyta; Embryophyta; Tracheophyta; Spermatophyta; Magnoliophyta; eudicotyledons; Gunneridae; Pentapetalae; rosids; malvids; Brassicales; Brassicaceae; Camelineae; Arabidopsis |
| LOC109750542 | TRIAE_CS42_2DL_TGACv1_159225_AA0534800 | 112 | 140 | 170 | 395 | 421 | 603 | -1.76292 | 9.49E-05 | down | COG0534^Mate efflux family protein | KEGG:ath:AT2G32230`KO:K18213 | GO:0009507^cellular_component^chloroplast`GO:0005739^cellular_component^mitochondrion`GO:0004526^molecular_function^ribonuclease P activity`GO:0001682^biological_process^tRNA 5'-leader removal`GO:0008033^biological_process^tRNA processing | PRRP1_ARATH^PRRP1_ARATH^Q:238-1683,H:84-568^58.93%ID^E:0^RecName: Full=Proteinaceous RNase P 1, chloroplastic/mitochondrial;^Eukaryota; Viridiplantae; Streptophyta; Embryophyta; Tracheophyta; Spermatophyta; Magnoliophyta; eudicotyledons; Gunneridae; Pentapetalae; rosids; malvids; Brassicales; Brassicaceae; Camelineae; Arabidopsis |
| LOC109745746 | TRIAE_CS42_5DL_TGACv1_434734_AA1440720 | 112 | 53 | 48 | 283 | 194 | 245 | -1.781 | 0.00011 | down | COG1525^nuclease | KEGG:ath:AT5G07350`KO:K15979 | GO:0005618^cellular_component^cell wall`GO:0009507^cellular_component^chloroplast`GO:0005737^cellular_component^cytoplasm`GO:0010494^cellular_component^cytoplasmic stress granule`GO:0005829^cellular_component^cytosol`GO:0005783^cellular_component^endoplasmic reticulum`GO:0005635^cellular_component^nuclear envelope`GO:0000932^cellular_component^P-body`GO:0048471^cellular_component^perinuclear region of cytoplasm`GO:0005886^cellular_component^plasma membrane`GO:0016442^cellular_component^RISC complex`GO:0003729^molecular_function^mRNA binding`GO:0004518^molecular_function^nuclease activity`GO:0003723^molecular_function^RNA binding`GO:0034605^biological_process^cellular response to heat`GO:0031047^biological_process^gene silencing by RNA`GO:0006402^biological_process^mRNA catabolic process`GO:0006397^biological_process^mRNA processing`GO:0010372^biological_process^positive regulation of gibberellin biosynthetic process`GO:0046686^biological_process^response to cadmium ion`GO:0009651^biological_process^response to salt stress | TSN1_ARATH^TSN1_ARATH^Q:547-3471,H:1-991^62.85%ID^E:0^RecName: Full=Ribonuclease TUDOR 1 {ECO:0000303\|PubMed:20396901};^Eukaryota; Viridiplantae; Streptophyta; Embryophyta; Tracheophyta; Spermatophyta; Magnoliophyta; eudicotyledons; Gunneridae; Pentapetalae; rosids; malvids; Brassicales; Brassicaceae; Camelineae; Arabidopsis |
| LOC109773004 | TRIAE_CS42_1DL_TGACv1_061982_AA0206800 | 272 | 292 | 306 | 685 | 1031 | 1277 | -1.8008 | 5.28E-05 | down | COG0740^Cleaves peptides in various proteins in a process that requires ATP hydrolysis. Has a chymotrypsin-like activity. Plays a major role in the degradation of misfolded proteins (By similarity) | KEGG:ath:AT1G49970`KO:K01358 | GO:0009507^cellular_component^chloroplast`GO:0009941^cellular_component^chloroplast envelope`GO:0009570^cellular_component^chloroplast stroma`GO:0009534^cellular_component^chloroplast thylakoid`GO:0009840^cellular_component^chloroplastic endopeptidase Clp complex`GO:0009532^cellular_component^plastid stroma`GO:0003723^molecular_function^RNA binding`GO:0004252^molecular_function^serine-type endopeptidase activity`GO:0009658^biological_process^chloroplast organization`GO:0006396^biological_process^RNA processing`GO:0006364^biological_process^rRNA processing | CLPR1_ARATH^CLPR1_ARATH^Q:166-1140,H:62-385^57.36%ID^E:1e-112^RecName: Full=ATP-dependent Clp protease proteolytic subunit-related protein 1, chloroplastic {ECO:0000303\|PubMed:11299370};^Eukaryota; Viridiplantae; Streptophyta; Embryophyta; Tracheophyta; Spermatophyta; Magnoliophyta; eudicotyledons; Gunneridae; Pentapetalae; rosids; malvids; Brassicales; Brassicaceae; Camelineae; Arabidopsis |
| LOC100828411 | TRIAE_CS42_2DL_TGACv1_158861_AA0527300 | 102 | 58 | 61 | 276 | 200 | 287 | -1.80545 | 8.31E-05 | down | COG1324^tolerance protein | . | GO:0009507^cellular_component^chloroplast`GO:0005507^molecular_function^copper ion binding`GO:0070207^biological_process^protein homotrimerization`GO:0010038^biological_process^response to metal ion | CUTA1_ORYSJ^CUTA1_ORYSJ^Q:193-528,H:65-176^93.75%ID^E:4e-70^RecName: Full=Protein CutA 1, chloroplastic;^Eukaryota; Viridiplantae; Streptophyta; Embryophyta; Tracheophyta; Spermatophyta; Magnoliophyta; Liliopsida; Poales; Poaceae; BOP clade; Oryzoideae; Oryzeae; Oryzinae; Oryza; Oryza sativa |
| LOC109777065 | TRIAE_CS42_6BS_TGACv1_514022_AA1654050 | 76 | 105 | 76 | 251 | 294 | 361 | -1.83451 | 5.58E-05 | down | COG0064^Allows the formation of correctly charged Asn-tRNA(Asn) or Gln-tRNA(Gln) through the transamidation of misacylated Asp- tRNA(Asn) or Glu-tRNA(Gln) in organisms which lack either or both of asparaginyl-tRNA or glutaminyl-tRNA synthetases. The reaction takes place in the presence of glutamine and ATP through an activated phospho-Asp-tRNA(Asn) or phospho-Glu-tRNA(Gln) (By similarity) | KEGG:osa:4350686`KO:K02434 | GO:0009507^cellular_component^chloroplast`GO:0005739^cellular_component^mitochondrion`GO:0005524^molecular_function^ATP binding`GO:0050567^molecular_function^glutaminyl-tRNA synthase (glutamine-hydrolyzing) activity`GO:0070681^biological_process^glutaminyl-tRNAGln biosynthesis via transamidation`GO:0006412^biological_process^translation | GATB_ORYSJ^GATB_ORYSJ^Q:1-1638,H:1-543^87.39%ID^E:0^RecName: Full=Glutamyl-tRNA(Gln) amidotransferase subunit B, chloroplastic/mitochondrial {ECO:0000255\|HAMAP-Rule:MF_03147};^Eukaryota; Viridiplantae; Streptophyta; Embryophyta; Tracheophyta; Spermatophyta; Magnoliophyta; Liliopsida; Poales; Poaceae; BOP clade; Oryzoideae; Oryzeae; Oryzinae; Oryza; Oryza sativa |
| *rps4* | AIG90430 | 218 | 266 | 329 | 1038 | 809 | 1030 | -1.8347 | 3.68E-05 | down | . | . | GO:0009507^cellular_component^chloroplast`GO:0015935^cellular_component^small ribosomal subunit`GO:0019843^molecular_function^rRNA binding`GO:0003735^molecular_function^structural constituent of ribosome`GO:0006412^biological_process^translation | RR4_WHEAT^RR4_WHEAT^Q:1-603,H:1-201^100%ID^E:7e-144^RecName: Full=30S ribosomal protein S4, chloroplastic;^Eukaryota; Viridiplantae; Streptophyta; Embryophyta; Tracheophyta; Spermatophyta; Magnoliophyta; Liliopsida; Poales; Poaceae; BOP clade; Pooideae; Triticodae; Triticeae; Triticinae; Triticum |
| LOC109775965 | TRIAE_CS42_3B_TGACv1_223462_AA0782590 | 39 | 32 | 35 | 205 | 93 | 79 | -1.83828 | 0.000114 | down | COG0237^Catalyzes the phosphorylation of the 3'-hydroxyl group of dephosphocoenzyme A to form coenzyme A (By similarity) | KEGG:osa:4326028`KO:K00859 | GO:0009507^cellular_component^chloroplast`GO:0005777^cellular_component^peroxisome`GO:0005773^cellular_component^vacuole`GO:0005524^molecular_function^ATP binding`GO:0004140^molecular_function^dephospho-CoA kinase activity`GO:0015937^biological_process^coenzyme A biosynthetic process | COAE_ORYSJ^COAE_ORYSJ^Q:1-690,H:1-230^82.61%ID^E:1e-117^RecName: Full=Dephospho-CoA kinase;^Eukaryota; Viridiplantae; Streptophyta; Embryophyta; Tracheophyta; Spermatophyta; Magnoliophyta; Liliopsida; Poales; Poaceae; BOP clade; Oryzoideae; Oryzeae; Oryzinae; Oryza; Oryza sativa |
| AA0671210 | TRIAE_CS42_3AL_TGACv1_207763_AA0671210 | 40 | 83 | 98 | 292 | 254 | 242 | -1.84202 | 5.65E-05 | down | . | . | GO:0009507^cellular_component^chloroplast`GO:0015935^cellular_component^small ribosomal subunit`GO:0019843^molecular_function^rRNA binding`GO:0003735^molecular_function^structural constituent of ribosome`GO:0006412^biological_process^translation | RR19_LOLPR^RR19_LOLPR^Q:1-213,H:23-93^95.77%ID^E:5e-43^RecName: Full=30S ribosomal protein S19, chloroplastic {ECO:0000255\|HAMAP-Rule:MF_00531};^Eukaryota; Viridiplantae; Streptophyta; Embryophyta; Tracheophyta; Spermatophyta; Magnoliophyta; Liliopsida; Poales; Poaceae; BOP clade; Pooideae; Poodae; Poeae; Poeae Chloroplast Group 2 (Poeae type); Loliinae; Lolium |
| LOC109736139 | TRIAE_CS42_5BL_TGACv1_405838_AA1336190 | 22 | 21 | 25 | 60 | 94 | 90 | -1.85595 | 0.000178 | down | COG1022^Amp-dependent synthetase and ligase | KEGG:ath:AT3G23790`KO:K01897 | GO:0009941^cellular_component^chloroplast envelope`GO:0016874^molecular_function^ligase activity`GO:0006631^biological_process^fatty acid metabolic process | AAE16_ARATH^AAE16_ARATH^Q:220-2214,H:59-722^64.62%ID^E:0^RecName: Full=Probable acyl-activating enzyme 16, chloroplastic;^Eukaryota; Viridiplantae; Streptophyta; Embryophyta; Tracheophyta; Spermatophyta; Magnoliophyta; eudicotyledons; Gunneridae; Pentapetalae; rosids; malvids; Brassicales; Brassicaceae; Camelineae; Arabidopsis |
| *rpl16* | AIG90465 | 625 | 1163 | 1447 | 3857 | 4385 | 3403 | -1.8587 | 2.41E-05 | down | . | . | GO:0009507^cellular_component^chloroplast`GO:0005840^cellular_component^ribosome`GO:0019843^molecular_function^rRNA binding`GO:0003735^molecular_function^structural constituent of ribosome`GO:0006412^biological_process^translation | RK16_WHEAT^RK16_WHEAT^Q:31-432,H:3-136^99.25%ID^E:1e-91^RecName: Full=50S ribosomal protein L16, chloroplastic {ECO:0000255\|HAMAP-Rule:MF_01342};^Eukaryota; Viridiplantae; Streptophyta; Embryophyta; Tracheophyta; Spermatophyta; Magnoliophyta; Liliopsida; Poales; Poaceae; BOP clade; Pooideae; Triticodae; Triticeae; Triticinae; Triticum |
| LOC100836564 | TRIAE_CS42_1BL_TGACv1_033345_AA0138870 | 33 | 50 | 57 | 212 | 157 | 155 | -1.91217 | 3.89E-05 | down | ENOG411126M^Dual specificity phosphatase, catalytic domain | KEGG:ath:AT3G01510 | GO:0009507^cellular_component^chloroplast`GO:0009569^cellular_component^chloroplast starch grain`GO:0009570^cellular_component^chloroplast stroma`GO:0043036^cellular_component^starch grain`GO:0019203^molecular_function^carbohydrate phosphatase activity`GO:0008138^molecular_function^protein tyrosine/serine/threonine phosphatase activity`GO:0005983^biological_process^starch catabolic process | LSF1_ARATH^LSF1_ARATH^Q:220-1782,H:71-590^60.8%ID^E:0^RecName: Full=Phosphoglucan phosphatase LSF1, chloroplastic;^Eukaryota; Viridiplantae; Streptophyta; Embryophyta; Tracheophyta; Spermatophyta; Magnoliophyta; eudicotyledons; Gunneridae; Pentapetalae; rosids; malvids; Brassicales; Brassicaceae; Camelineae; Arabidopsis |
| *rpl20* | AIG90450 | 43 | 66 | 59 | 193 | 197 | 244 | -1.92877 | 2.73E-05 | down | . | . | GO:0009507^cellular_component^chloroplast`GO:0005840^cellular_component^ribosome`GO:0019843^molecular_function^rRNA binding`GO:0003735^molecular_function^structural constituent of ribosome`GO:0006412^biological_process^translation | RK20_WHEAT^RK20_WHEAT^Q:1-357,H:1-119^100%ID^E:7e-69^RecName: Full=50S ribosomal protein L20, chloroplastic {ECO:0000255\|HAMAP-Rule:MF_00382};^Eukaryota; Viridiplantae; Streptophyta; Embryophyta; Tracheophyta; Spermatophyta; Magnoliophyta; Liliopsida; Poales; Poaceae; BOP clade; Pooideae; Triticodae; Triticeae; Triticinae; Triticum |
| *rbcL* | TRIAE_CS42_1AL_TGACv1_007391_AA0055320 | 1425 | 2084 | 3853 | 8936 | 8155 | 10980 | -1.93857 | 9.37E-06 | down | . | . | GO:0009507^cellular_component^chloroplast`GO:0000287^molecular_function^magnesium ion binding`GO:0004497^molecular_function^monooxygenase activity`GO:0016984^molecular_function^ribulose-bisphosphate carboxylase activity`GO:0009853^biological_process^photorespiration`GO:0019253^biological_process^reductive pentose-phosphate cycle | RBL_WHEAT^RBL_WHEAT^Q:1-684,H:1-228^97.81%ID^E:4e-145^RecName: Full=Ribulose bisphosphate carboxylase large chain;^Eukaryota; Viridiplantae; Streptophyta; Embryophyta; Tracheophyta; Spermatophyta; Magnoliophyta; Liliopsida; Poales; Poaceae; BOP clade; Pooideae; Triticodae; Triticeae; Triticinae; Triticum |
| AIG90436 | AIG90436 | 202735 | 329863 | 382553 | 1261726 | 1068216 | 1324396 | -2.00833 | 4.01E-06 | down | . | . | GO:0009507^cellular_component^chloroplast`GO:0000287^molecular_function^magnesium ion binding`GO:0004497^molecular_function^monooxygenase activity`GO:0016984^molecular_function^ribulose-bisphosphate carboxylase activity`GO:0009853^biological_process^photorespiration`GO:0019253^biological_process^reductive pentose-phosphate cycle | RBL_WHEAT^RBL_WHEAT^Q:1-1431,H:1-477^100%ID^E:0^RecName: Full=Ribulose bisphosphate carboxylase large chain;^Eukaryota; Viridiplantae; Streptophyta; Embryophyta; Tracheophyta; Spermatophyta; Magnoliophyta; Liliopsida; Poales; Poaceae; BOP clade; Pooideae; Triticodae; Triticeae; Triticinae; Triticum |
| AA2168590 | TRIAE_CS42_U_TGACv1_719502_AA2168590 | 6051 | 9738 | 15509 | 44098 | 38848 | 50863 | -2.10423 | 1.25E-06 | down | . | . | GO:0009507^cellular_component^chloroplast`GO:0000287^molecular_function^magnesium ion binding`GO:0004497^molecular_function^monooxygenase activity`GO:0016984^molecular_function^ribulose-bisphosphate carboxylase activity`GO:0009853^biological_process^photorespiration`GO:0019253^biological_process^reductive pentose-phosphate cycle | RBL_WHEAT^RBL_WHEAT^Q:1-309,H:1-103^99.03%ID^E:7e-53^RecName: Full=Ribulose bisphosphate carboxylase large chain;^Eukaryota; Viridiplantae; Streptophyta; Embryophyta; Tracheophyta; Spermatophyta; Magnoliophyta; Liliopsida; Poales; Poaceae; BOP clade; Pooideae; Triticodae; Triticeae; Triticinae; Triticum |
| *rps7* | TRIAE_CS42_1DL_TGACv1_067245_AA0238100 | 134 | 162 | 139 | 636 | 747 | 630 | -2.22704 | 4.20E-07 | down | COG0049^One of the primary rRNA binding proteins, it binds directly to 16S rRNA where it nucleates assembly of the head domain of the 30S subunit. Is located at the subunit interface close to the decoding center, probably blocks exit of the E-site tRNA (By similarity) | KEGG:sbi:SobiCp068`KEGG:sbi:SobiCp081`KO:K02992 | GO:0009507^cellular_component^chloroplast`GO:0005840^cellular_component^ribosome`GO:0015935^cellular_component^small ribosomal subunit`GO:0003729^molecular_function^mRNA binding`GO:0019843^molecular_function^rRNA binding`GO:0003735^molecular_function^structural constituent of ribosome`GO:0000028^biological_process^ribosomal small subunit assembly`GO:0006412^biological_process^translation | RR7_SORBI^RR7_SORBI^Q:197-3,H:71-135^98.46%ID^E:1e-37^RecName: Full=30S ribosomal protein S7, chloroplastic;^Eukaryota; Viridiplantae; Streptophyta; Embryophyta; Tracheophyta; Spermatophyta; Magnoliophyta; Liliopsida; Poales; Poaceae; PACMAD clade; Panicoideae; Andropogonodae; Andropogoneae; Sorghinae; Sorghum |
| LOC100831057 | TRIAE_CS42_5BS_TGACv1_424552_AA1390550 | 8 | 8 | 3 | 30 | 42 | 30 | -2.42124 | 2.19E-05 | down | . | KEGG:ag:BAM20979`KO:K20506 | GO:0009507^cellular_component^chloroplast`GO:0016787^molecular_function^hydrolase activity`GO:0016829^molecular_function^lyase activity`GO:0006952^biological_process^defense response`GO:0008152^biological_process^metabolic process | TCEA2_TULGE^TCEA2_TULGE^Q:25-927,H:77-380^43.99%ID^E:3e-79^RecName: Full=Tuliposide A-converting enzyme 2, chloroplastic;^Eukaryota; Viridiplantae; Streptophyta; Embryophyta; Tracheophyta; Spermatophyta; Magnoliophyta; Liliopsida; Liliales; Liliaceae; Tulipa |
| LOC109751369 | TRIAE_CS42_6BL_TGACv1_499617_AA1587700 | 74 | 122 | 136 | 487 | 645 | 645 | -2.4331 | 3.25E-08 | down | COG0060^amino acids such as valine, to avoid such errors it has two additional distinct tRNA(Ile)-dependent editing activities. One activity is designated as 'pretransfer' editing and involves the hydrolysis of activated Val-AMP. The other activity is designated 'posttransfer' editing and involves deacylation of mischarged Val-tRNA(Ile) (By similarity) | KEGG:ath:AT5G49030`KO:K01870 | GO:0009507^cellular_component^chloroplast`GO:0009570^cellular_component^chloroplast stroma`GO:0005739^cellular_component^mitochondrion`GO:0002161^molecular_function^aminoacyl-tRNA editing activity`GO:0005524^molecular_function^ATP binding`GO:0004822^molecular_function^isoleucine-tRNA ligase activity`GO:0046872^molecular_function^metal ion binding`GO:0000049^molecular_function^tRNA binding`GO:0006428^biological_process^isoleucyl-tRNA aminoacylation`GO:0048481^biological_process^plant ovule development | SYIM_ARATH^SYIM_ARATH^Q:160-3048,H:83-1081^68.2%ID^E:0^RecName: Full=Isoleucine--tRNA ligase, chloroplastic/mitochondrial {ECO:0000305};^Eukaryota; Viridiplantae; Streptophyta; Embryophyta; Tracheophyta; Spermatophyta; Magnoliophyta; eudicotyledons; Gunneridae; Pentapetalae; rosids; malvids; Brassicales; Brassicaceae; Camelineae; Arabidopsis |
| LOC109782252 | TRIAE_CS42_2DL_TGACv1_159111_AA0532630 | 77 | 36 | 33 | 328 | 294 | 282 | -2.64997 | 3.69E-09 | down | COG0332^Catalyzes the condensation reaction of fatty acid synthesis by the addition to an acyl acceptor of two carbons from malonyl-ACP. Catalyzes the first condensation reaction which initiates fatty acid synthesis and may therefore play a role in governing the total rate of fatty acid production. Possesses both acetoacetyl-ACP synthase and acetyl transacylase activities. Its substrate specificity determines the biosynthesis of branched- chain and or straight-chain of fatty acids (By similarity) | KEGG:ath:AT1G62640`KO:K00648 | GO:0009507^cellular_component^chloroplast`GO:0009570^cellular_component^chloroplast stroma`GO:0004315^molecular_function^3-oxoacyl-[acyl-carrier-protein] synthase activity`GO:0033818^molecular_function^beta-ketoacyl-acyl-carrier-protein synthase III activity`GO:0006633^biological_process^fatty acid biosynthetic process | FABH_ARATH^FABH_ARATH^Q:223-1215,H:69-403^69.85%ID^E:2e-164^RecName: Full=3-oxoacyl-[acyl-carrier-protein] synthase III, chloroplastic;^Eukaryota; Viridiplantae; Streptophyta; Embryophyta; Tracheophyta; Spermatophyta; Magnoliophyta; eudicotyledons; Gunneridae; Pentapetalae; rosids; malvids; Brassicales; Brassicaceae; Camelineae; Arabidopsis |
| LOC109784646 | TRIAE_CS42_1DL_TGACv1_062818_AA0220440 | 136 | 233 | 206 | 1007 | 1312 | 1519 | -2.75375 | 2.47E-10 | down | COG1346^cytolysis | KEGG:ath:AT1G32080 | GO:0009507^cellular_component^chloroplast`GO:0009941^cellular_component^chloroplast envelope`GO:0009706^cellular_component^chloroplast inner membrane`GO:0016021^cellular_component^integral component of membrane`GO:0016020^cellular_component^membrane`GO:1901974^molecular_function^glycerate transmembrane transporter activity`GO:0043879^molecular_function^glycolate transmembrane transporter activity`GO:0009658^biological_process^chloroplast organization`GO:1901975^biological_process^glycerate transmembrane transport`GO:0097339^biological_process^glycolate transmembrane transport`GO:0009853^biological_process^photorespiration | PLGG1_ARATH^PLGG1_ARATH^Q:331-1593,H:92-512^76.96%ID^E:0^RecName: Full=Plastidal glycolate/glycerate translocator 1, chloroplastic;^Eukaryota; Viridiplantae; Streptophyta; Embryophyta; Tracheophyta; Spermatophyta; Magnoliophyta; eudicotyledons; Gunneridae; Pentapetalae; rosids; malvids; Brassicales; Brassicaceae; Camelineae; Arabidopsis |
| *rps12* | AIG90451 | 117 | 218 | 192 | 1123 | 1391 | 1020 | -2.75931 | 2.36E-10 | down | . | KEGG:pop:Poptr_cp071`KO:K02950 | GO:0009507^cellular_component^chloroplast`GO:0005840^cellular_component^ribosome`GO:0015935^cellular_component^small ribosomal subunit`GO:0019843^molecular_function^rRNA binding`GO:0003735^molecular_function^structural constituent of ribosome`GO:0006412^biological_process^translation | RR12B_POPTR^RR12B_POPTR^Q:129-16,H:1-38^78.95%ID^E:1e-13^RecName: Full=30S ribosomal protein S12-B, chloroplastic;^Eukaryota; Viridiplantae; Streptophyta; Embryophyta; Tracheophyta; Spermatophyta; Magnoliophyta; eudicotyledons; Gunneridae; Pentapetalae; rosids; fabids; Malpighiales; Salicaceae; Saliceae; Populus |
| LOC109750402 | TRIAE_CS42_3AS_TGACv1_212447_AA0700930 | 31 | 46 | 38 | 184 | 339 | 264 | -2.79008 | 7.43E-10 | down | COG0652^peptidyl-prolyl cis-trans isomerase activity | KEGG:ath:AT1G74070`KO:K03768 | GO:0009507^cellular_component^chloroplast`GO:0009534^cellular_component^chloroplast thylakoid`GO:0003755^molecular_function^peptidyl-prolyl cis-trans isomerase activity`GO:0006457^biological_process^protein folding | CP26B_ARATH^CP26B_ARATH^Q:217-903,H:85-313^56.12%ID^E:2e-65^RecName: Full=Peptidyl-prolyl cis-trans isomerase CYP26-2, chloroplastic;^Eukaryota; Viridiplantae; Streptophyta; Embryophyta; Tracheophyta; Spermatophyta; Magnoliophyta; eudicotyledons; Gunneridae; Pentapetalae; rosids; malvids; Brassicales; Brassicaceae; Camelineae; Arabidopsis |
| LOC109769827 | TRIAE_CS42_2BL_TGACv1_130934_AA0420140 | 11 | 8 | 6 | 72 | 66 | 38 | -2.81412 | 8.13E-08 | down | COG1154^Catalyzes the acyloin condensation reaction between C atoms 2 and 3 of pyruvate and glyceraldehyde 3-phosphate to yield 1-deoxy-D-xylulose-5-phosphate (DXP) (By similarity) | KEGG:osa:4342614`KO:K01662 | GO:0009507^cellular_component^chloroplast`GO:0008661^molecular_function^1-deoxy-D-xylulose-5-phosphate synthase activity`GO:0046872^molecular_function^metal ion binding`GO:0052865^biological_process^1-deoxy-D-xylulose 5-phosphate biosynthetic process`GO:0016114^biological_process^terpenoid biosynthetic process`GO:0009228^biological_process^thiamine biosynthetic process | DXS2_ORYSJ^DXS2_ORYSJ^Q:52-2136,H:19-712^88.35%ID^E:0^RecName: Full=Probable 1-deoxy-D-xylulose-5-phosphate synthase 2, chloroplastic;^Eukaryota; Viridiplantae; Streptophyta; Embryophyta; Tracheophyta; Spermatophyta; Magnoliophyta; Liliopsida; Poales; Poaceae; BOP clade; Oryzoideae; Oryzeae; Oryzinae; Oryza; Oryza sativa |
| LOC109776509 | TRIAE_CS42_3B_TGACv1_226191_AA0814620 | 40 | 26 | 28 | 334 | 163 | 168 | -2.83161 | 6.17E-10 | down | ENOG4111GA1^Domain of unknown function (DUF3411) | KEGG:ath:AT3G08640 | GO:0009507^cellular_component^chloroplast`GO:0009941^cellular_component^chloroplast envelope`GO:0031969^cellular_component^chloroplast membrane`GO:0016021^cellular_component^integral component of membrane`GO:0009536^cellular_component^plastid`GO:0009793^biological_process^embryo development ending in seed dormancy`GO:0048366^biological_process^leaf development`GO:0009648^biological_process^photoperiodism`GO:0000302^biological_process^response to reactive oxygen species | RER3_ARATH^RER3_ARATH^Q:1-690,H:103-336^65.81%ID^E:4e-101^RecName: Full=Protein RETICULATA-RELATED 3, chloroplastic {ECO:0000303\|PubMed:23596191};^Eukaryota; Viridiplantae; Streptophyta; Embryophyta; Tracheophyta; Spermatophyta; Magnoliophyta; eudicotyledons; Gunneridae; Pentapetalae; rosids; malvids; Brassicales; Brassicaceae; Camelineae; Arabidopsis |
| LOC109733901 | TRIAE_CS42_2BL_TGACv1_130048_AA0402360 | 14 | 13 | 8 | 109 | 97 | 96 | -3.11325 | 3.06E-10 | down | ENOG410YAIP^plastid division protein CDP1, chloroplastic-like | KEGG:ath:AT3G19180 | GO:0009507^cellular_component^chloroplast`GO:0009706^cellular_component^chloroplast inner membrane`GO:0016021^cellular_component^integral component of membrane`GO:0009528^cellular_component^plastid inner membrane`GO:0043621^molecular_function^protein self-association`GO:0010020^biological_process^chloroplast fission`GO:0043572^biological_process^plastid fission | CDP1_ARATH^CDP1_ARATH^Q:88-1995,H:181-816^50.84%ID^E:3e-171^RecName: Full=Plastid division protein CDP1, chloroplastic;^Eukaryota; Viridiplantae; Streptophyta; Embryophyta; Tracheophyta; Spermatophyta; Magnoliophyta; eudicotyledons; Gunneridae; Pentapetalae; rosids; malvids; Brassicales; Brassicaceae; Camelineae; Arabidopsis |
| LOC109773104 | TRIAE_CS42_7AL_TGACv1_556453_AA1763350 | 26 | 34 | 39 | 338 | 349 | 347 | -3.3939 | 1.02E-13 | down | COG2217^p-type ATPase | KEGG:ath:AT4G37270 | GO:0009507^cellular_component^chloroplast`GO:0009941^cellular_component^chloroplast envelope`GO:0009706^cellular_component^chloroplast inner membrane`GO:0016021^cellular_component^integral component of membrane`GO:0009536^cellular_component^plastid`GO:0005524^molecular_function^ATP binding`GO:0016887^molecular_function^ATPase activity`GO:0008551^molecular_function^cadmium-exporting ATPase activity`GO:0046872^molecular_function^metal ion binding`GO:0016463^molecular_function^zinc-exporting ATPase activity`GO:0015633^molecular_function^zinc-transporting ATPase activity`GO:0006878^biological_process^cellular copper ion homeostasis`GO:0009642^biological_process^response to light intensity`GO:0055069^biological_process^zinc ion homeostasis | HMA1_ARATH^HMA1_ARATH^Q:1-1197,H:406-804^71.68%ID^E:2e-159^RecName: Full=Probable cadmium/zinc-transporting ATPase HMA1, chloroplastic;^Eukaryota; Viridiplantae; Streptophyta; Embryophyta; Tracheophyta; Spermatophyta; Magnoliophyta; eudicotyledons; Gunneridae; Pentapetalae; rosids; malvids; Brassicales; Brassicaceae; Camelineae; Arabidopsis |
| LOC109767458 | TRIAE_CS42_5DL_TGACv1_433388_AA1411710 | 41 | 14 | 23 | 566 | 503 | 582 | -4.4174 | 5.55E-21 | down | COG0465^Acts as a processive, ATP-dependent zinc metallopeptidase for both cytoplasmic and membrane proteins. Plays a role in the quality control of integral membrane proteins (By similarity) | KEGG:osa:4326311`KO:K08955 | GO:0009941^cellular_component^chloroplast envelope`GO:0016020^cellular_component^membrane`GO:0005739^cellular_component^mitochondrion`GO:0005524^molecular_function^ATP binding`GO:0004176^molecular_function^ATP-dependent peptidase activity`GO:0046872^molecular_function^metal ion binding`GO:0004222^molecular_function^metalloendopeptidase activity`GO:0008237^molecular_function^metallopeptidase activity`GO:0006508^biological_process^proteolysis | FTSH5_ORYSJ^FTSH5_ORYSJ^Q:1-2007,H:1-677^81.59%ID^E:0^RecName: Full=ATP-dependent zinc metalloprotease FTSH 5, mitochondrial;^Eukaryota; Viridiplantae; Streptophyta; Embryophyta; Tracheophyta; Spermatophyta; Magnoliophyta; Liliopsida; Poales; Poaceae; BOP clade; Oryzoideae; Oryzeae; Oryzinae; Oryza; Oryza sativa |
| LOC109785372 | TRIAE_CS42_7AL_TGACv1_556691_AA1768950 | 5 | 10 | 7 | 175 | 199 | 238 | -4.78964 | 1.25E-20 | down | . | . | GO:0009507^cellular_component^chloroplast`GO:0005524^molecular_function^ATP binding`GO:0043295^molecular_function^glutathione binding`GO:0004363^molecular_function^glutathione synthase activity`GO:0000287^molecular_function^magnesium ion binding`GO:0042803^molecular_function^protein homodimerization activity | GSHB_BRAJU^GSHB_BRAJU^Q:232-1584,H:68-522^61.32%ID^E:0^RecName: Full=Glutathione synthetase, chloroplastic;^Eukaryota; Viridiplantae; Streptophyta; Embryophyta; Tracheophyta; Spermatophyta; Magnoliophyta; eudicotyledons; Gunneridae; Pentapetalae; rosids; malvids; Brassicales; Brassicaceae; Brassiceae; Brassica |
| LOC109779367 | TRIAE_CS42_1BL_TGACv1_033291_AA0138510 | 17 | 13 | 14 | 434 | 518 | 847 | -5.36036 | 2.61E-27 | down | . | . | GO:0009507^cellular_component^chloroplast`GO:0003824^molecular_function^catalytic activity | YCF23_PYRYE^YCF23_PYRYE^Q:160-888,H:11-255^36.59%ID^E:2e-47^RecName: Full=Uncharacterized protein ycf23;^Eukaryota; Rhodophyta; Bangiophyceae; Bangiales; Bangiaceae; Pyropia |
