# Supplementary material for: A cosmopolitan fungal pathogen of dicots adopts an endophytic lifestyle on cereal crops and protects them from major fungal diseases
Source: ISME J. 2020 Aug 19;14(12):3120–35. doi: 10.1038/s41396-020-00744-6 (PMC7784893; doi:10.1038/s41396-020-00744-6)
Supplement: Supplementary file 7 — Supplementary Table 7 [file 41396_2020_744_MOESM7_ESM.docx]

**Supplementary Table 7** Wheat DEGs associated with the chloroplast inner membrane and chloroplast starch grain in DT-8 treated and control wheat spikes

| **gene** | **gene_id** | **DT-8 Sample 1_count** | **DT-8 Sample 2_count** | **DT-8 Sample 3_count** | **Control Sample 1_count** | **Control Sample 2_count** | **Control Sample 3_count** | **logFC** | **FDR** | **exp** | **eggnog** | **Kegg** | **GO** | **uniprot_hit** |
| --- | --- | --- | --- | --- | --- | --- | --- | --- | --- | --- | --- | --- | --- | --- |
| LOC109745942 | TRIAE_CS42_7BS_TGACv1_592411_AA1937580 | 926 | 999 | 1066 | 7 | 13 | 13 | 6.458033 | 1.62E-35 | up | COG4638^rieske 2fe-2S domain-containing protein | KEGG:ath:AT4G25650 | GO:0009507^cellular_component^chloroplast`GO:0009941^cellular_component^chloroplast envelope`GO:0009706^cellular_component^chloroplast inner membrane`GO:0016021^cellular_component^integral component of membrane`GO:0009536^cellular_component^plastid`GO:0051537^molecular_function^2 iron, 2 sulfur cluster binding`GO:0010277^molecular_function^chlorophyllide a oxygenase [overall] activity`GO:0046872^molecular_function^metal ion binding`GO:0015031^biological_process^protein transport | PTC52_ARATH^PTC52_ARATH^Q:190-1506,H:73-557^48.48%ID^E:1e-160^RecName: Full=Protochlorophyllide-dependent translocon component 52, chloroplastic;^Eukaryota; Viridiplantae; Streptophyta; Embryophyta; Tracheophyta; Spermatophyta; Magnoliophyta; eudicotyledons; Gunneridae; Pentapetalae; rosids; malvids; Brassicales; Brassicaceae; Camelineae; Arabidopsis |
| LOC109755686 | TRIAE_CS42_4BS_TGACv1_328584_AA1090480 | 271 | 249 | 234 | 8 | 7 | 5 | 5.182797 | 3.02E-23 | up | . | . | GO:0009706^cellular_component^chloroplast inner membrane`GO:0005516^molecular_function^calmodulin binding`GO:0016491^molecular_function^oxidoreductase activity`GO:0015031^biological_process^protein transport | TIC32_PEA^TIC32_PEA^Q:10-942,H:2-313^66.35%ID^E:2e-144^RecName: Full=Short-chain dehydrogenase TIC 32, chloroplastic;^Eukaryota; Viridiplantae; Streptophyta; Embryophyta; Tracheophyta; Spermatophyta; Magnoliophyta; eudicotyledons; Gunneridae; Pentapetalae; rosids; fabids; Fabales; Fabaceae; Papilionoideae; Fabeae; Pisum |
| LOC100832197 | TRIAE_CS42_4BS_TGACv1_329699_AA1103360 | 1258 | 708 | 935 | 82 | 69 | 73 | 3.667417 | 2.84E-16 | up | ENOG410XPWC^solute carrier family 17 | KEGG:osa:4347913 | GO:0009706^cellular_component^chloroplast inner membrane`GO:0016021^cellular_component^integral component of membrane`GO:0009536^cellular_component^plastid`GO:0005315^molecular_function^inorganic phosphate transmembrane transporter activity`GO:0015229^molecular_function^L-ascorbic acid transporter activity`GO:0098656^biological_process^anion transmembrane transport`GO:0010028^biological_process^xanthophyll cycle | PHT44_ORYSJ^PHT44_ORYSJ^Q:190-1698,H:66-561^81.18%ID^E:0^RecName: Full=Probable anion transporter 4, chloroplastic;^Eukaryota; Viridiplantae; Streptophyta; Embryophyta; Tracheophyta; Spermatophyta; Magnoliophyta; Liliopsida; Poales; Poaceae; BOP clade; Oryzoideae; Oryzeae; Oryzinae; Oryza; Oryza sativa |
| LOC109738799 | TRIAE_CS42_4DL_TGACv1_343749_AA1139150 | 2082 | 1095 | 1138 | 207 | 201 | 235 | 2.719851 | 5.44E-10 | up | COG4638^rieske 2fe-2S domain-containing protein | KEGG:ath:AT3G44880`KO:K13071 | GO:0009507^cellular_component^chloroplast`GO:0009941^cellular_component^chloroplast envelope`GO:0009706^cellular_component^chloroplast inner membrane`GO:0009534^cellular_component^chloroplast thylakoid`GO:0009535^cellular_component^chloroplast thylakoid membrane`GO:0051537^molecular_function^2 iron, 2 sulfur cluster binding`GO:0010277^molecular_function^chlorophyllide a oxygenase [overall] activity`GO:0051536^molecular_function^iron-sulfur cluster binding`GO:0046872^molecular_function^metal ion binding`GO:0032441^molecular_function^pheophorbide a oxygenase activity`GO:0008219^biological_process^cell death`GO:0015996^biological_process^chlorophyll catabolic process`GO:0009816^biological_process^defense response to bacterium, incompatible interaction`GO:0009908^biological_process^flower development`GO:0010154^biological_process^fruit development | PAO_ARATH^PAO_ARATH^Q:145-1605,H:48-537^72.65%ID^E:0^RecName: Full=Pheophorbide a oxygenase, chloroplastic;^Eukaryota; Viridiplantae; Streptophyta; Embryophyta; Tracheophyta; Spermatophyta; Magnoliophyta; eudicotyledons; Gunneridae; Pentapetalae; rosids; malvids; Brassicales; Brassicaceae; Camelineae; Arabidopsis |
| LOC109783704 | TRIAE_CS42_4AL_TGACv1_289623_AA0974220 | 1899 | 1087 | 1237 | 336 | 362 | 393 | 1.926056 | 1.93E-05 | up | ENOG410XPWC^solute carrier family 17 | KEGG:osa:4347913 | GO:0009706^cellular_component^chloroplast inner membrane`GO:0016021^cellular_component^integral component of membrane`GO:0009536^cellular_component^plastid`GO:0005315^molecular_function^inorganic phosphate transmembrane transporter activity`GO:0015229^molecular_function^L-ascorbic acid transporter activity`GO:0098656^biological_process^anion transmembrane transport`GO:0010028^biological_process^xanthophyll cycle | PHT44_ORYSJ^PHT44_ORYSJ^Q:208-1830,H:55-591^81.97%ID^E:0^RecName: Full=Probable anion transporter 4, chloroplastic;^Eukaryota; Viridiplantae; Streptophyta; Embryophyta; Tracheophyta; Spermatophyta; Magnoliophyta; Liliopsida; Poales; Poaceae; BOP clade; Oryzoideae; Oryzeae; Oryzinae; Oryza; Oryza sativa |
| LOC109773061 | TRIAE_CS42_1DL_TGACv1_061488_AA0196780 | 26 | 50 | 50 | 137 | 114 | 134 | -1.6328 | 0.00114 | down | ENOG411126M^Dual specificity phosphatase, catalytic domain | KEGG:ath:AT3G01510 | GO:0009507^cellular_component^chloroplast`GO:0009569^cellular_component^chloroplast starch grain`GO:0009570^cellular_component^chloroplast stroma`GO:0043036^cellular_component^starch grain`GO:0019203^molecular_function^carbohydrate phosphatase activity`GO:0008138^molecular_function^protein tyrosine/serine/threonine phosphatase activity`GO:0005983^biological_process^starch catabolic process | LSF1_ARATH^LSF1_ARATH^Q:226-1788,H:71-590^60.99%ID^E:0^RecName: Full=Phosphoglucan phosphatase LSF1, chloroplastic;^Eukaryota; Viridiplantae; Streptophyta; Embryophyta; Tracheophyta; Spermatophyta; Magnoliophyta; eudicotyledons; Gunneridae; Pentapetalae; rosids; malvids; Brassicales; Brassicaceae; Camelineae; Arabidopsis |
| AA0138870 | TRIAE_CS42_1BL_TGACv1_033345_AA0138870 | 40 | 56 | 76 | 185 | 196 | 261 | -1.92265 | 4.50E-05 | down | ENOG411126M^Dual specificity phosphatase, catalytic domain | KEGG:ath:AT3G01510 | GO:0009507^cellular_component^chloroplast`GO:0009569^cellular_component^chloroplast starch grain`GO:0009570^cellular_component^chloroplast stroma`GO:0043036^cellular_component^starch grain`GO:0019203^molecular_function^carbohydrate phosphatase activity`GO:0008138^molecular_function^protein tyrosine/serine/threonine phosphatase activity`GO:0005983^biological_process^starch catabolic process | LSF1_ARATH^LSF1_ARATH^Q:220-1782,H:71-590^60.8%ID^E:0^RecName: Full=Phosphoglucan phosphatase LSF1, chloroplastic;^Eukaryota; Viridiplantae; Streptophyta; Embryophyta; Tracheophyta; Spermatophyta; Magnoliophyta; eudicotyledons; Gunneridae; Pentapetalae; rosids; malvids; Brassicales; Brassicaceae; Camelineae; Arabidopsis |
| LOC109773104 | TRIAE_CS42_1DL_TGACv1_062818_AA0220440 | 93 | 41 | 38 | 274 | 338 | 340 | -2.49281 | 3.78E-08 | down | COG1346^cytolysis | KEGG:ath:AT1G32080 | GO:0009507^cellular_component^chloroplast`GO:0009941^cellular_component^chloroplast envelope`GO:0009706^cellular_component^chloroplast inner membrane`GO:0016021^cellular_component^integral component of membrane`GO:0016020^cellular_component^membrane`GO:1901974^molecular_function^glycerate transmembrane transporter activity`GO:0043879^molecular_function^glycolate transmembrane transporter activity`GO:0009658^biological_process^chloroplast organization`GO:1901975^biological_process^glycerate transmembrane transport`GO:0097339^biological_process^glycolate transmembrane transport`GO:0009853^biological_process^photorespiration | PLGG1_ARATH^PLGG1_ARATH^Q:331-1593,H:92-512^76.96%ID^E:0^RecName: Full=Plastidal glycolate/glycerate translocator 1, chloroplastic;^Eukaryota; Viridiplantae; Streptophyta; Embryophyta; Tracheophyta; Spermatophyta; Magnoliophyta; eudicotyledons; Gunneridae; Pentapetalae; rosids; malvids; Brassicales; Brassicaceae; Camelineae; Arabidopsis |
| LOC109784646 | TRIAE_CS42_2BL_TGACv1_130048_AA0402360 | 17 | 27 | 17 | 132 | 141 | 117 | -2.69477 | 1.85E-08 | down | ENOG410YAIP^plastid division protein CDP1, chloroplastic-like | KEGG:ath:AT3G19180 | GO:0009507^cellular_component^chloroplast`GO:0009706^cellular_component^chloroplast inner membrane`GO:0016021^cellular_component^integral component of membrane`GO:0009528^cellular_component^plastid inner membrane`GO:0043621^molecular_function^protein self-association`GO:0010020^biological_process^chloroplast fission`GO:0043572^biological_process^plastid fission | CDP1_ARATH^CDP1_ARATH^Q:88-1995,H:181-816^50.84%ID^E:3e-171^RecName: Full=Plastid division protein CDP1, chloroplastic;^Eukaryota; Viridiplantae; Streptophyta; Embryophyta; Tracheophyta; Spermatophyta; Magnoliophyta; eudicotyledons; Gunneridae; Pentapetalae; rosids; malvids; Brassicales; Brassicaceae; Camelineae; Arabidopsis |
| LOC109733901 | TRIAE_CS42_7AL_TGACv1_556453_AA1763350 | 28 | 16 | 16 | 229 | 213 | 215 | -3.46963 | 1.86E-13 | down | COG2217^p-type ATPase | KEGG:ath:AT4G37270 | GO:0009507^cellular_component^chloroplast`GO:0009941^cellular_component^chloroplast envelope`GO:0009706^cellular_component^chloroplast inner membrane`GO:0016021^cellular_component^integral component of membrane`GO:0009536^cellular_component^plastid`GO:0005524^molecular_function^ATP binding`GO:0016887^molecular_function^ATPase activity`GO:0008551^molecular_function^cadmium-exporting ATPase activity`GO:0046872^molecular_function^metal ion binding`GO:0016463^molecular_function^zinc-exporting ATPase activity`GO:0015633^molecular_function^zinc-transporting ATPase activity`GO:0006878^biological_process^cellular copper ion homeostasis`GO:0009642^biological_process^response to light intensity`GO:0055069^biological_process^zinc ion homeostasis | HMA1_ARATH^HMA1_ARATH^Q:1-1197,H:406-804^71.68%ID^E:2e-159^RecName: Full=Probable cadmium/zinc-transporting ATPase HMA1, chloroplastic;^Eukaryota; Viridiplantae; Streptophyta; Embryophyta; Tracheophyta; Spermatophyta; Magnoliophyta; eudicotyledons; Gunneridae; Pentapetalae; rosids; malvids; Brassicales; Brassicaceae; Camelineae; Arabidopsis |
