# Supplementary material for: A cosmopolitan fungal pathogen of dicots adopts an endophytic lifestyle on cereal crops and protects them from major fungal diseases
Source: ISME J. 2020 Aug 19;14(12):3120–35. doi: 10.1038/s41396-020-00744-6 (PMC7784893; doi:10.1038/s41396-020-00744-6)
Supplement: Supplementary file 8 — Supplementary Figures legends [file 41396_2020_744_MOESM8_ESM.docx]

**Supplementary Figures legends**

**Supplementary Fig. 1 *S. sclerotiorum* grows in wheat but does not kill the plants**. **a** and **b** Wheat growth stage of sampling of winter wheat growing under natural field conditions in Dangyang County, Hubei province. **c** Detection of *S. sclerotiorum* in wheat seedlings by PCR amplification using specific primers described by Qin L et al [24]. **d** Wheat seedlings growing after inoculation with hyphae of strain DT-8 at the seedling-emergence stage; red arrows indicate white *S. sclerotiorum* colonies around seedlings; the photograph was taken at 15 dpi. **e** Virulent strain DT-8VF could kill rapeseed, but not wheat seedlings; the hypovirulent DT-8 strain could kill neither wheat seedling nor rapeseed seedlings. 14-day-old wheat seedlings and 7-day-old rapeseed seedlings had been transferred to half MS agar medium in square plates (12 × 12 cm) and inoculated with strains DT-8VF and DT-8 (6 dpi).

**Supplementary Fig. 2 *S. sclerotiorum growing* endophytically in wheat roots visualized with transmission electron microscopy.** **a** Hyphae of strain DT-8VF in root cells and a hypha crossing the plant cell walls (red arrow). **b** Enlargement of the marked region (red arrow) in (a) showing the broken plant cell wall. **c** Hyphae of strain DT-8VF in the intracellular and intercellular spaces of roots; **d** Enlargement of (c) showing the substance deposited around the fungal cell wall (dark arrow) and a potential membrane surrounding the fungal cell wall (red arrow); **e** and **f** Hyphae of strain DT-8 in root cells. *hy*, fungal hyphae; PC, plant cell; FCW, fungal cell wall; PCW, plant cell wall.

**Supplementary Fig. 3 The root of non-treated wheat seedling visualized with scanning electron microscopy.** **a** No hyphae could be observed in the non-treated roots; **b** Enlargement of the marked region (red boxed regions) in (a).

**Supplementary Fig. 4 Various mycovirus-mediated hypovirulent strains of *S. sclerotiorum* could grow endophytically in wheat and promote wheat plant growth.** **a** Hypovirulent strains AH98. SCH941 and T1-1-20 could kill neither wheat nor rapeseed, while a virulent strain Ep-1PNA367 could kill rapeseed, but not kill wheat. 14-day-old wheat seedlings and 7-day-old rapeseed seedlings were transferred to half MS agar medium in square plates (12 × 12 cm) and inoculated with strains for 6 dpi. **b** Hyphae of all tested strains could be observed in wheat roots visualized by scanning electron microscopy. Lower panels, images enlarged from the marked regions of upper panels. **c** Hyphae of all tested *S. sclerotiorum* strains growing endophytically in wheat roots visualized with by transmission electron microscopy. **d** and **e** All tested strains could promote the growth of wheat seedlings. Photographs taken and shoot fresh weights measured at 25 dpi (*t*-test, *p* < 0.01) (n = 5). *hy* indicates hyphae. Error bars indicate standard deviation and different letters indicate significant differences.

**Supplementary Fig. 5 *S. sclerotiorum* colonized wheat stems from an inoculated wheat root. a** Upper panels, confocal microscopy images showing that mCherry (red) could be detected in wheat stems; right, images enlarged from the red boxes on the left; lower panels, images of non-inoculated wheat obtained under the same observation conditions. Wheat was inoculated with strain DT-8VF^RFP^ and allowed to grow for 45 days before sampling. Scale bars, 20 μm for the main images and 10 μm for enlarged images. **b** The strain DT-8VF^RFP^ of *S. sclerotiorum* was recovered on hygromycin-amended PDA plates (50 µg/ml) from the stalk of a DT-8VF^RFP^-inoculated wheat plant after 45 days growth in soil growing greenhouse, Photograph was taken after 8 days incubation on hygromycin-amended PDA medium. **c** Examination of four emerging colonies of *S. sclerotiorum* (b) by PCR amplification using specific primers described by Qin L et al [24]. Lane M, DNA size markers; lanes 1–4, DNA samples from emerging colonies on hygromycin-amended PDA medium from wheat stems; lane 5, negative control; lane 6, DNA from *S. sclerotiorum* (positive control).

**Supplementary Fig. 6 Detection of *S. sclerotiorum* and mycovirus DNA in DT-8 treated wheat plants by PCR amplification**. **a** wheat roots; **b** flag leaves; and **c** spike of DT-8 treated wheat plants from the field during the filling stage. The root, flag leaf and spike which shared the same sample number were taken from the same wheat plant.

**Supplementary Fig. 7 *S*. *sclerotiorum* growing endophytically in roots of oat and maize.** **a** Hyphae in strain DT-8-treated root of oat. **b** Enlargement of the regions in dotted boxes in (a) showed hyphae in the intercellular and intracellular spaces. **c** Hyphae in strain DT-8-treated root of maize. **d** Enlargement of the regions in dotted boxes in (c); *hy* indicates hyphae, arrows indicate hyphae in root cells. Images were visualized by scanning electron microscopy.

**Supplementary Fig. 8** **Gene Ontology (GO) enrichment analyses in wheat spike and flag leaves responding to the endophytic growth of *S. sclerotiorum*.** **a** The graphs show the biological process analysis of Gene Ontology (GO) in spikes (*p* < 0.01). The ‘defense response’ (GO: 0006952) related to disease resistance pathways was significantly enriched. **b** The graphs show the biological process analysis of Gene Ontology (GO) in flag leaves (*p* < 0.01). The ‘defense response’ (GO: 0006952) related to disease resistance pathways was significantly enriched. **c** The graphs show the cellular component analysis of Gene Ontology (GO) in flag leaves (*p* < 0.01). “Chloroplast” (GO: 0009507) and “chloroplast inner membrane” (GO: 0009706) were significantly enriched. **d** The graphs show the cellular component analysis of Gene Ontology (GO) in wheat spikes (*p* < 0.01). “Chloroplast inner membrane” (GO: 0009706) and “chloroplast starch grain” (GO: 0009569) were significantly enriched in spikes.

**Supplementary Fig. 9 Disease resistance-associated responses in wheat flag leaves harboring endophytic *S. sclerotiorum*.** **a** Schematic diagram of wheat resistance pathways enriched in the GO analysis. The ‘defense response’ (GO: 0006952) related to disease resistance pathways was significantly enriched. The number in the top bracket in each box is a *p-*value and the lower bracket in the same box shows the number of enriched genes and the total genes in the pathway. The *p*-value in the dark-colored box is lower than those in light-colored boxes. **b** Heat map showing the transcriptional profiles of 89 wheat genes that were associated with defense response in wheat flag leaves from DT-8-treated and control-treated plants grown under natural conditions (|log2FC| >1, FDR < 0.01). Significantly regulated genes are represented in colors, red for up-regulated genes and green for down-regulated genes. All genes' gene_id were listed in Supplementary Table 4, genes labeled with "*" do not have common name. **c** and **d** PTI and Effector-Triggered Immunity (ETI) pathway-related genes were enriched following KEGG enrichment analysis. **e** Salicylic acid (SA), Abscisic acid (ABA), and Jasmonic acid (JA) pathway-related genes enriched following KEGG analysis. **f** and **g** Determination of JA and ABA content in flag leaves (*t*-test, *p* < 0.01) (n = 6). Error bars indicate standard deviation and different letters indicate significant differences.

**Supplementary Fig. 10 Growth-associated processes in flag leaves harboring endophytic *S. sclerotiorum*.** **a** Schematic diagram of wheat growth-associated processes enriched in the GO analysis. “Chloroplast” (GO: 0009507) and “chloroplast inner membrane” (GO: 0009706) were significantly enriched. The number in the upper bracket in each box is the *P*-value, and the lower bracket in the same box shows the number of enriched genes and the total genes in the pathway. The *p*-values in the dark-colored boxes are lower than those in light-colored boxes. **b** Heat map showing the transcriptional profiles of 94 wheat genes that were associated with the chloroplast in strain DT-8-treated and control wheat flag leaves from plants grown under natural conditions (|log2FC| >1, FDR < 0.01). All genes' gene_id were listed in Supplementary Table 6 genes labeled with "*" do not have common name.. Significantly up-regulated genes are highlighted in red and down-regulated genes in green. **c** and **d** Determination of the photosynthetic rate (n = 3) and chlorophyll content (n = 4) in the flag leaves (*t*-test, *p* < 0.01). Error bars indicate standard deviation and different letters indicate significant differences.

**Supplementary Fig. 11 Growth-associated processes in wheat spikes harboring endophytic *S. sclerotiorum*.** **a** Schematic diagram of wheat growth-associated processes enriched in the GO analysis. “Chloroplast inner membrane” (GO: 0009706) and “chloroplast starch grain” (GO: 0009569) were significantly enriched. The number in the upper bracket in each box is the *p*-value, and the lower bracket in the same box shows the number of enriched genes and the total genes in the pathway. The *p*-values in the dark-colored boxes are lower than those in light-colored boxes. **b** Heat map showing the transcriptional profiles of 9 wheat genes that were associated with the wheat chloroplast inner membrane, and chloroplast starch grain in DT-8-treated and control wheat spikes from plants grown under natural conditions (|log2FC| >1, FDR < 0.01). Significantly up-regulated genes are highlighted in red and down-regulated genes in green. All genes' gene_id were listed in Supplementary Table 7, genes labeled with "*" do not have common name.**c** Indole-3-acetic acid (IAA) pathways related genes enriched by KEGG analysis. **d** Determination of IAA content in the flag leaves (*t*-test, *p* < 0.01) (n = 6). Error bars indicate standard deviation and different letters indicate significant differences.
