# Supplementary figures and images for: A cosmopolitan fungal pathogen of dicots adopts an endophytic lifestyle on cereal crops and protects them from major fungal diseases
Source: ISME J. 2020 Aug 19;14(12):3120–35. doi: 10.1038/s41396-020-00744-6 (PMC7784893; doi:10.1038/s41396-020-00744-6)

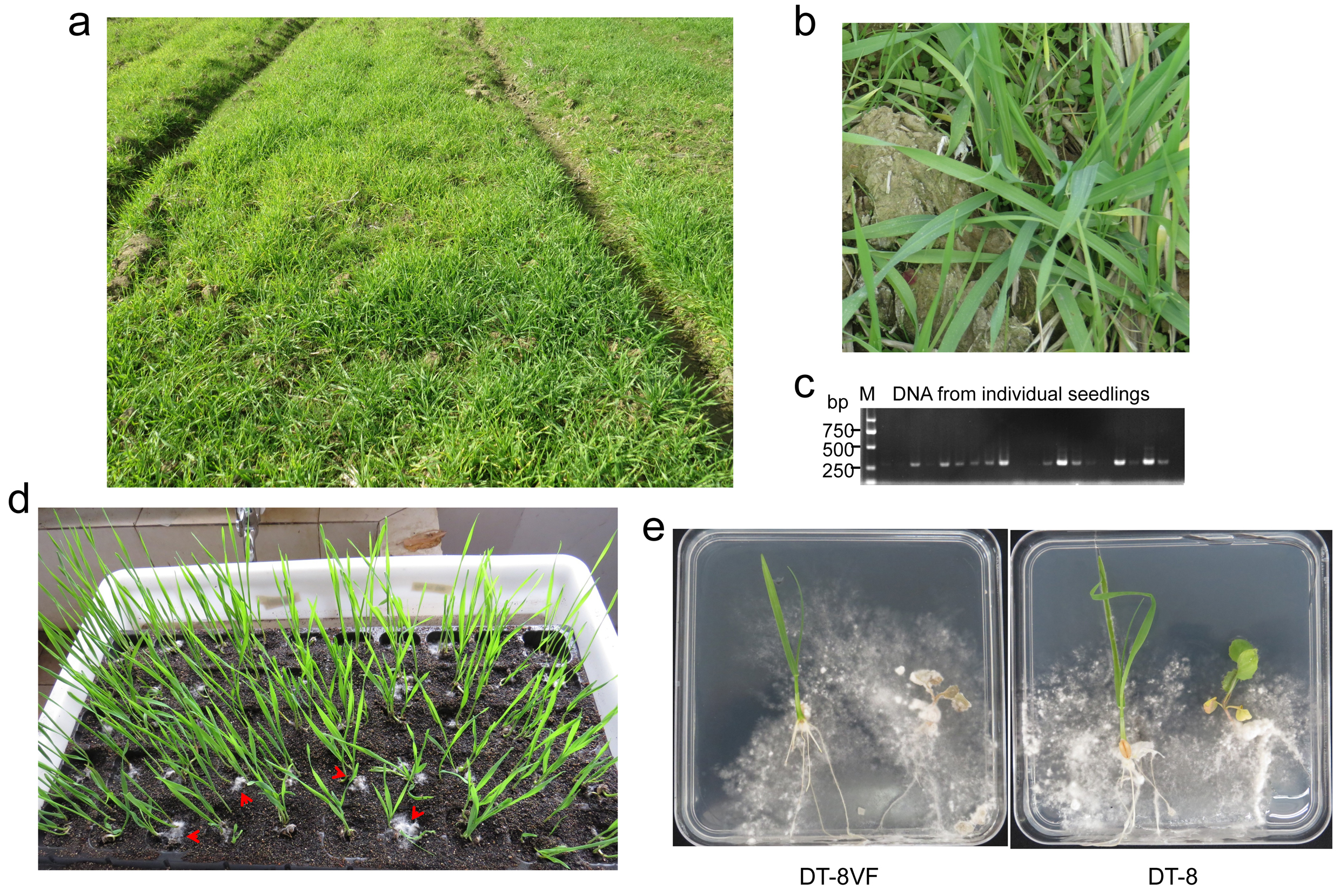

Supplement: Supplementary file 9 — Supplementary Fig. 1 [file 41396_2020_744_MOESM9_ESM.jpg]

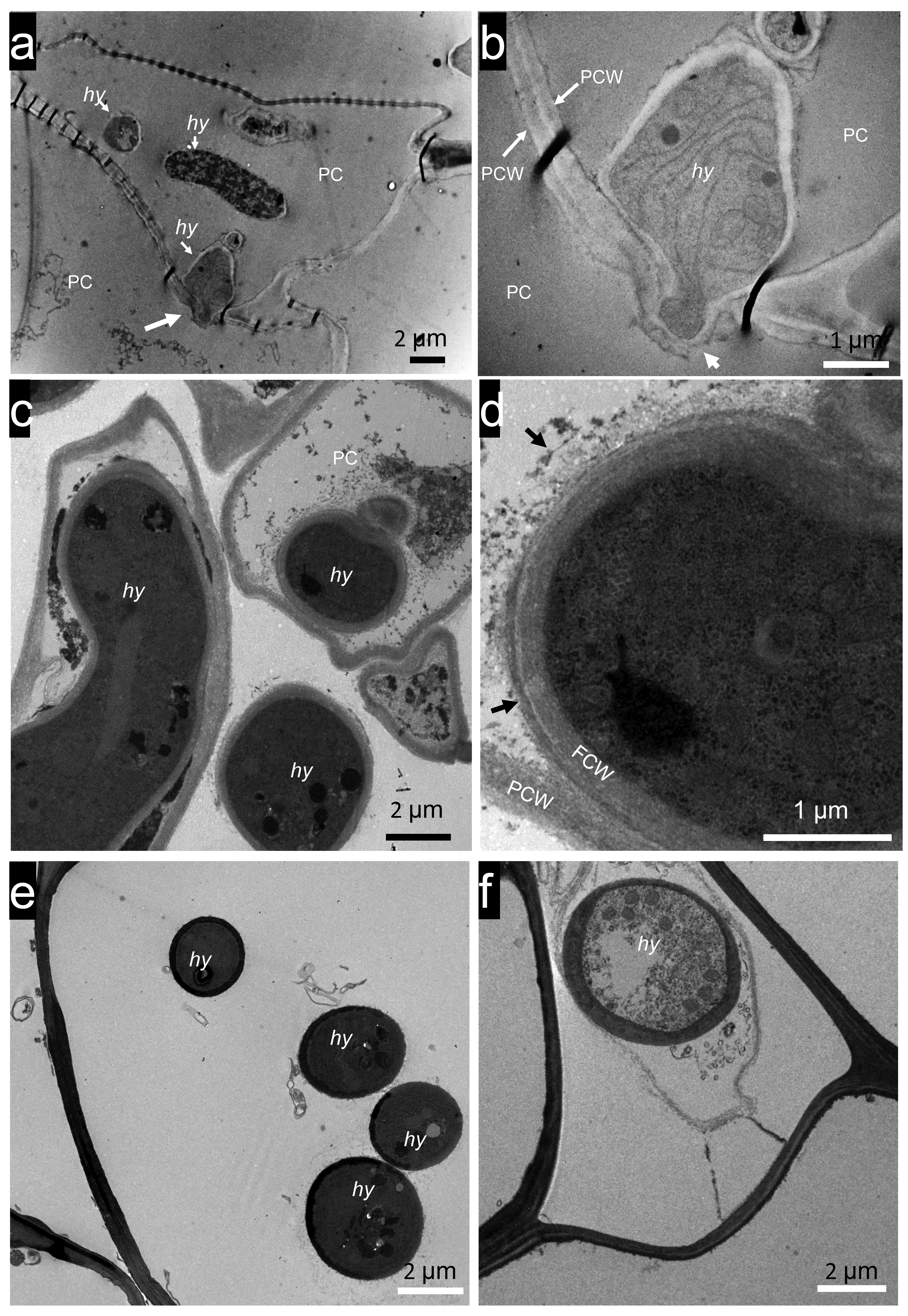

Supplement: Supplementary file 10 — Supplementary Fig. 2 [file 41396_2020_744_MOESM10_ESM.jpg]

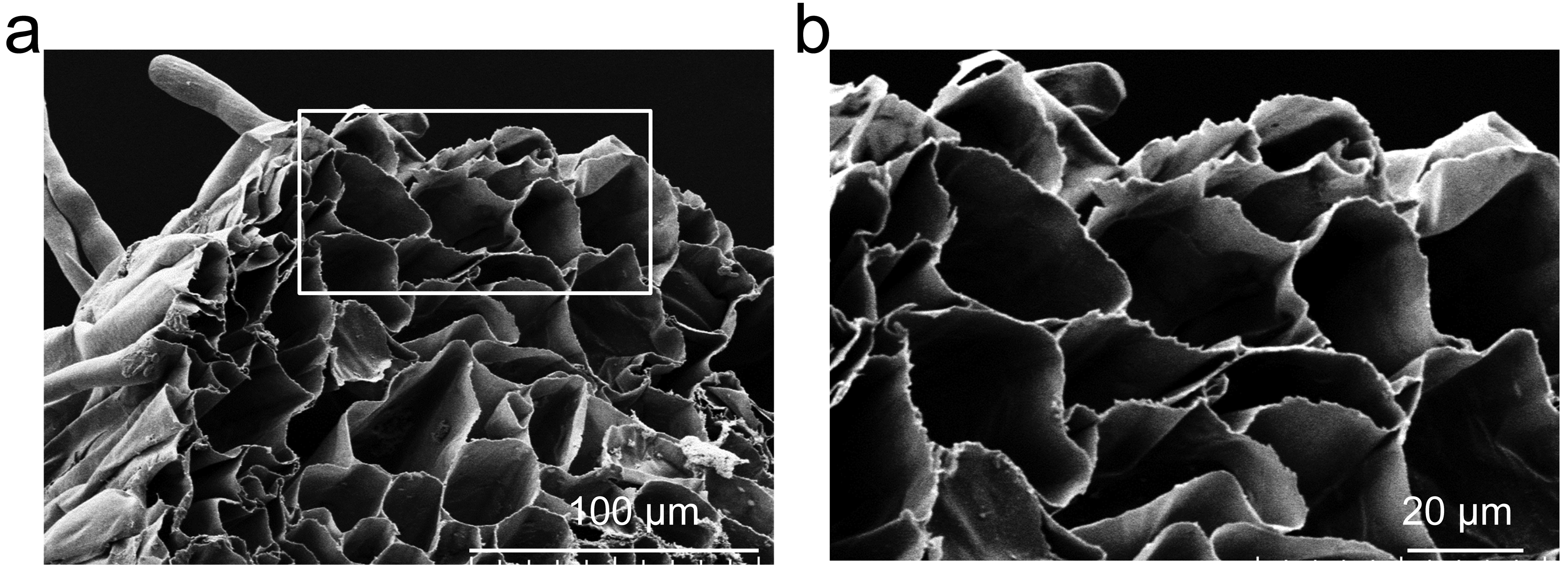

Supplement: Supplementary file 11 — Supplementary Fig. 3 [file 41396_2020_744_MOESM11_ESM.jpg]

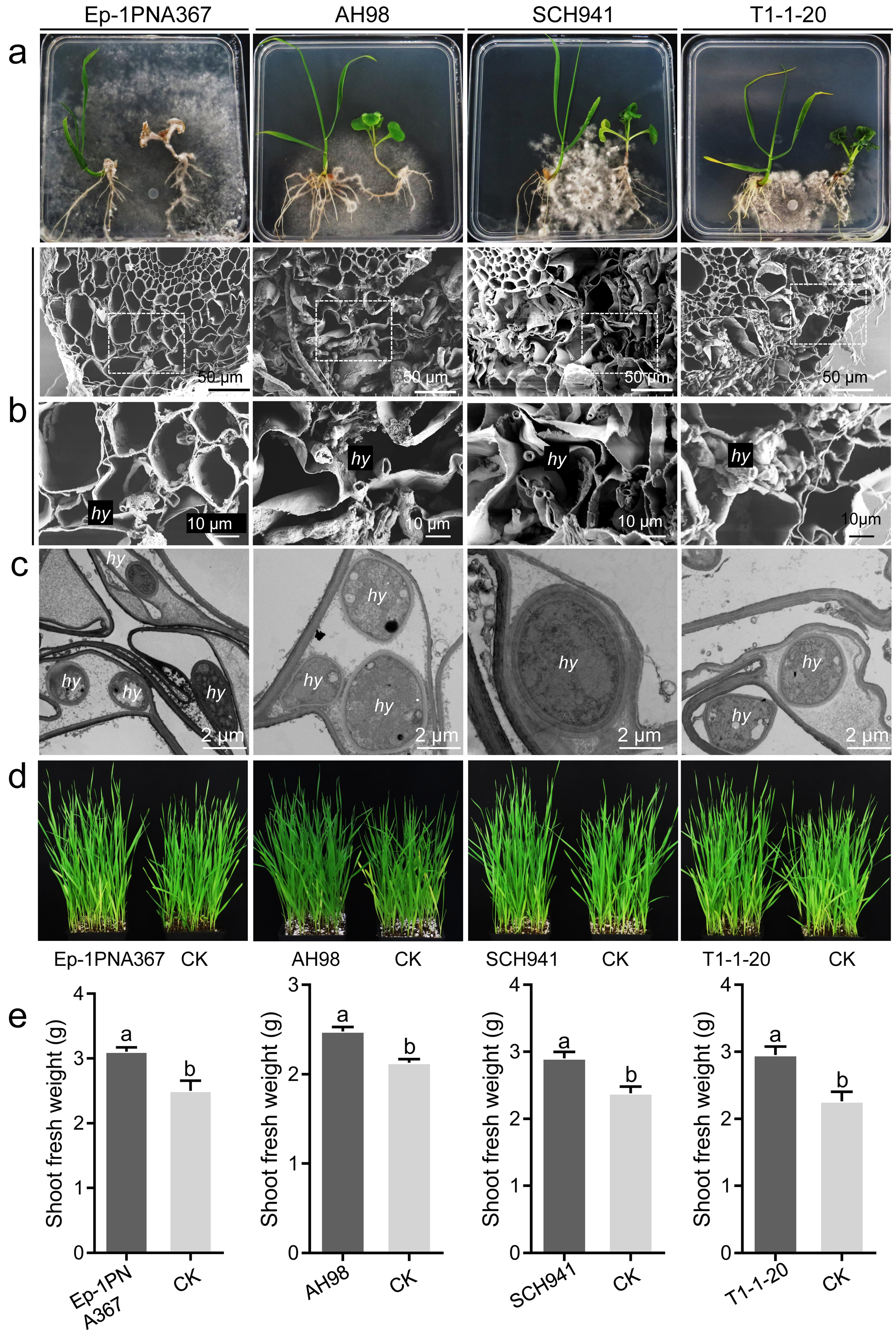

Supplement: Supplementary file 12 — Supplementary Fig. 4 [file 41396_2020_744_MOESM12_ESM.jpg]

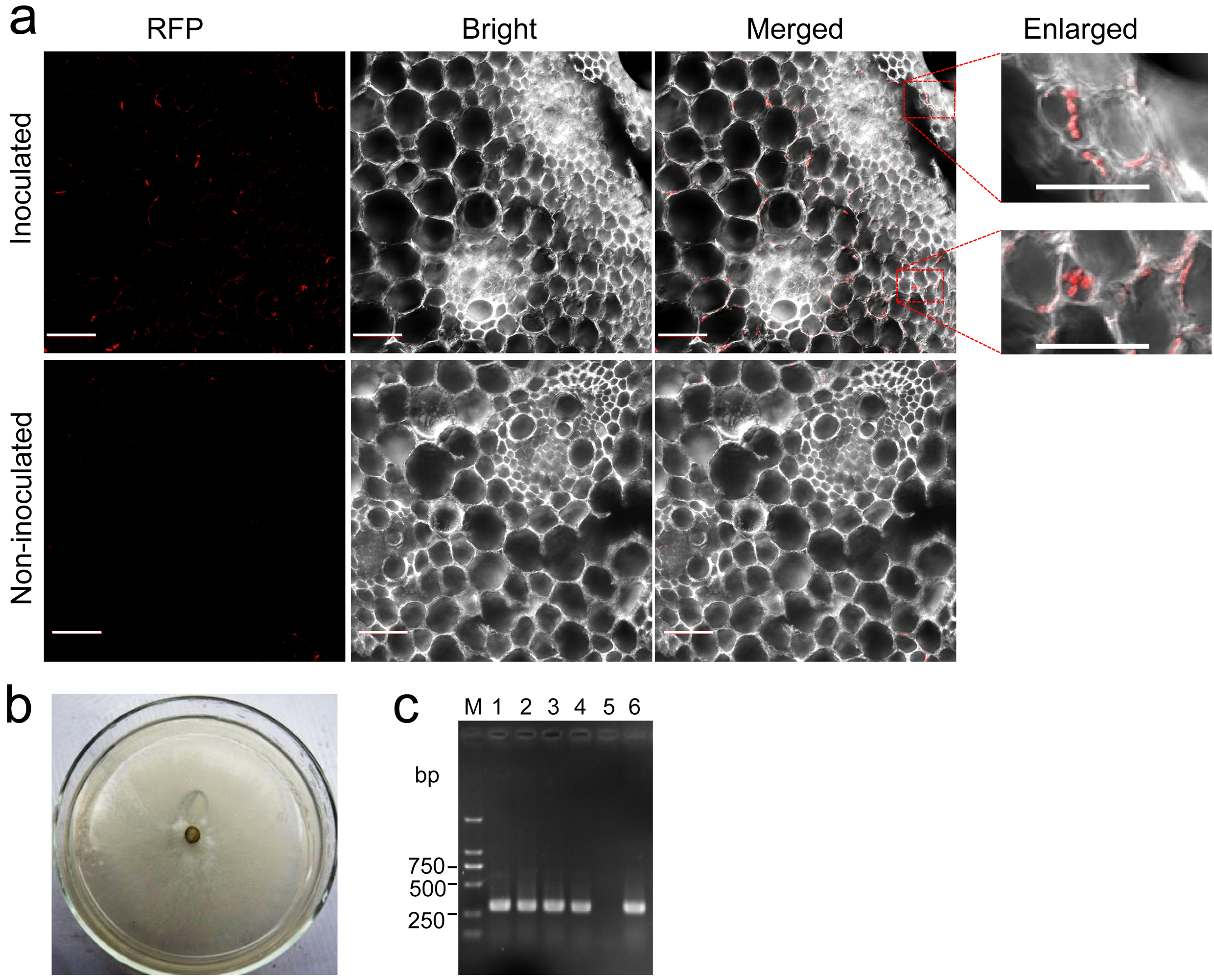

Supplement: Supplementary file 13 — Supplementary Fig. 5 [file 41396_2020_744_MOESM13_ESM.jpg]

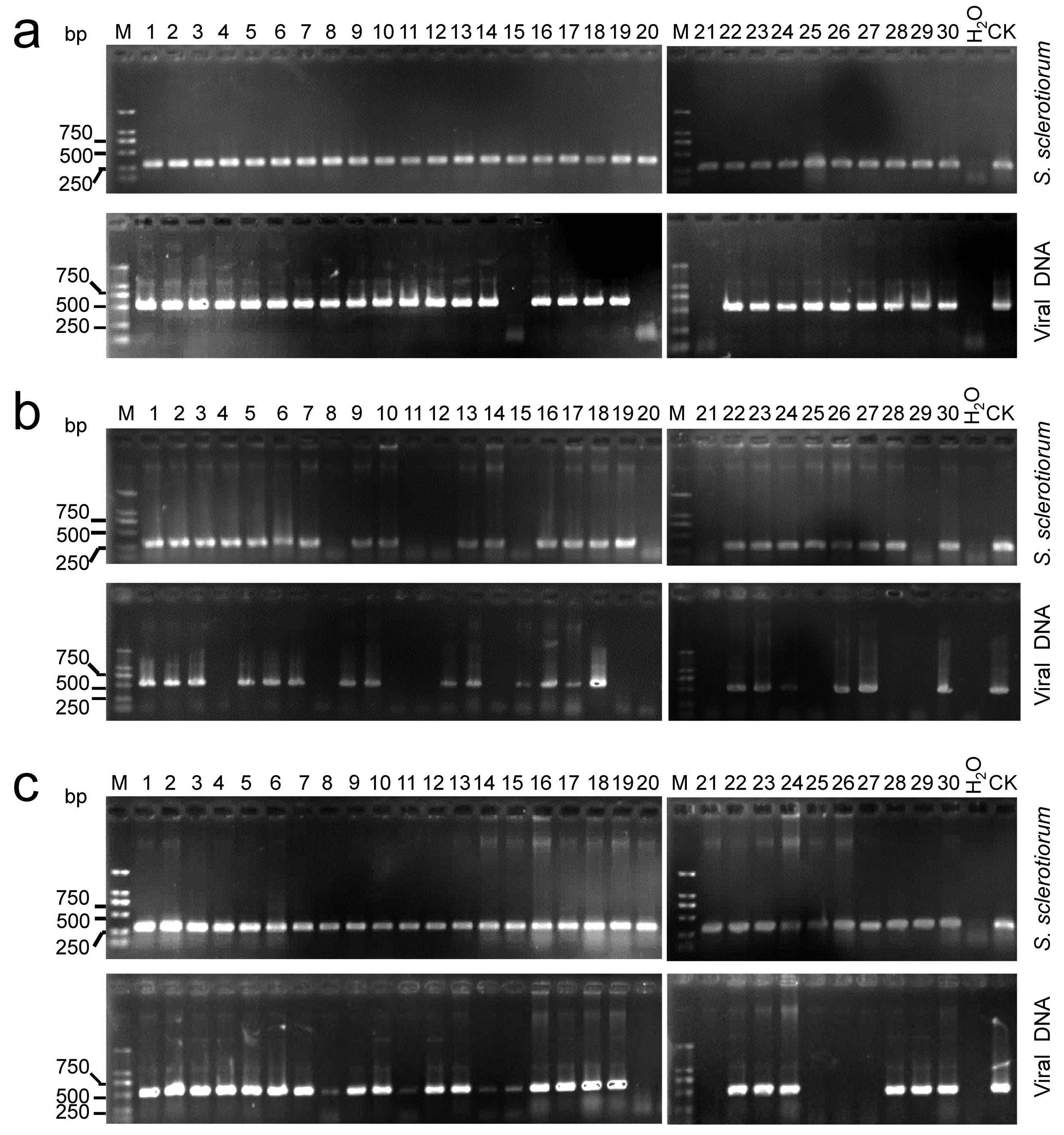

Supplement: Supplementary file 14 — Supplementary Fig. 6 [file 41396_2020_744_MOESM14_ESM.jpg]

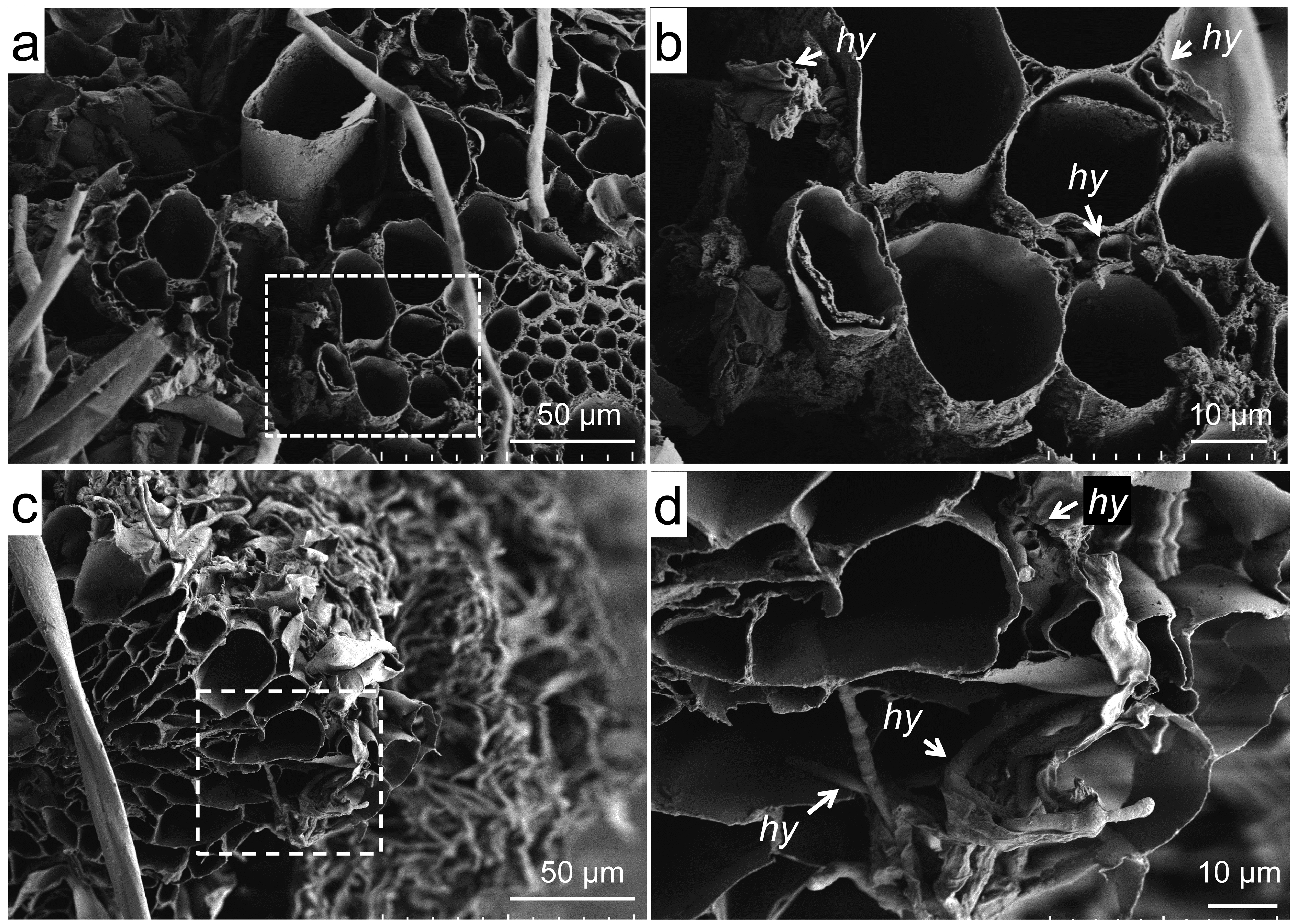

Supplement: Supplementary file 15 — Supplementary Fig. 7 [file 41396_2020_744_MOESM15_ESM.jpg]

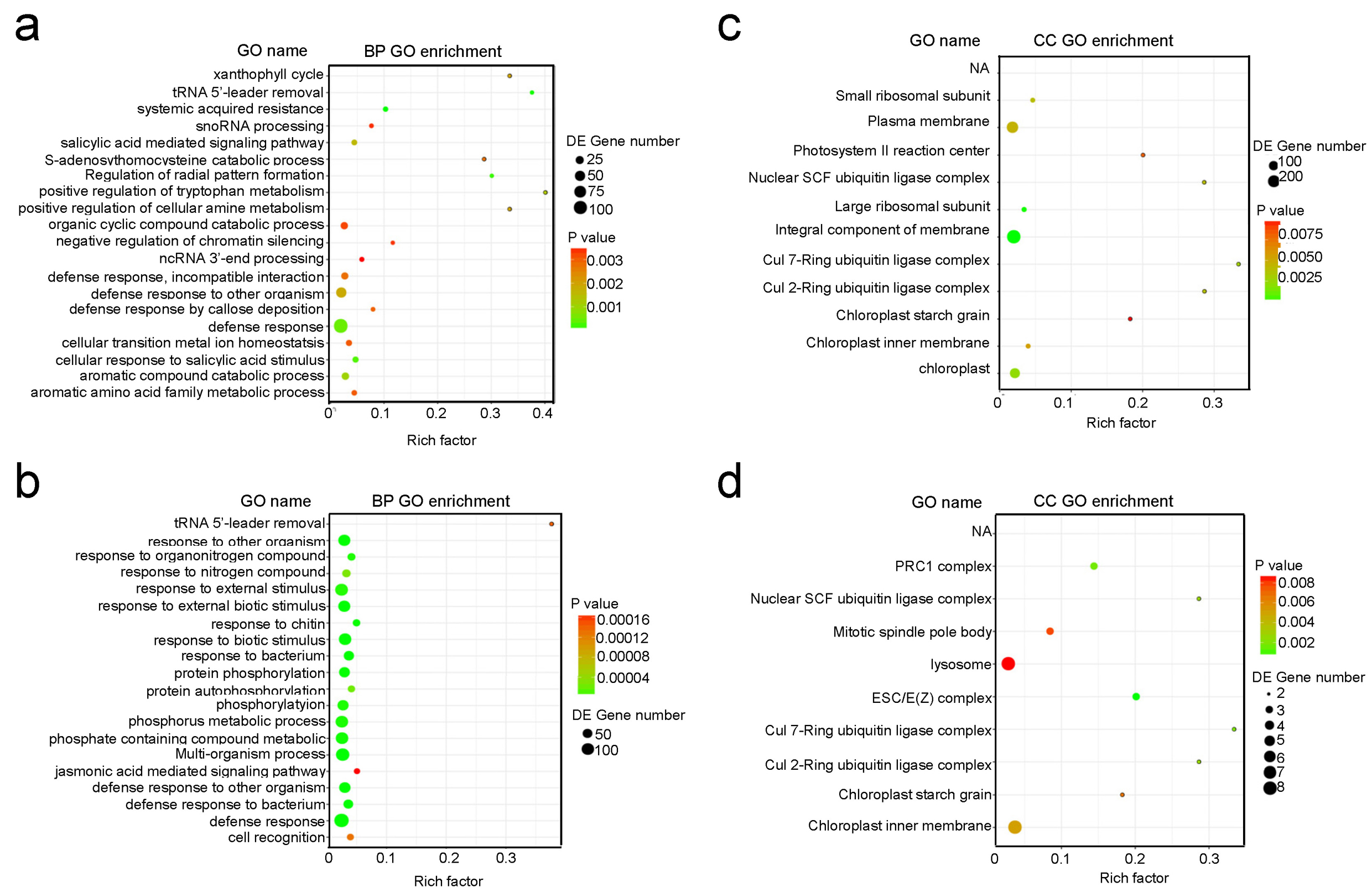

Supplement: Supplementary file 16 — Supplementary Fig. 8 [file 41396_2020_744_MOESM16_ESM.jpg]

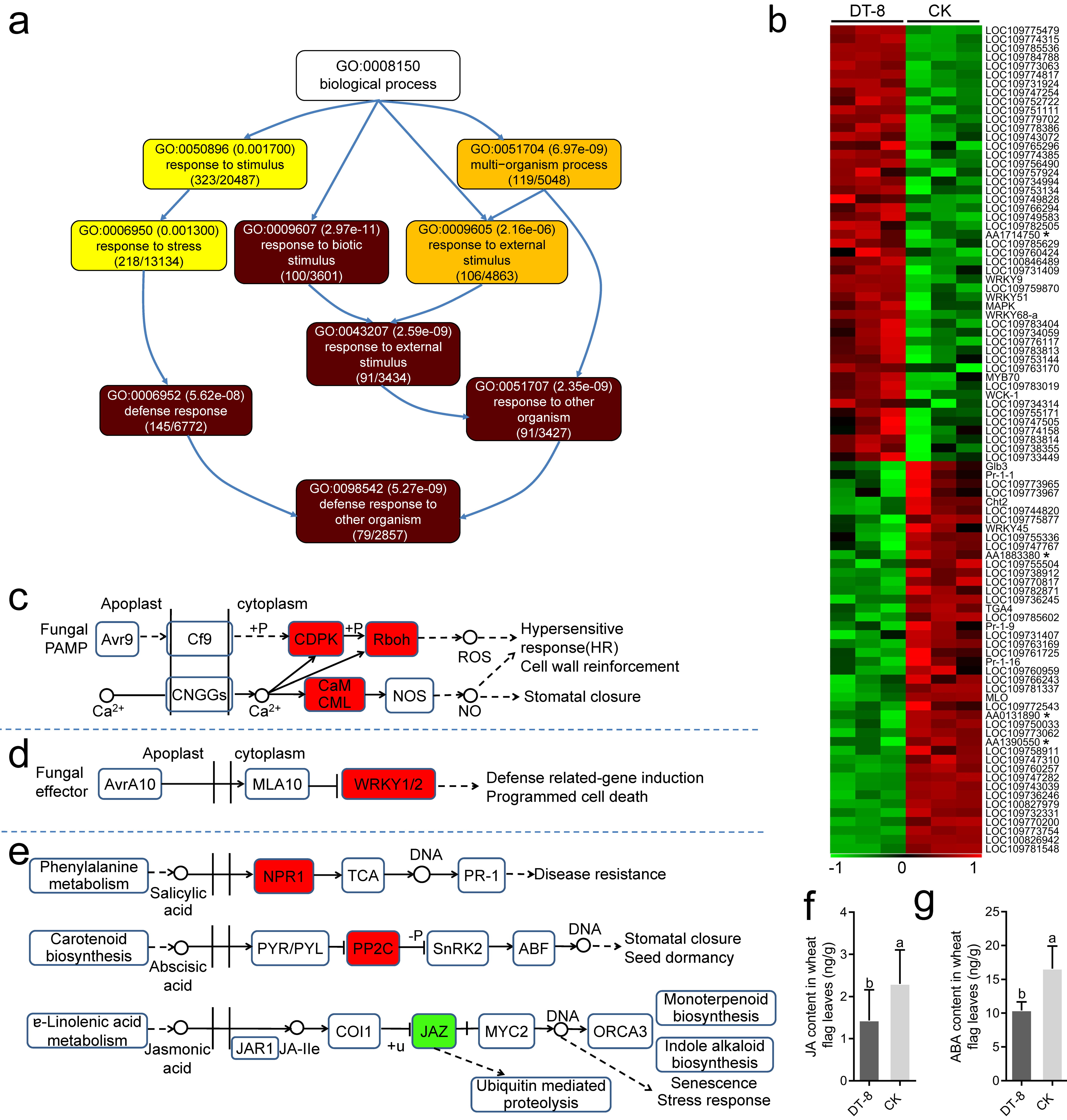

Supplement: Supplementary file 17 — Supplementary Fig. 9 [file 41396_2020_744_MOESM17_ESM.jpg]

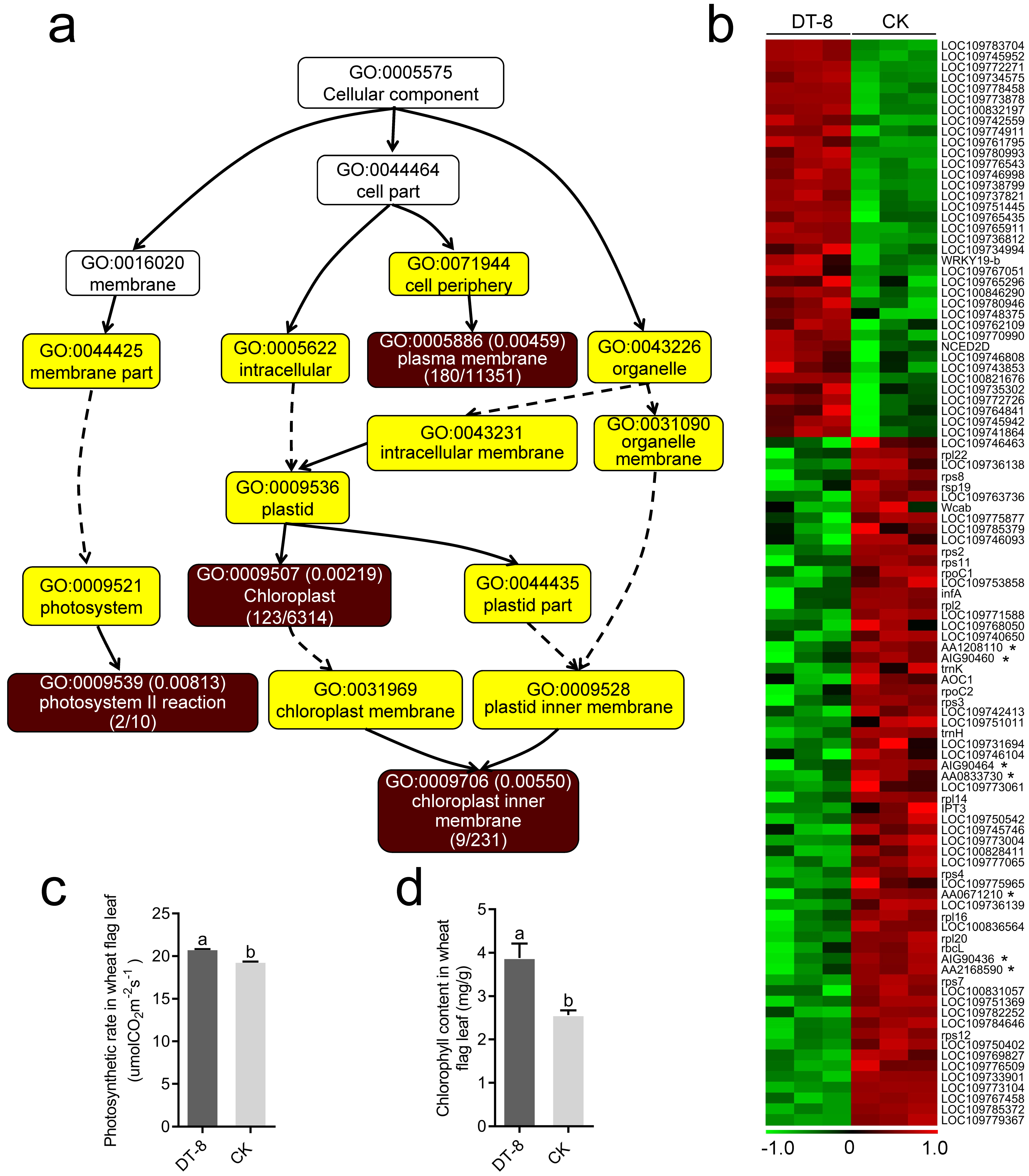

Supplement: Supplementary file 18 — Supplementary Fig. 10 [file 41396_2020_744_MOESM18_ESM.jpg]

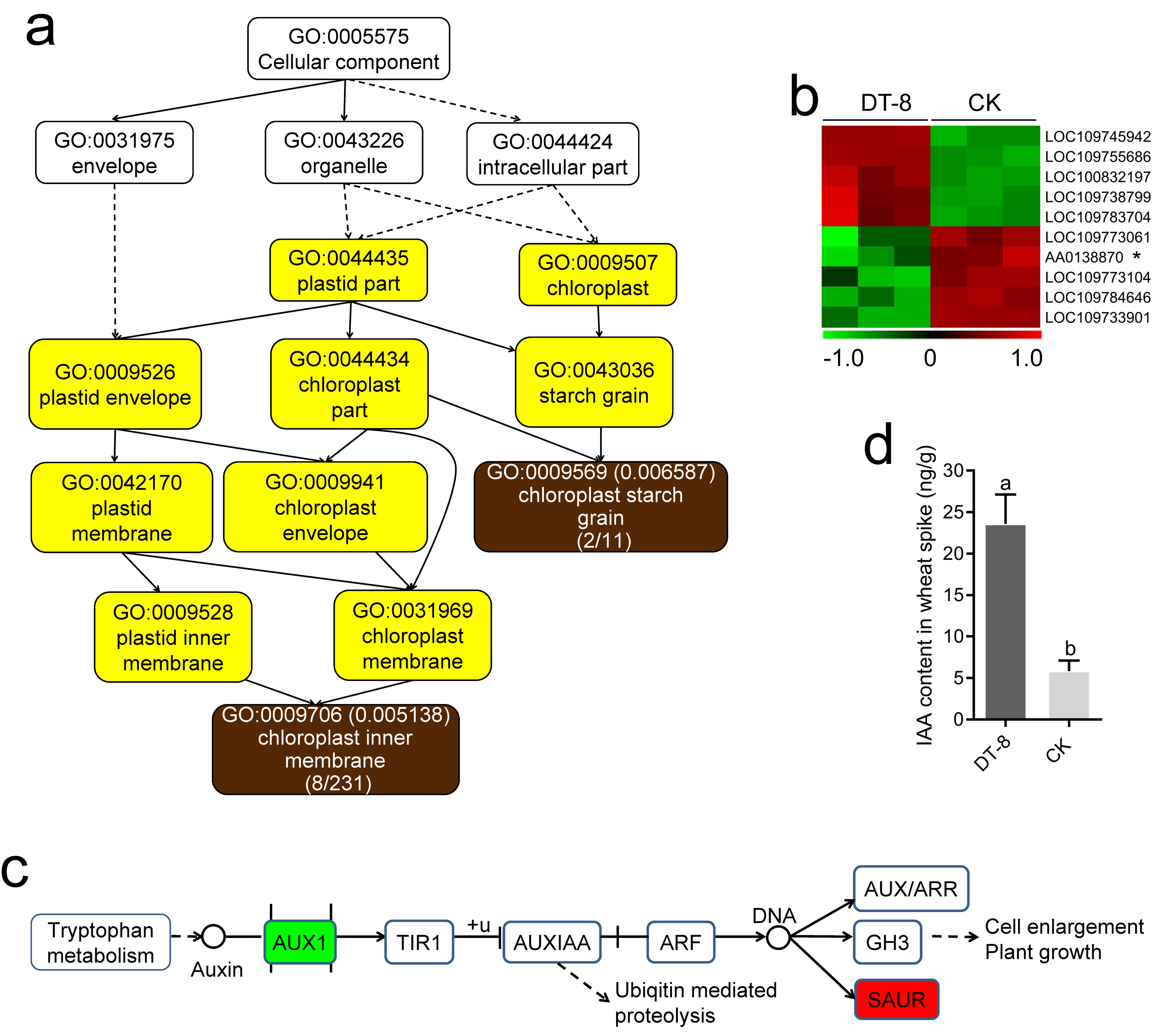

Supplement: Supplementary file 19 — Supplementary Fig. 11 [file 41396_2020_744_MOESM19_ESM.jpg]
